# Palladium-catalysed formation of vicinal all-carbon quaternary centres via propargylation

**DOI:** 10.1038/ncomms12382

**Published:** 2016-08-25

**Authors:** Xin Huang, Shangze Wu, Wangteng Wu, Pengbin Li, Chunling Fu, Shengming Ma

**Affiliations:** 1 Department of Chemistry, Laboratory of Molecular Recognition and Synthesis, Zhejiang University, Hangzhou, Zhejiang 310027, China

## Abstract

Construction of two vicinal all-carbon quaternary carbon centres is of great importance due to the common presence of such units in natural and unnatural molecules with attractive functions. However, it remains a significant challenge. Here, we have developed a palladium-catalysed general coupling for the efficient connection of two tertiary carbon atoms: Specifically, propargylic carbonate has been treated with a fully loaded soft functionalized nucleophile to connect such two fully loaded carbon atoms with a simple palladium catalyst. It is observed that the central chirality in the optically active tertiary propargylic carbonates has been remembered and transferred into the products with very high efficiency. The triple bond and the functional groups such as ester, cyano and unsaturated C–C bonds make this method a relatively general solution for such a purpose due to their high synthetic versatility.

## Abstract (web summary)

Formation of vicinal, quaternary stereocentres is challenging, but such products are commonly encountered in nature. Here, the authors report a palladium catalysed process to form vicinal quaternary carbons via propargylation, including in enantiopure form when optically active starting materials are employed.

## Graphical abstract

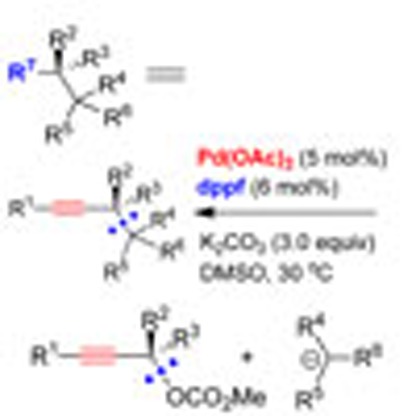

Carbon-carbon bond formation is an ever-lasting topic in organic chemistry-related sciences such as synthesis, drug discovery and materials. Of particular interest is a contiguous array of all-carbon quaternary centres, which are found in many complex natural products with remarkable biological activities^1^,^2^,^3^,^4^. However, the efficient creation of such entities by carbon-carbon bond formation reaction is still challenging^5^,^6^,^7^. The most direct approach would be the coupling reaction of two related carbon species; however, this is largely underdeveloped due to the extremely strong steric effect and the intrinsic challenge of β-H elimination forming the olefins (equations (1) and (2), Fig. 1a). For a selection of such efforts, see refs 8, 9, 10, 11. So far, the commonly notable approach is the allylic substitution reaction with an issue of regioselectivity, which is obviously still in its early stage in terms of the scope (equation (3), Fig. 1a)^12^,^13^,^14^,^15^,^16^,^17^,^18^,^19^.

Propargylic carbonates are a class of very common organic compounds with already well-established versatile attractive reactivities in organic synthesis. Since Tsuji's first report in 1985 (ref. 20), palladium-catalysed transformations of propargylic alcohols or their derivatives have become a useful tool for constructing carbon–carbon and carbon–heteroatom bonds. In most cases, these reactions give rise to the corresponding allenyl palladium species **A** via different approaches (*S*_*N*_*2*′-type oxidative addition plus *S*_*N*_*2*-type oxidative addition followed by isomerization via intermediate **B**), which was followed by the coupling reaction with a hard carbon nucleophile to form the allene derivatives or by the attack of two molecules of soft carbon nucleophiles to form the corresponding alkene derivatives (Path 1, Fig. 1b)^21^,^22^. In rare cases, the direct propargylic substitution with simple carbon nucleophiles has been reported as the minor byproducts or major products (Path 2, Fig. 1b)^23^,^24^,^25^,^26^,^27^; however, to the best of our knowledge, the propargylations of tertiary propargylic alcohol derivatives with fully loaded soft carbon nucleophiles have never been realized. We reasoned that, by applying a suitable ligand together with optimized reaction parameters, the *S*_*N*_*2*′-type oxidative addition would be shut down; with that the *S*_*N*_*2*-type oxidative addition would give η^1^-propargylic palladium **C** exclusively with the absolute configuration inverted. If such a ligand makes the palladium in **C** coordination-saturated, this intermediate would NOT isomerize to η^1^-allenylic palladium **A** mostly via the intermediacy of η^3^-dinuclear palladium intermediate **B**, as shown by Ogoshi *et al*.^28^. In addition, the intrinsic β-H elimination should also be avoided for the same reason. Finally, the highly reactive propargylic nature may make this intermediate **C** reactive enough to undergo reaction with a sterically hindered tertiary carbon nucleophile. If the stereoselectivity may be controlled, an efficient, highly stereoselective connection of two tertiary carbon atoms may be fulfilled. As an extra bonus, the synthetic potential of the C–C triple bond is attractive, since it may undergo further deliberate synthetic elaboration for different purposes as compared with the allylic approach shown in equation (3) of Fig. 1a.

In this paper, we wish to report our recent realization of such a concept: the palladium-catalysed reaction of tertiary propargylic carbonates with different fully-loaded carbon nucleophiles to form such carbon–carbon bonds, which nicely remembers the central chirality in the starting propargylic carbonates by applying dppf as the proper ligand.

## Results

### Optimization of the reaction

**Figure 1:**
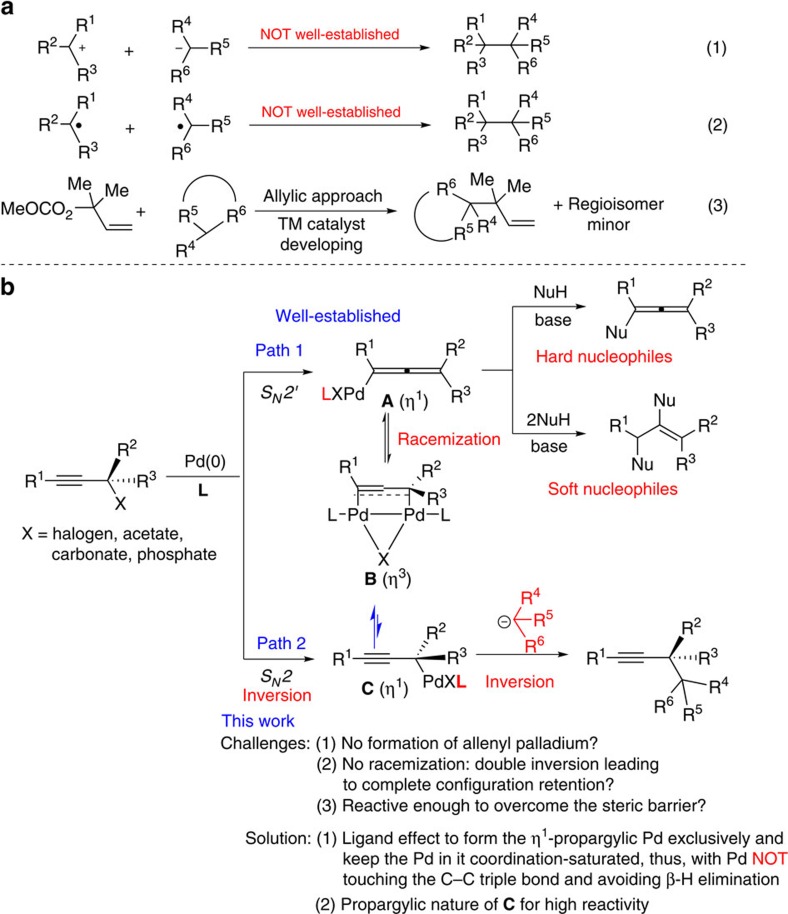
Formation of vicinal all-carbon quaternary centres. (**a**) Previous works. (**b**) This work: new concept for vicinal all-carbon quaternary centres.

Initially we were studying the coupling cyclization reactions of 2-(2,3-butadienyl)malonitrile **2a** with propargylic carbonate **1a** for the synthesis of allene compounds **5aa** and **6aa** (ref. 29). After many trial and error methods with different ligands, Pd catalysts and other reaction parameters, we failed to observe the formation of the expected products **5aa** and **6aa** from the reaction of methyl (2-methyl-4-phenylbut-3-yn-2-yl) carbonate **1a** (0.3 mmol) with 2-(buta-2,3-dien-1-yl)malononitrile **2a** (1.5 equiv.) under the catalysis of Pd(OAc)_2_ (5 mol%) and a ligand (6 mol%) with K_2_CO_3_ (3.0 equiv.) at 30 °C in dimethylsulphoxide (DMSO) for 6 h (entries 1–7, Table 1). However, when we used dppe, dppb and BINAP as the ligand, surprisingly, the reaction afforded the targeted propargylic substitution product **3aa** in Fig. 1 with two continuous carbon centres, albeit in very low yields, exclusively (entries 2, 4 and 5, Table 1). Encouraged by this exciting discovery of the formation of two vicinal all-carbon quaternary centres, the influence of the critical reaction parameters was investigated and it was found that **3aa** was formed in 94% isolated yield by running the reaction with dppf, a very bulky ligand (entry 8, Table 1)! After screening a series of solvents, DMSO was still proved to be the best: the reactions in tetrahydrofuran, CH_3_CN, dimethylformamide or dimethylacetamide all afforded lower yields of **3aa** with some recovery of **1a** (entries 11–14, Table 1). Thus, the conditions presented in entry 8 of Table 1 have been chosen as the standard for further study.

### Substrate scope

With the optimized conditions in hand (entry 8, Table 1), we first examined the reactivity of various tertiary propargylic carbonates **1** with 2-(buta-2,3-dien-1-yl)malononitrile **2a**. The substrates with the R^1^ substituent of the alkyne unit being aryl bearing either electron-deficient or electron-rich groups could all proceed smoothly, affording the expected 1,6-allenyne products in 85–90% yields (**3aa**–**3da**); moreover, the method could be extended to 3-thienyl or *n*-Bu-substituted propargylic carbonates in 84% yield (**3ea**, **3fa**). In addition, the carbonates where R^2^, R^3^ bore different groups could also be introduced into this transformation in good yields (**3ga**–**3ia**). It is worthy of mention that the reaction afforded the corresponding products **3ja** and **3ka** in moderate yields when cyclic tertiary propargylic carbonates **1j** and **1k** (see Supplementary Information for the structures) were applied. The practicality of this reaction has been demonstrated by running the reaction of **1a** on a 10 mmol scale (**3aa**) and **1j** on an 8.4 mmol scale (**3ja**). We next explored the scope of functionalized tertiary carbon nucleophiles under optimized conditions. For the 2,3-allenyl malononitriles, we could introduce the *n*-pentyl group at the 2-position (**3lb**) or the *Cy* group at the 4-position (**3mc**); to our delight, different propargylic malononitriles could be applicable to this reaction, affording the 1,6-diyne products in 84–92% yields (**3ad**–**3of**); when various allyl malononitriles were used, the 1,6-enyne products were obtained in excellent yields (**3ag**–**3pi**); we also prepared the 1,5-diphenylhexyne derivatives **3aj** in 94% yield and **3gj** in 92% yield with 2-benzylmalononitrile used as the nucleophile. In addition, the method is also proper to a C_10_-alkyl-substituted malononitrile (**3ak**). Besides the malononitriles, the tertiary carbon nucleophiles could also be substituted methyl 2-cyanoacetates (**3al**–**3an**) and dimethyl malonate (**3ao** and **3ap**).

## Discussion

**Figure 2 f2:**
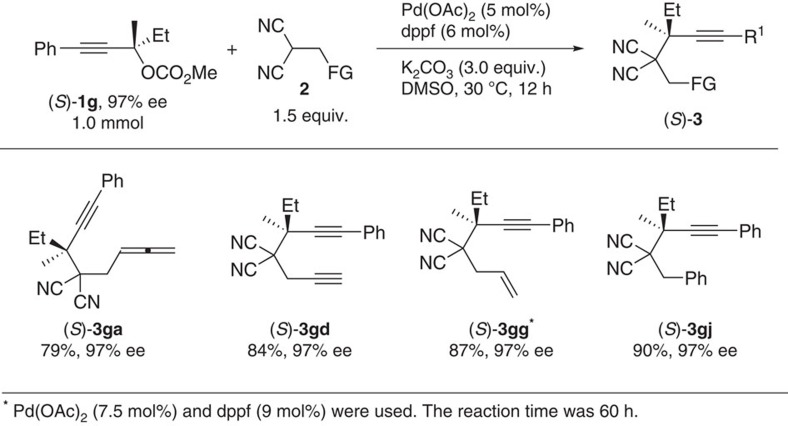
Highly entioselective synthesis by chirality transfer. Excellent efficiency of chirality transfer was found when using (*S*)-**1g**.

In order to unveil [Fig f2][Fig f3]the mechanism (Fig. 4), the reactions of (*S*)-**1g** (refs 30, 31) (97% ee), which has a chiral quaternary carbon centre, with different functionalized malononitriles were tried. To our surprise, all the corresponding products, (*S*)-**3ga**, (*S*)-**3gd**, (*S*)-**3gg** and (*S*)-**3gj**, were formed with the absolute configuration of the propargylic carbon atom remaining intact and with essentially no loss of enantiomeric purity (Fig. 2).

**Figure 3 f3:**
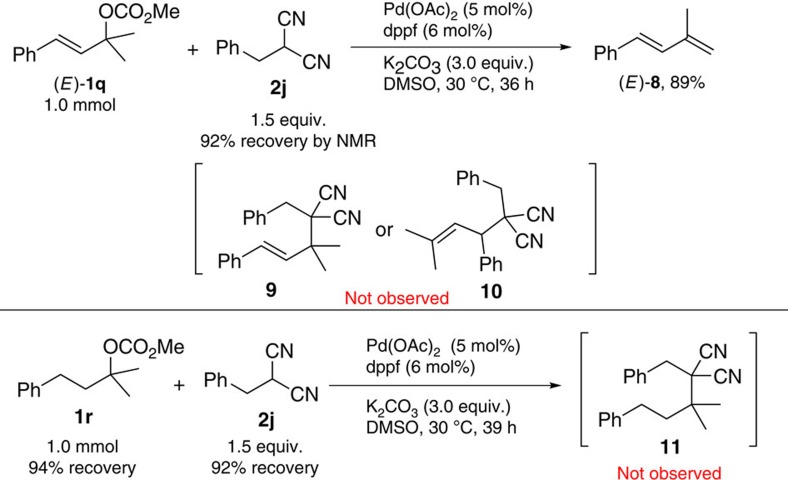
The unique role of the carbon–carbon triple bond. (*E*)-**1q** and **1r** failed to afford the corresponding vicinal all-carbon quaternary centres.

In order to demonstrate the role of the unique carbon–carbon triple bond, some control experiments were conducted: (1) the reaction of tertiary allylic carbonate (*E*)-**1q** with 2-benzylmalononitrile **2j** under the standard conditions failed to afford the expected allyl nucleophilic product **9** or **10**. Instead, the β-elimination product, that is, conjugated diene (*E*)-**8**, was afforded in 89% yield. (2) On replacing carbonate (*E*)-**1q** with saturated tertiary carbonate **1r**, no reaction occurred (Fig. 3). Thus, it is concluded that the carbon–carbon triple bond in **1** plays a unique role in this nucleophilic substitution reaction.

**Figure 4:**
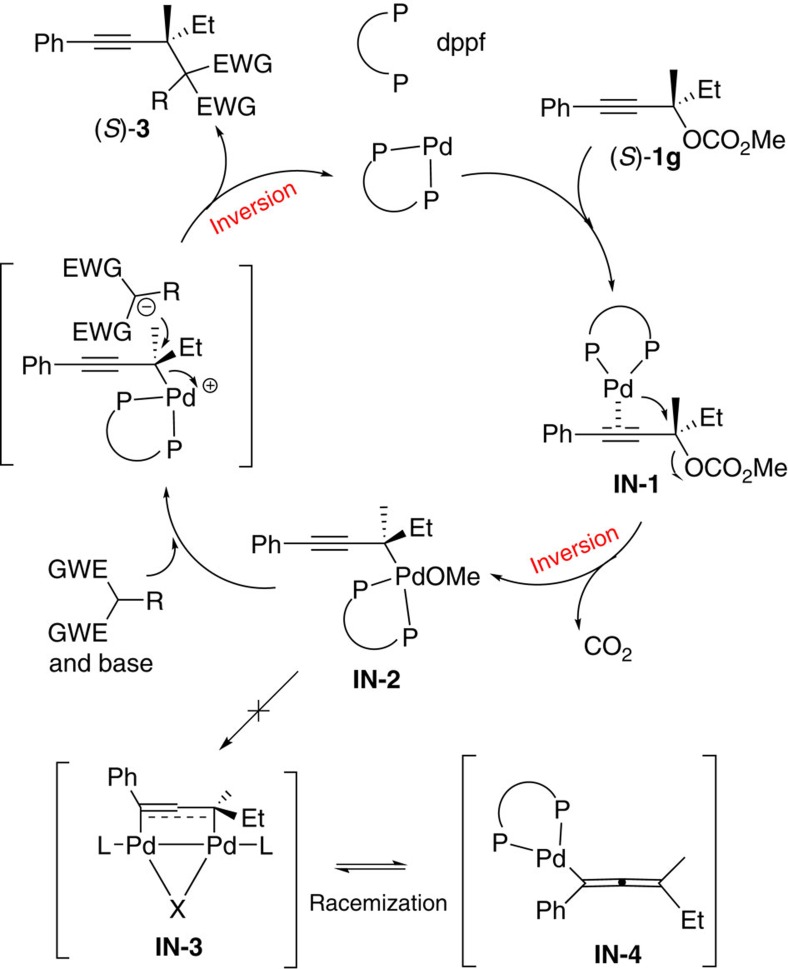
A plausible mechanism. Dppf helps to keep the tetra-dentated palladium coordination saturated avoiding racemization.

Based on these results, we propose a possible mechanism, as shown in Fig. 4. The coordination of the C–C triple bond in (*S*)-**1g** with Pd(0) leads to the formation of coordination complex **IN-1**. Oxidative addition of the chiral tertiary propargylic carbonate (*S*)-**1g** with Pd(0) would afford the propargyl η^1^-palladium intermediate **IN-2** with the first inversion of the absolute configuration of the propargylic carbon atom. Dppf helps to keep this tetra-dentated palladium without forming η^3^-dinuclear palladium intermediate **IN-3**, which avoids any possible racemization through the σ–π–σ isomerization process^28^. Subsequently, the *in-situ*-generated soft carbon nucleophile would attack this chiral carbon atom from the back side of the C–Pd bond to afford the final nucleophilic substitution product (*S*)-**3** with the absolute configuration inverted for the second time and the regeneration of the catalytically active Pd(0). Overall, the absolute configuration of this propargylic carbon atom remains NOT inverted. Of course, the complete exclusion of allenyl palladium intermediate **IN-3** is NOT possible, although such an option may most likely lead to at least partial racemization (see also Fig. 2)^32^.

**Figure 5:**
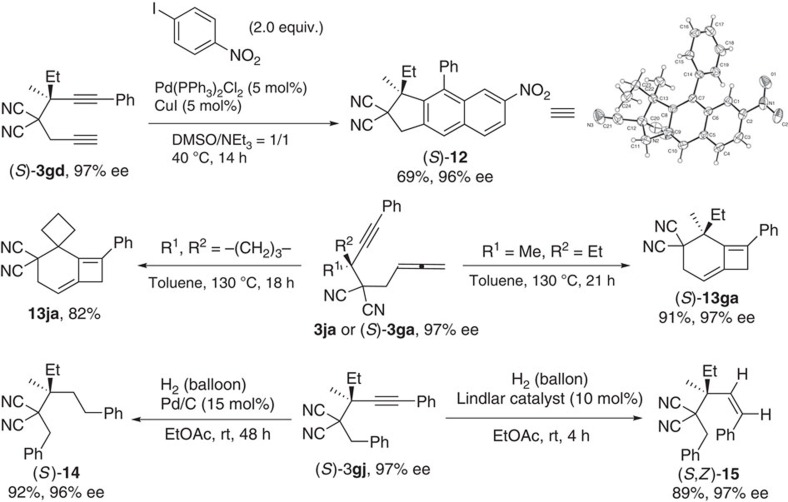
The synthetic applications. Several molecules with two continuous all-carbon quaternary carbons centres were afforded.

Such propargylation products have been demonstrated to be very useful in synthesis of different molecules with two continuous all-carbon quaternary carbon centres in high efficiency, as shown in Fig. 5: (1) Tricyclic 1*H*-cyclopenta[*b*]naphthalene derivative (*S*)-**12** was prepared from a tandem reaction of (*S*)-**3gd** and 1-iodo-4-nitrobenzene^33^. In fact, the absolute configuration of the chiral propargylation products (*S*)-**3ga**, (*S*)-**3gd**, (*S*)-**3gg** and (*S*)-**3gj** has been established on the basis of an X-ray single-crystal diffraction study of this product. (2) The 1,6-allenyne derivative **3ja** and (*S*)-**3ga** could go through a thermal [2+2] reaction^34^,^35^ to construct the tricyclic product **13ja** containing two highly strained four-membered rings and bicyclic product (*S*)-**13ga** containing one highly strained four-membered ring in very high yields. (3) In addition, the hydrogenation or semi-hydrogenation of the carbon–carbon triple bond in (*S*)-**3gj** afforded saturated (*S*)-**14** under 15 mol% of Pd/C catalyst or (*S,Z*)-**15** with a *Z*-double bond under 10 mol% of the Lindlar catalyst, respectively^36^,^37^.

In conclusion, we have developed a new concept of a palladium-catalysed propargylation of functionalized tri-substituted carbon nucleophiles with tertiary propargylic carbonates, which provides a very practical and useful method to construct a C–C bond between two tert-carbon atoms forming different 1,6-allenyne, 1,6-diyne, 1,6-enyne and 1,6-arenyne derivatives, which are important types of versatile compounds in organic chemistry-related disciplines. Moreover, no racemization occurred to the central chirality in the tertiary propargylic carbonates, which makes enantioselective synthesis of such molecules possible. Due to the presence of the triple bond and the demonstrated scope of the substrates, this reaction expands the scope of the existing methodologies for the construction of two vicinal all-carbon quaternary centres. Further studies on expanding the scope of the reaction, the mechanism, synthetic applications and development of enantioselective version of this reaction are being actively pursued in our laboratory.

## Methods

### Materials

DMSO was stirred with CaH_2_ for 12 h at 80 °C and distilled *in vacuo* before use. Pd(OAc)_2_ was purchased from Acros. Dppf was purchased from Energy Chemical. K_2_CO_3_ was purchased from Sinopharm Chemical Reagent Co., Ltd. Pd/C was purchased from Alfa Aesar. Lindlar catalyst was purchased from J&K. Other commercially available chemicals were purchased and used without additional purification unless noted otherwise.

### General spectroscopic methods

^1^H nuclear magnetic resonance (NMR) spectra were recorded on a Bruker-300 MHz spectrometer and ^13^C NMR spectra were recorded at 75 MHz. All ^1^H NMR experiments were measured with tetramethylsilane (0 p.p.m.) or the signal of residual CHCl_3_ (7.26 p.p.m.) in CDCl_3_ as the internal reference; ^13^C NMR experiments were measured relative to the signal of CDCl_3_ (77.0 p.p.m.). Infrared spectra were recorded from films of pure samples on sodium chloride plates for liquid samples or in the form of KBr discs for solid samples. Mass and HRMS spectra were carried out in EI mode. Elemental analysis was carried out by Elementar Vario MICRO cube. Thin-layer chromatography was performed on pre-coated glass-back plates and visualized with UV light at 254 nm. Flash column chromatography was performed on silica gel. ^1^H NMR, ^13^C NMR and HPLC spectra (for chiral compounds) are supplied for all compounds (see Supplementary Figs 1–137). See Supplementary Methods for the characterization data of compounds not listed in this part.

### Synthesis of 3aa

To a flame-dried Schlenk tube containing K_2_CO_3_ (414.2 mg, 3.0 mmol) were added Pd(OAc)_2_ (11.4 mg, 0.05 mmol), dppf (33.4 mg, 0.06 mmol), **1a** (217.5 mg, 1.0 mmol)/DMSO (8.0 ml) and **2a** (178.0 mg, 1.5 mmol)/DMSO (2.0 ml) sequentially under nitrogen atmosphere. The reaction was complete after being stirred at 30 °C for 11 h as monitored by TLC (eluent: petroleum ether/ethyl acetate=20/1). After cooling to room temperature (rt), the resulting mixture was quenched with an aqueous solution of diluted hydrochloric acid (*v/v*=10%, prepared via diluting commercially available concentrated hydrochloric acid with water according to the volume ratio; 10 ml) slowly and extracted with ethyl ether (30 ml × 3). The combined organic layer was washed with water and brine, and dried over anhydrous Na_2_SO_4_. After filtration and evaporation of the solvent, chromatography on silica gel (eluent: petroleum ether/ethyl acetate=20/1) afforded **3aa** (226.4 mg, 89%) as a liquid: ^1^H NMR (300 MHz, CDCl_3_) *δ* 7.46–7.38 (m, 2H, ArH), 7.36–7.25 (m, 3H, ArH), 5.40–5.28 (m, 1H, CH=), 4.95 (dt, *J*_1_=6.7 Hz, *J*_2_=2.4 Hz, 2H, =CH_2_), 2.82 (dt, *J*_1_=7.6 Hz, *J*_2_=2.3 Hz, 2H, CH_2_), 1.63 (s, 6H, Me × 2); ^13^C NMR (75 MHz, CDCl_3_) *δ* 210.5, 131.6, 128.7, 128.2, 121.5, 113.9, 87.9, 85.9, 83.0, 77.1, 48.7, 39.4, 33.7, 25.7; IR (neat, cm^−1^) 3,063, 2,985, 2,942, 2,869, 2,245, 2,221, 1,956, 1,598, 1,491, 1,470, 1,459, 1,443, 1,393, 1,373, 1,291, 1,252, 1,161, 1,091, 1,071, 1,028; MS (EI): *m/z* (%) 260 (M^+^, 100); HRMS calcd. for C_18_H_16_N_2_ (M^+^): 260.1313; found: 260.1310.

### Synthesis of (*S*)-3ga

Following the procedure for the synthesis of **3aa**, the reaction of K_2_CO_3_ (413.7 mg, 3.0 mmol), Pd(OAc)_2_ (11.4 mg, 0.05 mmol), dppf (33.3 mg, 0.06 mmol), (*S*)-**1g** (97% ee, 233.2 mg, 1.0 mmol)/DMSO (8.0 ml) and **2a** (177.5 mg, 1.5 mmol)/DMSO (2.0 ml) at 30 °C for 12 h afforded (*S*)-**3ga** (217.5 mg, 79%) as a liquid (eluent: petroleum ether/ethyl acetate=20/1): 97% ee (HPLC conditions: Chiralcel IC column, hexane/*i*-PrOH=200/1, 0.6 ml/min, *λ*=214 nm, *t*_R_(major)=35.3 min, *t*_R_(minor)=37.8 min); [*α*]_D_^20^=+32.2 (*c*=1.055, CHCl_3_); ^1^H NMR (300 MHz, CDCl_3_) *δ* 7.49–7.39 (m, 2H, ArH), 7.38–7.26 (m, 3H, ArH), 5.41–5.28 (m, 1H, =CH), 4.95 (dt, *J*_1_=6.5 Hz, *J*_2_=2.3 Hz, 2H, =CH_2_), 2.94–2.75 (m, 2H, CH_2_), 2.11–1.95 (m, 1H, one proton of CH_2_), 1.86–1.71 (m, 1H, one proton of CH_2_), 1.56 (s, 3H, Me), 1.21 (t, *J*=7.4 Hz, 3H, Me); ^13^C NMR (75 MHz, CDCl_3_) *δ* 210.7, 131.7, 128.8, 128.3, 121.7, 114.1, 87.3, 86.7, 83.1, 77.1, 49.3, 44.1, 33.7, 30.7, 21.7, 9.5; IR (neat, cm^−1^) 3,062, 2,978, 2,941, 2,883, 2,247, 2,228, 1,956, 1,598, 1,491, 1,461, 1,443, 1,386, 1,321, 1,250, 1,131, 1,091, 1,070; MS (EI): *m/z* (%) 274 (M^+^, 100); HRMS calcd. for C_19_H_18_N_2_ (M^+^): 274.1470; Found: 274.1469.

### Synthesis of (*S*)-12

To a flame-dried Schlenk tube were added Pd(PPh_3_)_2_Cl_2_ (17.6 mg, 0.025 mmol), CuI (4.9 mg, 0.025 mmol), 1-iodo-4-nitrobenzene (249.3 mg, 1.0 mmol), (*S*)-**3gd** (97% ee, 130.2 mg, 0.5 mmol)/DMSO (2.0 ml) and NEt_3_ (2.0 ml) sequentially under nitrogen atmosphere. The reaction was complete after being stirred at 40 °C for 14 h as monitored by TLC (eluent: petroleum ether/ethyl acetate=10/1). After cooling to rt, the resulting mixture was diluted with ethyl acetate (30 ml) and washed with water (20 ml). The organic layer was separated and the aqueous layer was extracted with ethyl acetate (20 ml). The combined organic layer was washed with brine and dried over anhydrous Na_2_SO_4_. After filtration and evaporation, the residue was purified by chromatography (eluent: petroleum ether/ethyl acetate/dichloromethane=10/1/1) on silica gel to afford (*S*)-**12** (129.5 mg, 69%) as a solid: 96% ee (HPLC conditions: Chiralcel OD-H column, hexane/*i*-PrOH=80/20, 1.0 ml min^−1^, *λ*=214 nm, *t*_R_(major)=9.5 min, *t*_R_(minor)=10.3 min); [*α*]_D_^20^=−33.9 (*c*=0.975, CHCl_3_); m.p. 205–206 °C (*n*-hexane/DCM); ^1^H NMR (300 MHz, CDCl_3_) *δ* 8.26–8.18 (m, 2H, ArH), 8.00–7.93 (m, 1H, ArH), 7.90 (s, 1H, ArH), 7.62–7.49 (m, 3H, ArH), 7.34–7.22 (m, 2H, ArH), 3.93 (dd, *J*_1_=16.4 Hz, *J*_2_=1.1 Hz, 1H, one proton of CH_2_), 3.84 (dd, *J*_1_=16.5 Hz, *J*_2_=1.2 Hz, 1H, one proton of CH_2_), 1.95–1.78 (m, 1H, one proton of CH_2_), 1.58–1.39 (m, 1H, one proton of CH_2_), 1.42 (s, 3H, Me), 0.98 (t, *J*=7.5 Hz, 3H, Me); ^13^C NMR (75 MHz, CDCl_3_) *δ* 145.7, 141.9, 139.2, 138.0, 135.4, 135.3, 133.1, 130.4, 130.1, 129.1, 128.9, 128.50, 128.46, 123.7, 123.1, 119.9, 115.3, 114.8, 57.3, 44.8, 41.6, 32.3, 22.9, 9.2; IR (KBr, cm^−1^) 3,078, 3,057, 2,978, 2,931, 2,883, 2,249, 1,628, 1,610, 1,584, 1,531, 1,490, 1,460, 1,442, 1,391, 1,377, 1,339, 1,287, 1,266, 1,218, 1,090, 1,054, 1,028; MS (EI): *m/z* (%) 381 (M^+^, 51.55), 352 (100); anal. calcd. for C_24_H_19_N_3_O_2_ (%): C 75.57, H 5.02, N 11.02; found: C 75.60, H 5.05, N 11.19.

### Synthesis of 13ja

To a flame-dried Schlenk tube was added **3ja** (273.2 mg, 1.0 mmol)/toluene (10 ml) under nitrogen atmosphere. The reaction was complete after being stirred at 130 °C for 18 h as monitored by TLC (eluent: petroleum ether/ethyl acetate=20/1). After cooling to rt and evaporation of the solvent, the residue was purified by chromatography (eluent: petroleum ether/ethyl acetate/dichloromethane=20/1/1) on silica gel to afford **13ja** (223.1 mg, 82%) as a solid: m.p. 133–135 °C (*n*-hexane/DCM); ^1^H NMR (300 MHz, CDCl_3_) *δ* 7.54–7.29 (m, 5H, ArH), 5.17 (t, *J*=3.9 Hz, 1H, =CH), 3.32 (s, 2H, CH_2_), 2.86 (d, *J*=3.9 Hz, 2H, CH_2_), 2.80–2.43 (m, 4H, CH_2_ × 2), 2.40–2.21 (m, 2H, CH_2_); ^13^C NMR (75 MHz, CDCl_3_) *δ* 142.1, 139.1, 138.5, 133.4, 128.7, 128.6, 127.2, 115.2, 102.0, 44.6, 42.4, 35.9, 31.9, 28.6, 14.0; IR (KBr, cm^−1^) 3,056, 2,991, 2,941, 2,914, 2,247, 1,754, 1,595, 1,490, 1,445, 1,435, 1,353, 1,321, 1,246, 1,180, 1,157, 1,097, 1,033; MS (EI): *m/z* (%) 272 (M^+^, 39.83), 244 (100); anal. calcd. for C_19_H_16_N_2_ (%): C 83.79, H 5.92, N 10.29; found: C 83.58, H 5.85, N 10.25.

### Synthesis of 14

To a dry Schlenk tube were added Pd/C (dry, *w/w* (Pd)=10%, 32.2 mg, 0.03 mmol) and **3gj** (63.0 mg, 0.2 mmol)/EtOAc (4 ml) sequentially. The resulting mixture was frozen with a liquid nitrogen bath, degassed and refilled with H_2_ for three times. Then the reaction was allowed to stir at rt with a H_2_ balloon. After 48 h, the reaction was completed as monitored by TLC (petroleum ether/ethyl acetate=20/1). The mixture was filtrated through a short column of silica gel with EtOAc (20 ml × 3). After evaporation, the residue was purified by chromatography (eluent: petroleum ether/ethyl acetate=15/1) on silica gel to afford **14** (57.1 mg, 89%) as a solid: m.p. 112–113 °C (hexane/DCM); ^1^H NMR (300 MHz, CDCl_3_): *δ* 7.50–7.15 (m, 10H, ArH), 3.13 (s, 2H, CH_2_), 2.86–2.64 (m, 2H, CH_2_), 2.12–1.70 (m, 4H, CH_2_ × 2), 1.34 (s, 3H, Me), 1.14 (t, *J*=7.4 Hz, 3H, Me); ^13^C NMR (75 MHz, CDCl_3_) *δ* 141.2, 132.7, 130.5, 128.7, 128.63, 128.58, 128.2, 126.3, 115.0, 49.7, 43.5, 38.4, 38.0, 30.8, 29.0, 21.5, 9.0; IR (KBr, cm^−1^) 3,087, 3,066, 3,024, 2,975, 2,949, 2,881, 2,239, 1,599, 1,498, 1,472, 1,455, 1,435, 1,386, 1,239, 1,090, 1,031, 1,010; MS (EI): *m/z* (%) 316 (M^+^, 2.17), 91 (100); anal. calcd. for C_22_H_24_N_2_ (%): C 83.50, H 7.64, N 8.85; found: C 83.41, H 7.72, N 8.75.

### Synthesis of (*S*,*Z*)-15

Following procedure for the synthesis of **14**, the reaction of lindlar catalyst (palladium on calcium carbonate, *w/w* (Pd)=5%, poisoned with lead acetate, 42.5 mg, 0.02 mmol), (*S*)-**3gj** (62.0 mg. 0.2 mmol) and H_2_ in EtOAc (4.0 ml) at rt for 4 h afforded (*S,Z*)-**15** (55.7 mg, 89%) as a solid (eluent: petroleum ether/ethyl acetate=30/1): 97% ee (high-performance liquid chromatography (HPLC) conditions: Chiralcel AD-H column, hexane/*i*-PrOH=200/1, 0.6 ml min^−1^, *λ*=214 nm, *t*_R_(minor)=35.5 min, *t*_R_(major)=41.2 min); [*α*]_D_^20^=+142.7 (*c*=0.96, CHCl_3_); m.p. 112–113 °C (hexane/dichloromethane (DCM)); ^1^H NMR (300 MHz, CDCl_3_): *δ* 7.47–7.23 (m, 8H, ArH), 7.22–7.14 (m, 2H, ArH), 7.09 (d, *J*=12.9 Hz, 1H, CH=), 5.62 (d, *J*=12.9 Hz, 1H, CH=), 3.22 (d, *J*=13.2 Hz, 1H, one proton of CH_2_), 3.11 (d, *J*=13.2 Hz, 1H, one proton of CH_2_), 2.04–1.88 (m, 1H, one proton of CH_2_), 1.81–1.67 (m, 1H, one proton of CH_2_), 1.02 (t, *J*=7.4 Hz, 3H, Me), 0.98 (s, 3H, Me); ^13^C NMR (75 MHz, CDCl_3_) *δ* 137.4, 135.9, 132.8, 130.4, 130.1, 128.8, 128.5, 128.0, 127.8, 127.1, 114.60, 114.56, 51.1, 49.4, 38.8, 31.8, 18.8, 9.0; IR (KBr, cm^−1^) 3,062, 3,033, 2,974, 2,941, 2,881, 2,242, 1,599, 1,492, 1,456, 1,442, 1,388, 1,124, 1,090, 1,071, 1,025; MS (EI): *m/z* (%) 314 (M^+^, 0.13), 159 (100); anal. calcd. for C_22_H_22_N_2_ (%): C 84.04, H 7.05, N 8.91; found: C 83.95, H 7.11, N 8.78.

### Data availability

The authors declare that all the data supporting the findings of this study are available within the article and its Supplementary Information files.

## Additional information

**How to cite this article:** Huang, X. *et al*. Palladium-catalysed formation of vicinal all-carbon quaternary centres via propargylation. *Nat. Commun.* 7:12382 doi: 10.1038/ncomms12382 (2016).

## Supplementary Material

Supplementary Information Supplementary Figures 1-137, Supplementary Methods and Supplementary References

## Figures and Tables

**Table 1:**
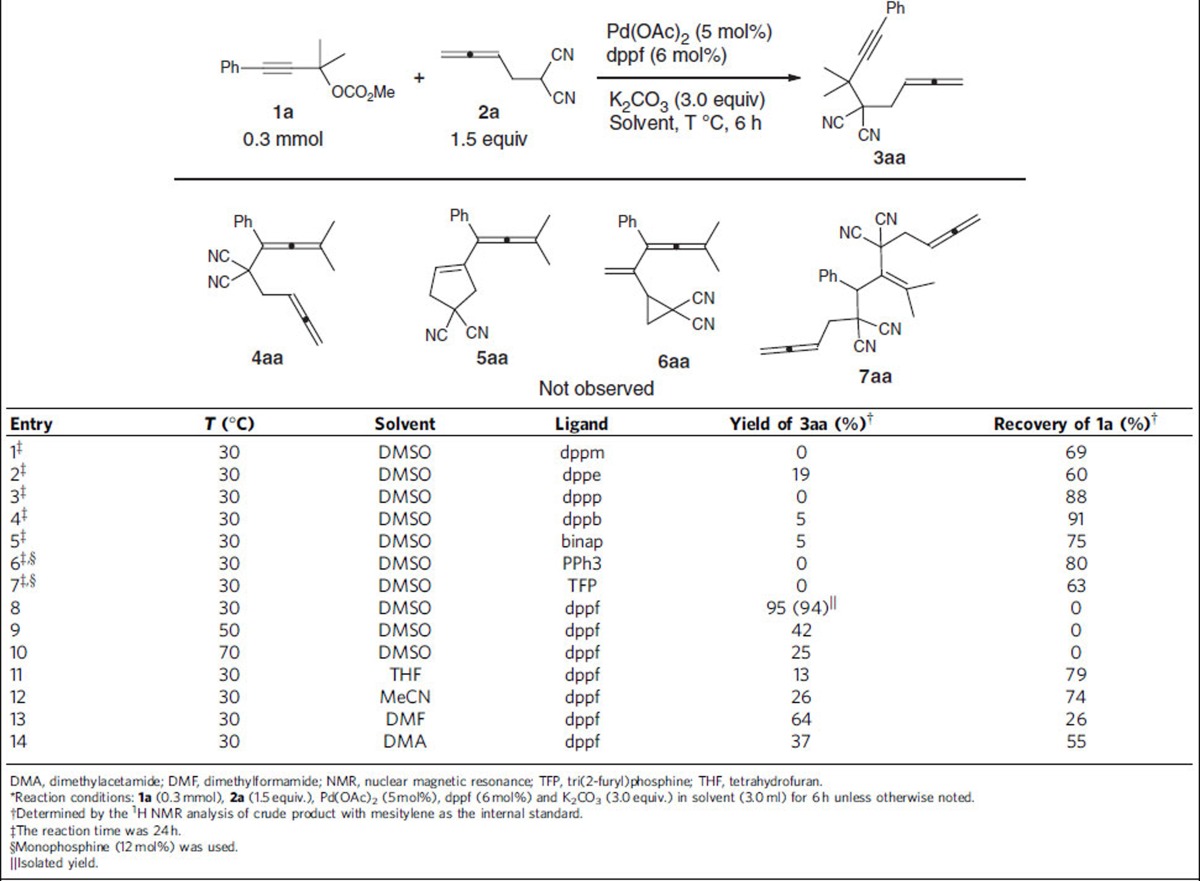
Optimization of reaction conditions*.

**Table 2 t2:**
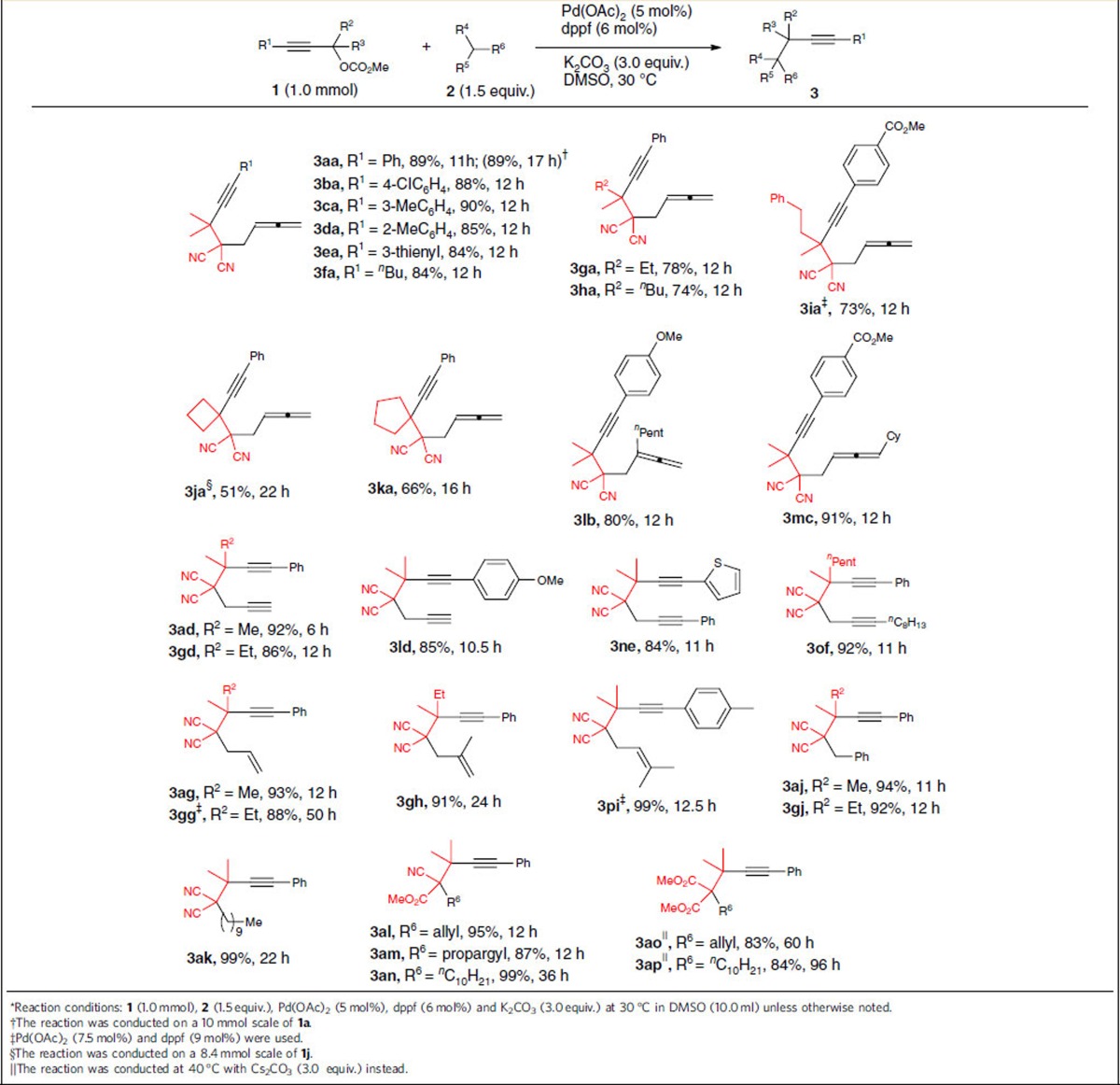
The scope of the tertiary propargylic carbonates and tri-substituted carbon nucleophiles*^†^^‡^^§^^||^.
