# Supplementary material for: Palladium-catalysed formation of vicinal all-carbon quaternary centres via propargylation
Source: Nat Commun. 2016 Aug 25;7:12382. doi: 10.1038/ncomms12382 (PMC5007298; doi:10.1038/ncomms12382)
Supplement: Supplementary Information — Supplementary Figures 1-137, Supplementary Methods and Supplementary References [file ncomms12382-s1.pdf]

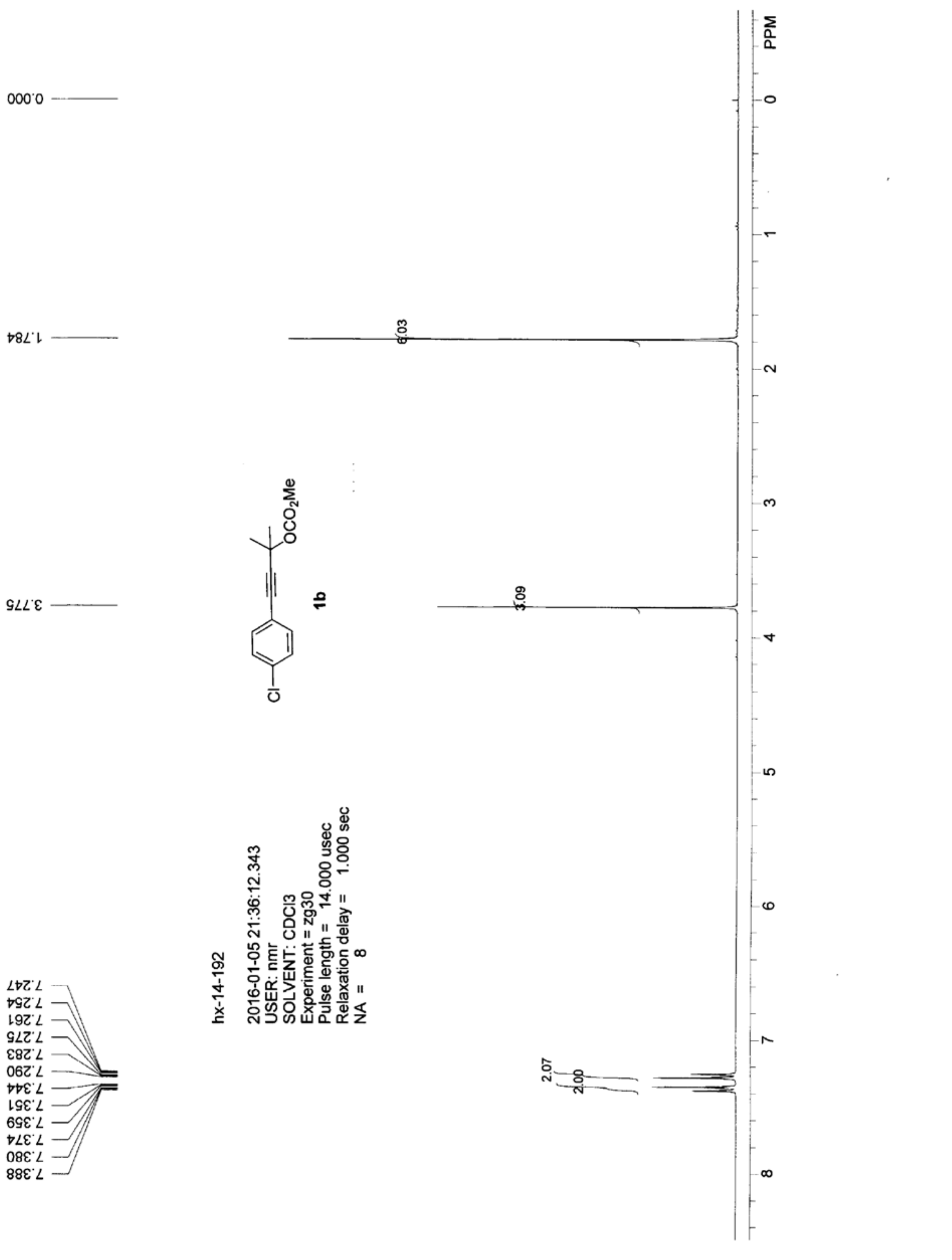

Supplementary Figure 1. **^{1}H NMR (300 MHz, $CDCl_3$) spectrum for 1b.**

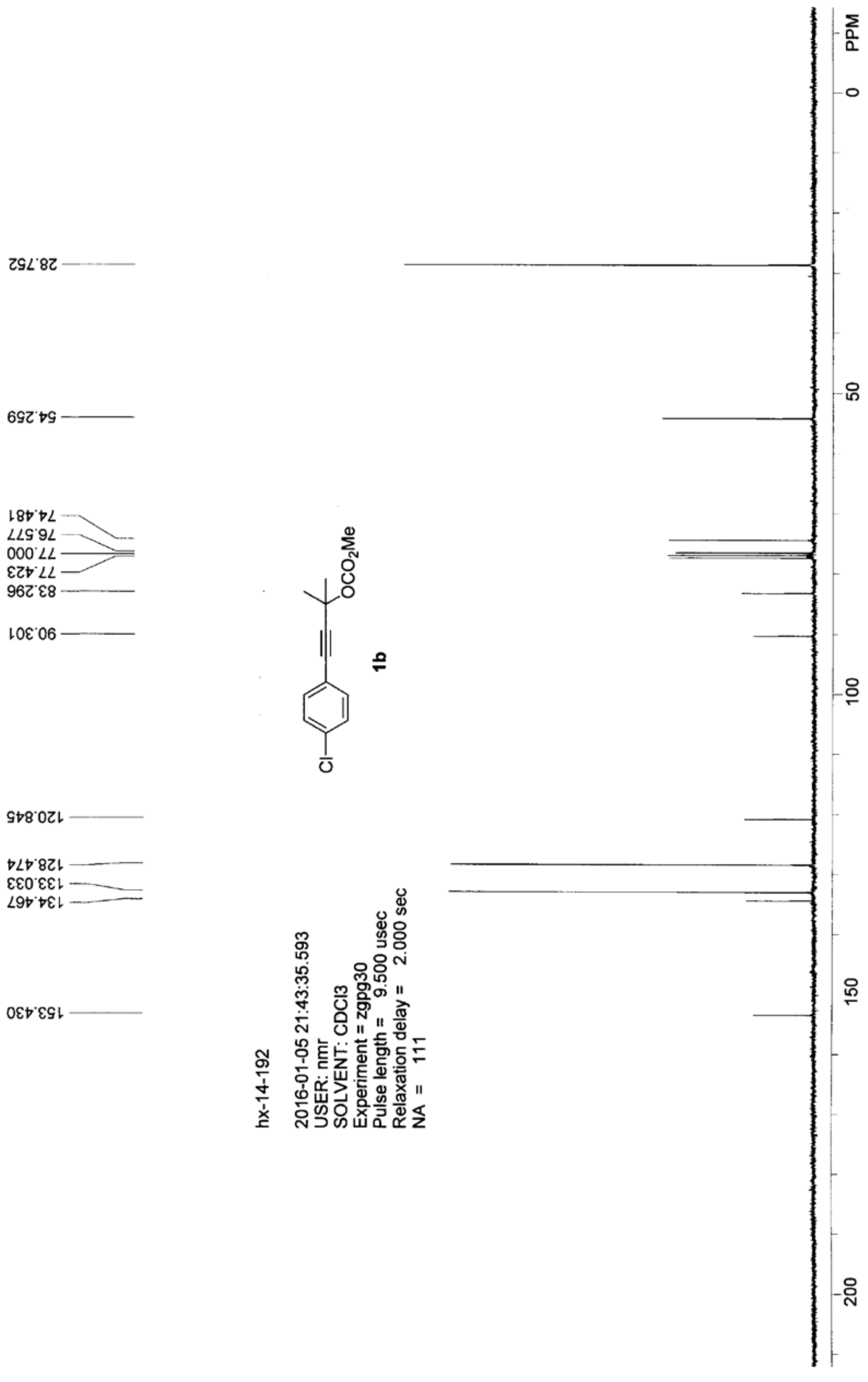

Supplementary Figure 2. ^{13}**C NMR (75 MHz,** $CDCl_3$**) spectrum for 1b.**

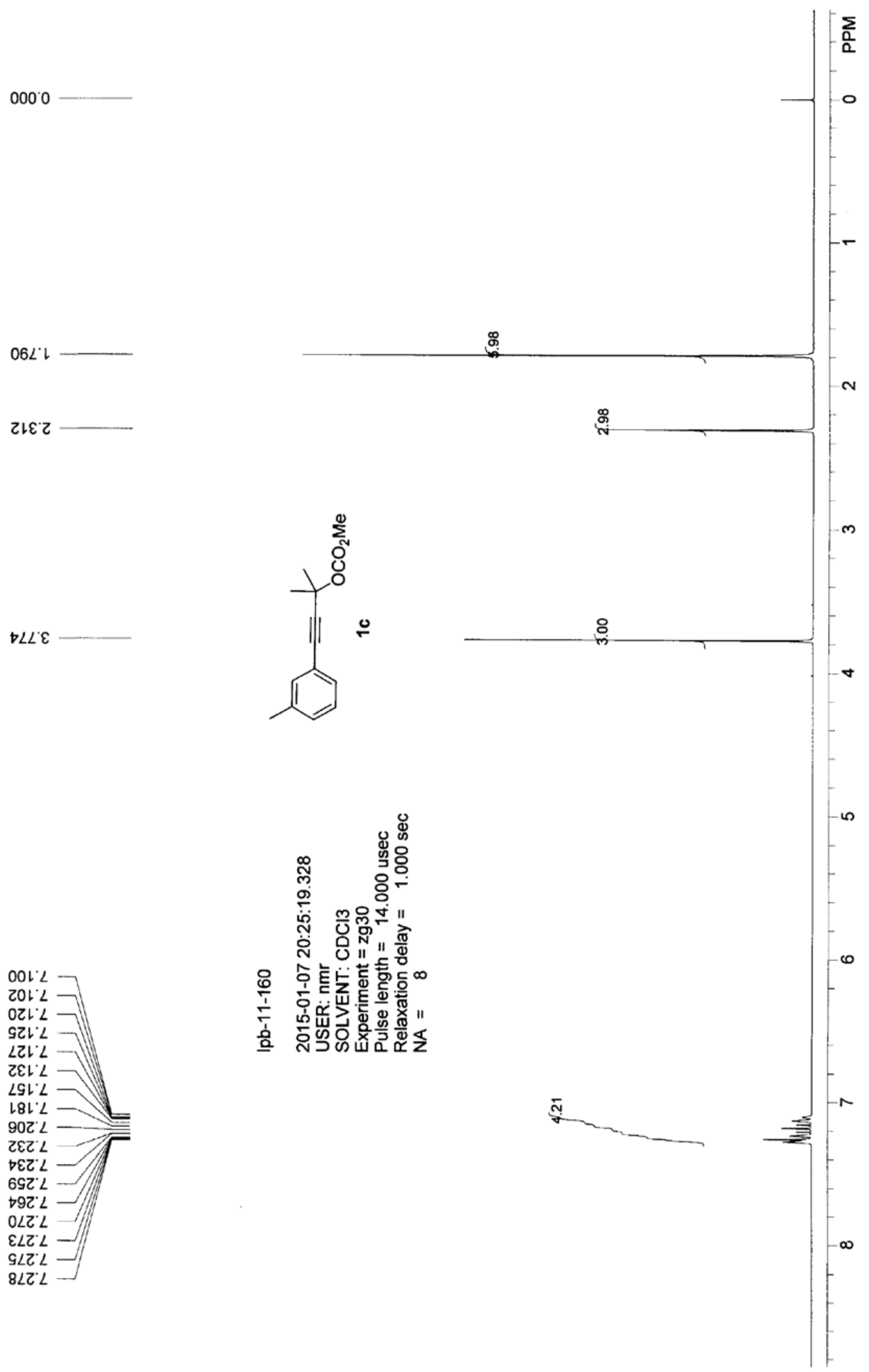

Supplementary Figure 3. ^{1}**H NMR (300 MHz,** $CDCl_3$**) spectrum for 1c.**

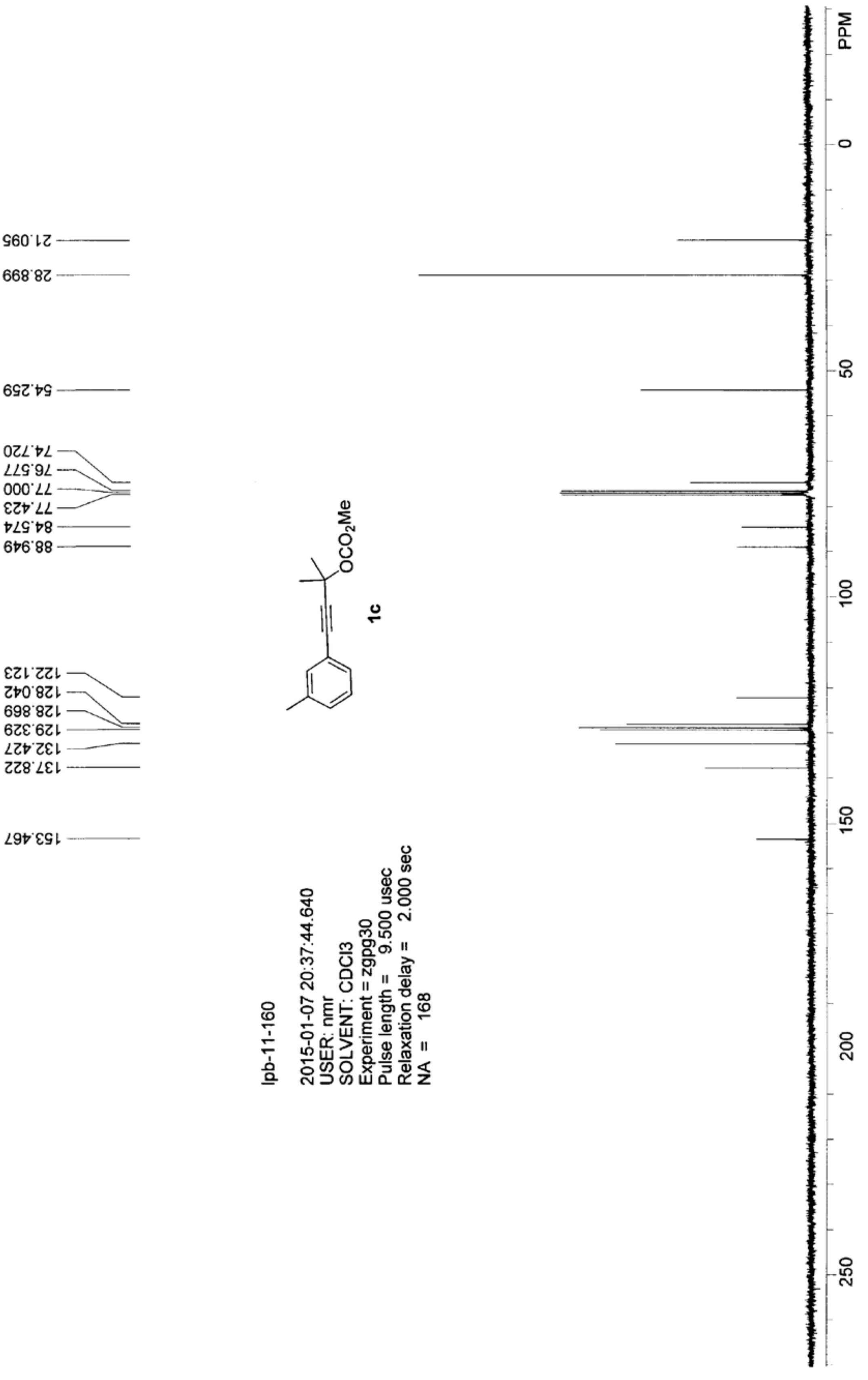

Supplementary Figure 4. ^{13}**C NMR (75 MHz, CDCl$_3$) spectrum for 1c.**

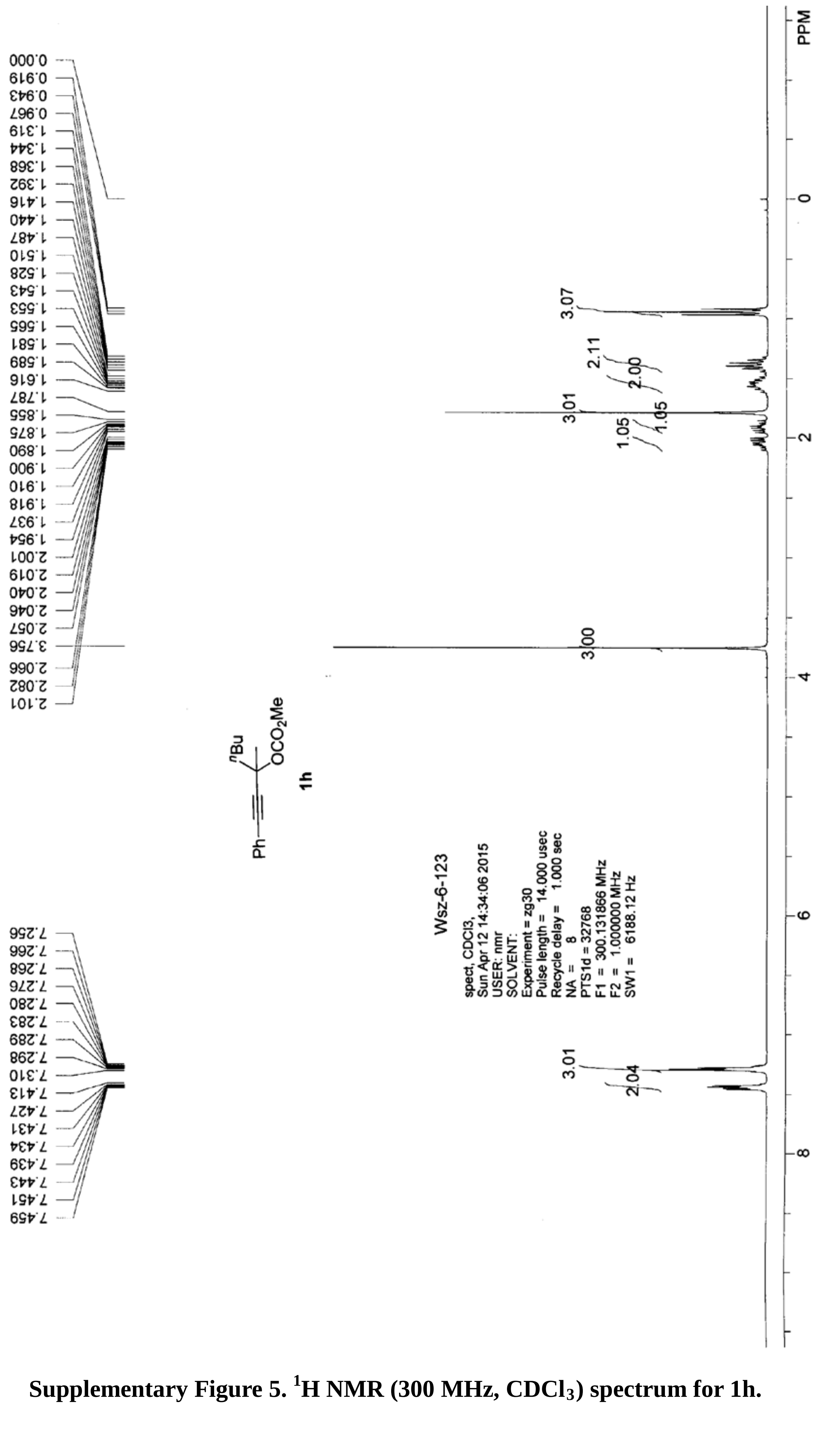

Supplementary Figure 5. ^{1}H NMR (300 MHz, $CDCl_3$) spectrum for 1h.

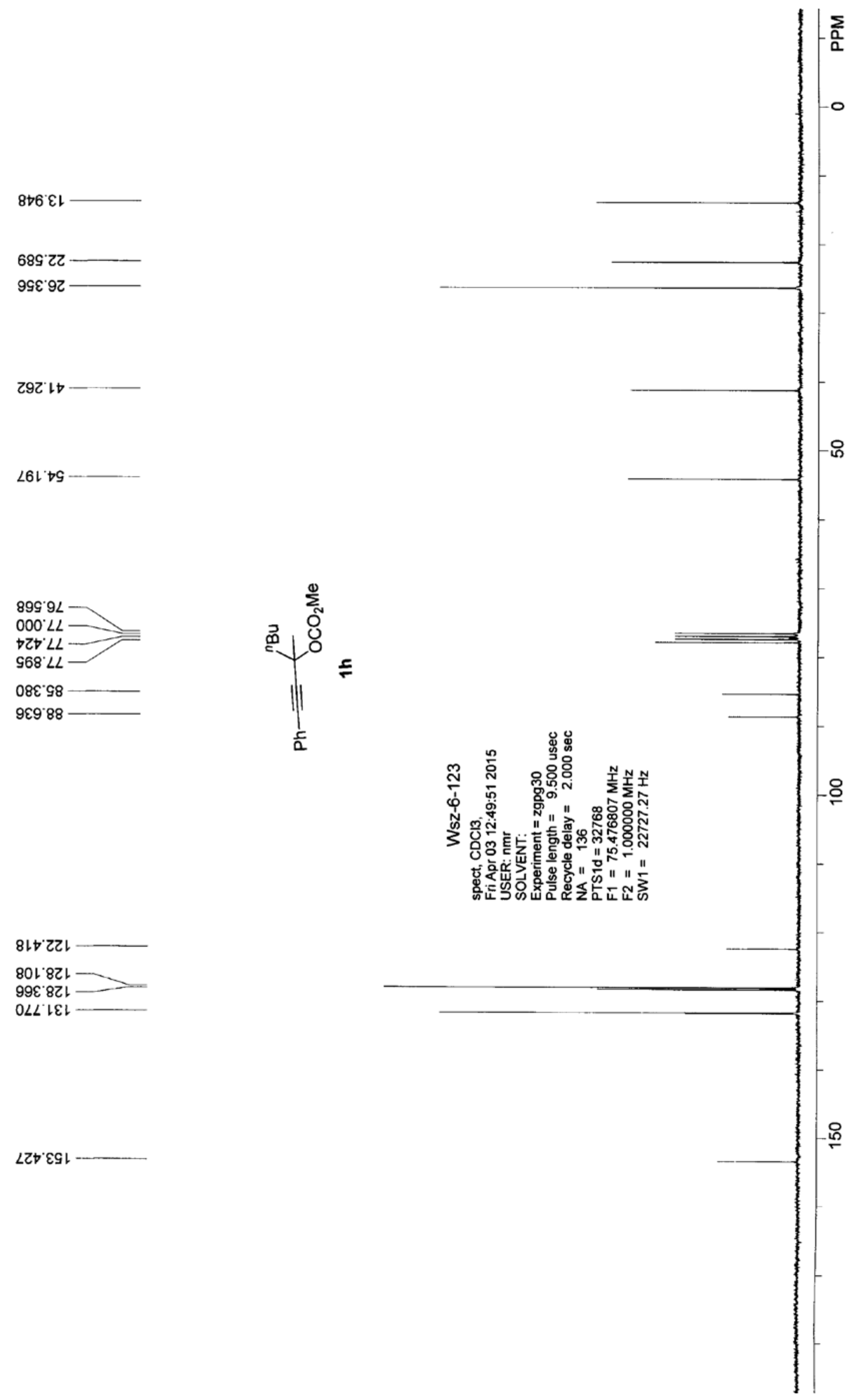

Supplementary Figure 6. ^{13}C NMR (75 MHz, $CDCl_3$) spectrum for 1h.

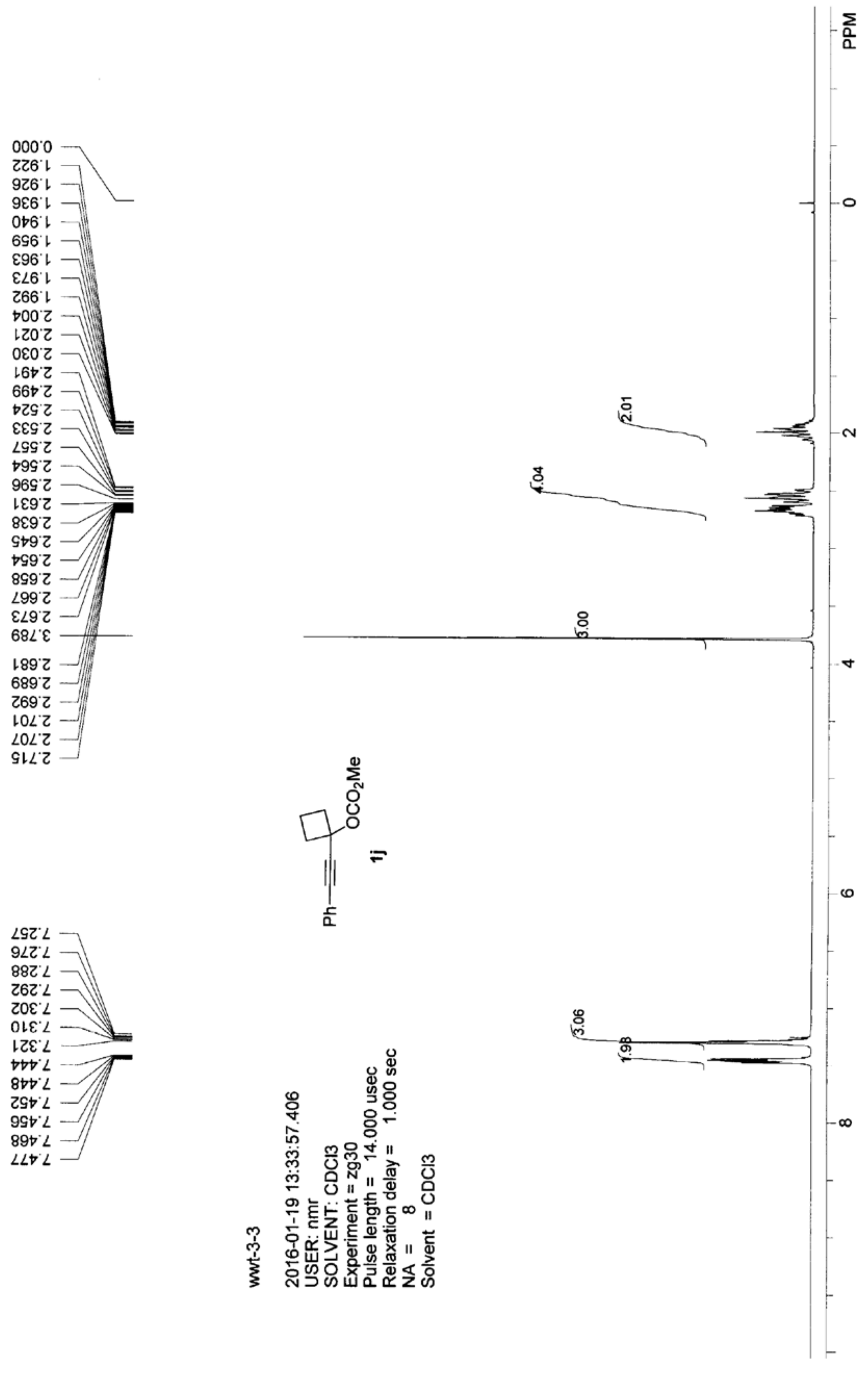

Supplementary Figure 7. ^{1}H NMR (300 MHz, $CDCl_3$) spectrum for 1j.

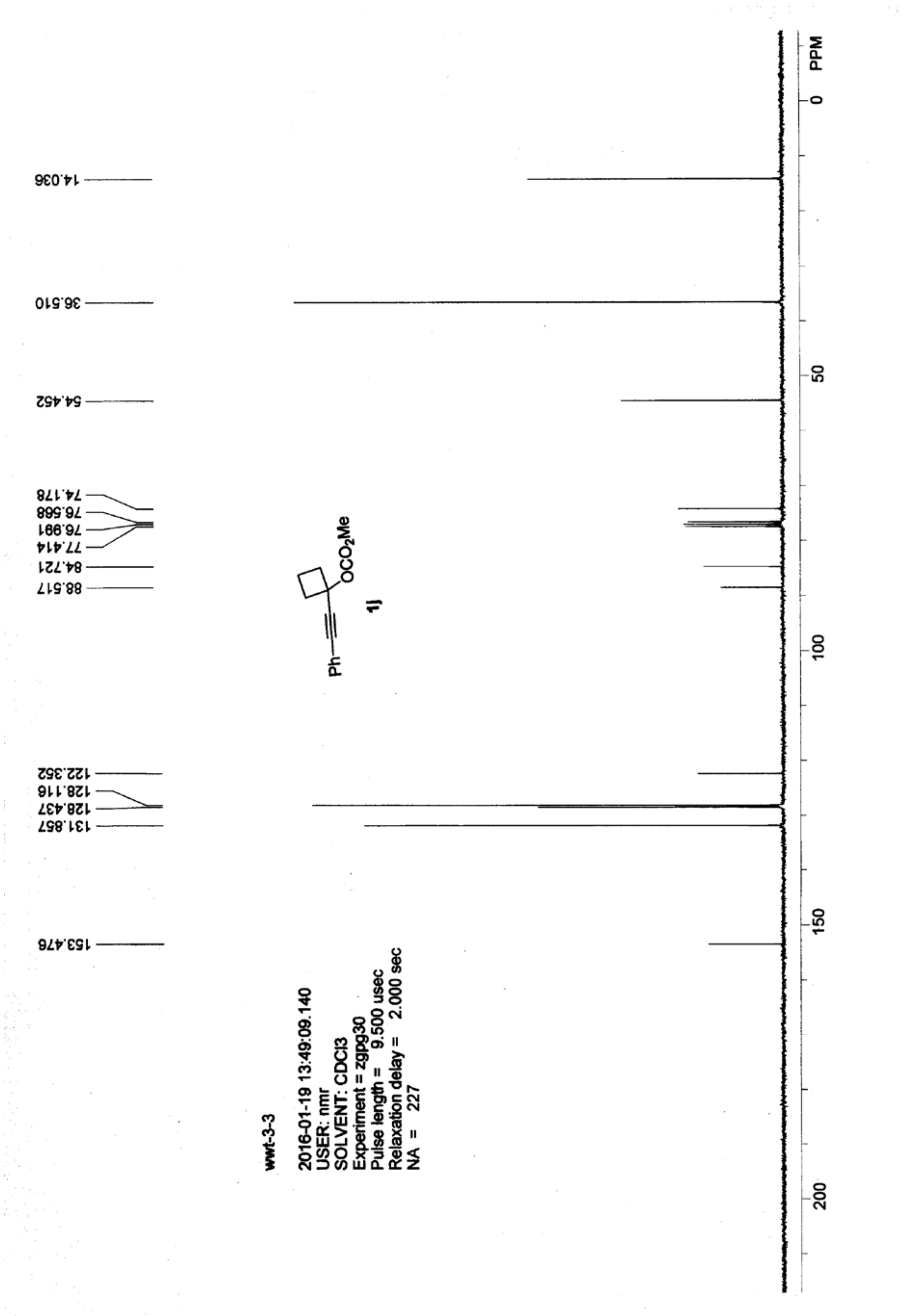

Supplementary Figure 8. **^{13}C NMR (75 MHz, $CDCl_3$) spectrum for 1j.**

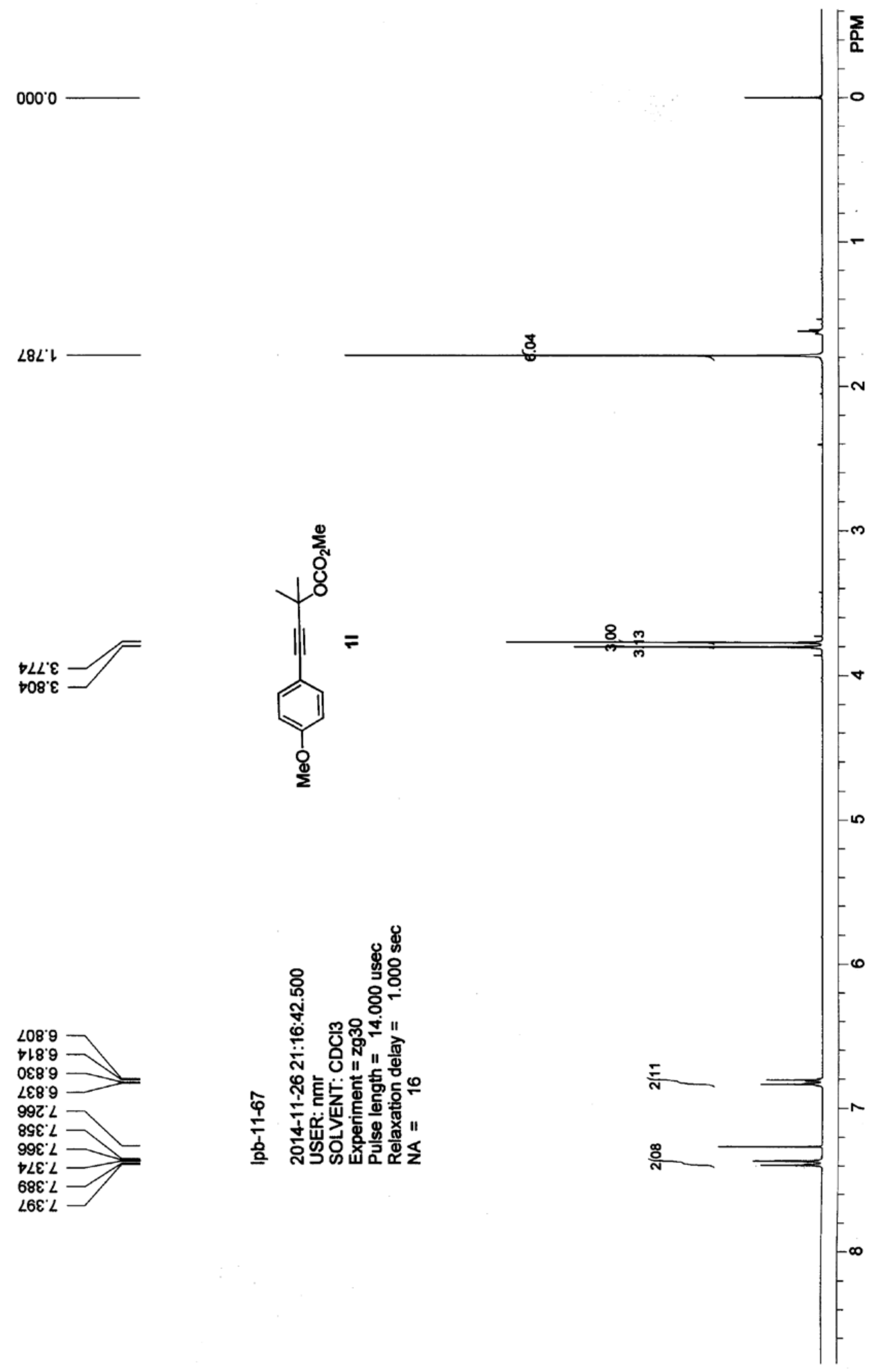

Supplementary Figure 9. **^{1}H NMR (300 MHz, $CDCl_3$) spectrum for 1l.**

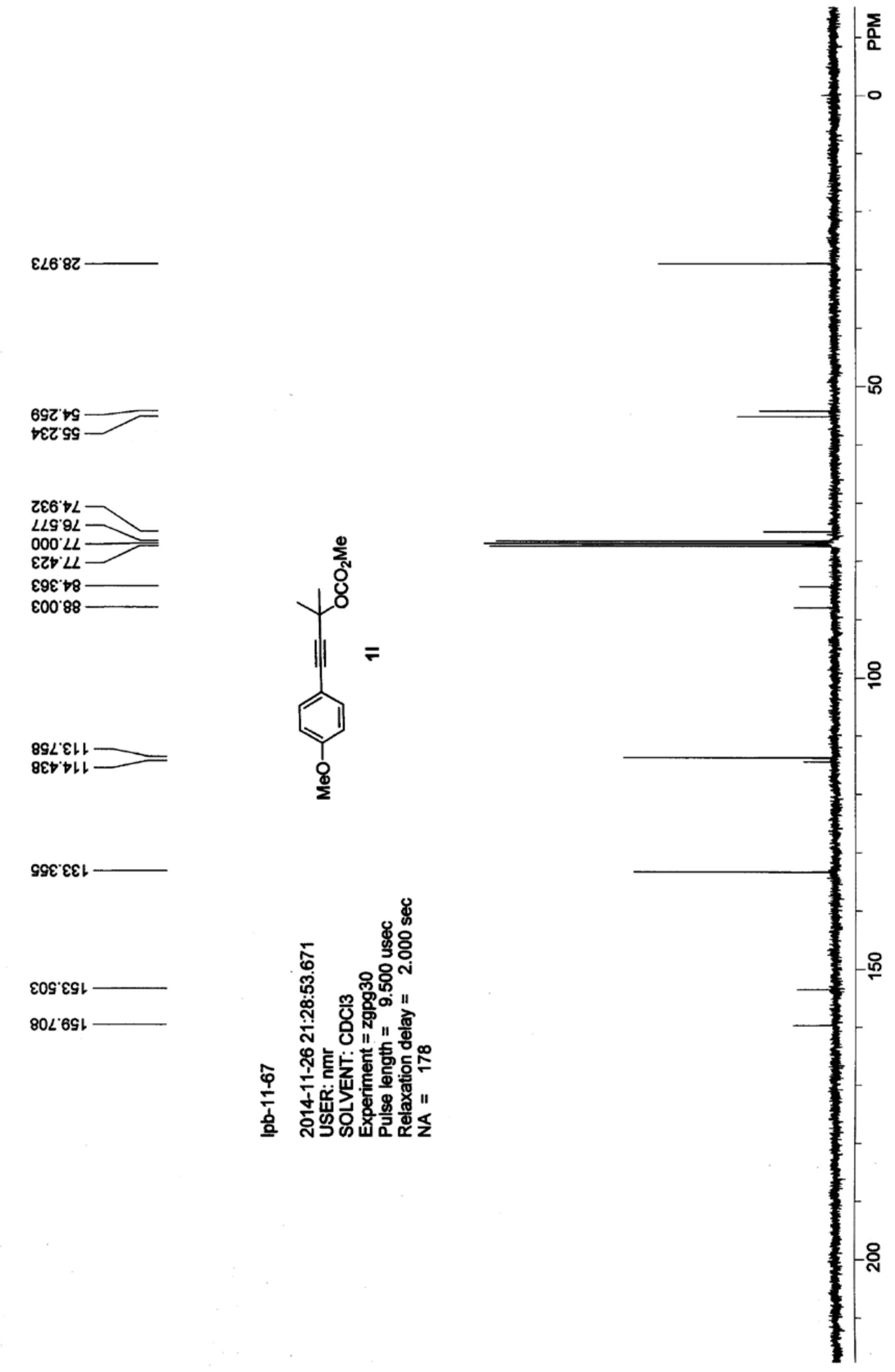

Supplementary Figure 10. ^{13}C NMR (75 MHz, $CDCl_3$) spectrum for 1l.

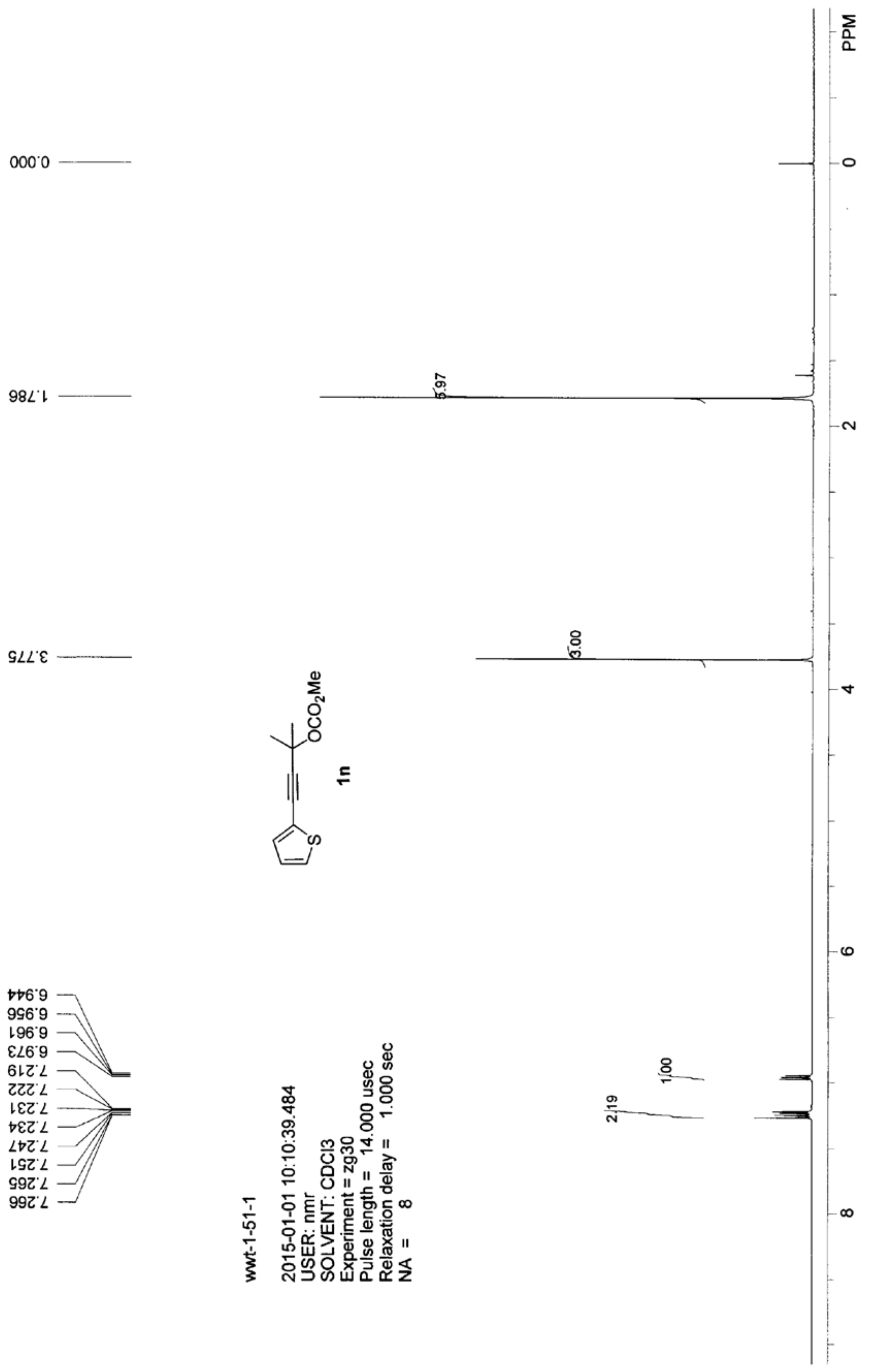

Supplementary Figure 11. **^{1}H NMR (300 MHz, $CDCl_3$) spectrum for 1n.**

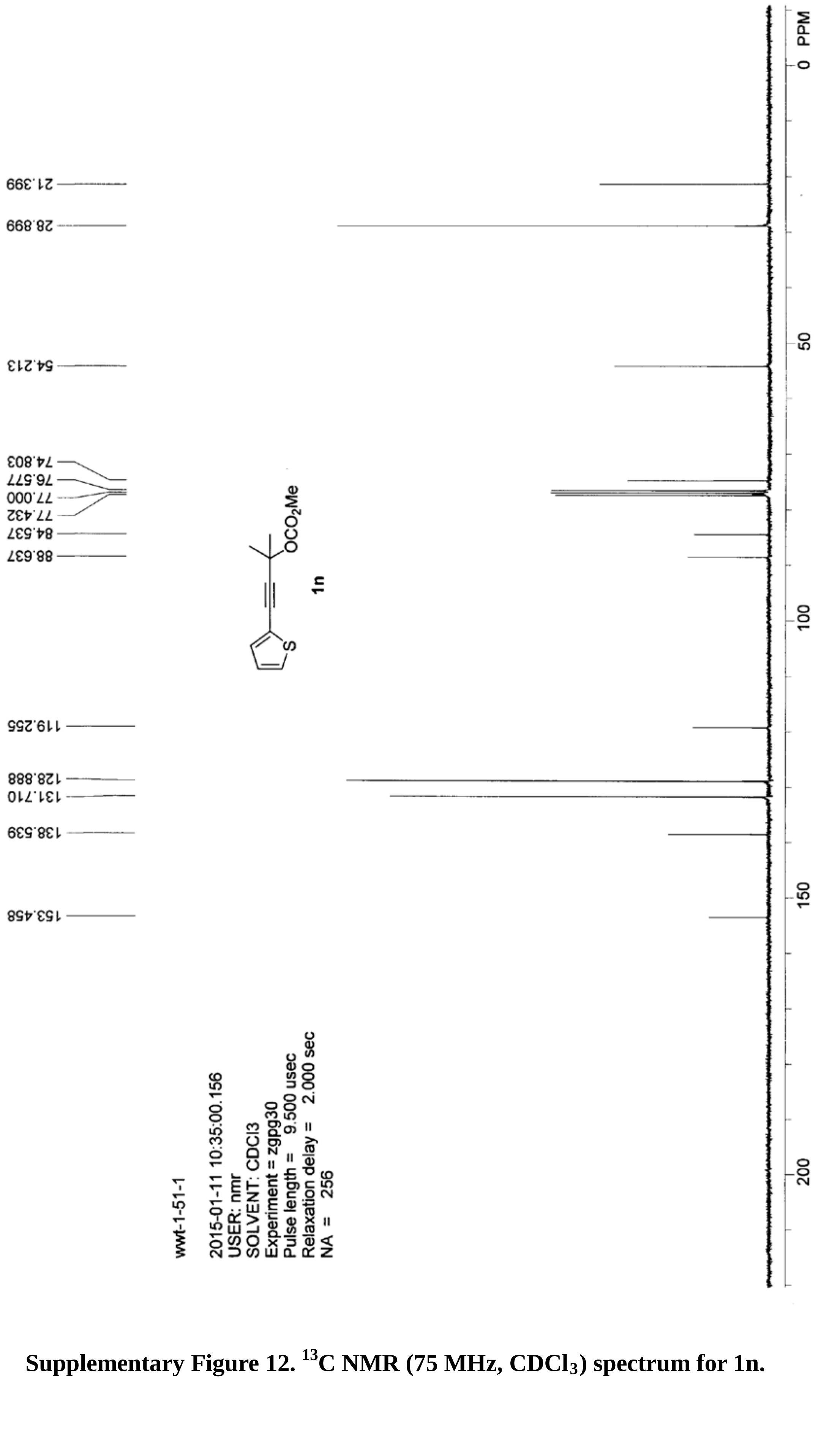

Supplementary Figure 12. ^{13}**C NMR (75 MHz,** $CDCl_3$**) spectrum for 1n.**

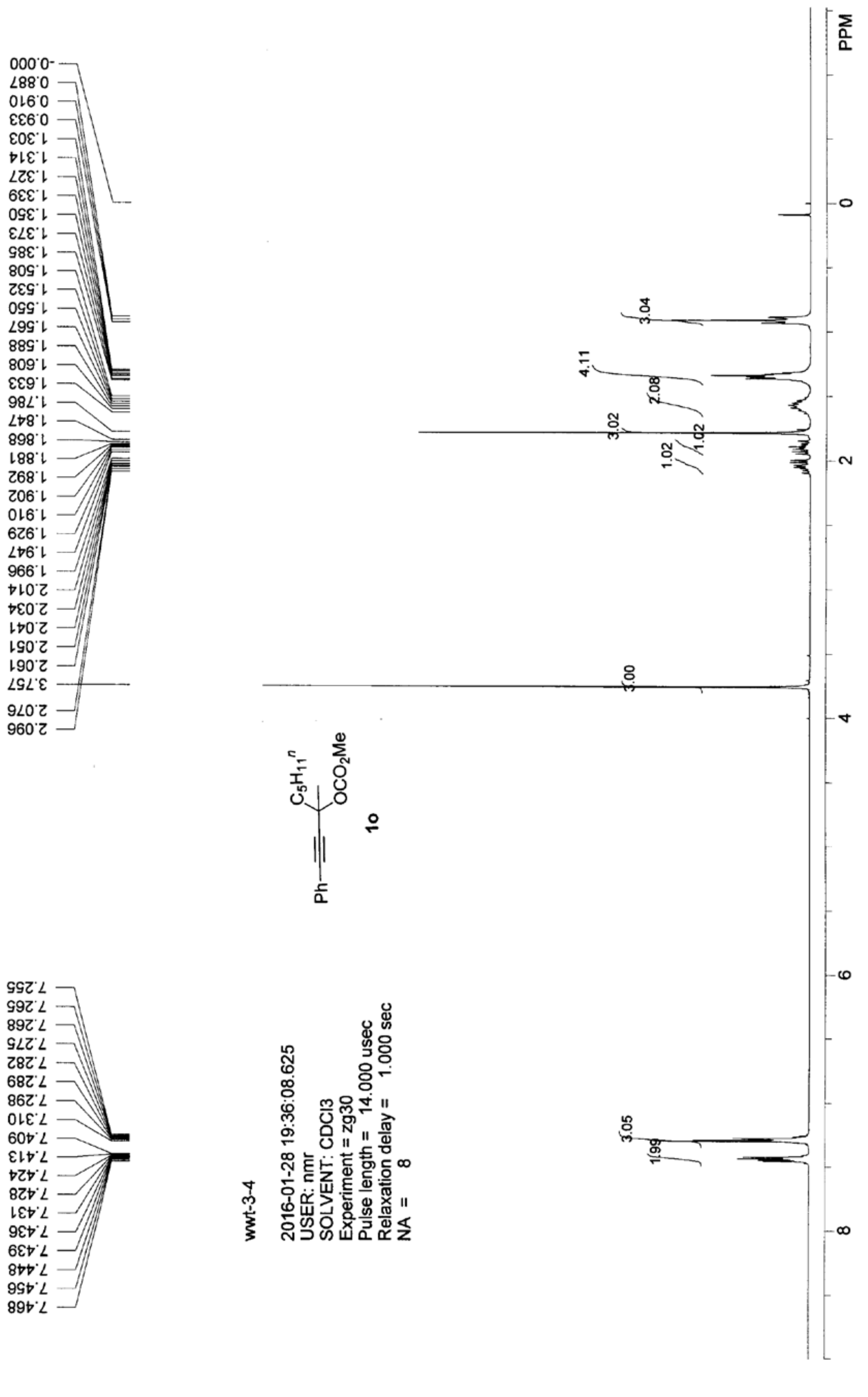

Supplementary Figure 13. ^{1}H **NMR (300 MHz,** $CDCl_3$**) spectrum for 1o.**

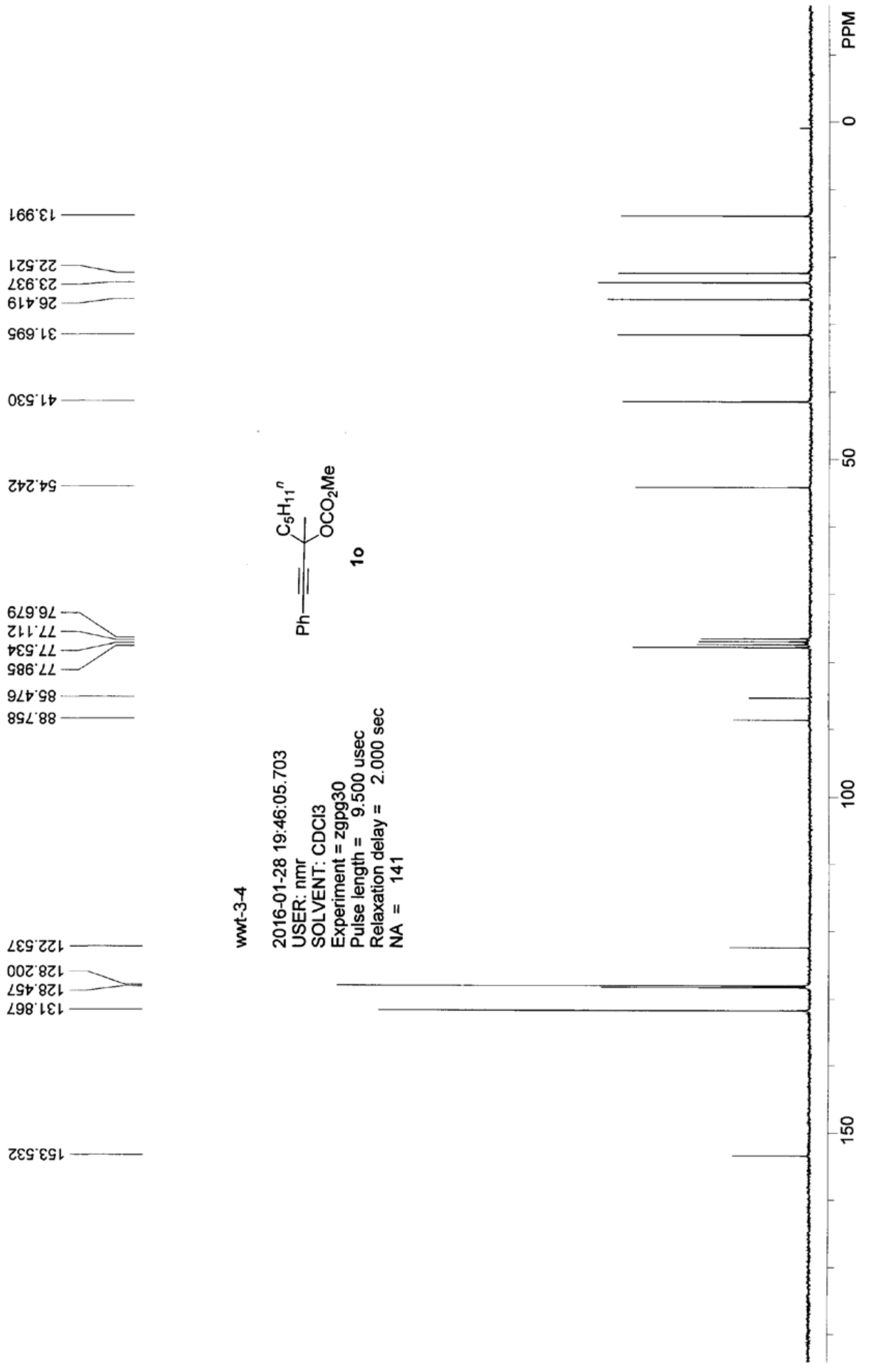

Supplementary Figure 14. ^{13}**C NMR (75 MHz, CDCl$_3$) spectrum for 1o.**

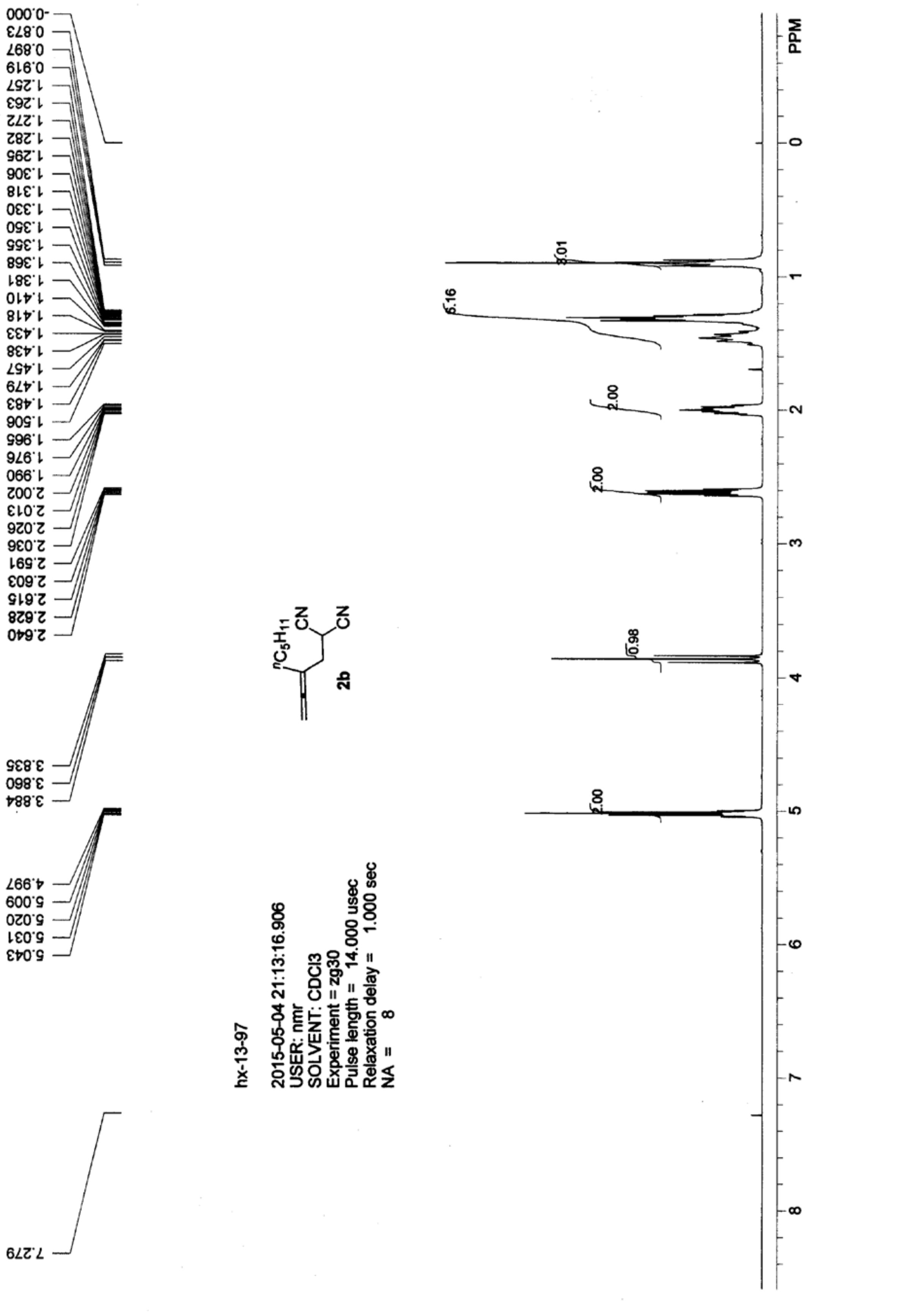

Supplementary Figure 15. ^{1}H NMR (300 MHz, $CDCl_3$) spectrum for 2b.

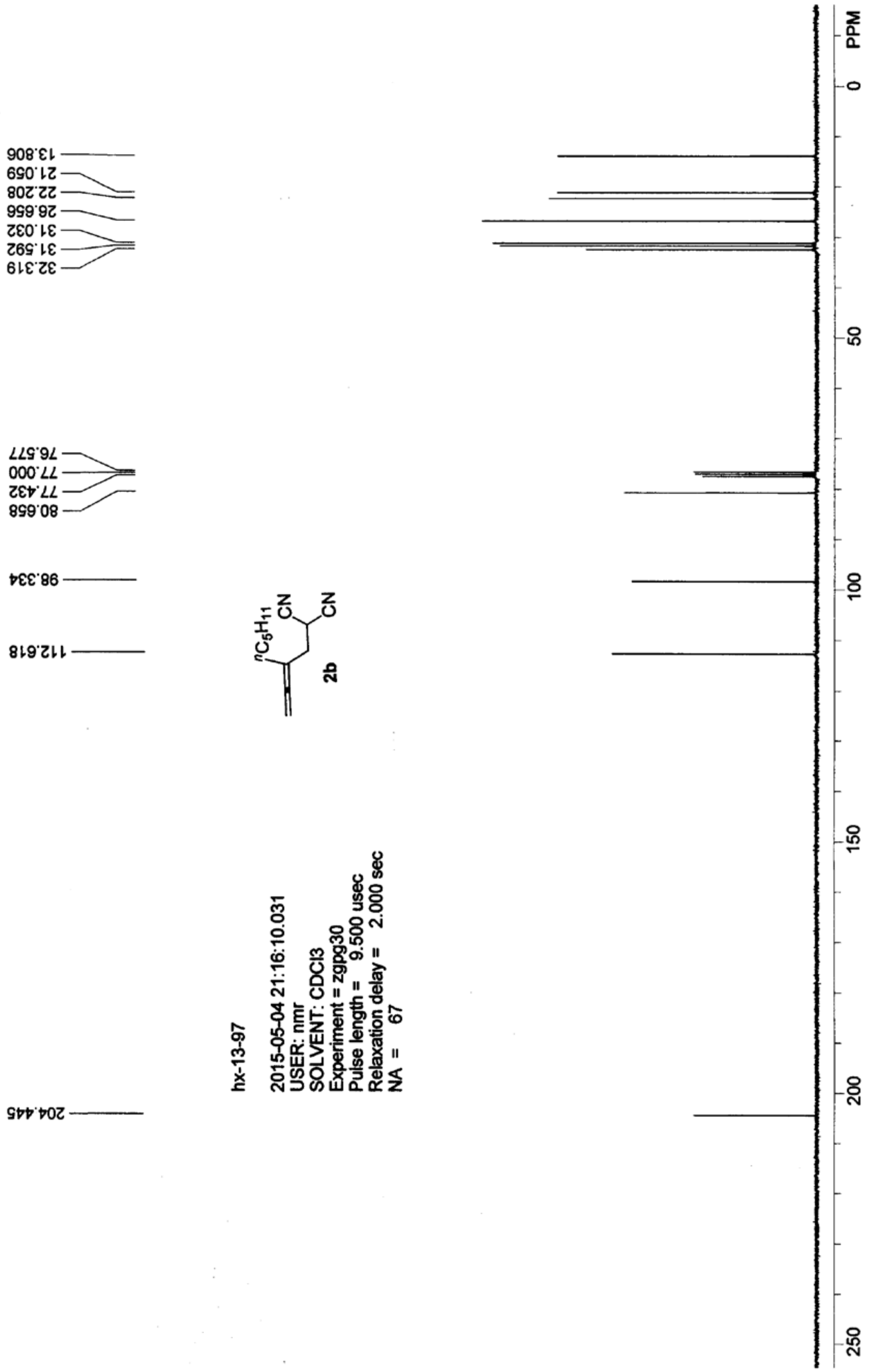

Supplementary Figure 16. ^{13}**C NMR (75 MHz, CDCl$_3$) spectrum for 2b.**

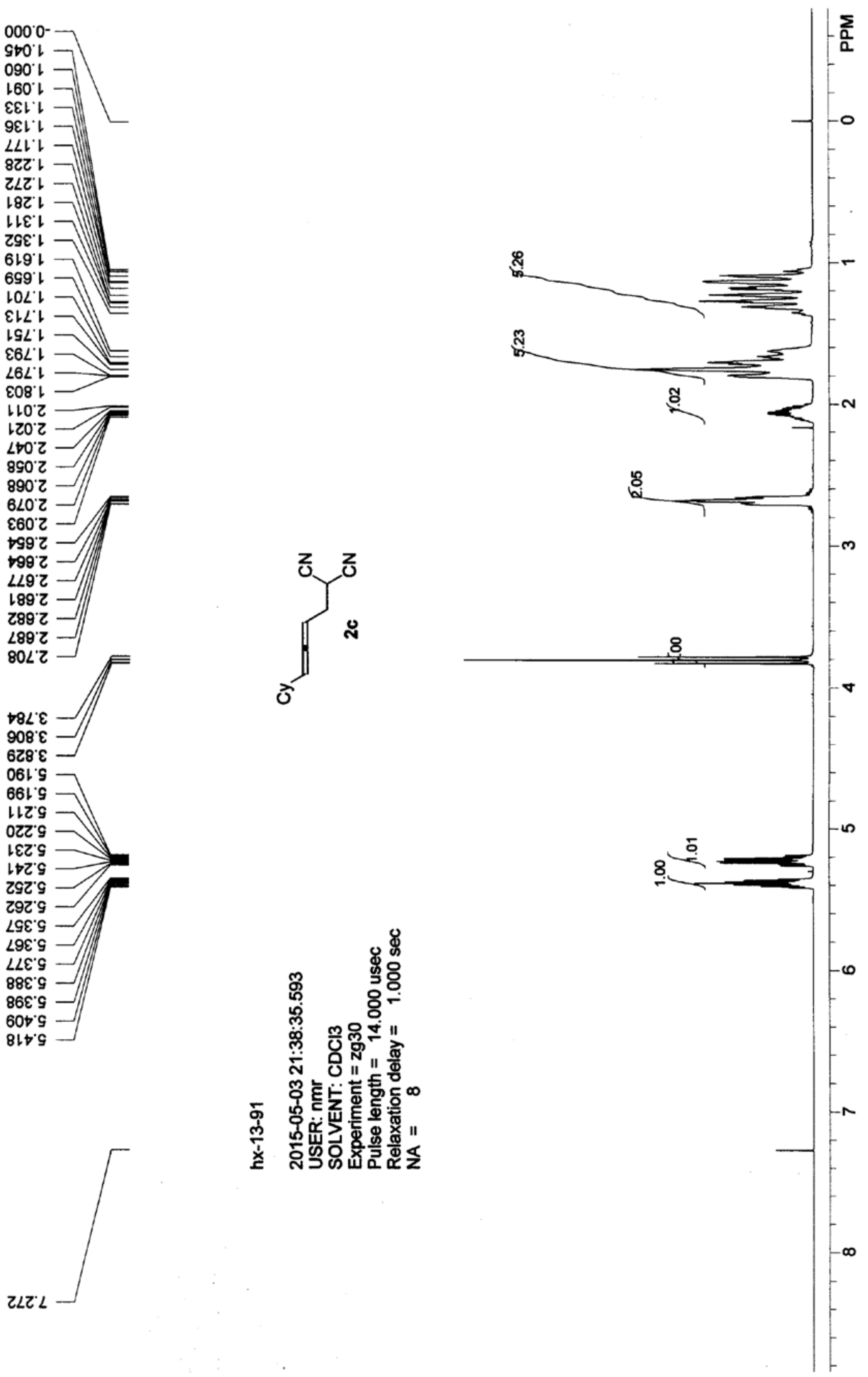

Supplementary Figure 17. ^{1}H NMR (300 MHz, $CDCl_3$) spectrum for 2c.

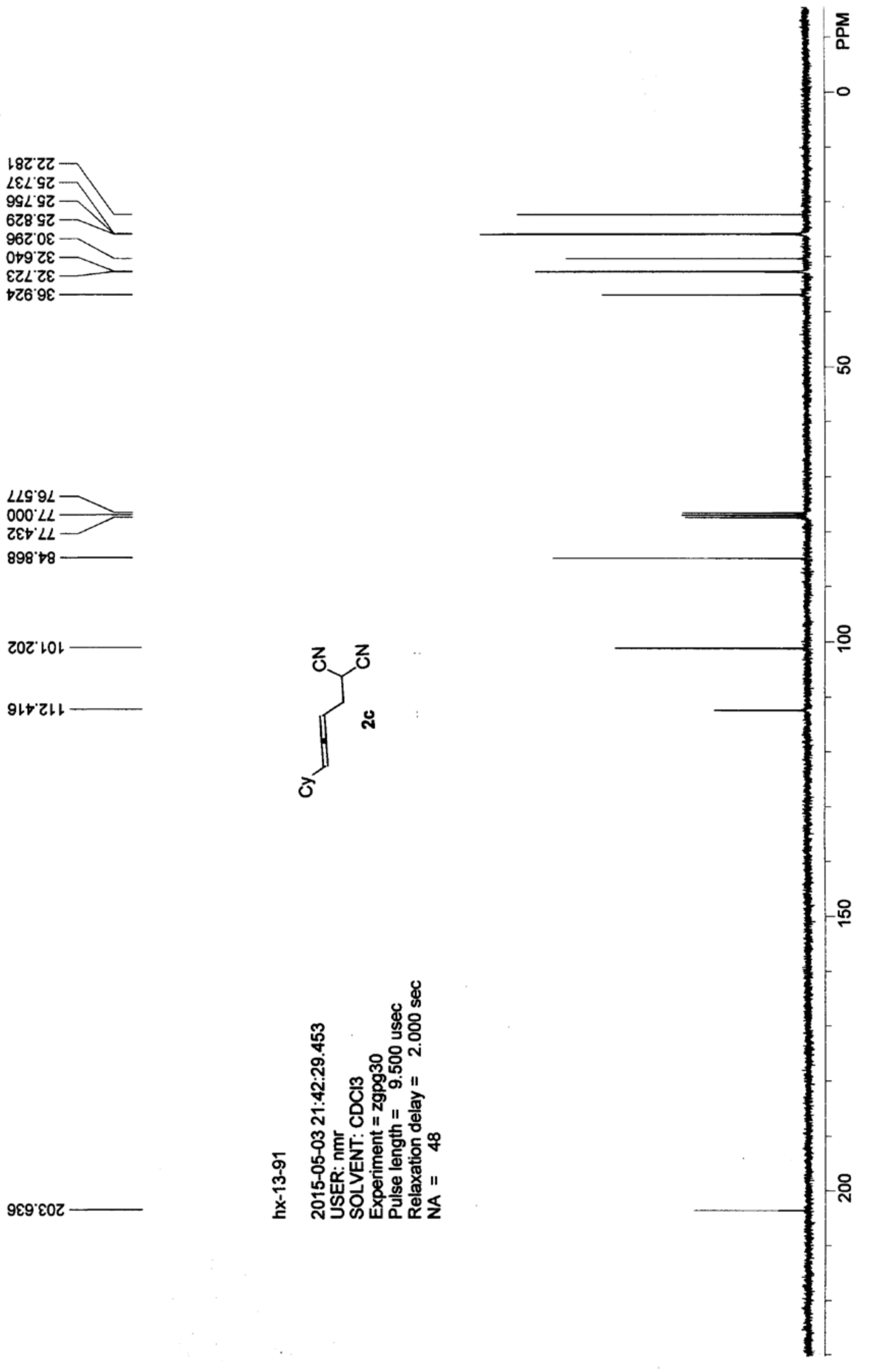

Supplementary Figure 18. ^{13}C NMR (75 MHz, $CDCl_3$) spectrum for 2c.

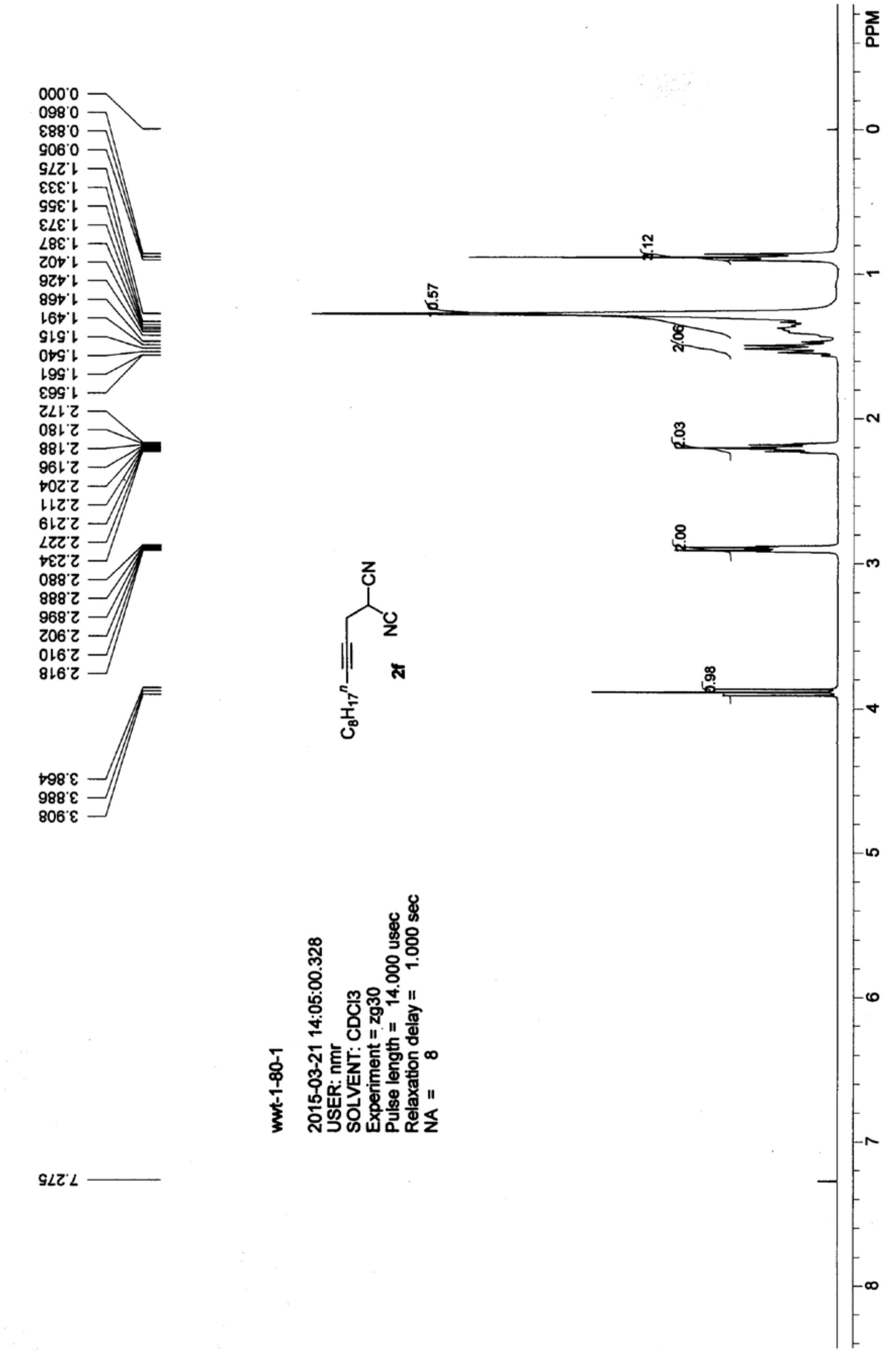

Supplementary Figure 19. ^{1}**H NMR (300 MHz,** $CDCl_3$**) spectrum for 2f.**

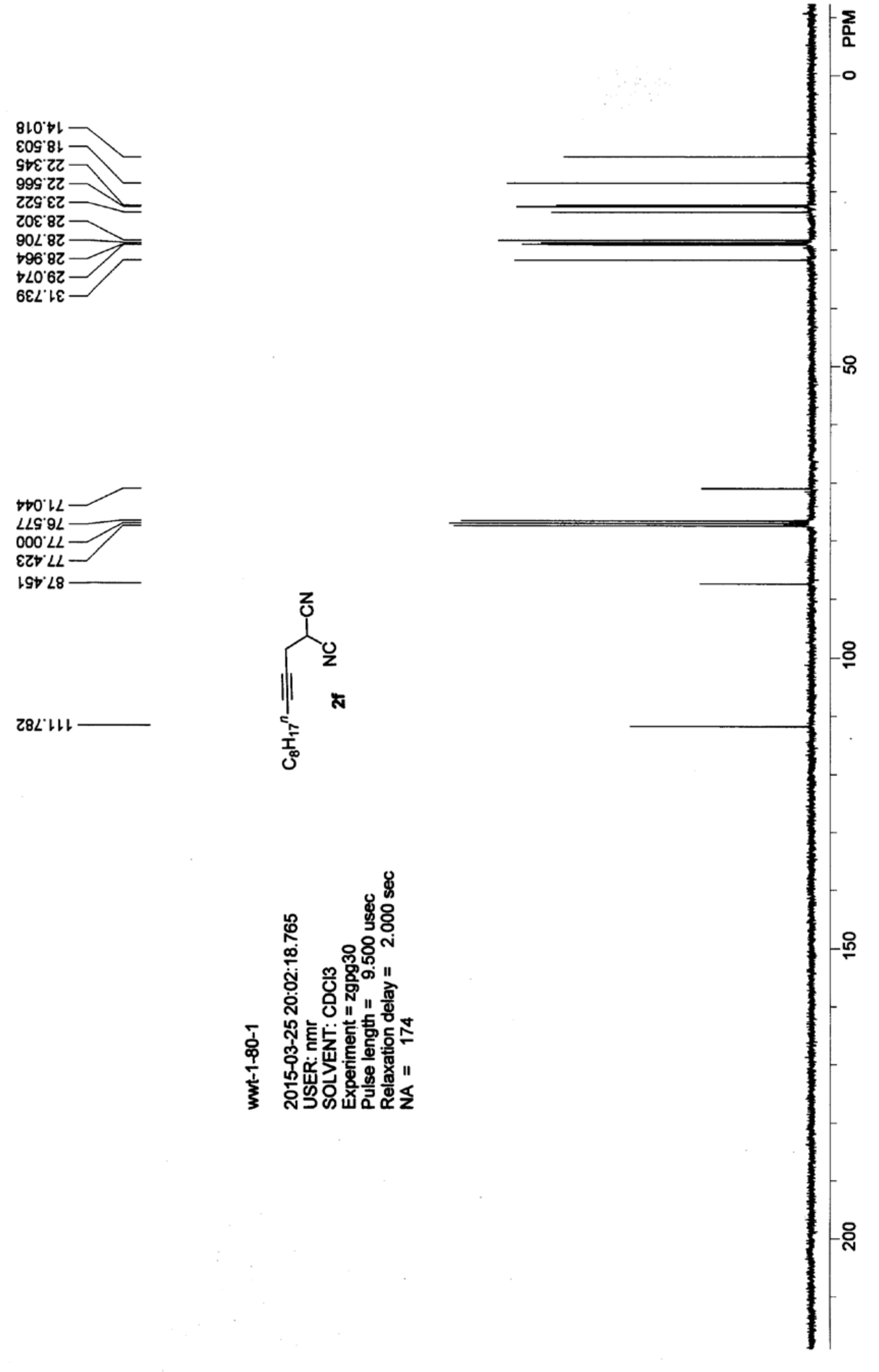

Supplementary Figure 20. **^{13}C NMR (75 MHz, $CDCl_3$) spectrum for 2f.**

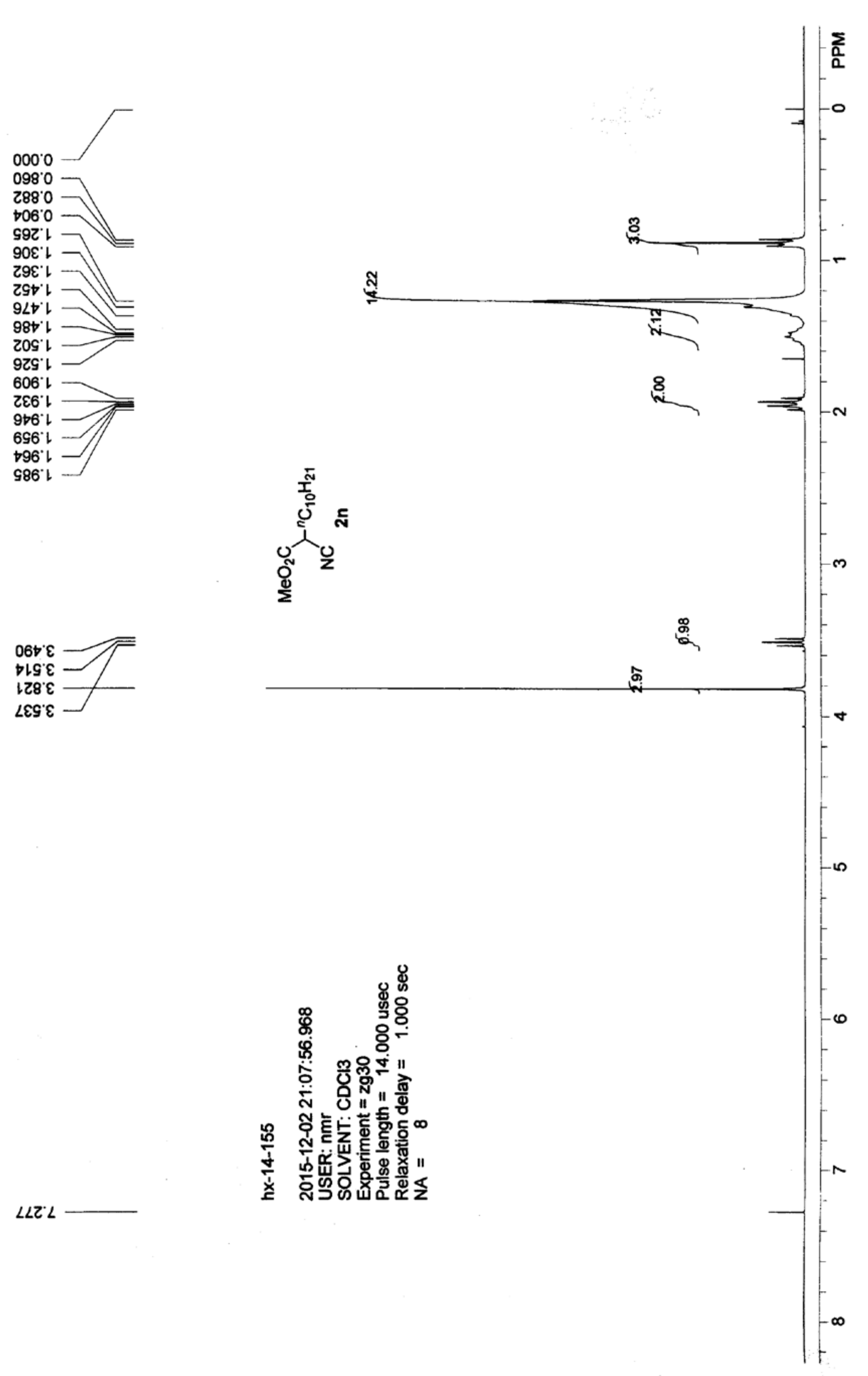

Supplementary Figure 21. ^{1}**H NMR (300 MHz,** $CDCl_3$**) spectrum for 2n.**

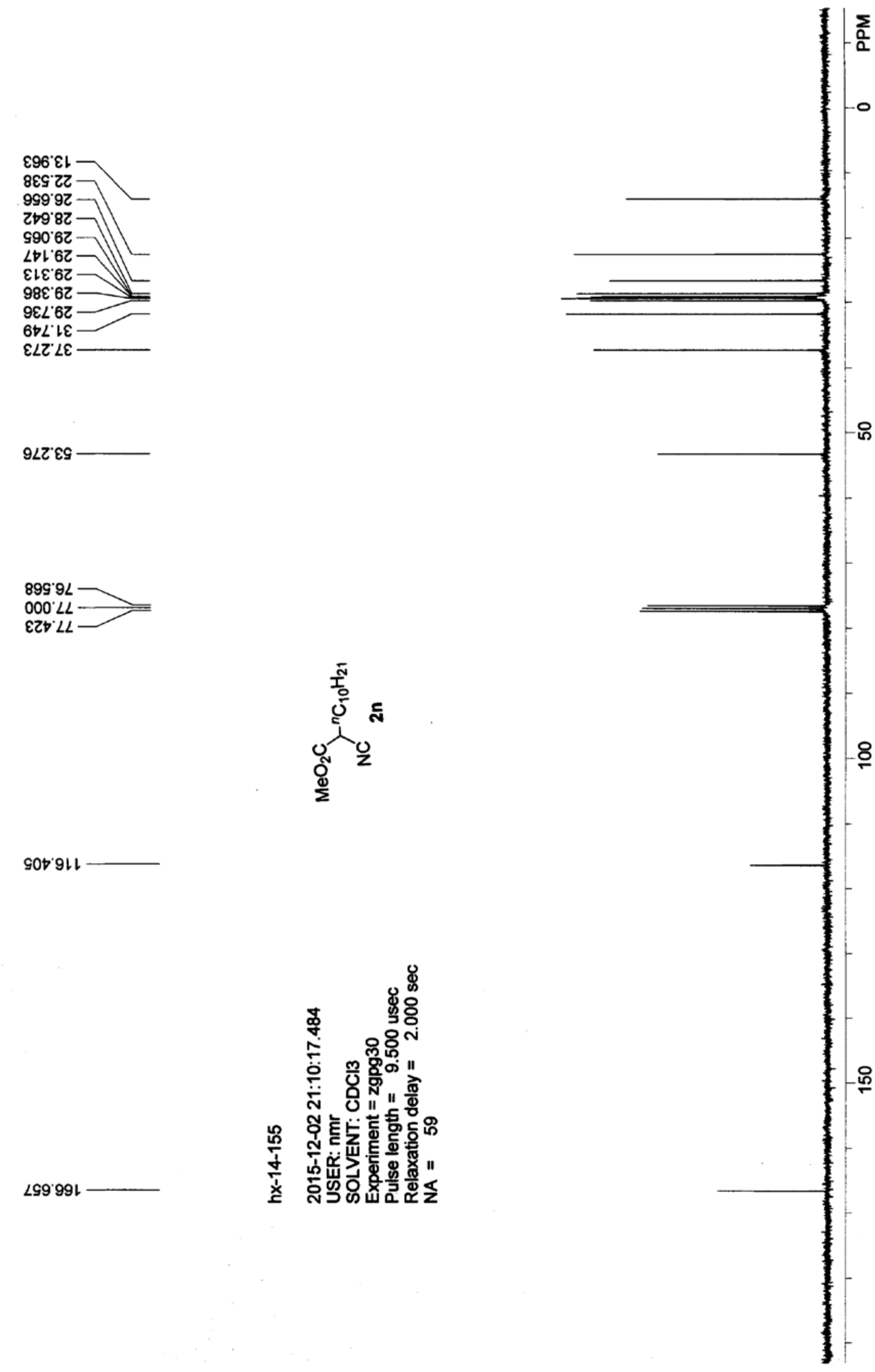

Supplementary Figure 22. ^{13}**C NMR (75 MHz,** $CDCl_3$**) spectrum for 2n.**

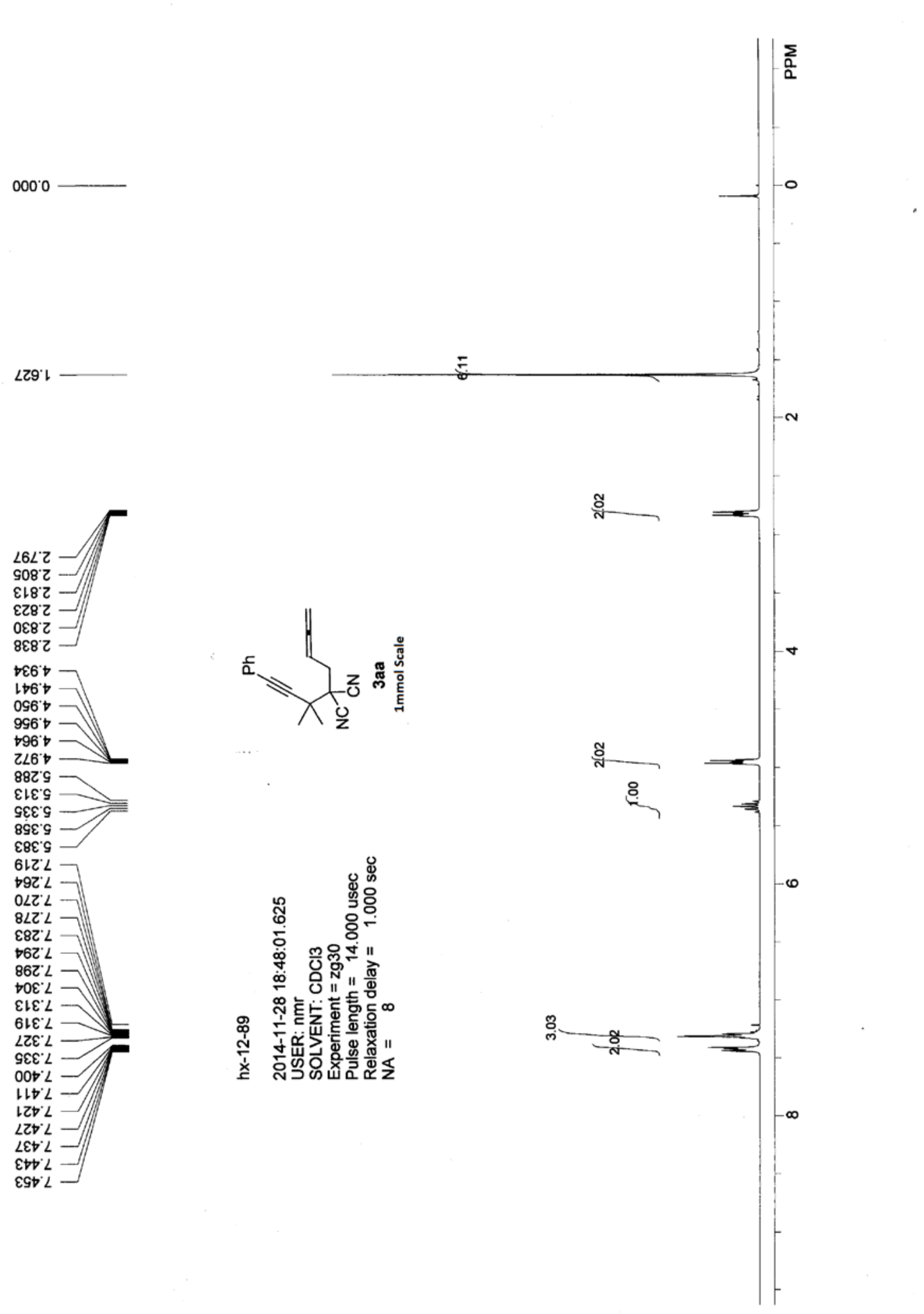

Supplementary Figure 23. ^{1}H NMR (300 MHz, $CDCl_3$) spectrum for 3aa.

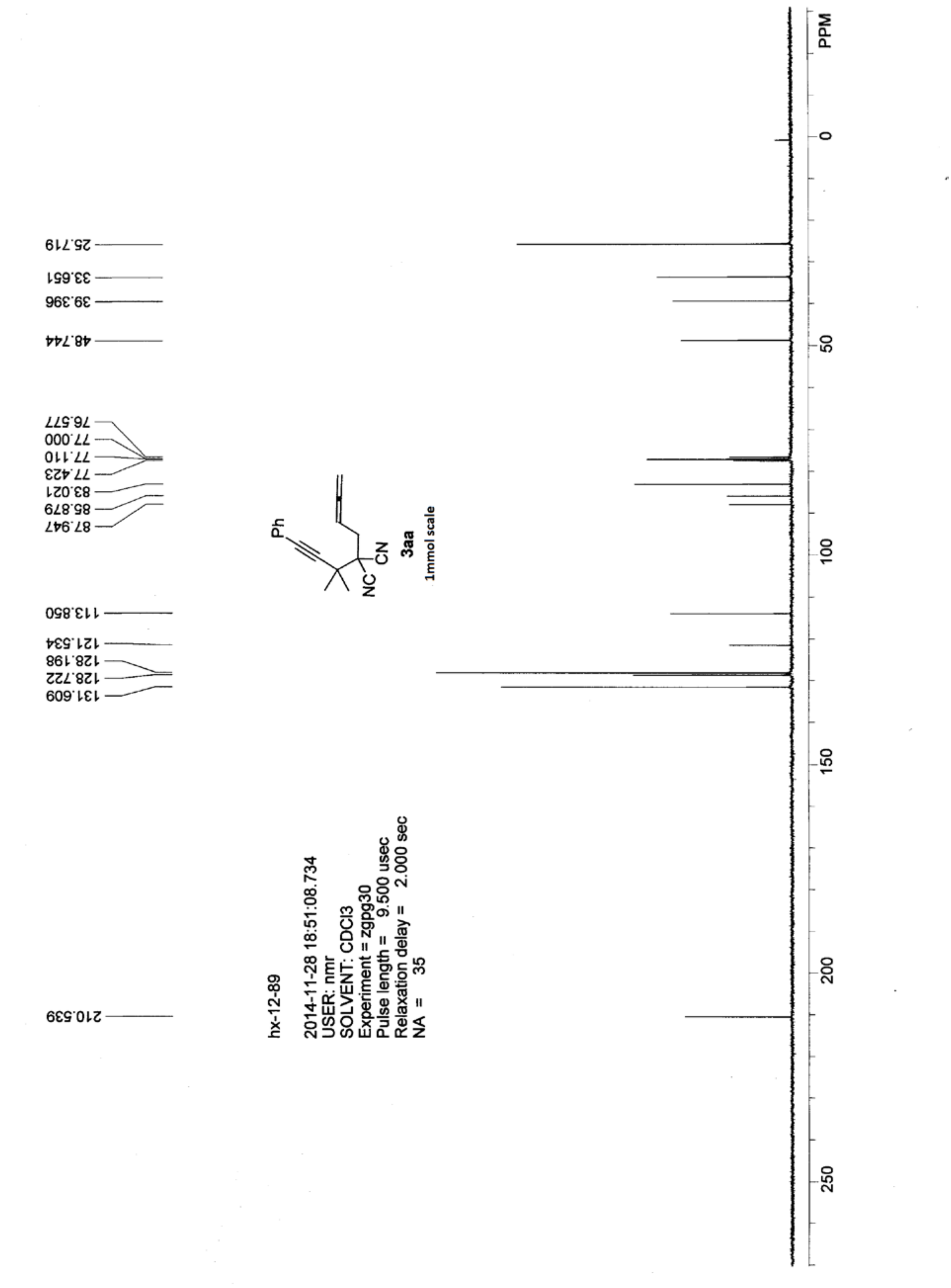

Supplementary Figure 24. ^{13}**C NMR (75 MHz, CDCl$_3$) spectrum for 3aa.**

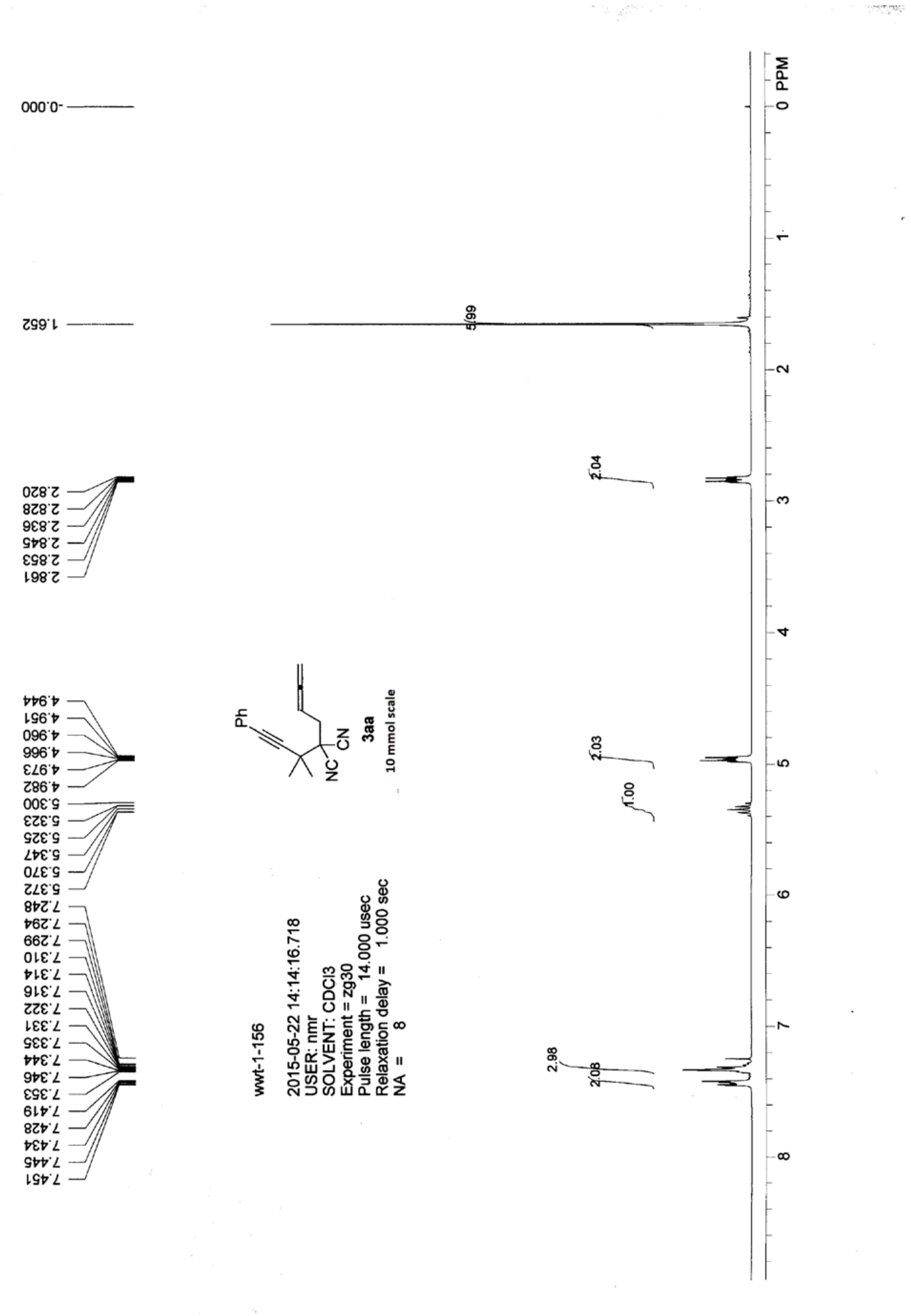

Supplementary Figure 25. ^{1}H NMR (300 MHz, $CDCl_3$) spectrum for 3aa.

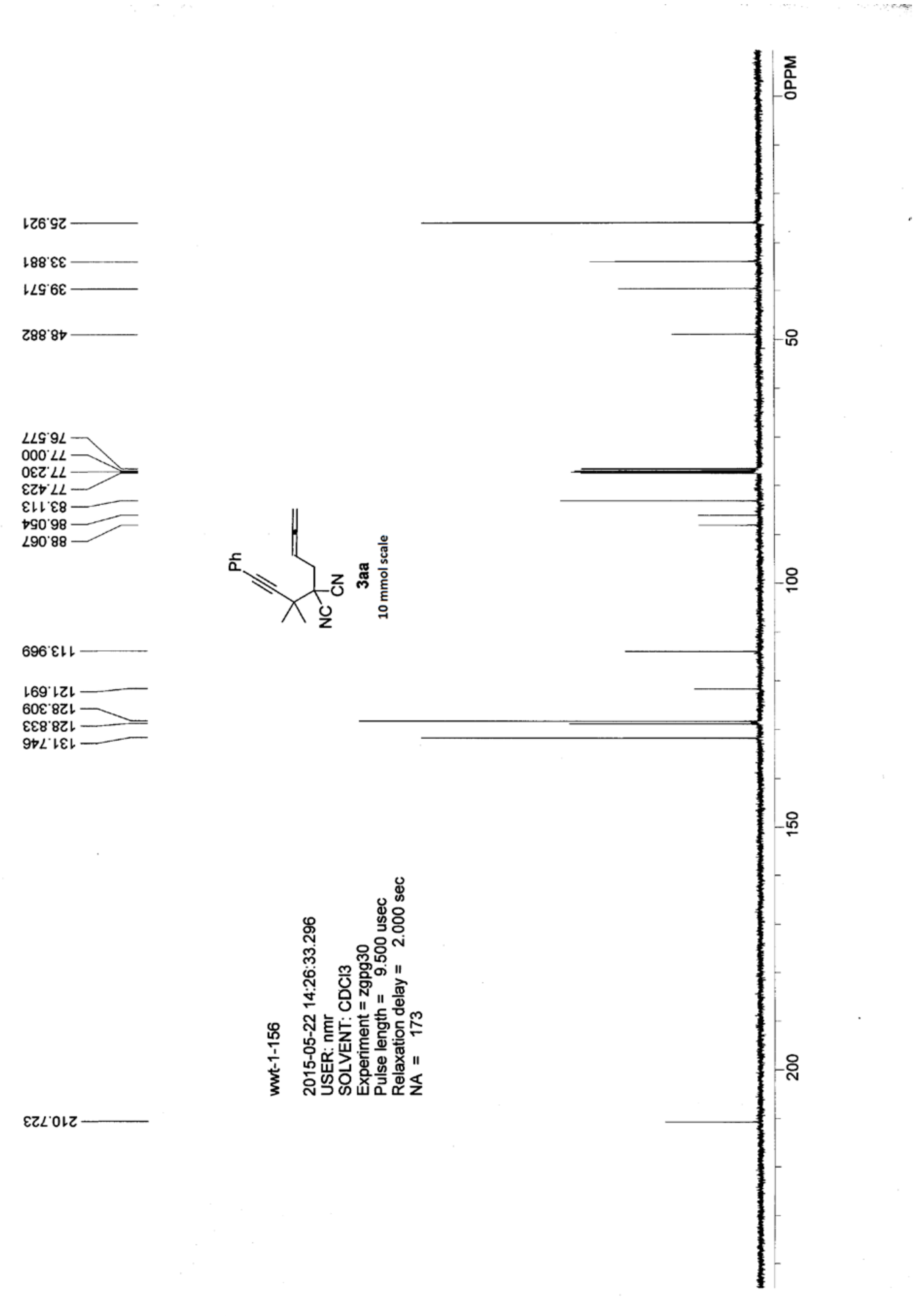

Supplementary Figure 26. ^{13}C NMR (75 MHz, $CDCl_3$) spectrum for 3aa.

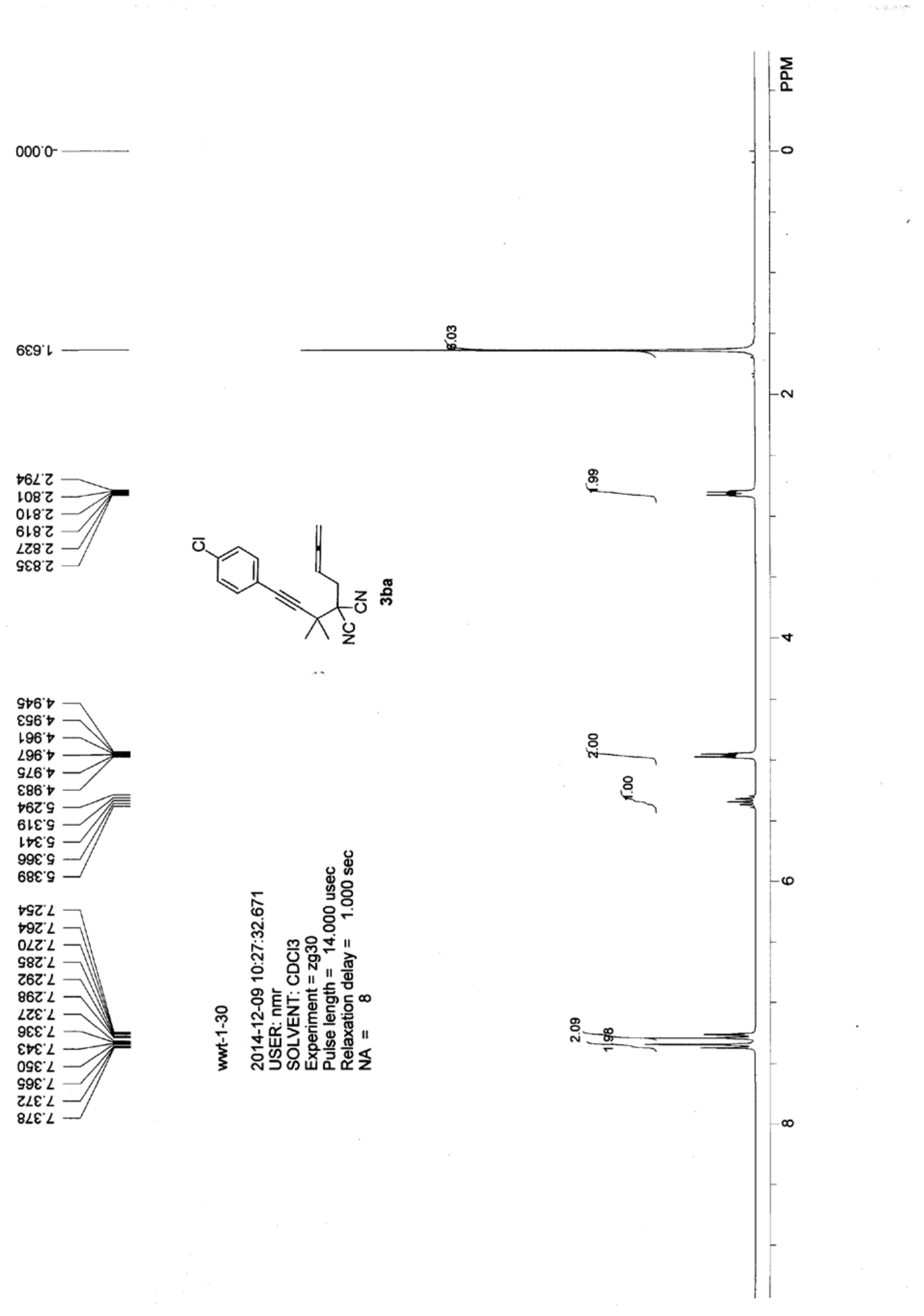

Supplementary Figure 27. **^{1}H NMR (300 MHz, $CDCl_3$) spectrum for 3ba.**

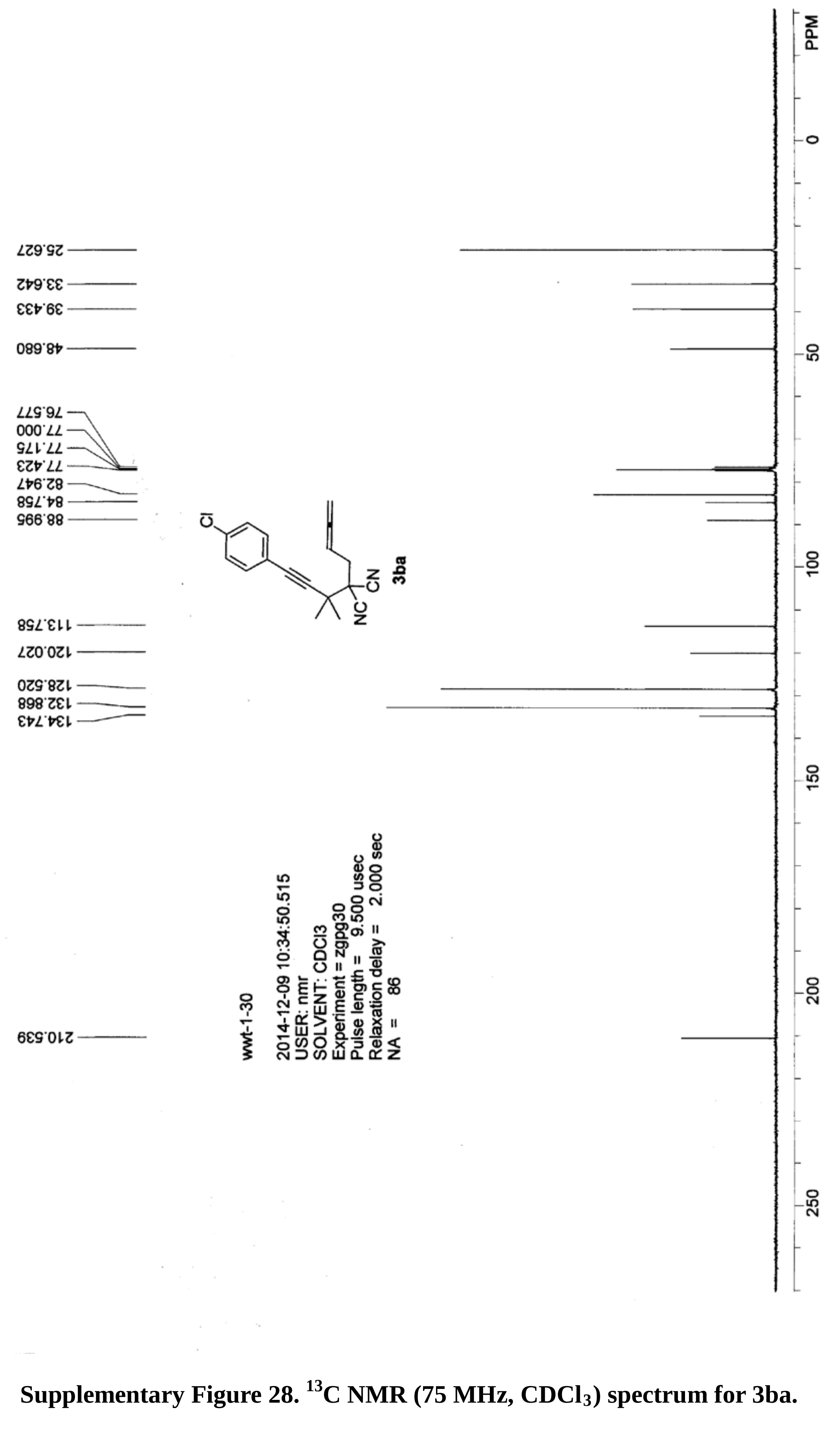

Supplementary Figure 28. ^{13}C NMR (75 MHz, $CDCl_3$) spectrum for 3ba.

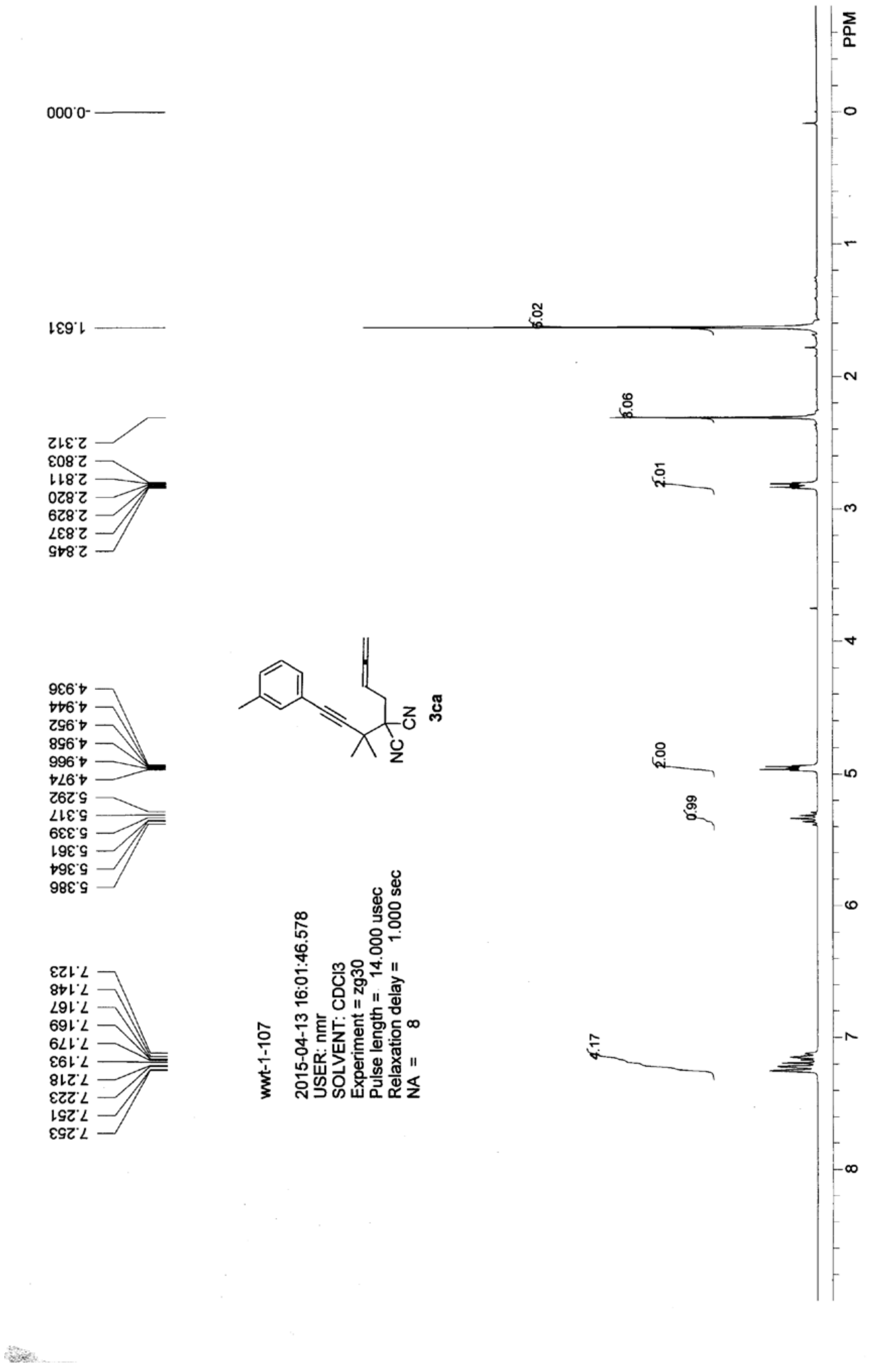

Supplementary Figure 29. ^{1}H NMR (300 MHz, $CDCl_3$) spectrum for 3ca.

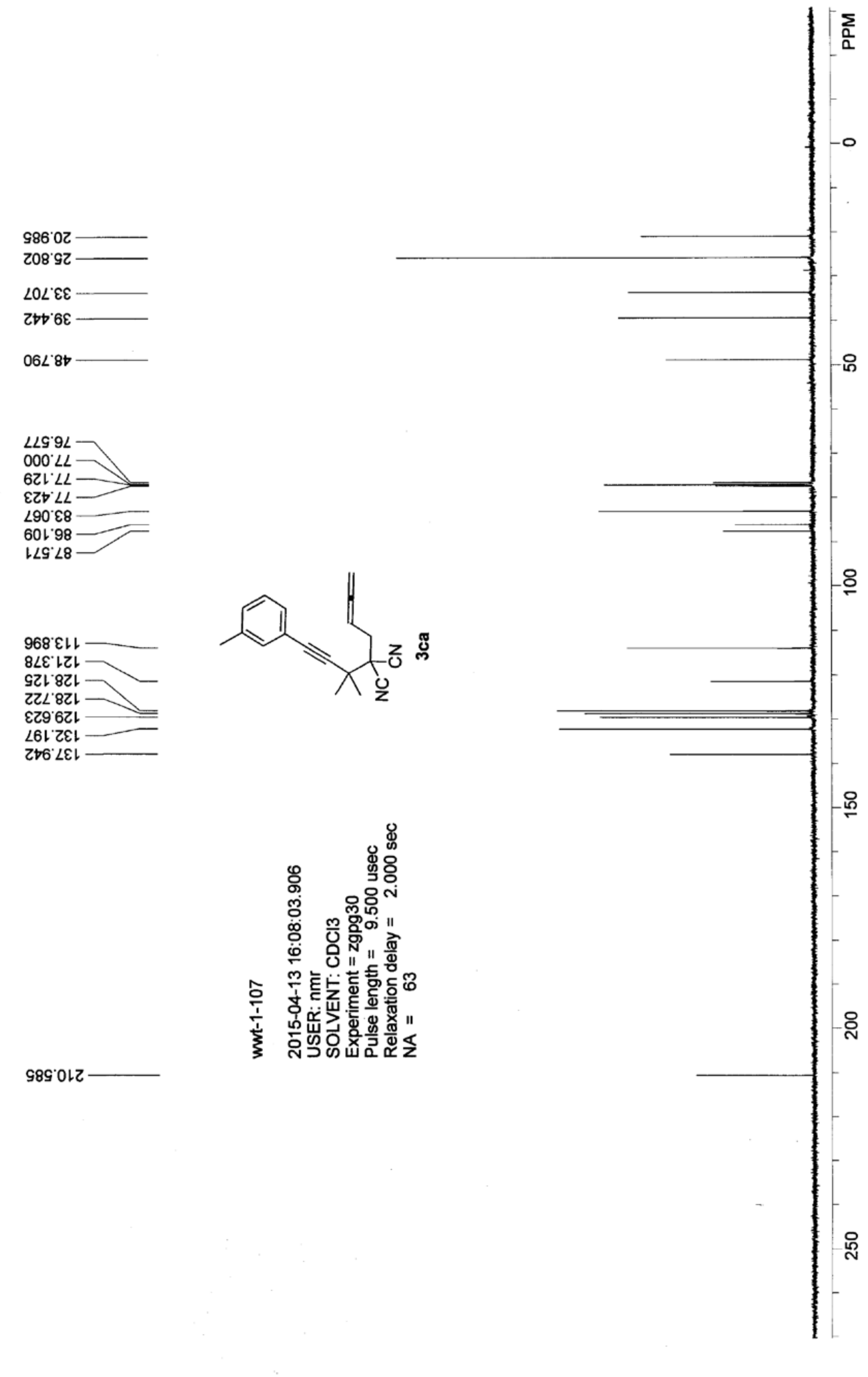

Supplementary Figure 30. **^{13}C NMR (75 MHz, $CDCl_3$) spectrum for 3ca.**

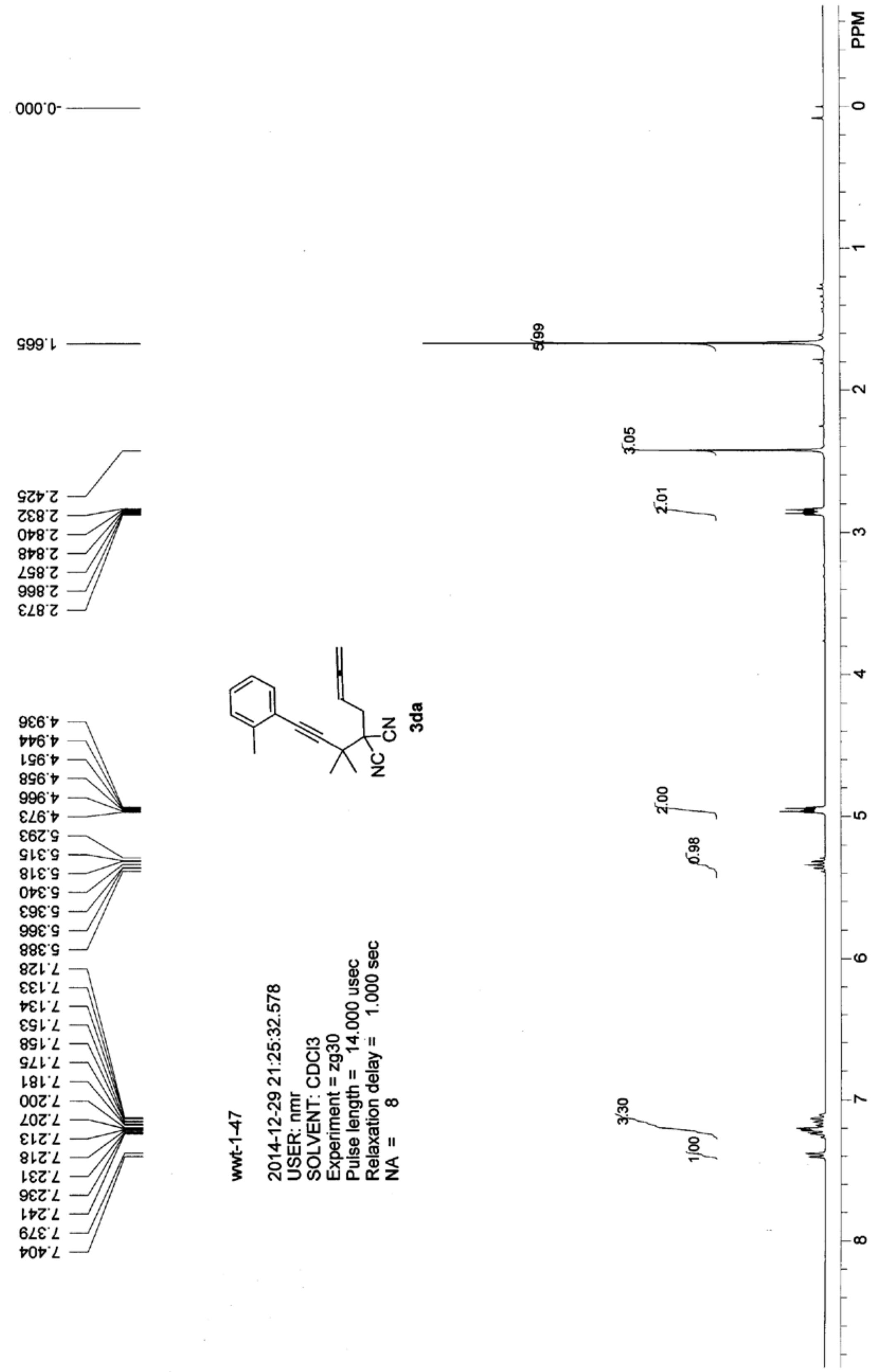

Supplementary Figure 31. **^{1}H NMR (300 MHz, $CDCl_3$) spectrum for 3da.**

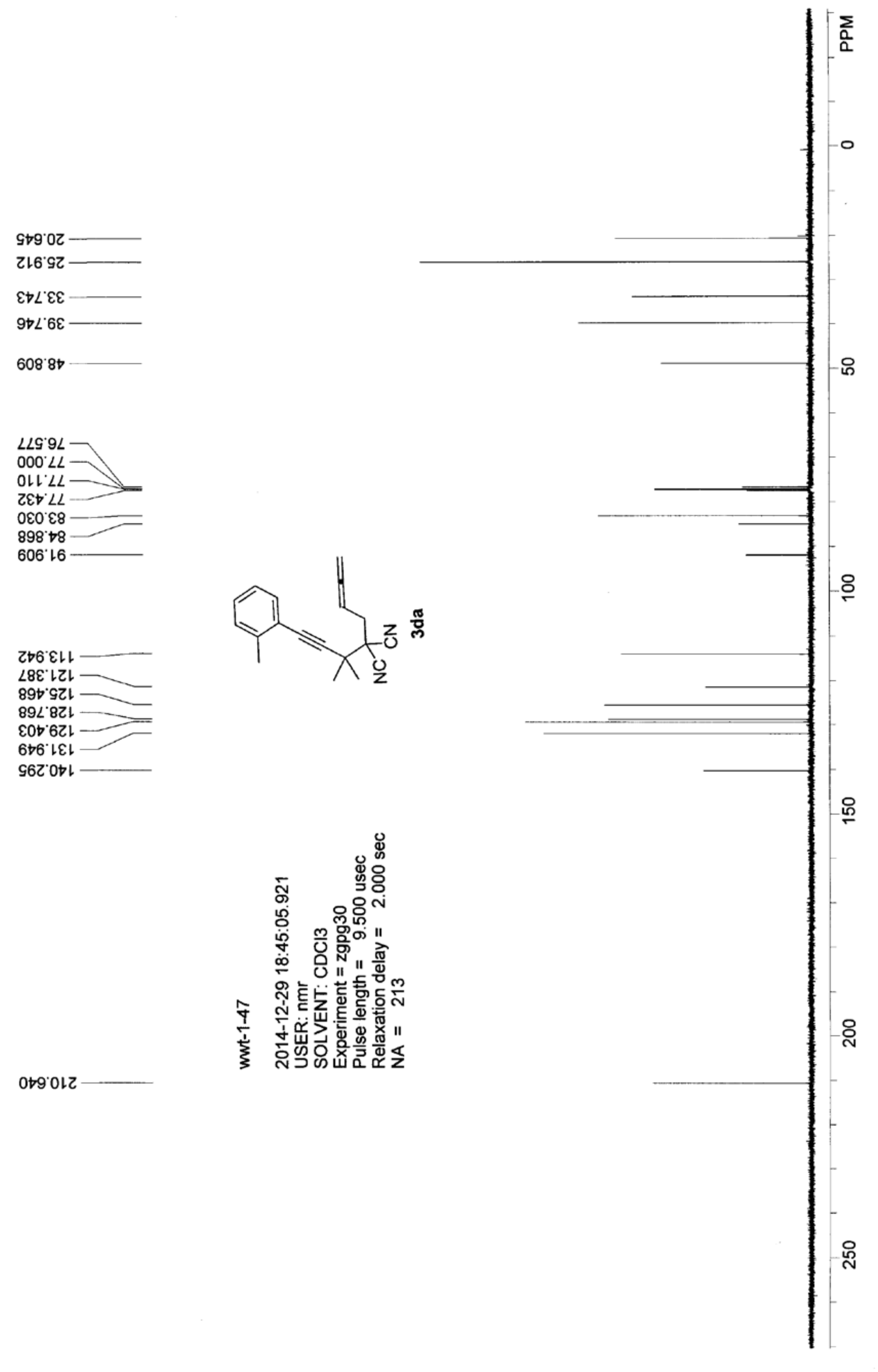

Supplementary Figure 32. ^{13}**C NMR (75 MHz,** $CDCl_3$**) spectrum for 3da.**

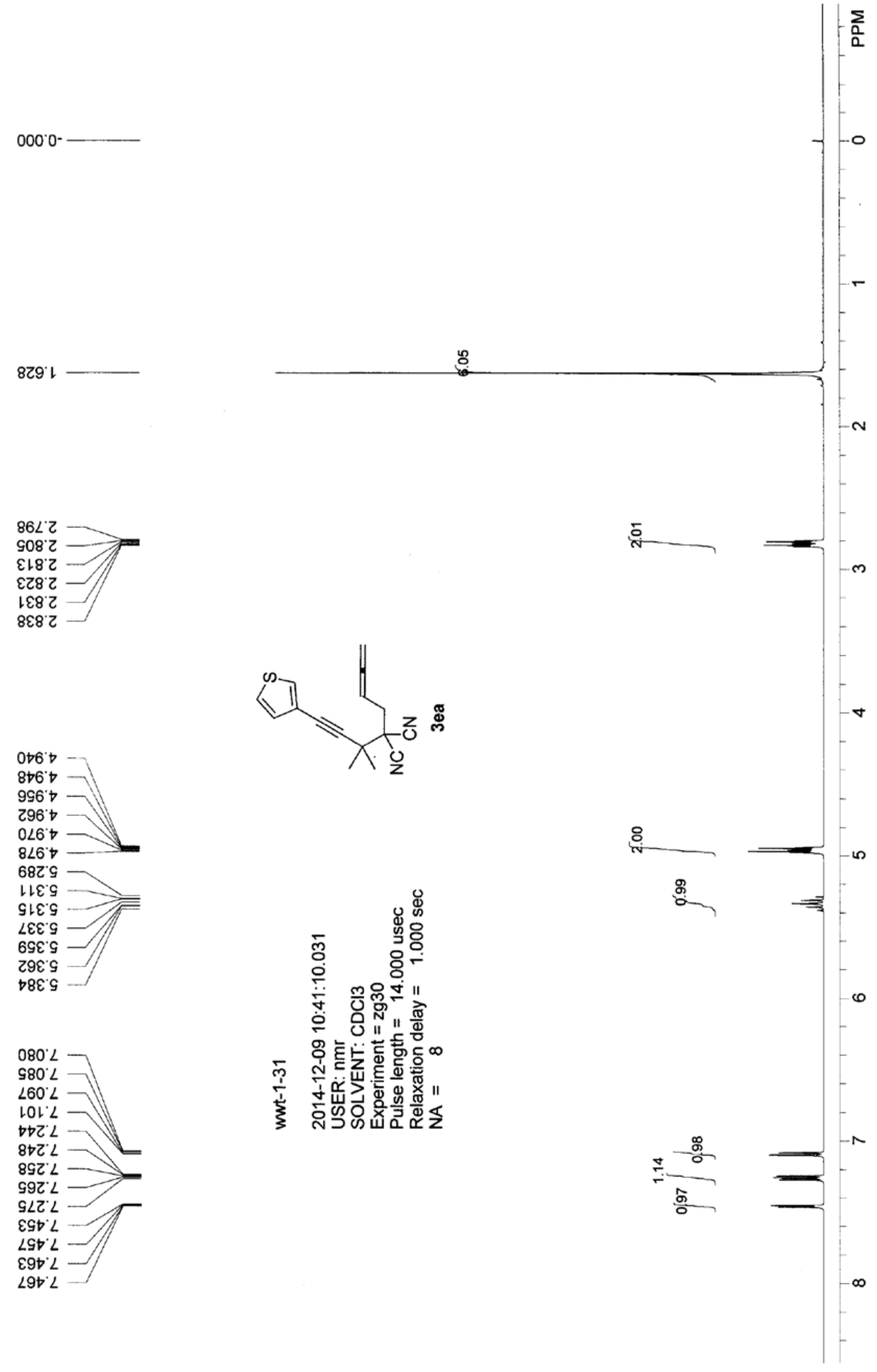

Supplementary Figure 33. **^{1}H NMR (300 MHz, $CDCl_3$) spectrum for 3ea.**

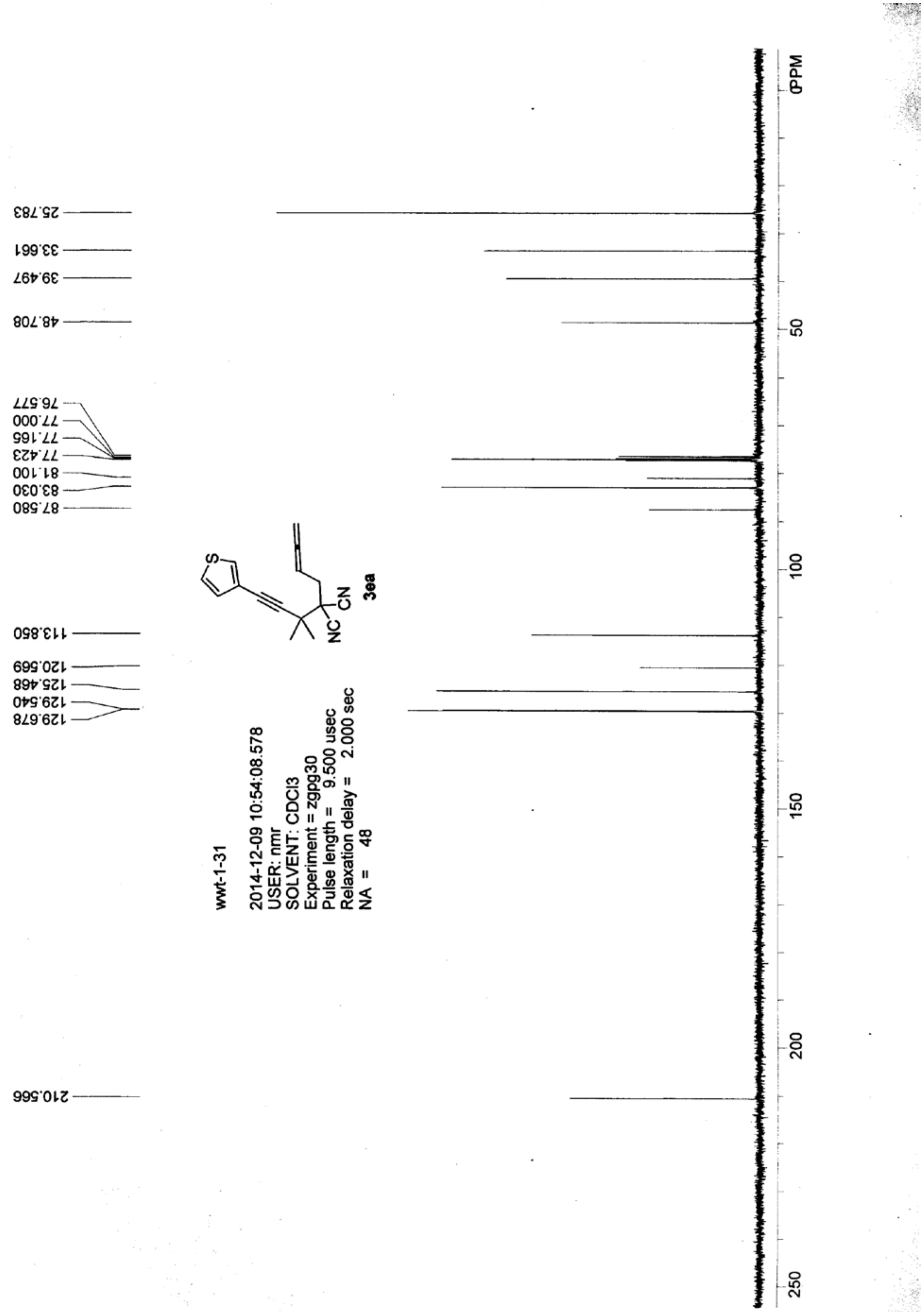

Supplementary Figure 34. ^{13}C NMR (75 MHz, $CDCl_3$) spectrum for 3ea.

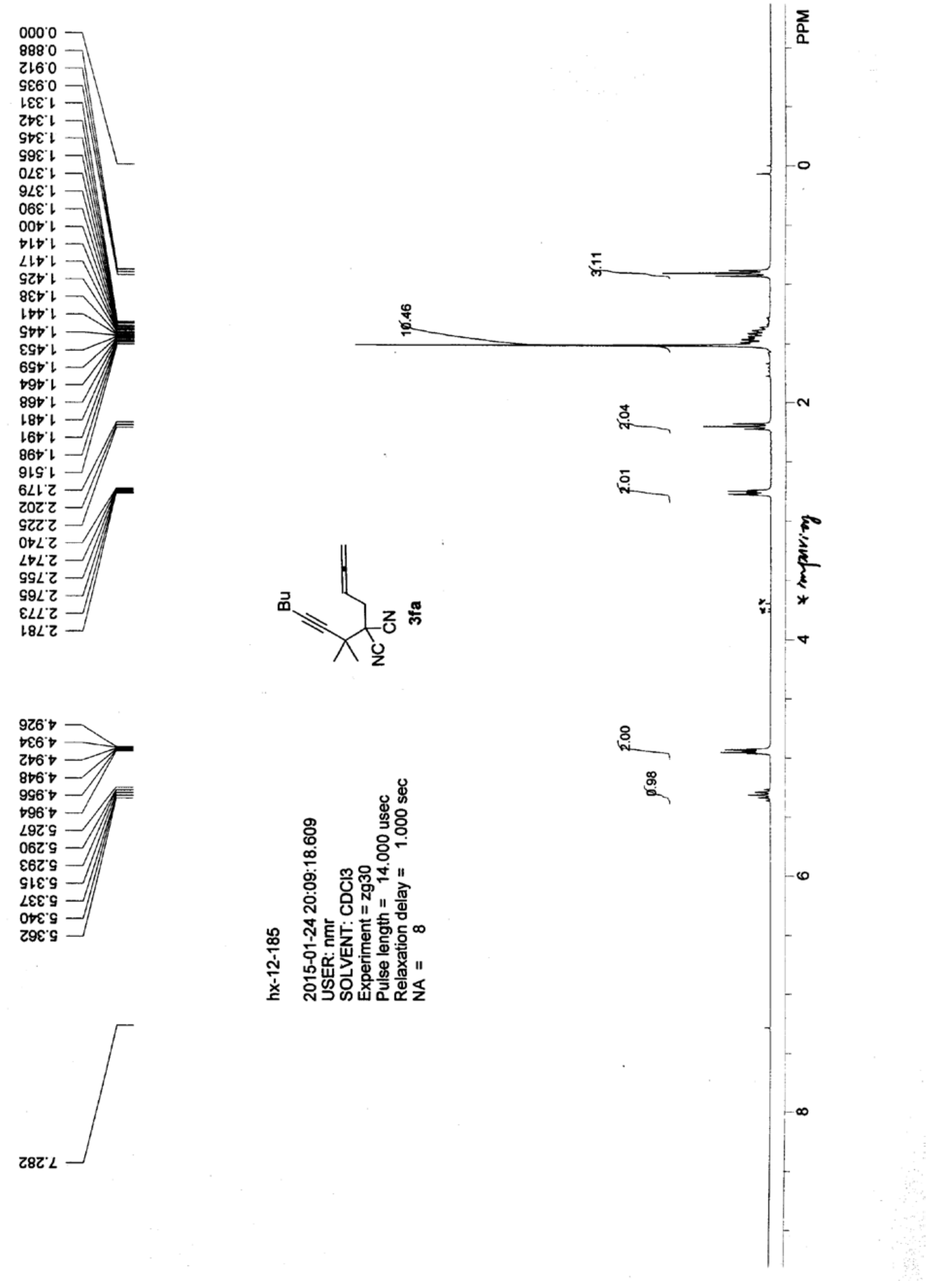

Supplementary Figure 35. ^{1}H NMR (300 MHz, $CDCl_3$) spectrum for 3fa.

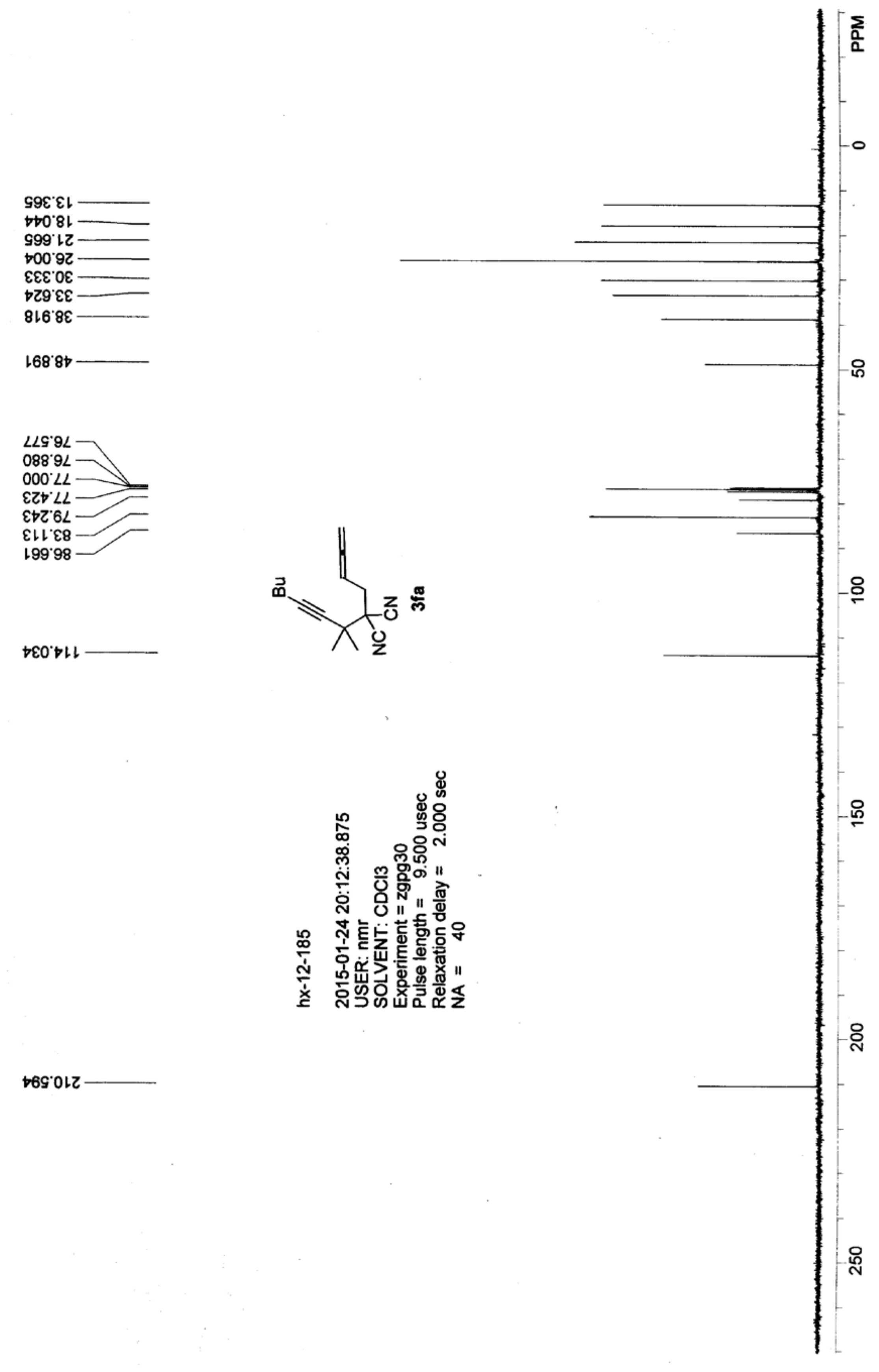

Supplementary Figure 36. **^{13}C NMR (75 MHz, $CDCl_3$) spectrum for 3fa.**

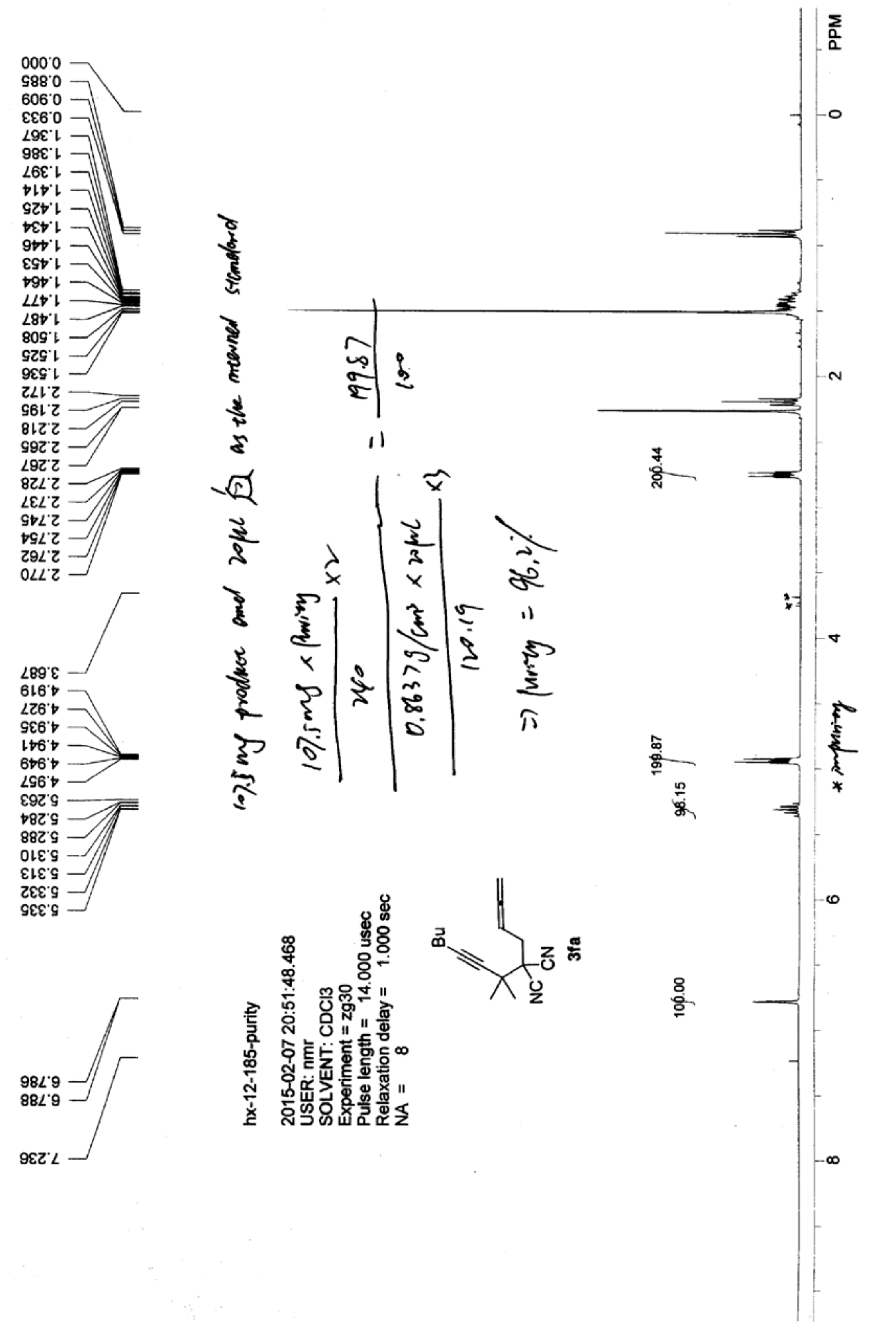

Supplementary Figure 37. ^{1}H NMR (300 MHz, $CDCl_3$) spectrum for the purity of 3fa.

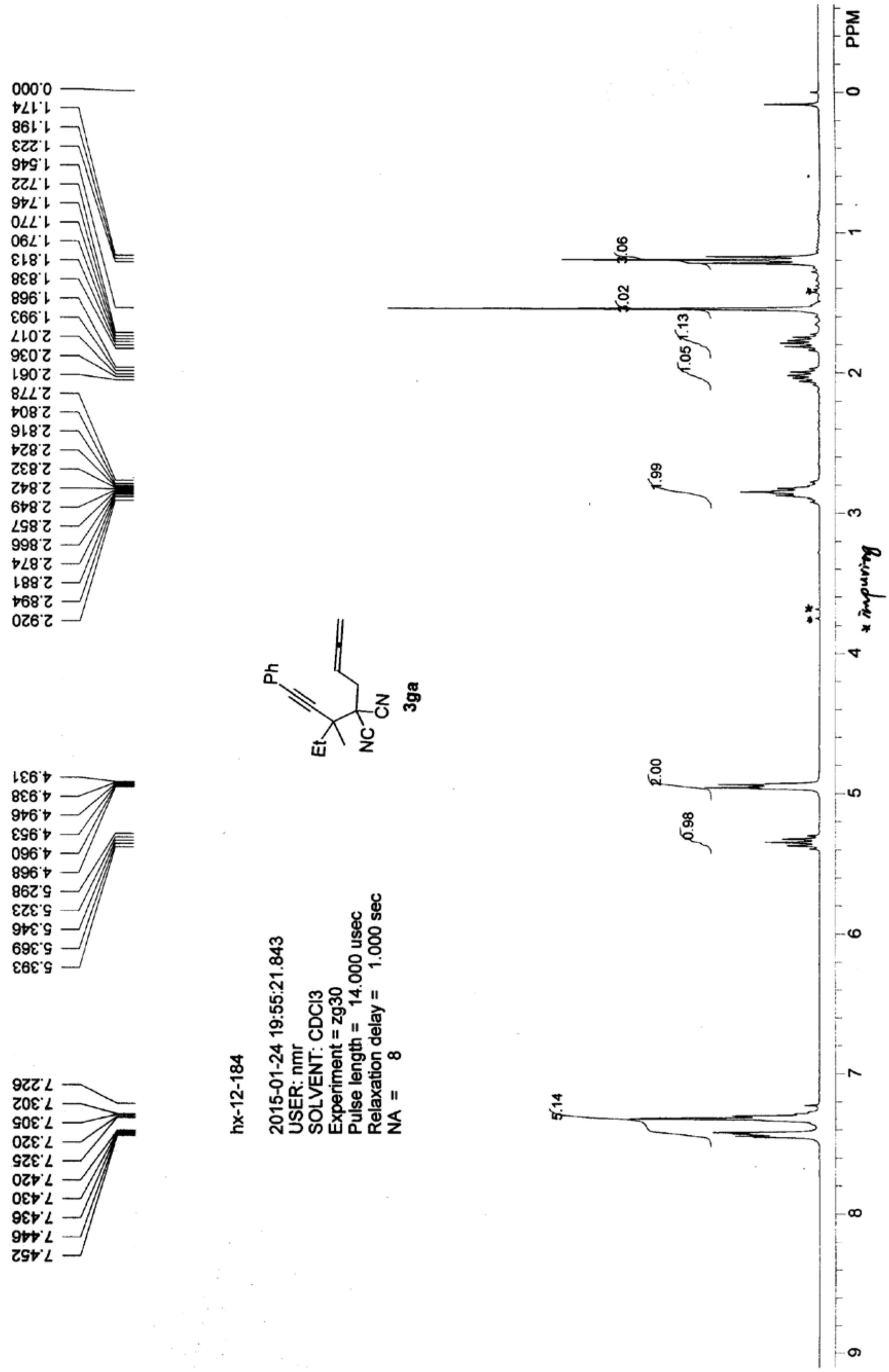

Supplementary Figure 38. ^{1}H NMR (300 MHz, $CDCl_3$) spectrum for 3ga.

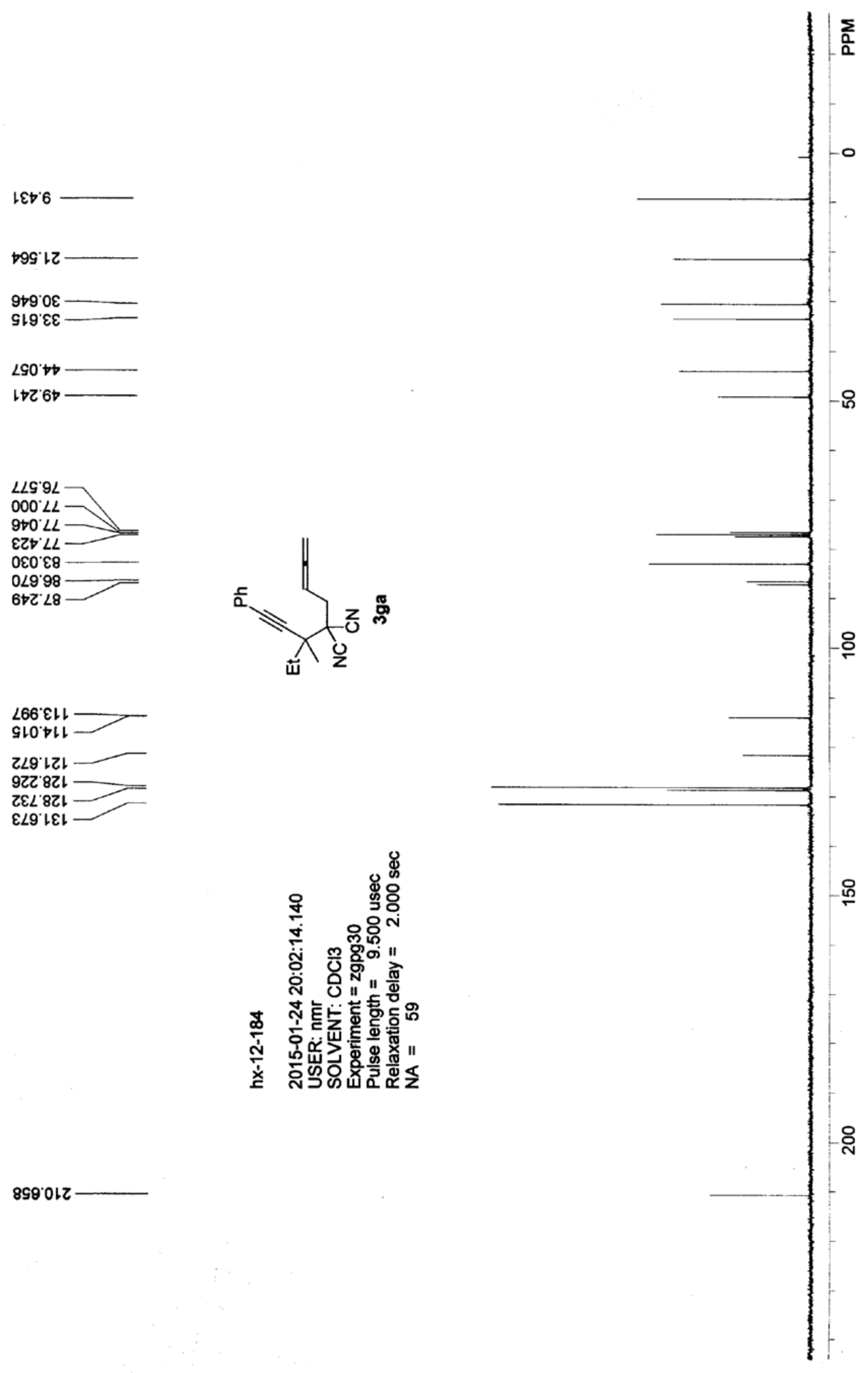

Supplementary Figure 39. ^{13}C NMR (75 MHz, $CDCl_3$) spectrum for 3ga.

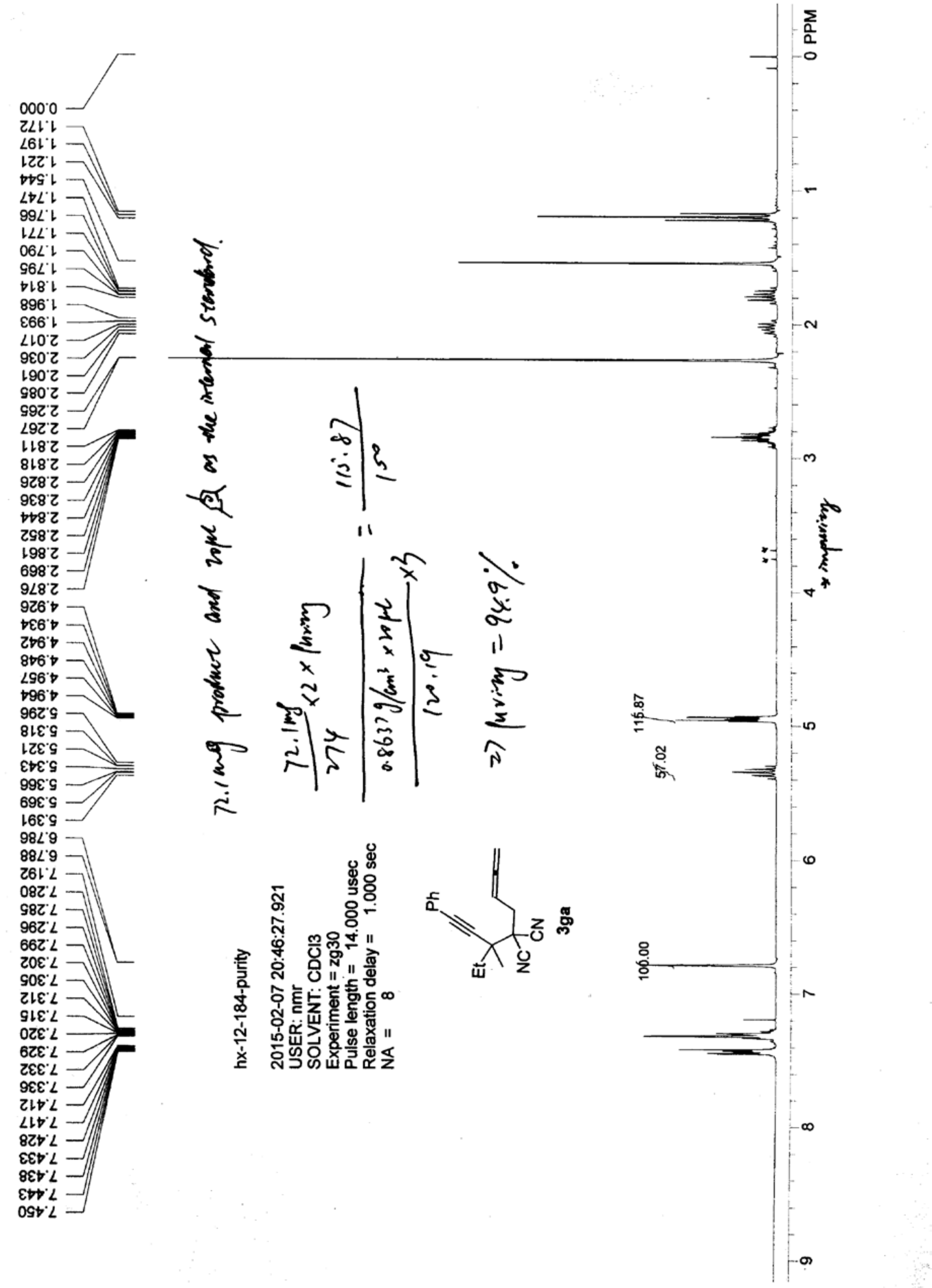

Supplementary Figure 40. ^{1}**H NMR (300 MHz,** $CDCl_3$**) spectrum for the purity of 3ga.**

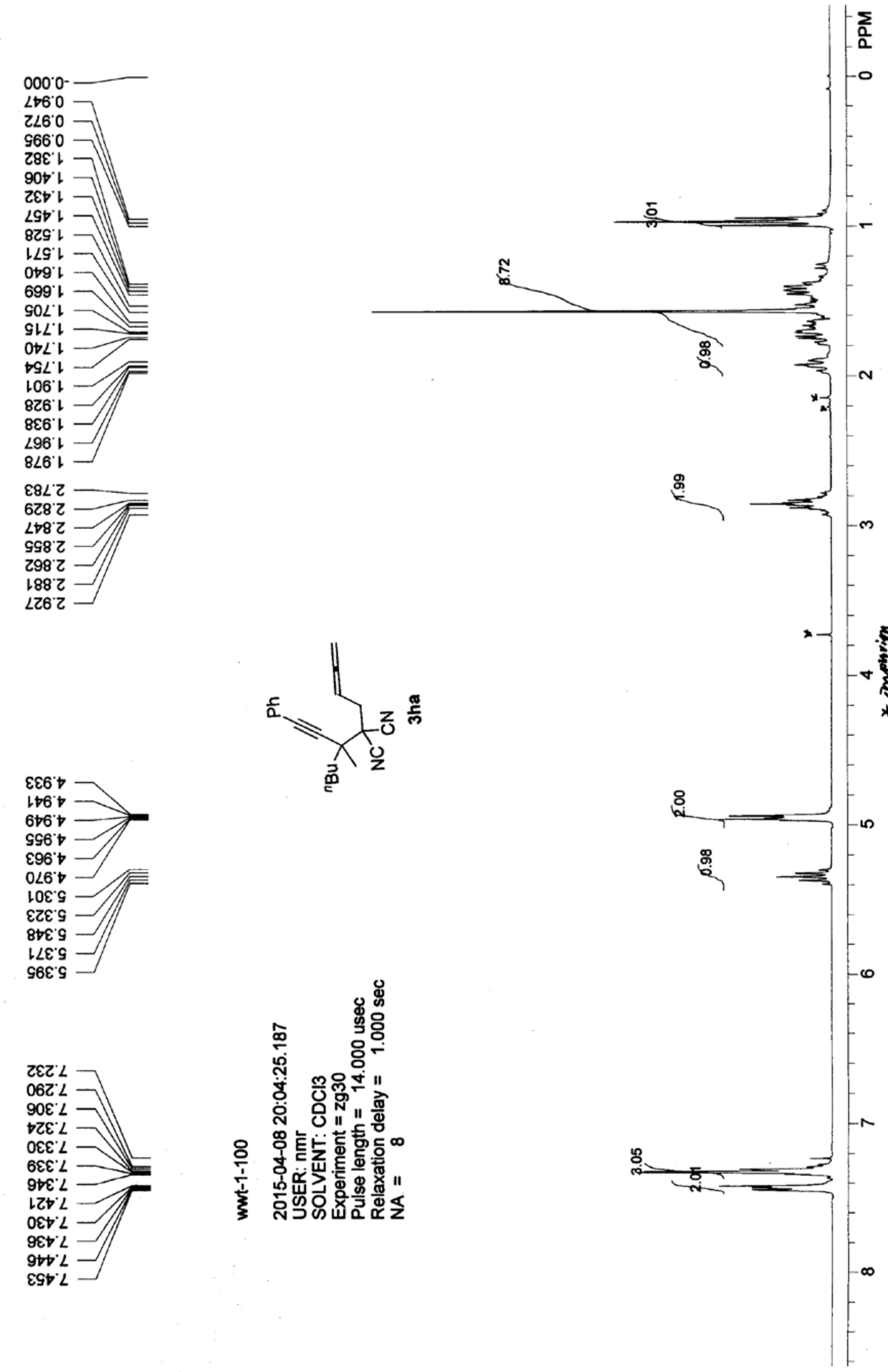

Supplementary Figure 41. ^{1}H NMR (300 MHz, $CDCl_3$) spectrum for 3ha.

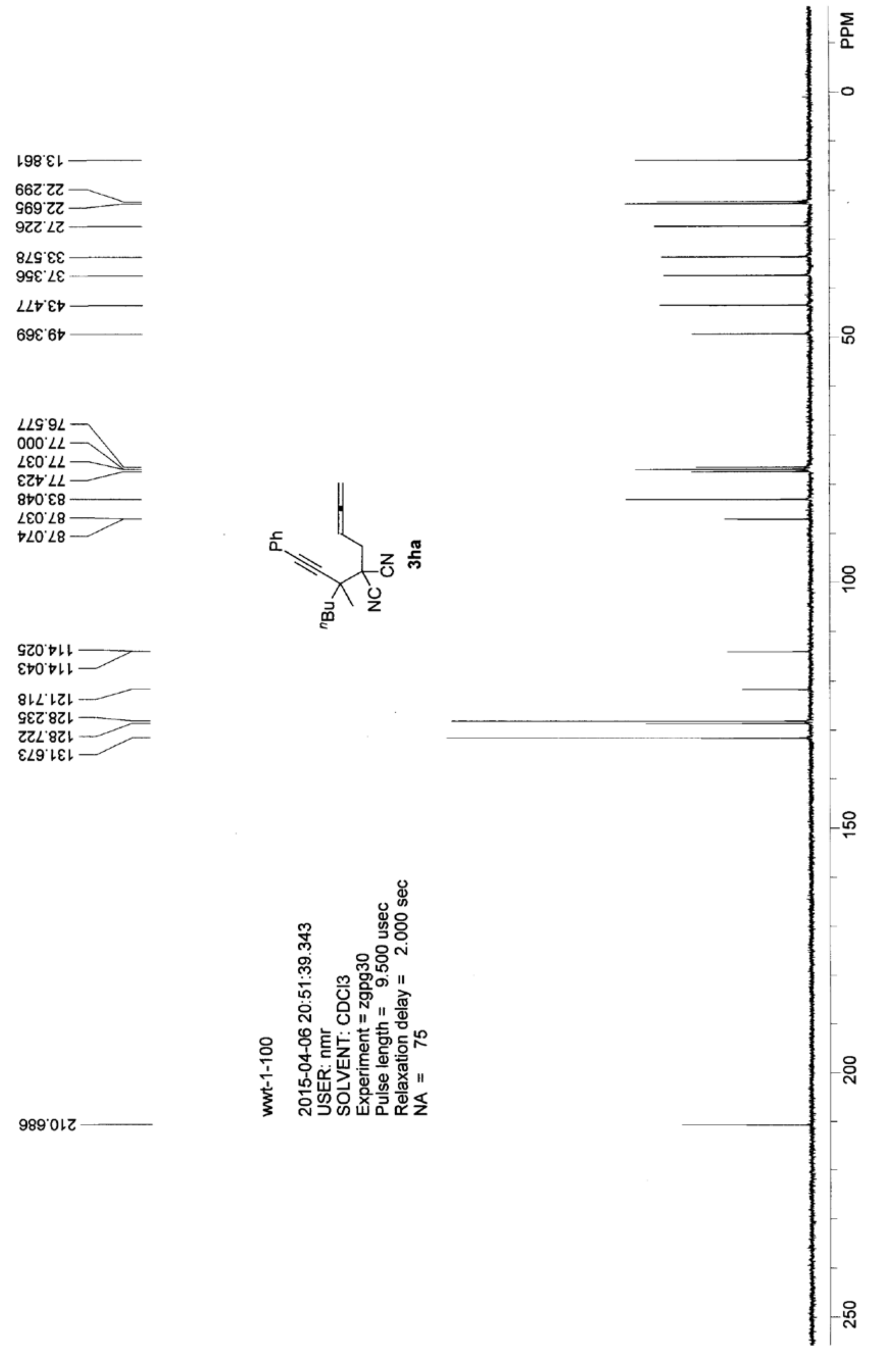

Supplementary Figure 42. ^{13}**C NMR (75 MHz, CDCl**$_3$**) spectrum for 3ha.**

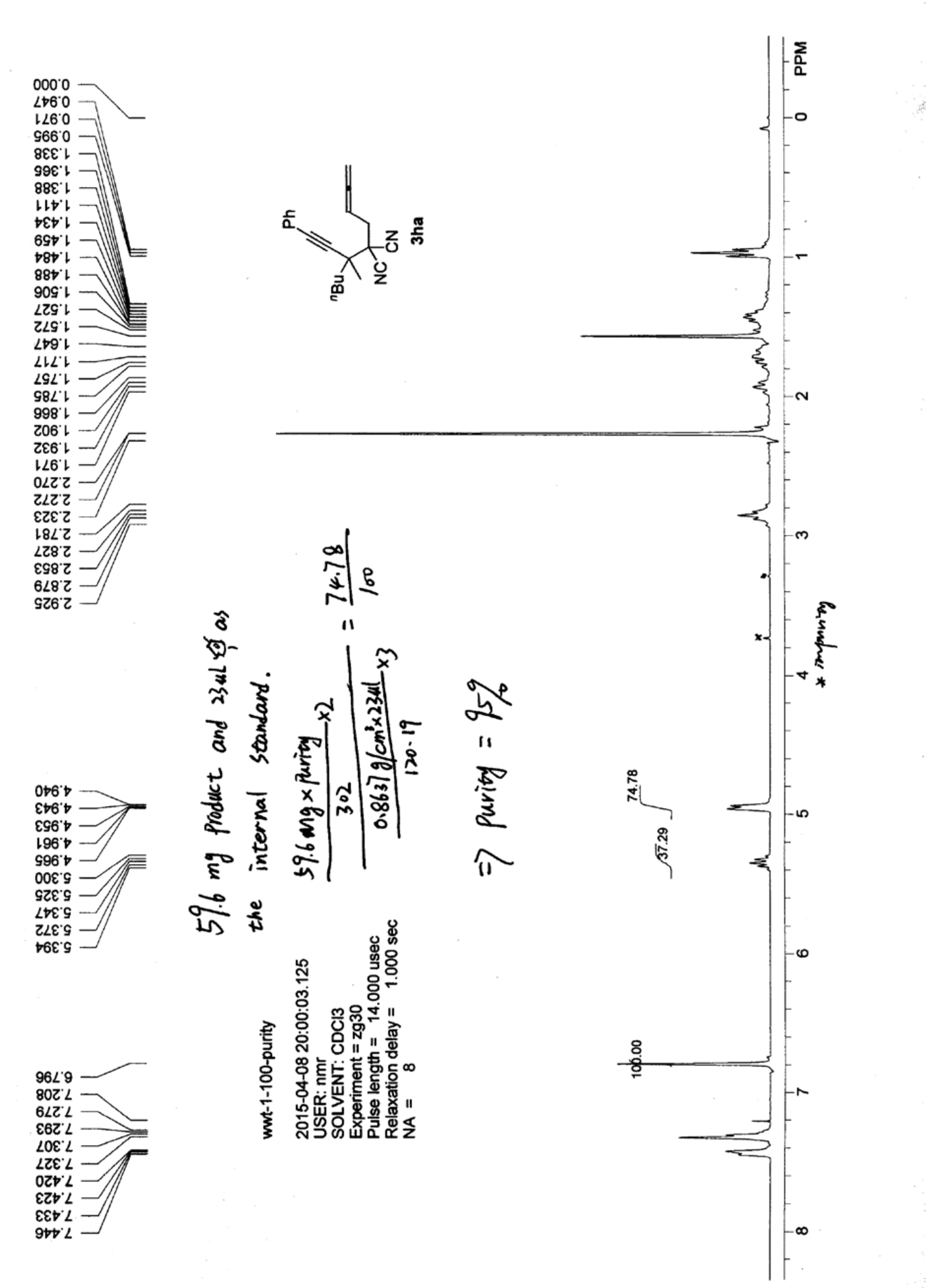

Supplementary Figure 43. ^{1}**H NMR (300 MHz,** $CDCl_3$**) spectrum for the purity of 3ha.**

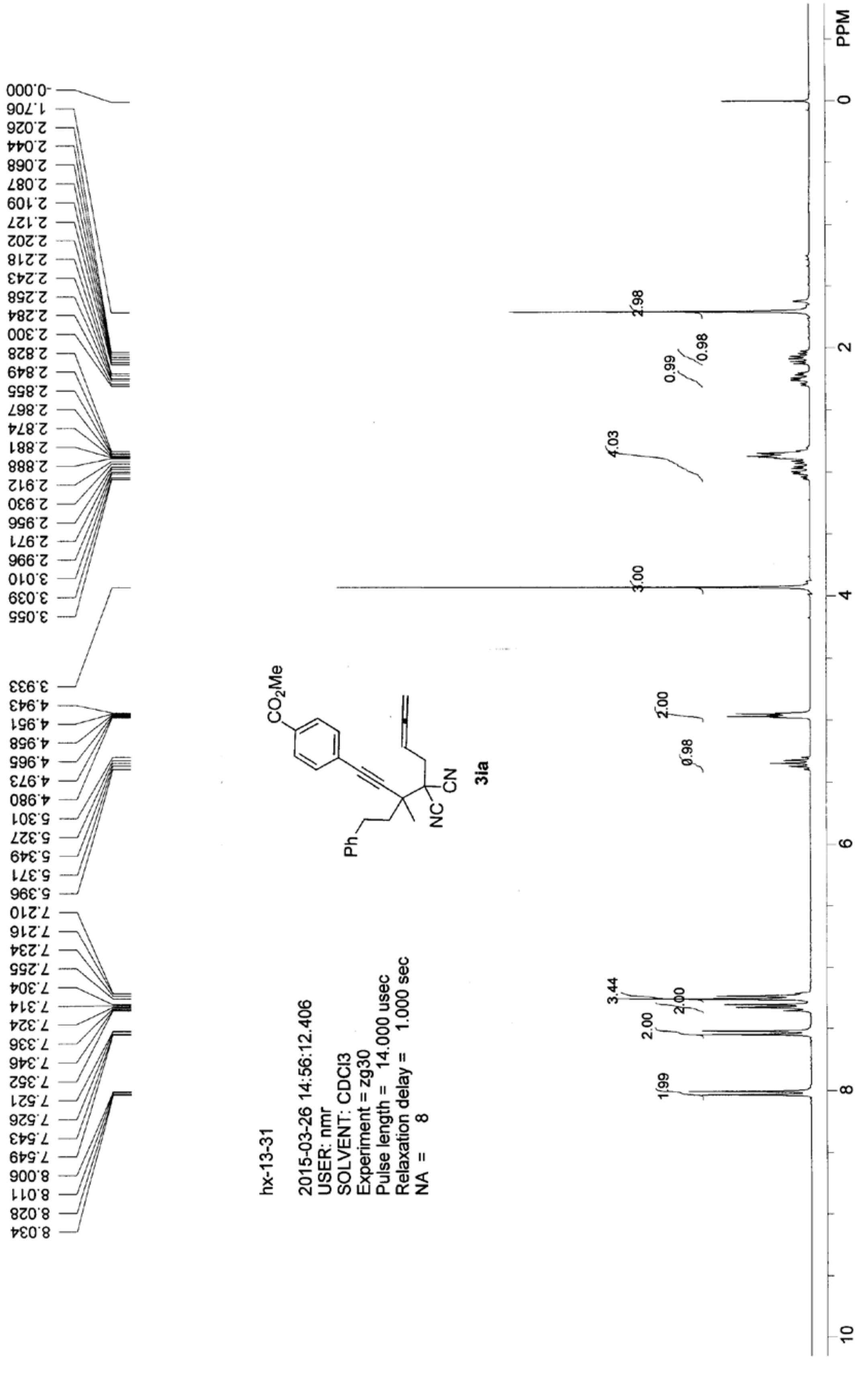

Supplementary Figure 44. ^{1}H NMR (300 MHz, $CDCl_3$) spectrum for 3ia.

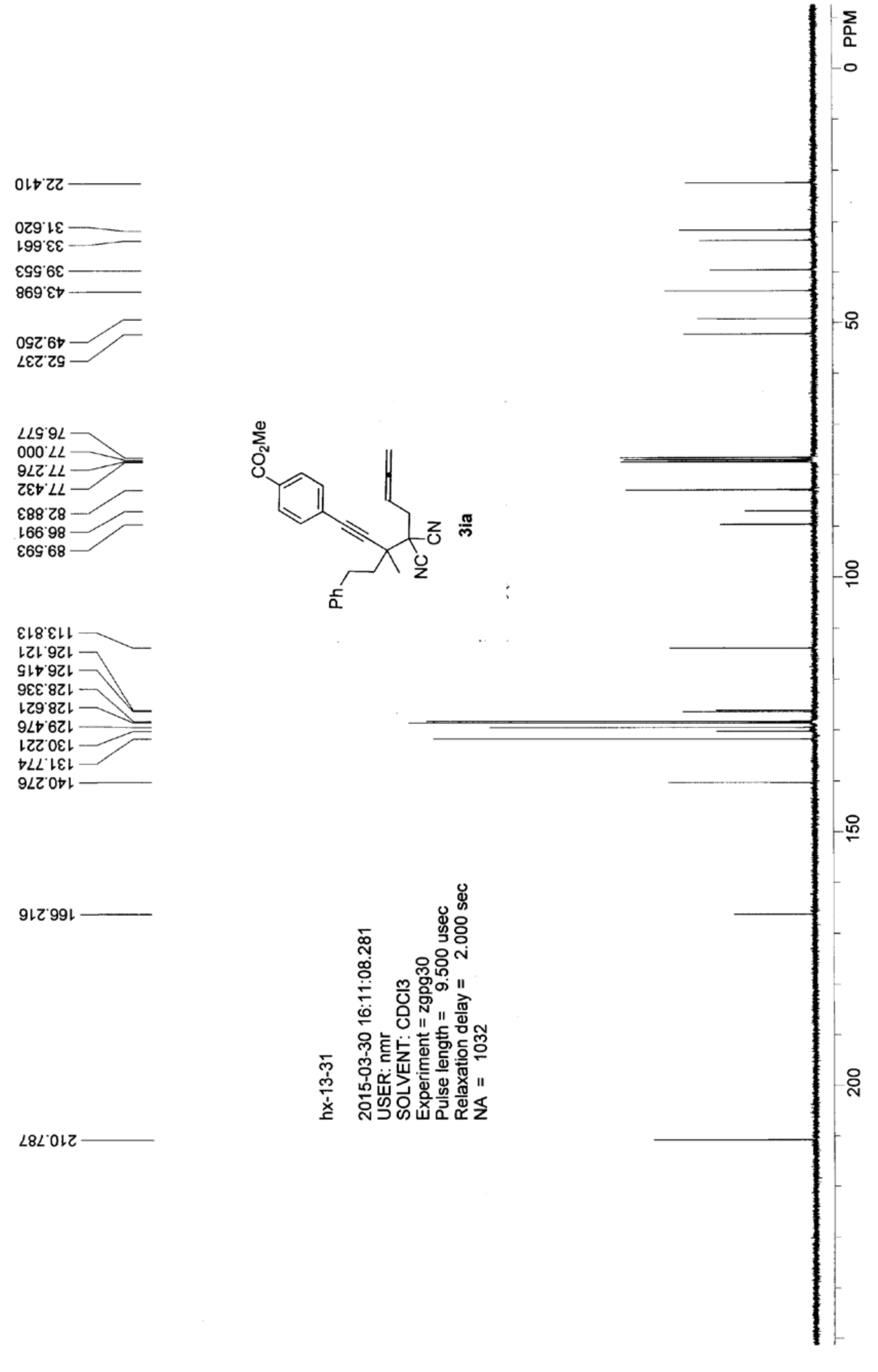

Supplementary Figure 45. ^{13}**C NMR (75 MHz, CDCl$_3$) spectrum for 3ia.**

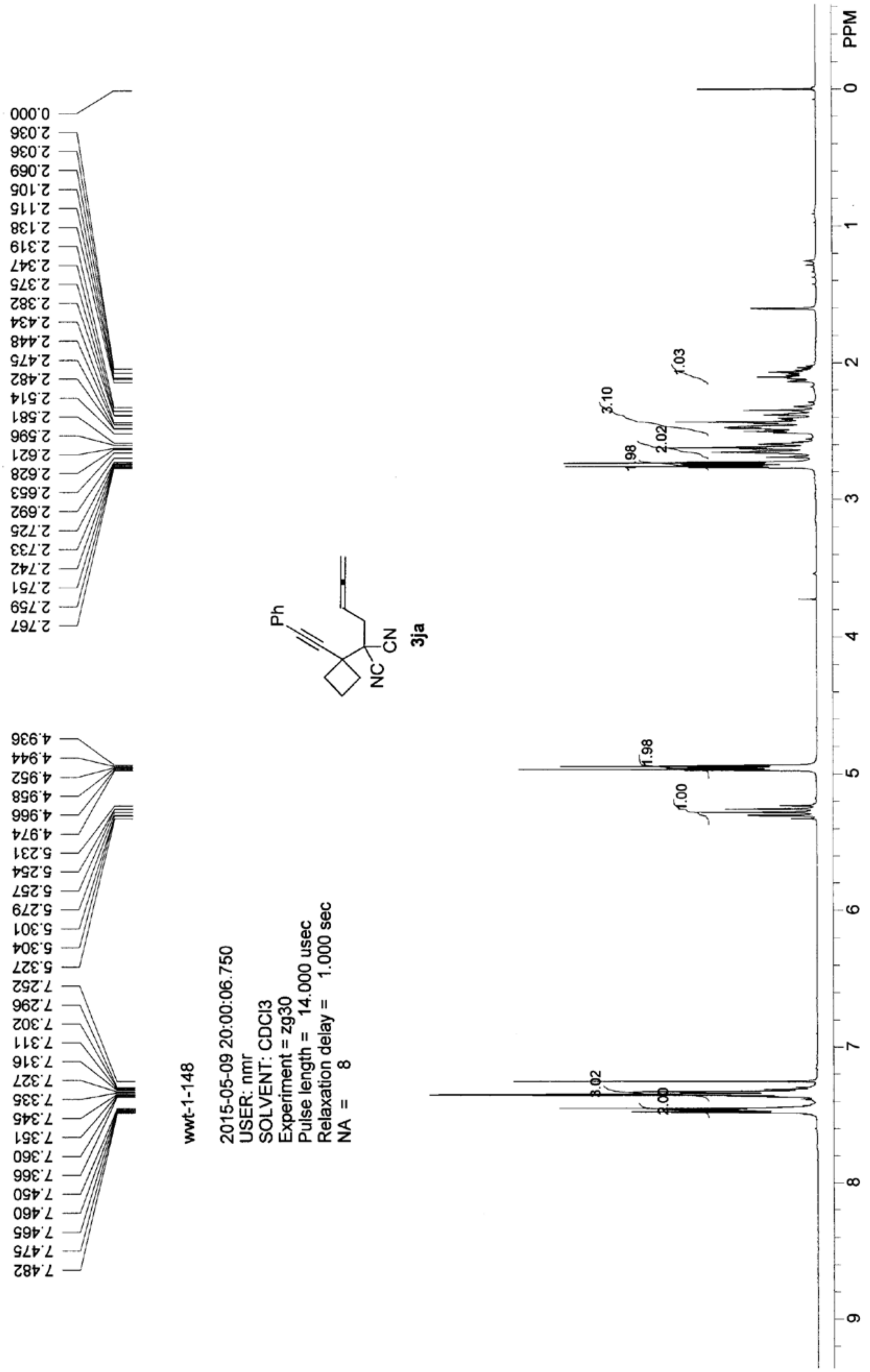

Supplementary Figure 46. ^{1}H NMR (300 MHz, $CDCl_3$) spectrum for 3ja.

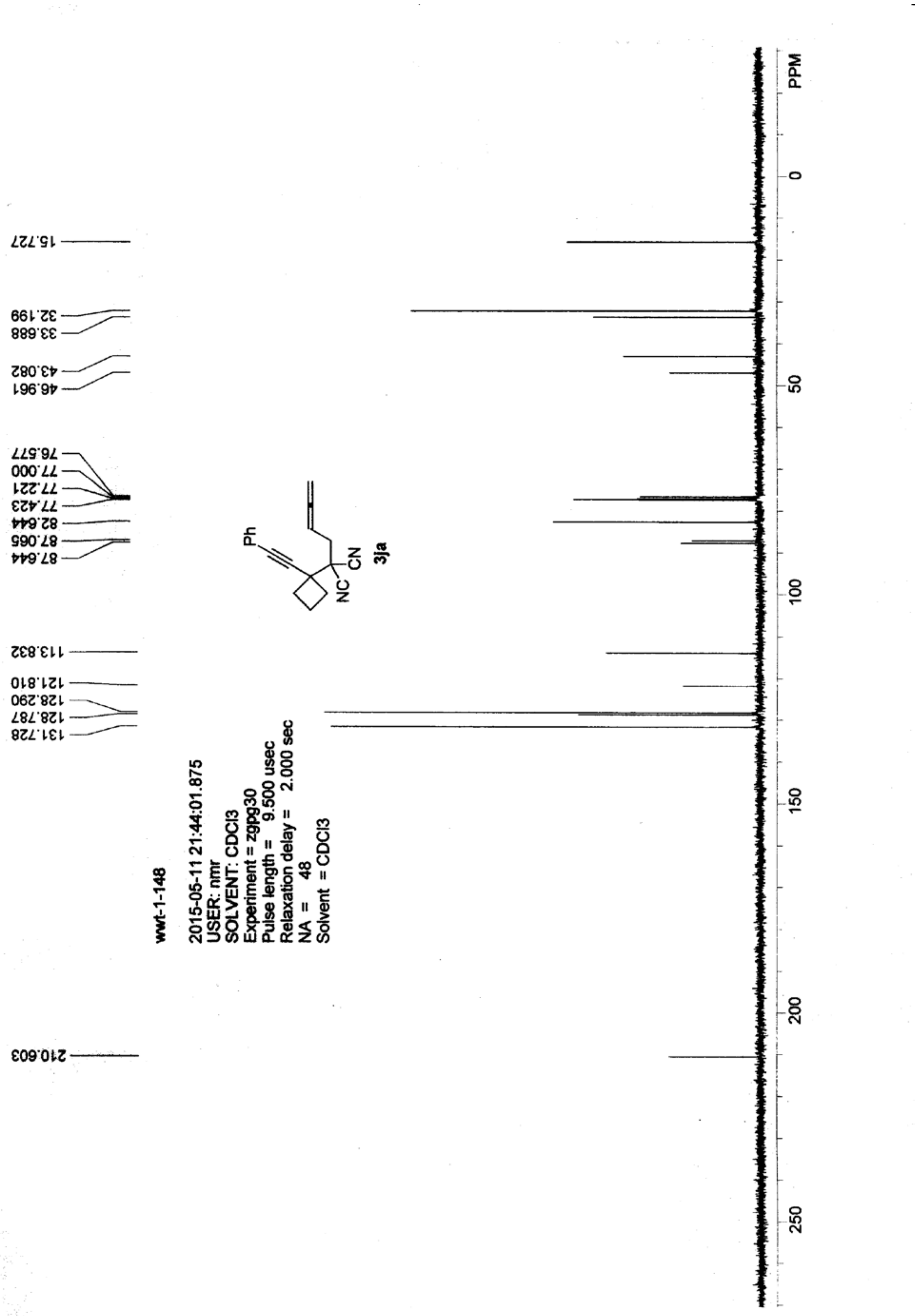

Supplementary Figure 47. ^{13}**C NMR (75 MHz, CDCl$_3$) spectrum for 3ja.**

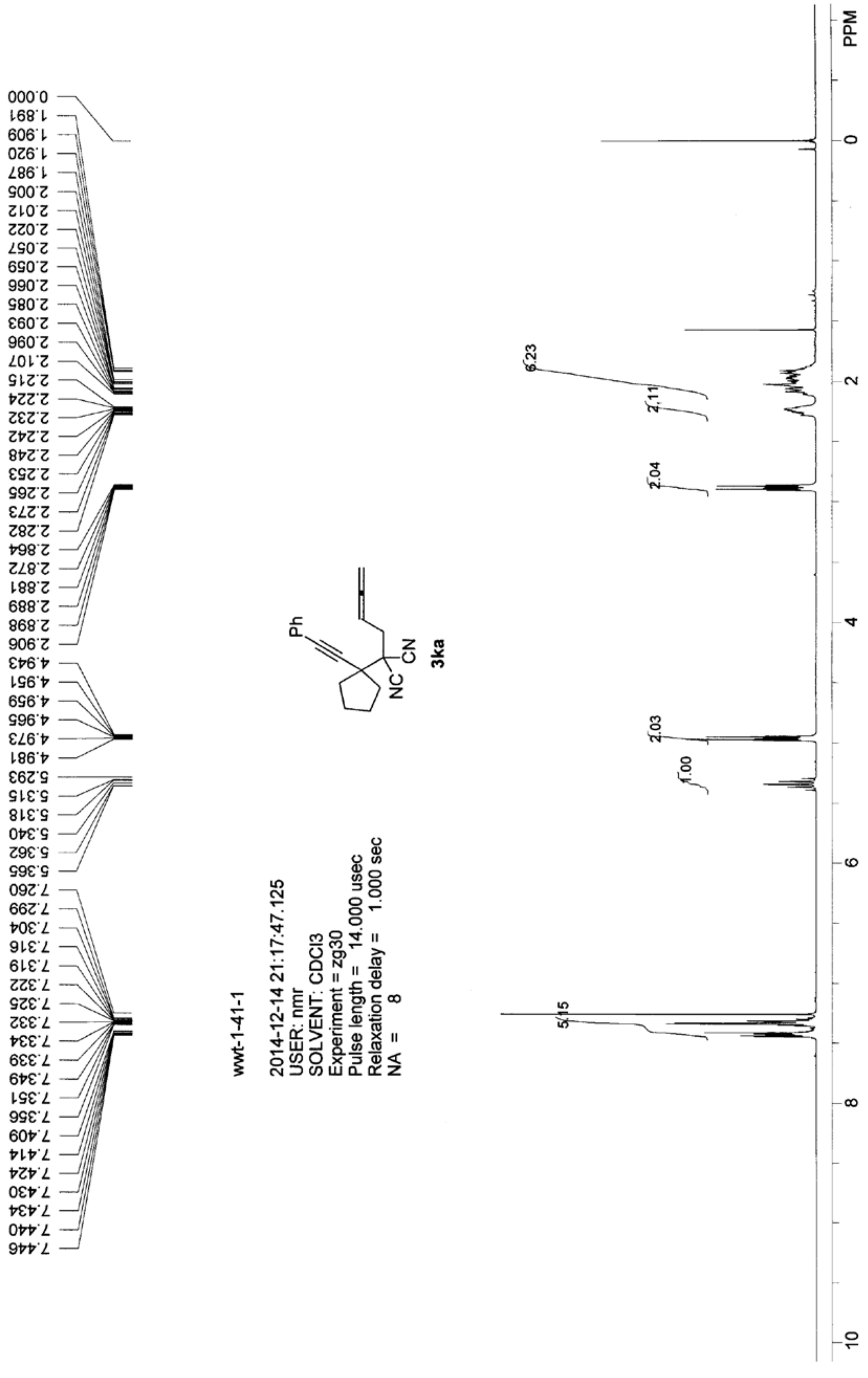

Supplementary Figure 48. **^{1}H NMR (300 MHz, $CDCl_3$) spectrum for 3ka.**

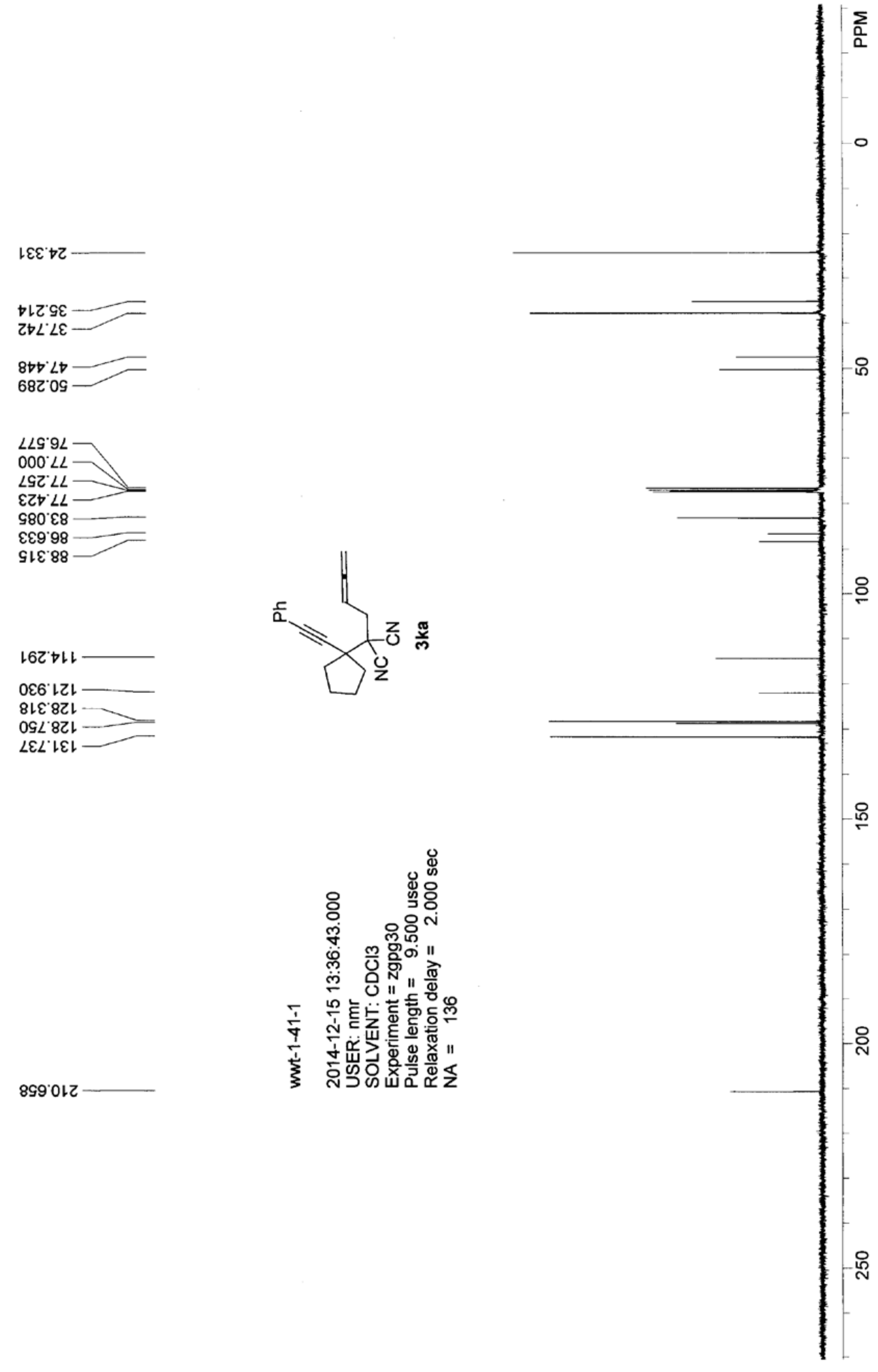

Supplementary Figure 49. ^{13}**C NMR (75 MHz, CDCl$_3$) spectrum for 3ka.**

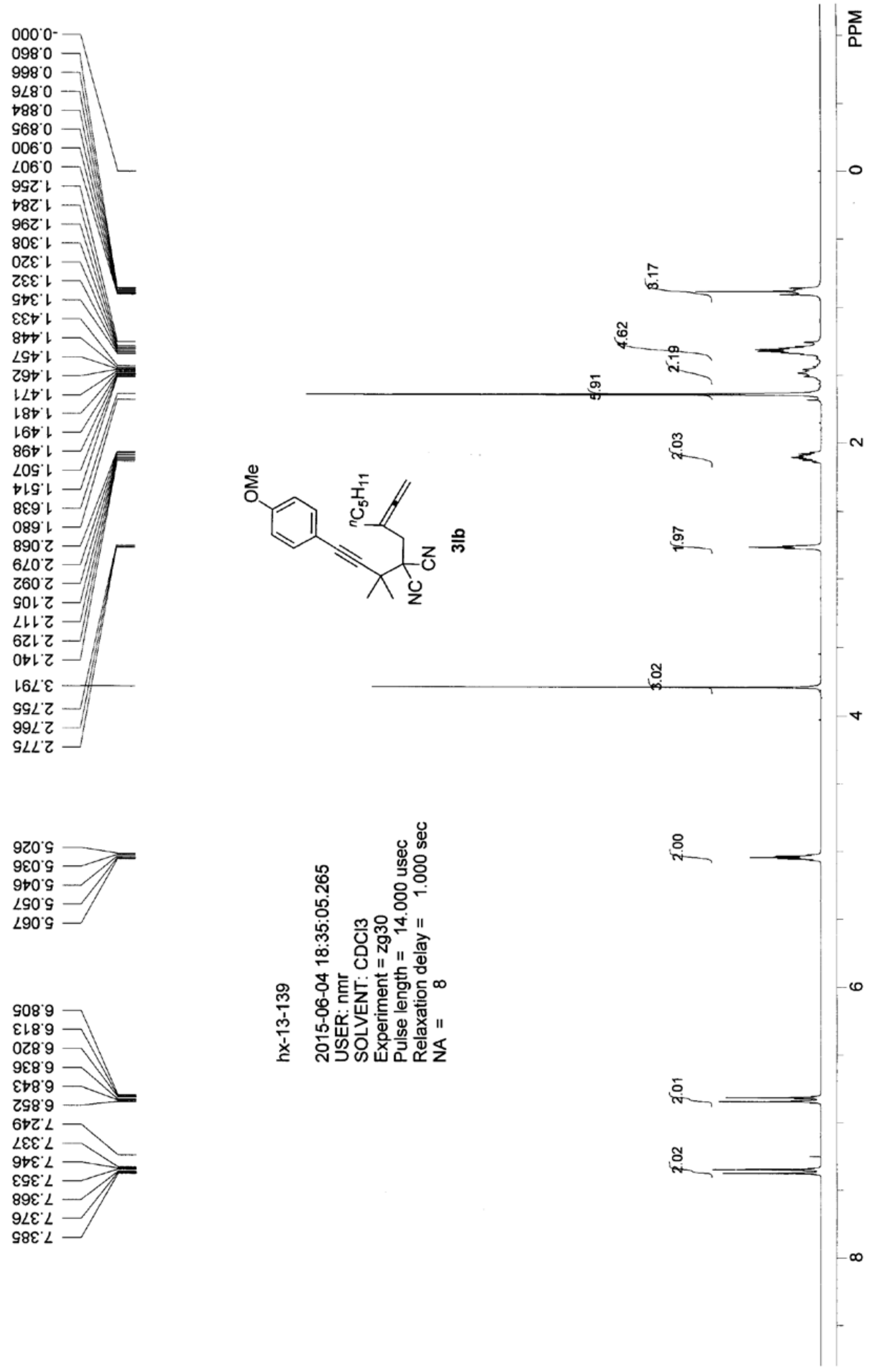

Supplementary Figure 50. ^{1}**H NMR (300 MHz, CDCl$_3$) spectrum for 3lb.**

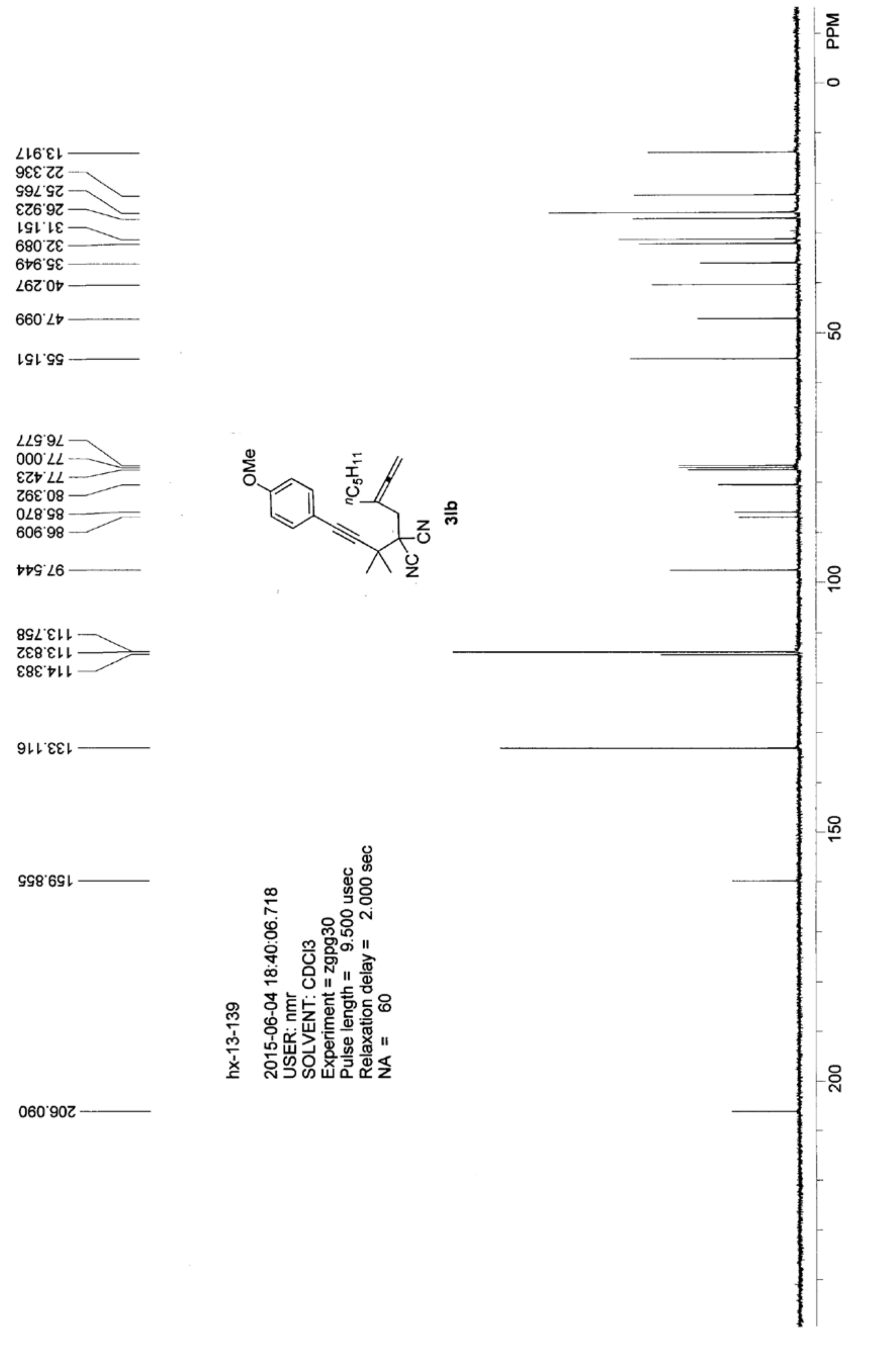

Supplementary Figure 51. **^{13}C NMR (75 MHz, $CDCl_3$) spectrum for 3lb.**

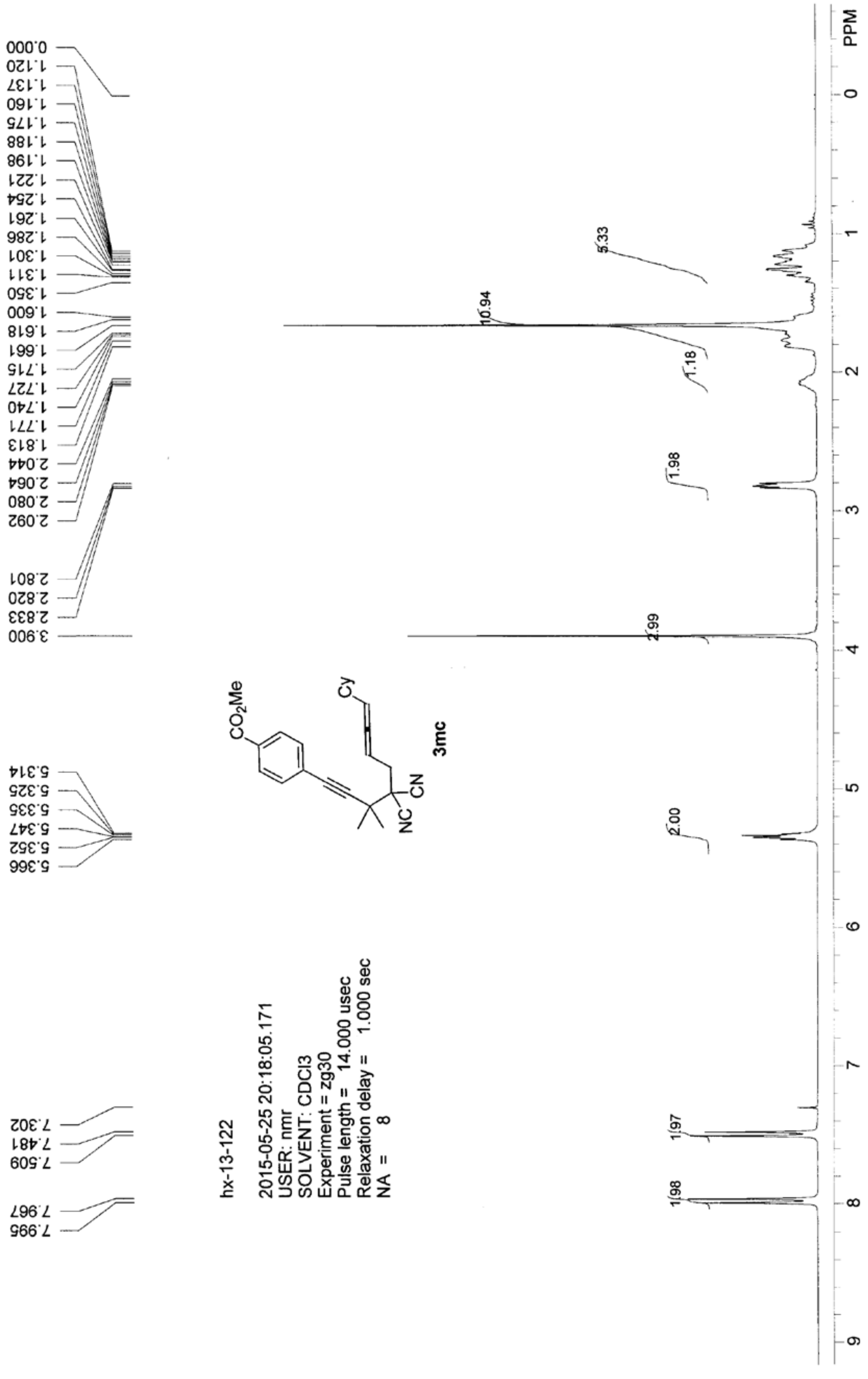

Supplementary Figure 52. **^{1}H NMR (300 MHz, $CDCl_3$) spectrum for 3mc.**

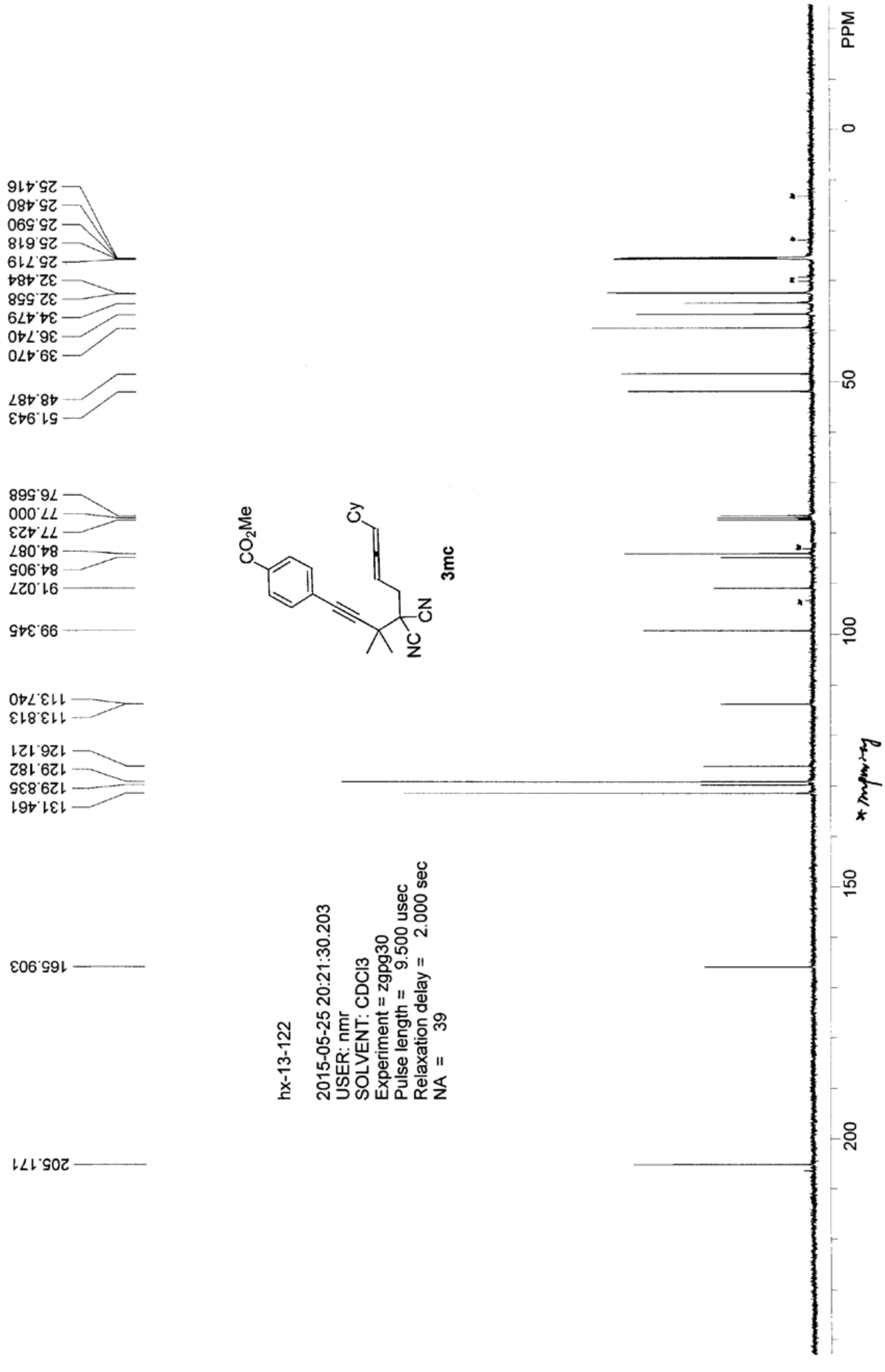

Supplementary Figure 53. ^{13}**C NMR (75 MHz,** $CDCl_3$**) spectrum for 3mc.**

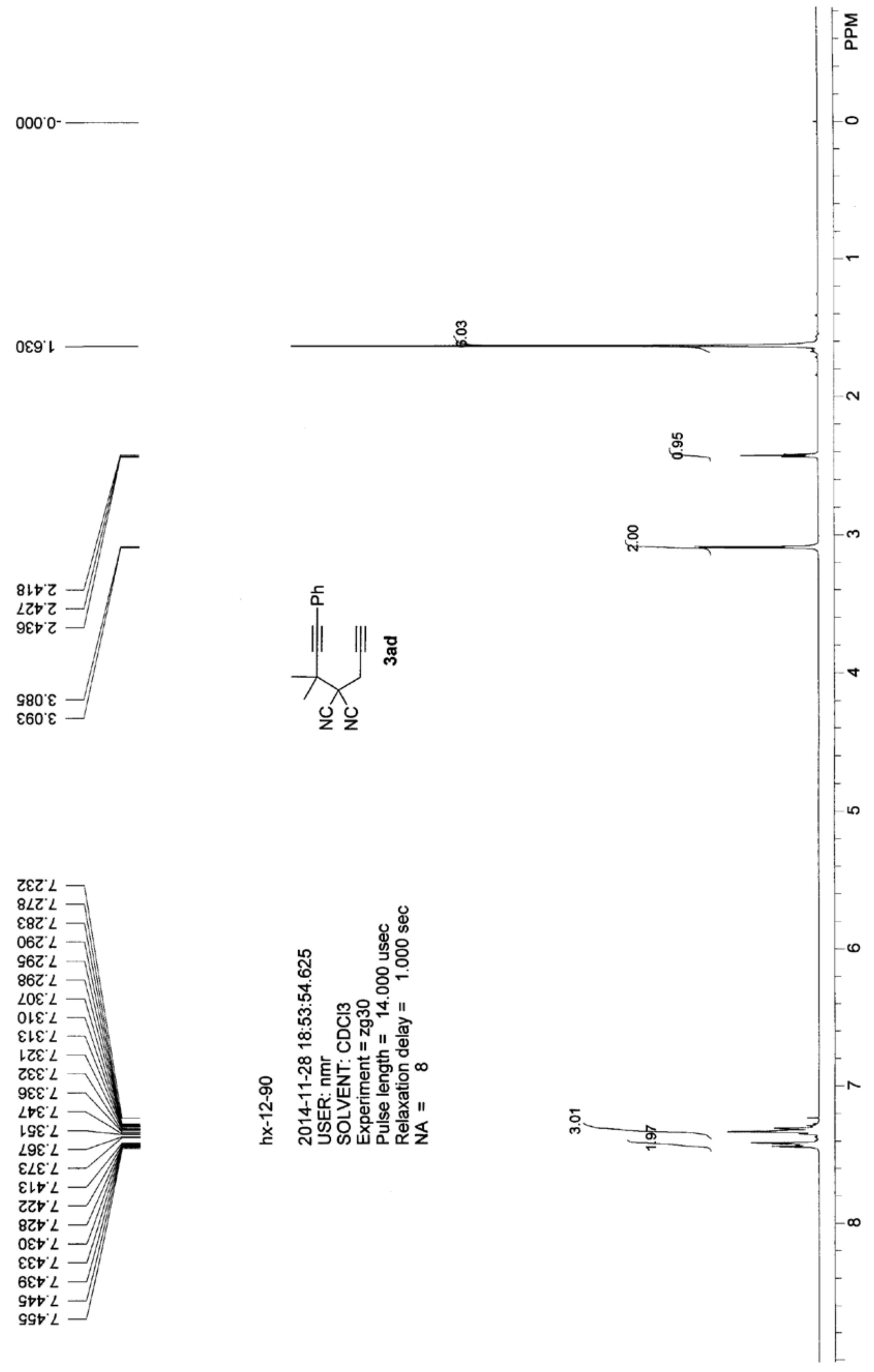

Supplementary Figure 54. ^{1}H NMR (300 MHz, $CDCl_3$) spectrum for 3ad.

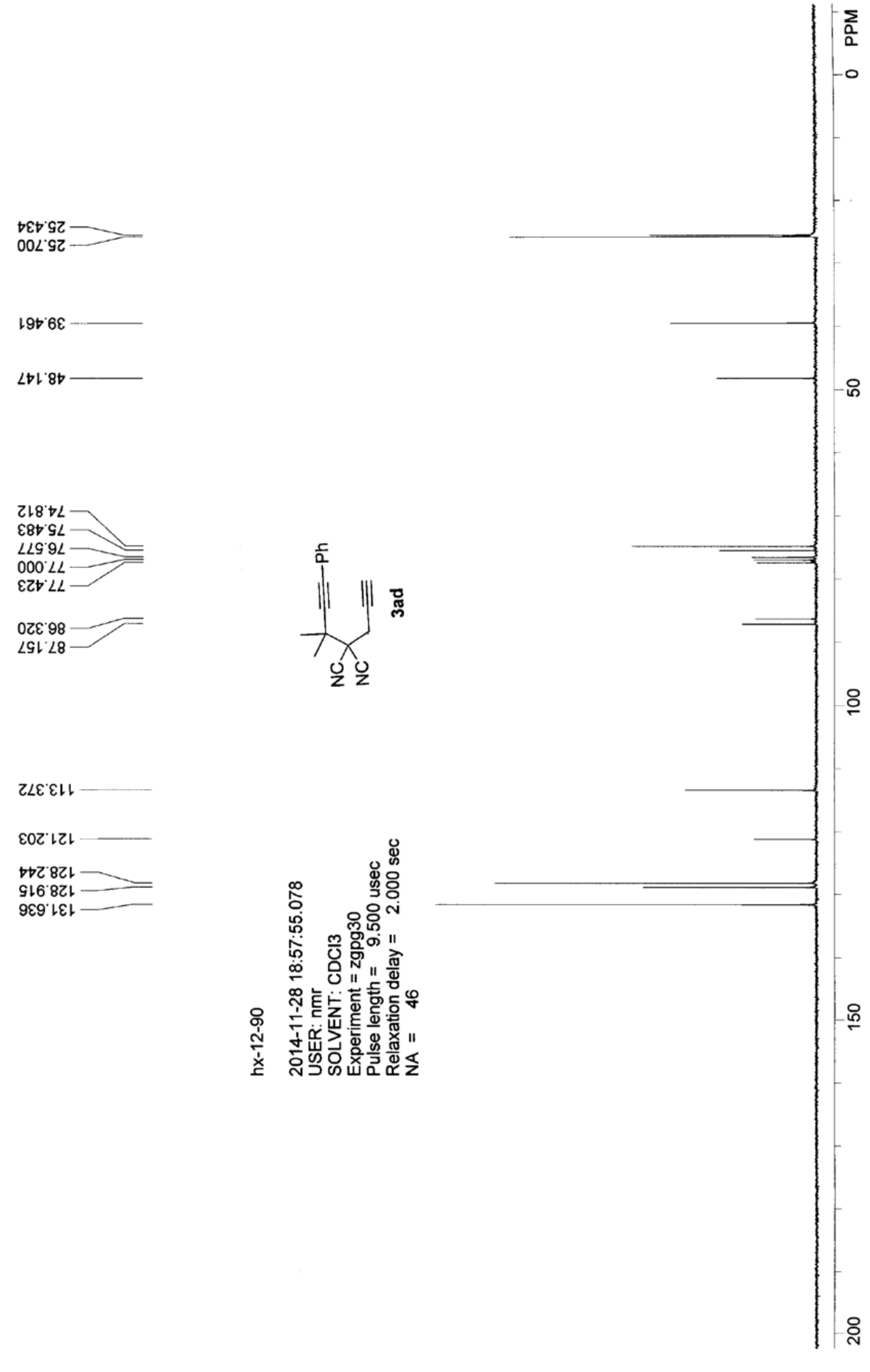

Supplementary Figure 55. ^{13}C NMR (75 MHz, $CDCl_3$) spectrum for 3ad.

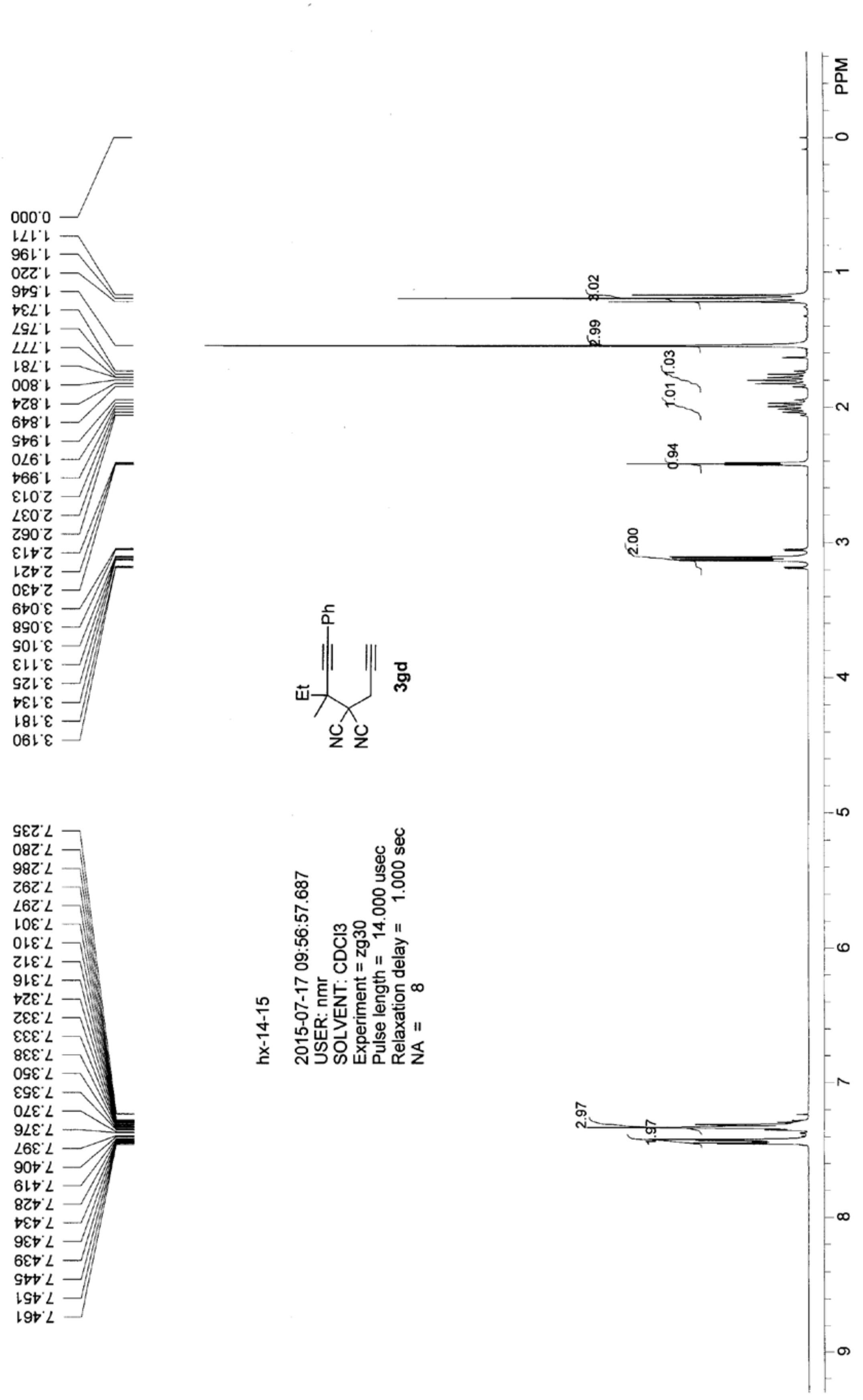

Supplementary Figure 56. ^{1}H NMR (300 MHz, $CDCl_3$) spectrum for 3gd.

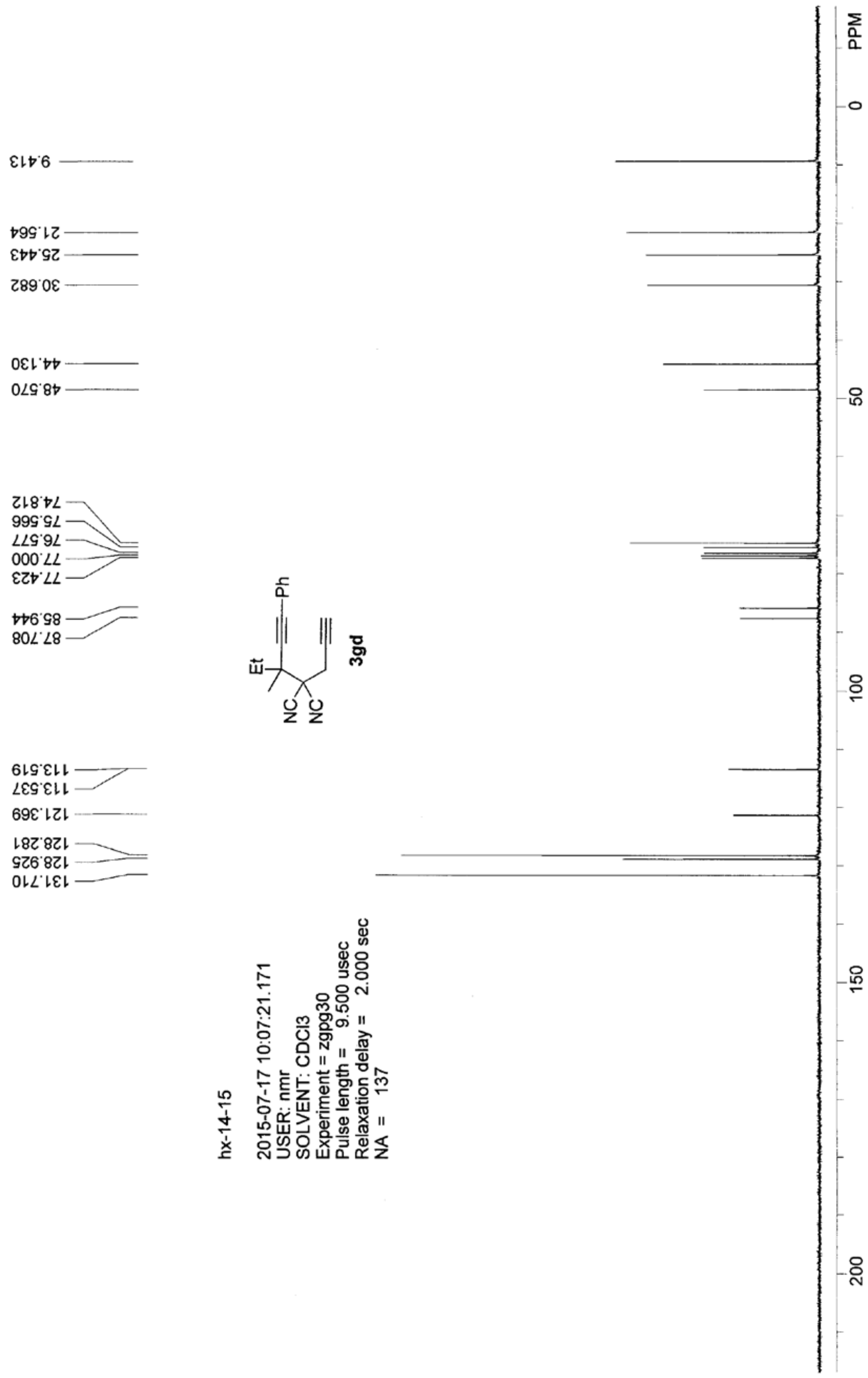

Supplementary Figure 57. ^{13}**C NMR (75 MHz, CDCl**$_3$**) spectrum for 3gd.**

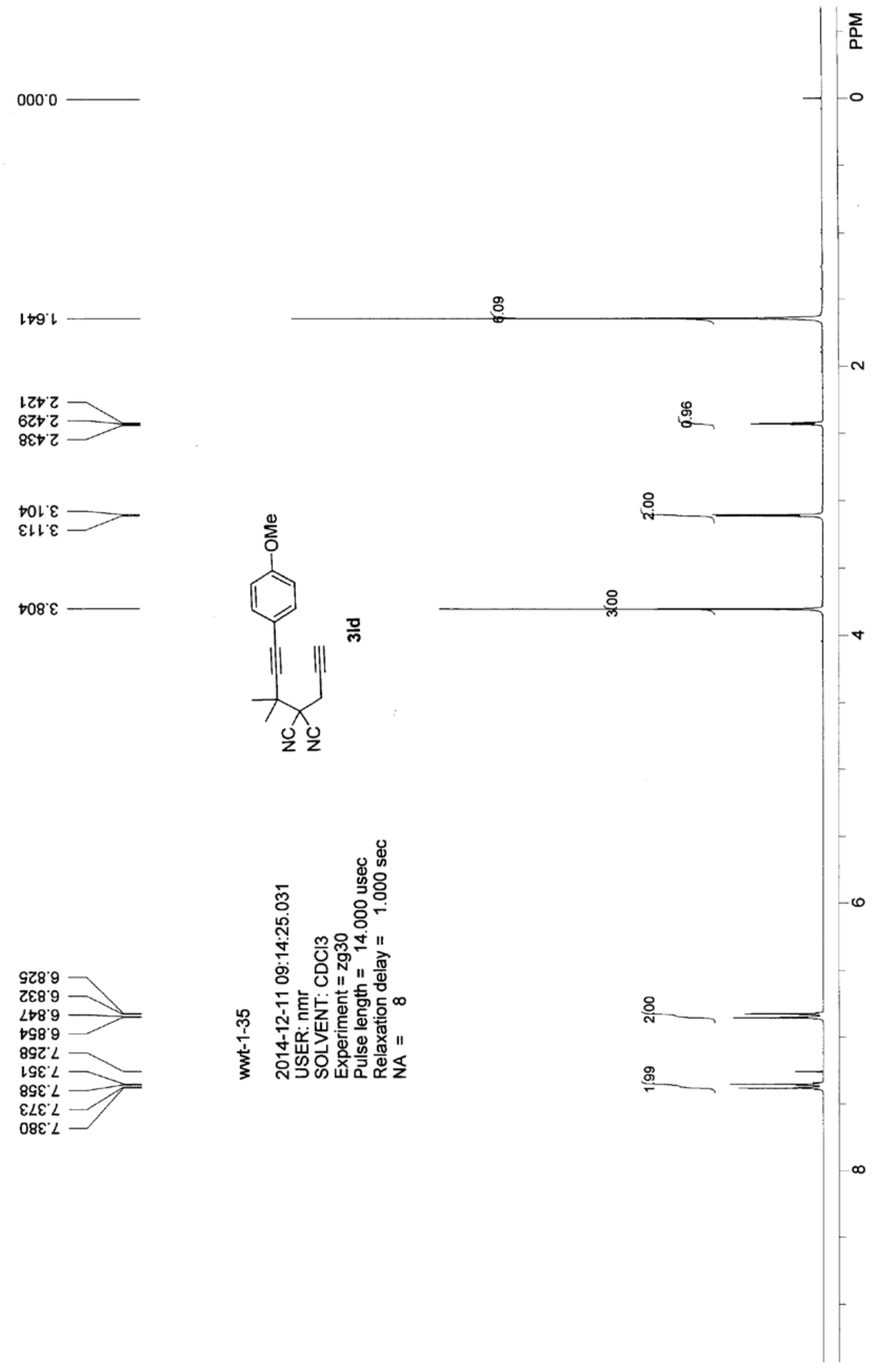

Supplementary Figure 58. **^{1}H NMR (300 MHz, $CDCl_3$) spectrum for 3ld.**

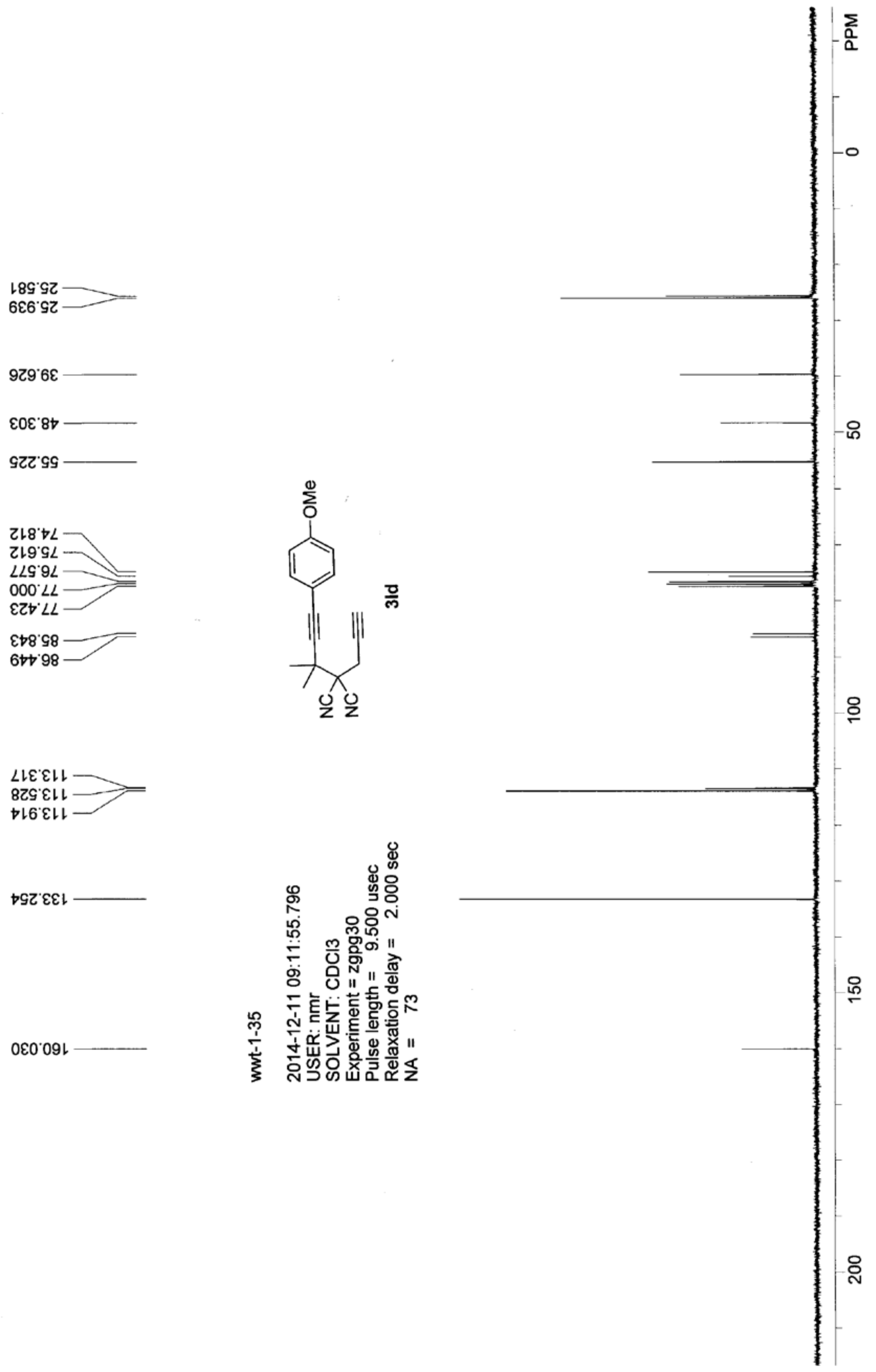

Supplementary Figure 59. ^{13}C **NMR (75 MHz,** $CDCl_3$**) spectrum for 3ld.**

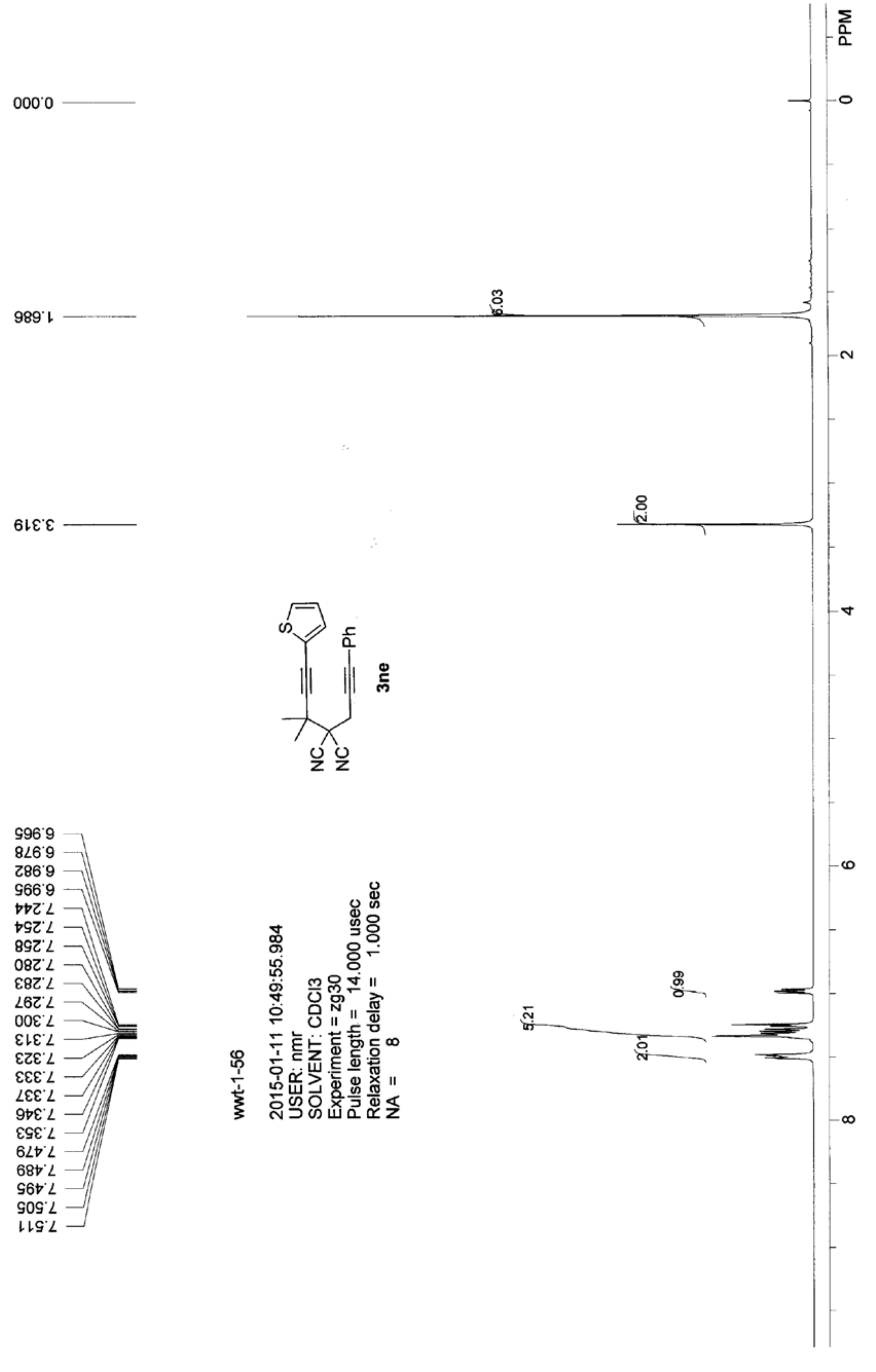

Supplementary Figure 60. ^{1}**H NMR (300 MHz,** $CDCl_3$**) spectrum for 3ne.**

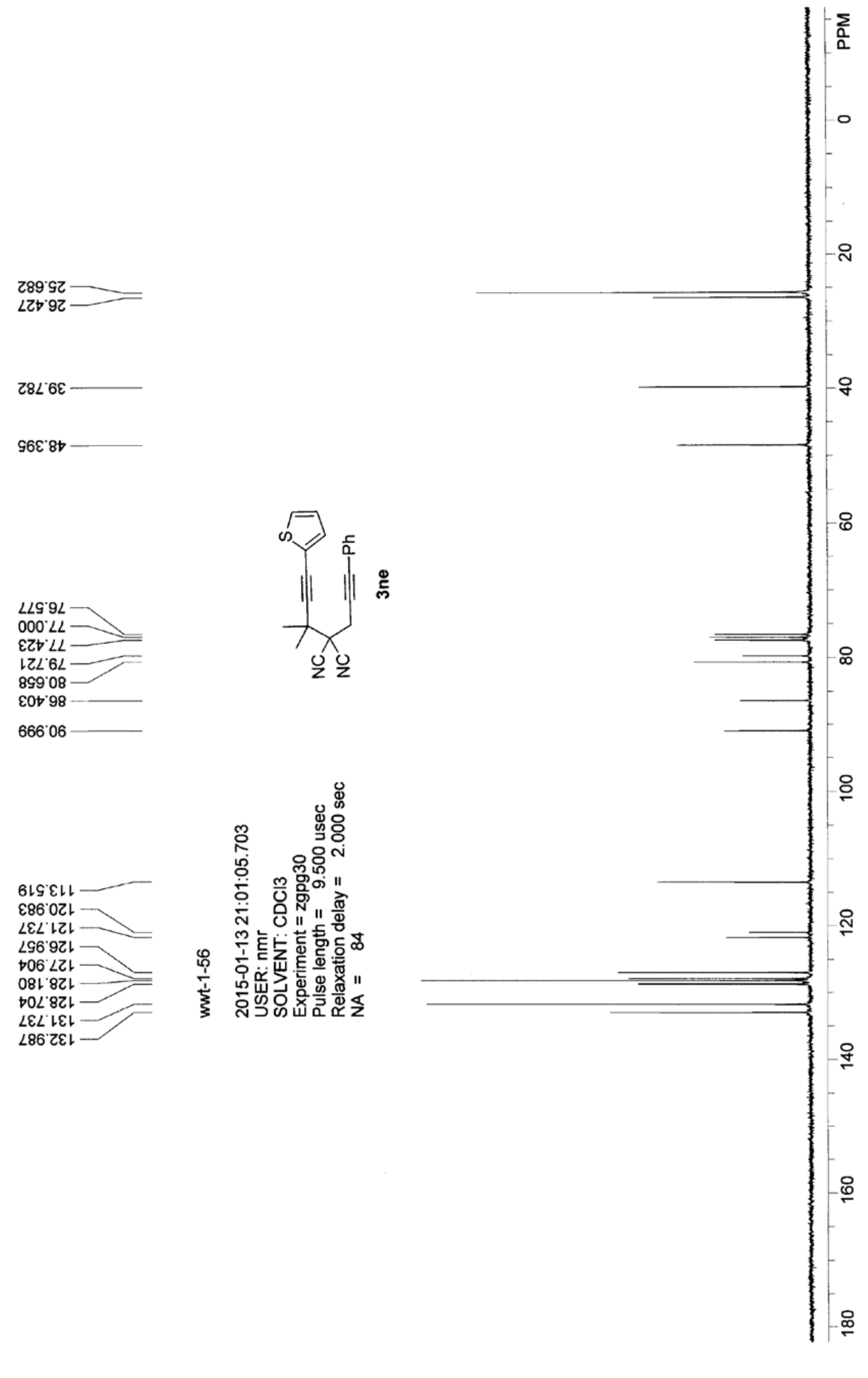

Supplementary Figure 61. ^{13}**C NMR (75 MHz, CDCl$_3$) spectrum for 3ne.**

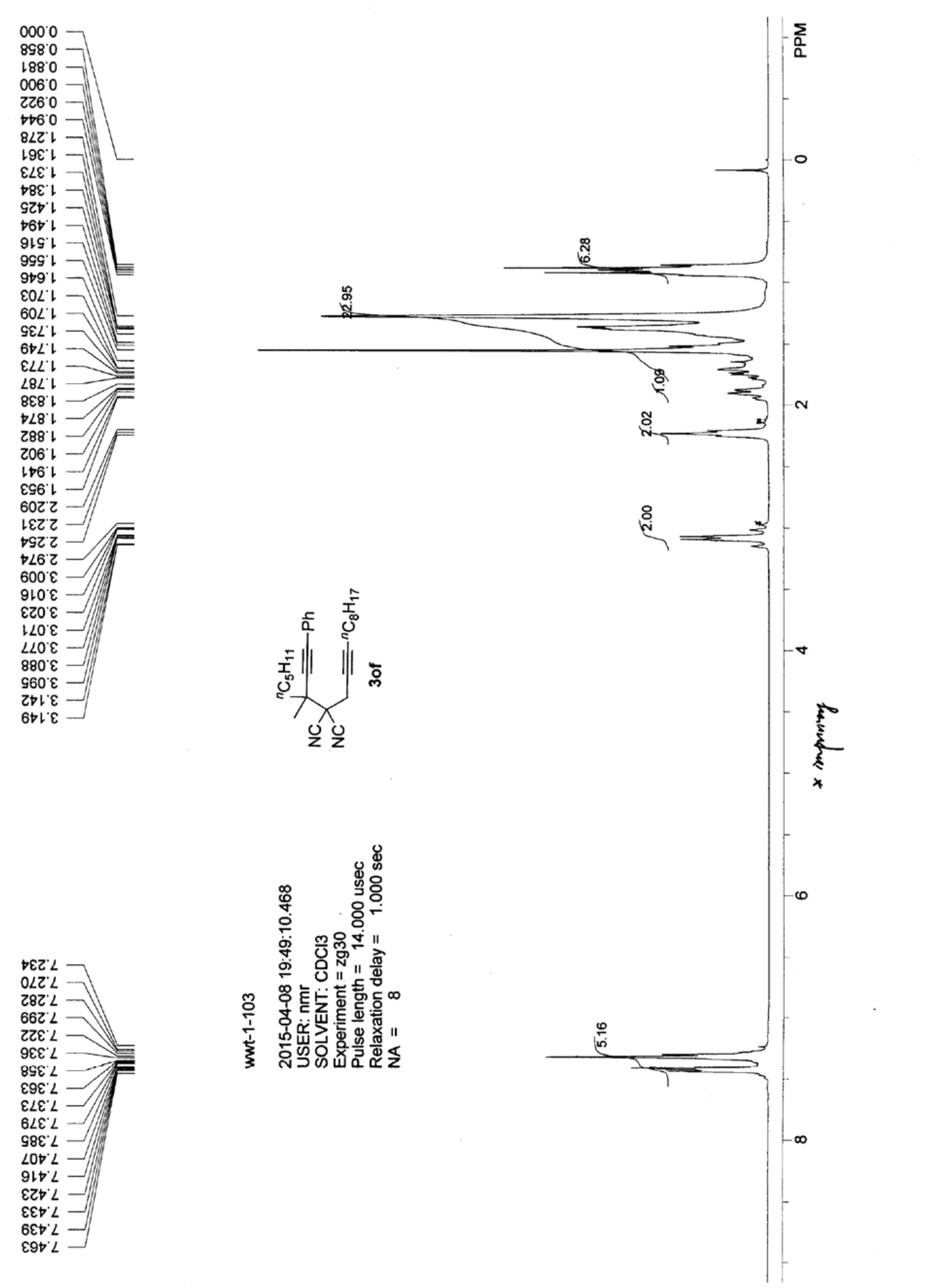

Supplementary Figure 62. ^{1}**H NMR (300 MHz,** $CDCl_3$**) spectrum for 3of.**

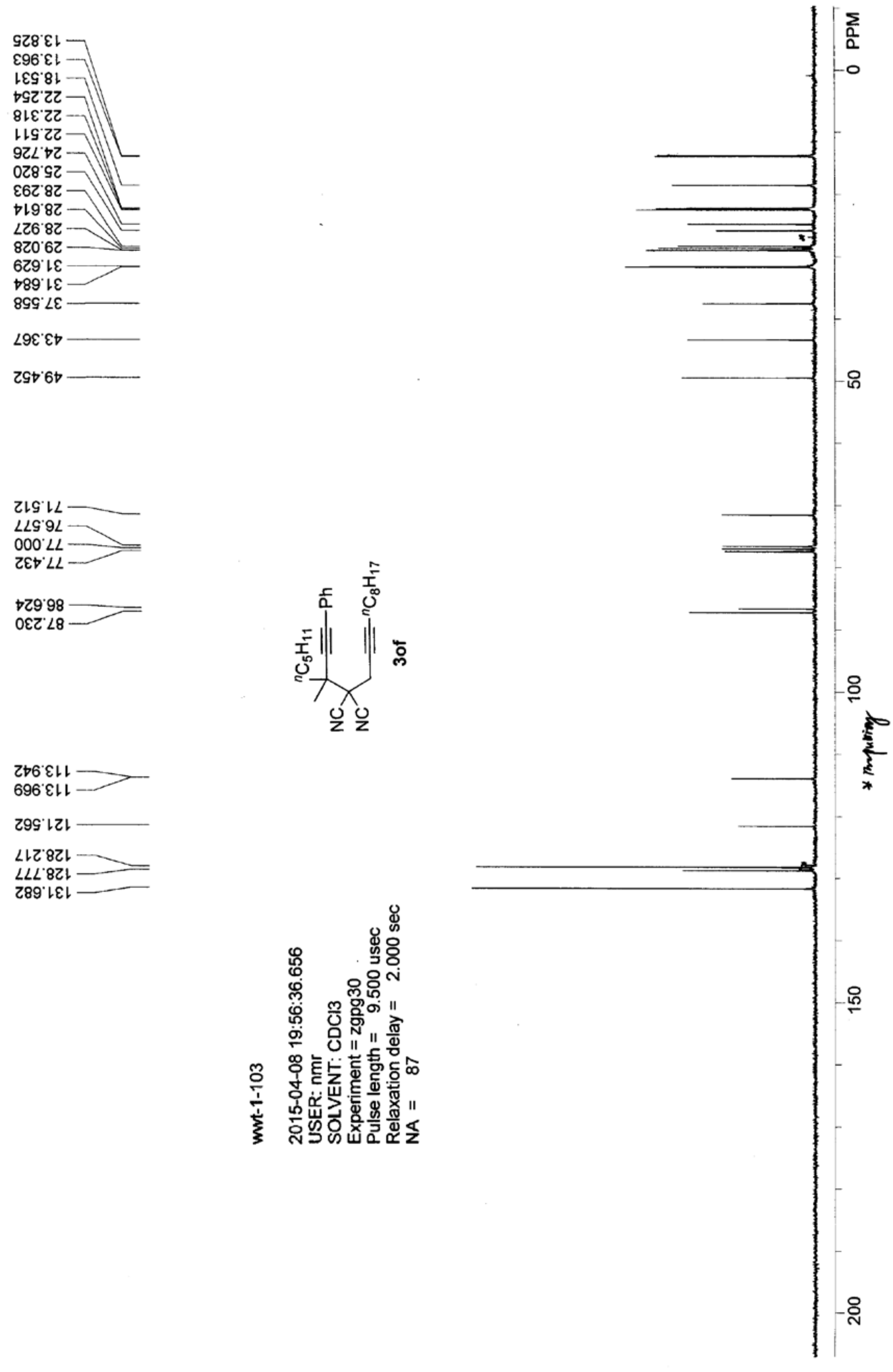

Supplementary Figure 63. ^{13}C NMR (75 MHz, $CDCl_3$) spectrum for 3of.

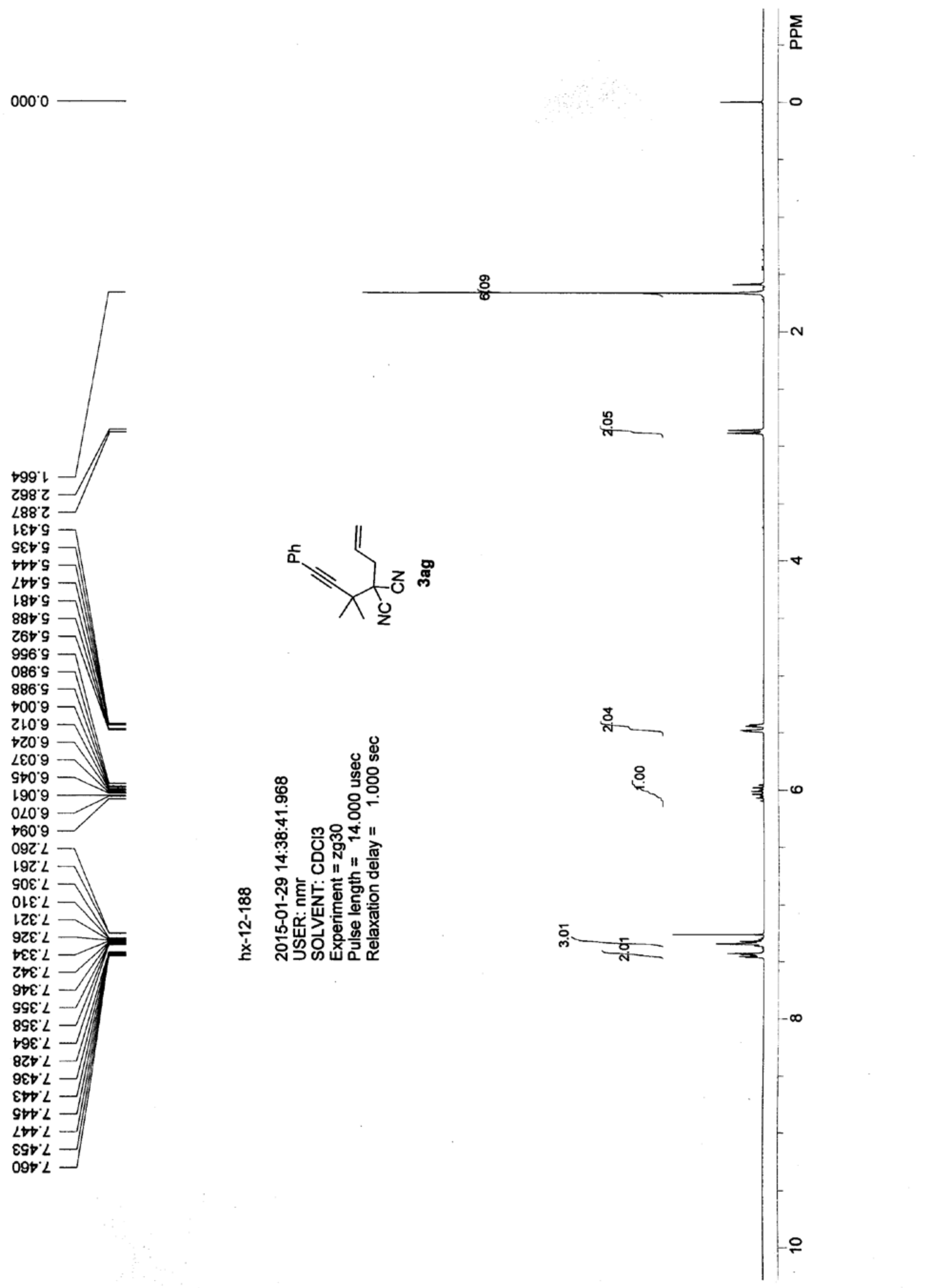

Supplementary Figure 64. ^{1}H NMR (300 MHz, $CDCl_3$) spectrum for 3ag.

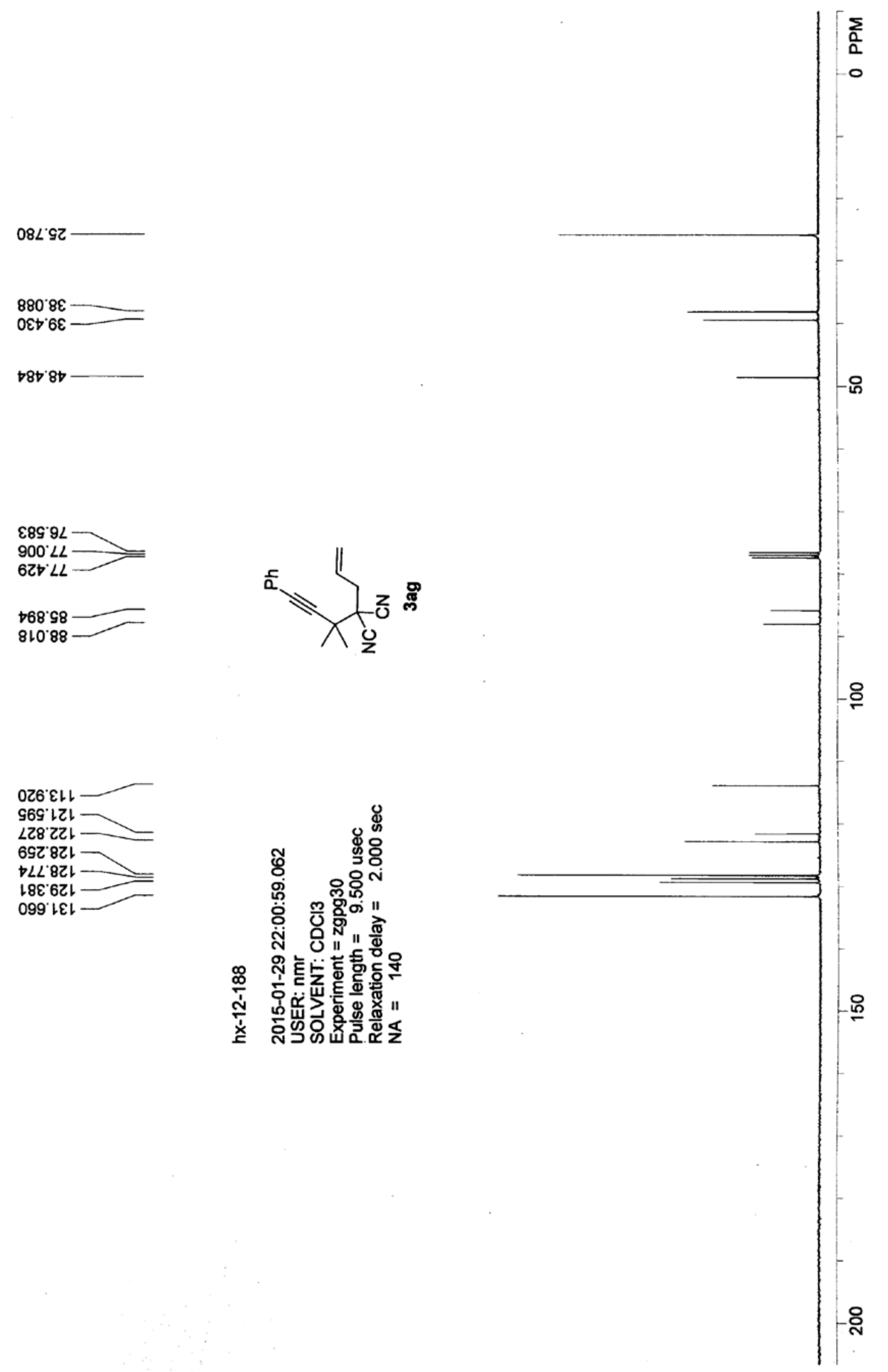

Supplementary Figure 65. ^{13}**C NMR (75 MHz,** $CDCl_3$**) spectrum for 3ag.**

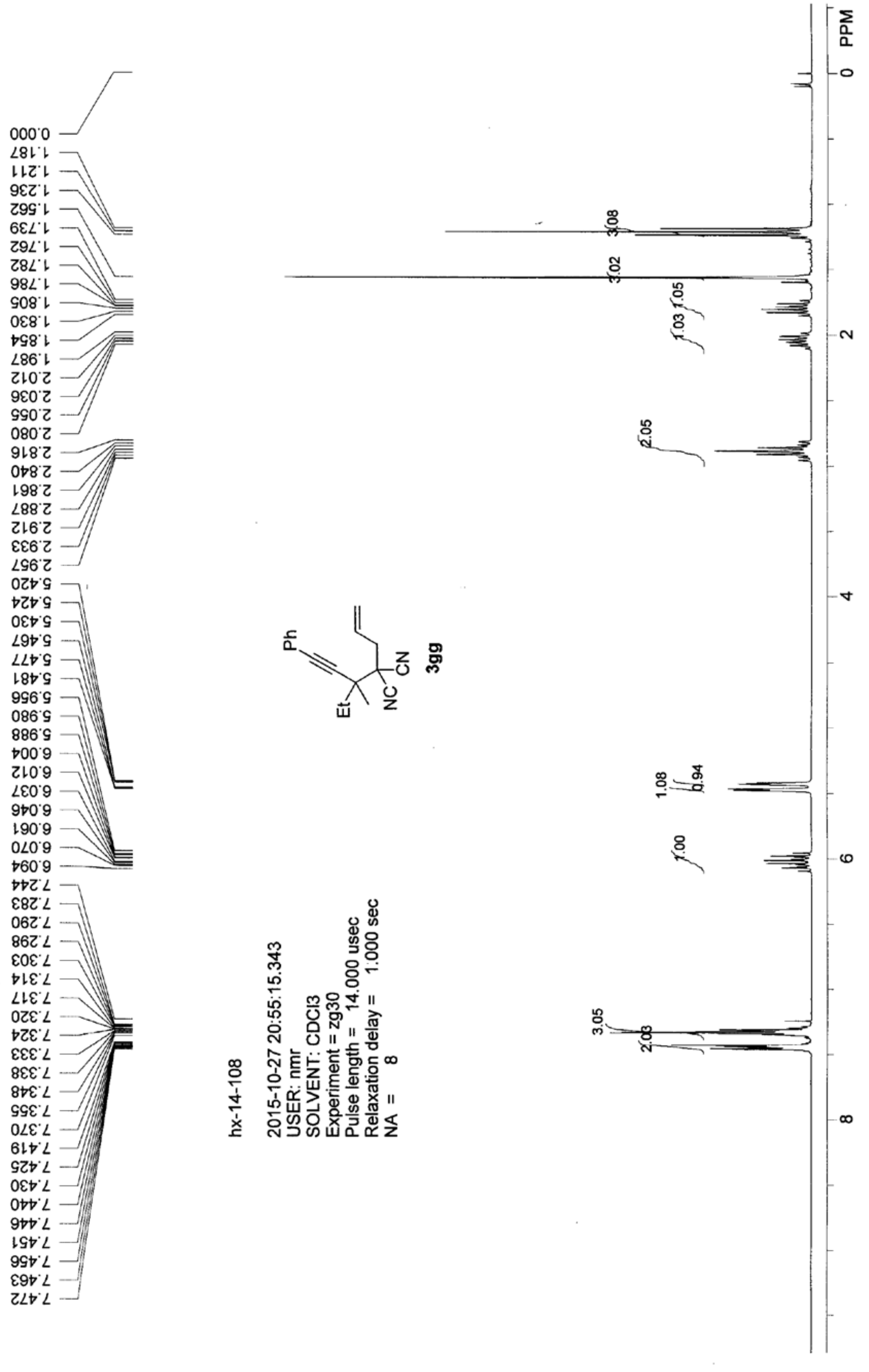

Supplementary Figure 66. **^{1}H NMR (300 MHz, $CDCl_3$) spectrum for 3gg.**

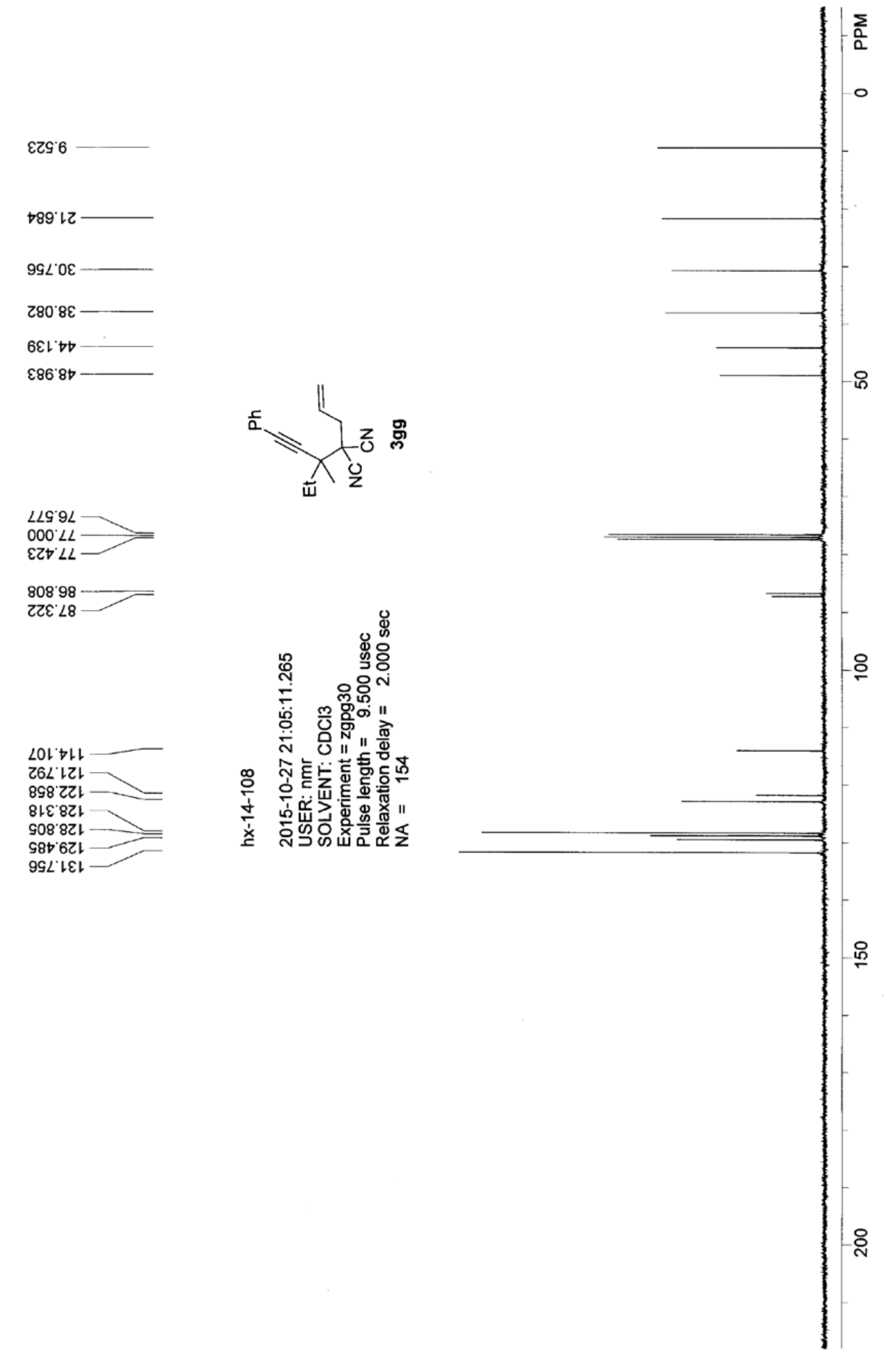

Supplementary Figure 67. ^{13}**C NMR (75 MHz,** $CDCl_3$**) spectrum for 3gg.**

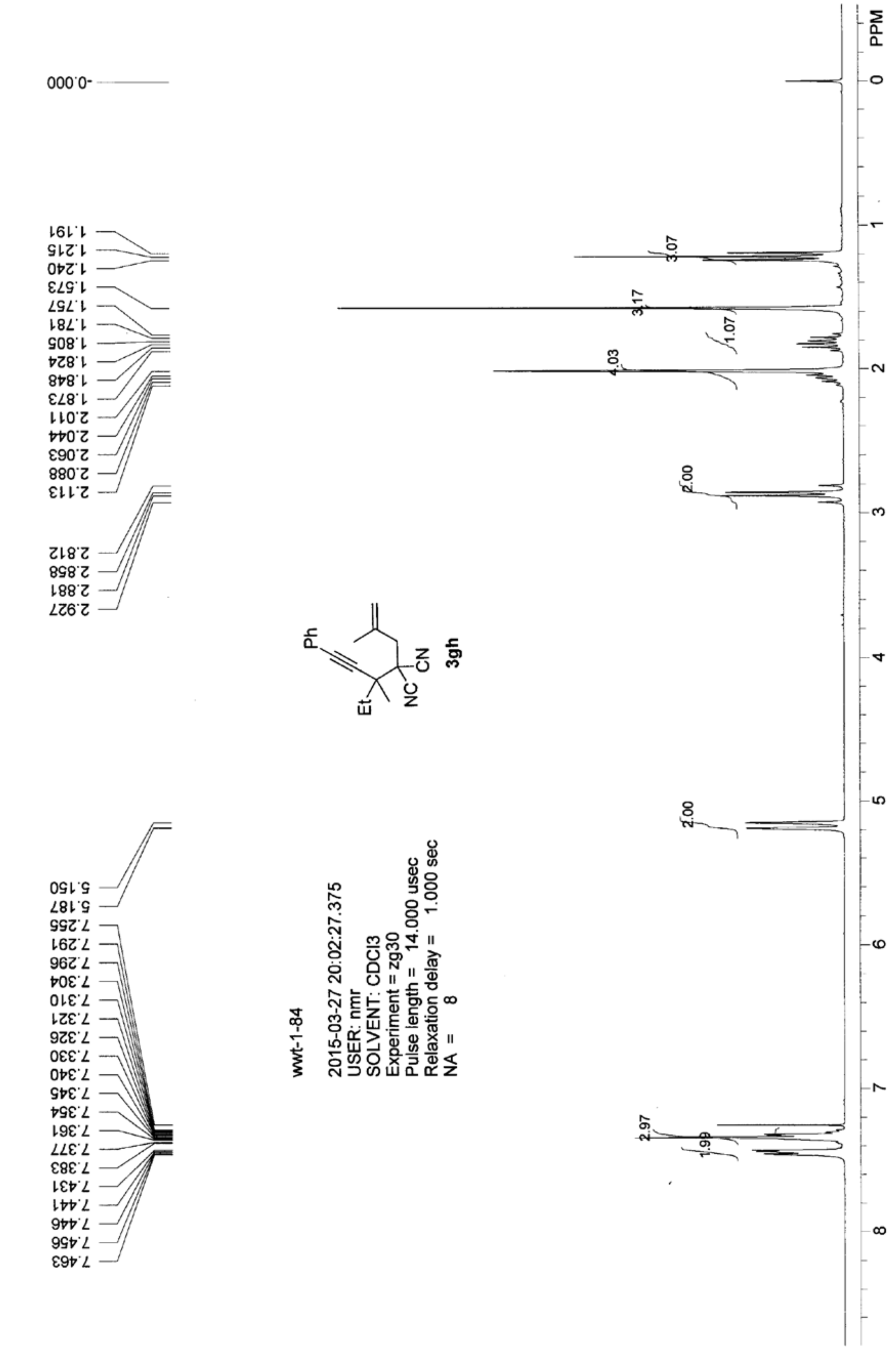

Supplementary Figure 68. ^{1}H NMR (300 MHz, $CDCl_3$) spectrum for 3gh.

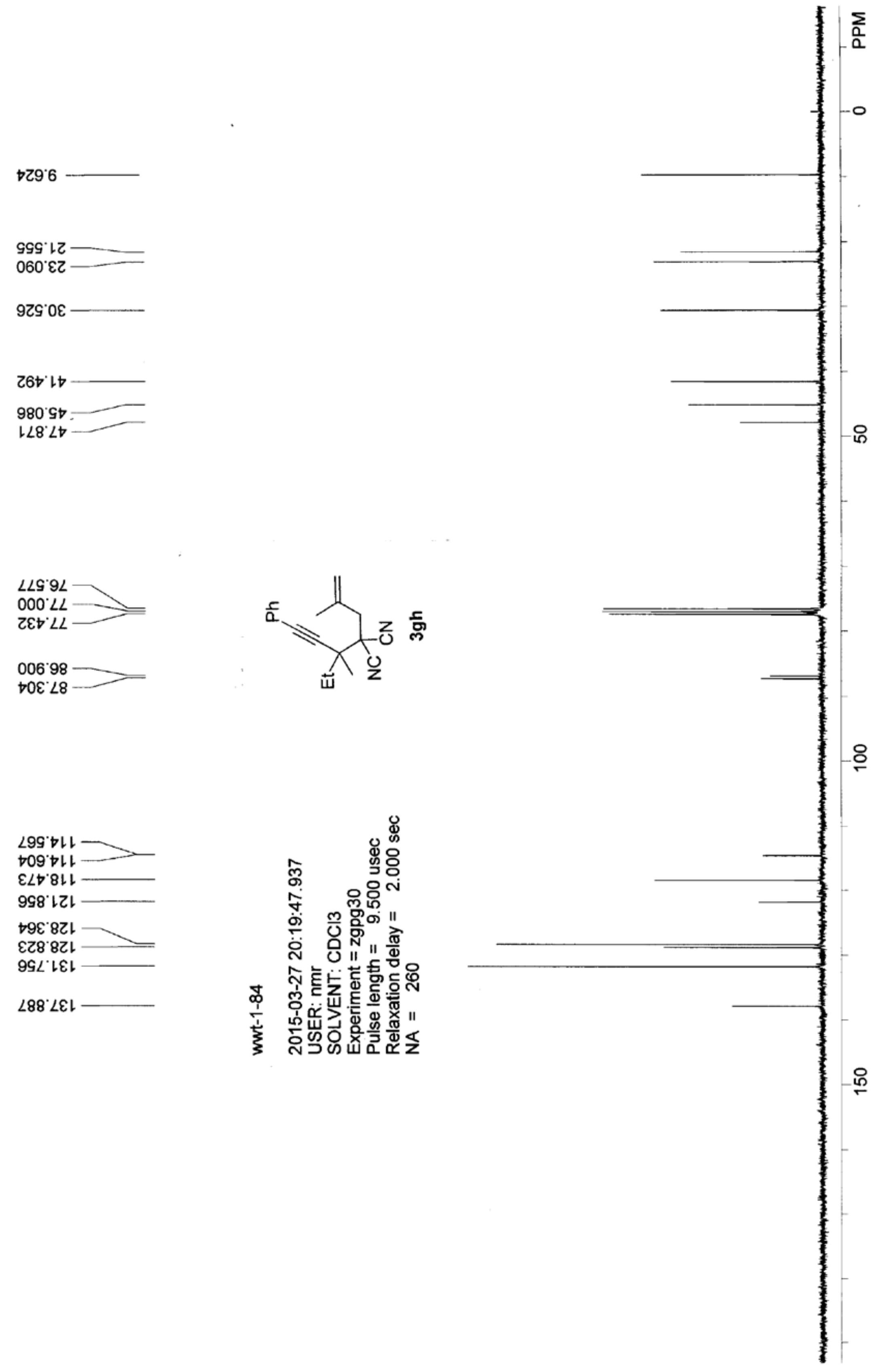

Supplementary Figure 69. ^{13}**C NMR (75 MHz,** $CDCl_3$**) spectrum for 3gh.**

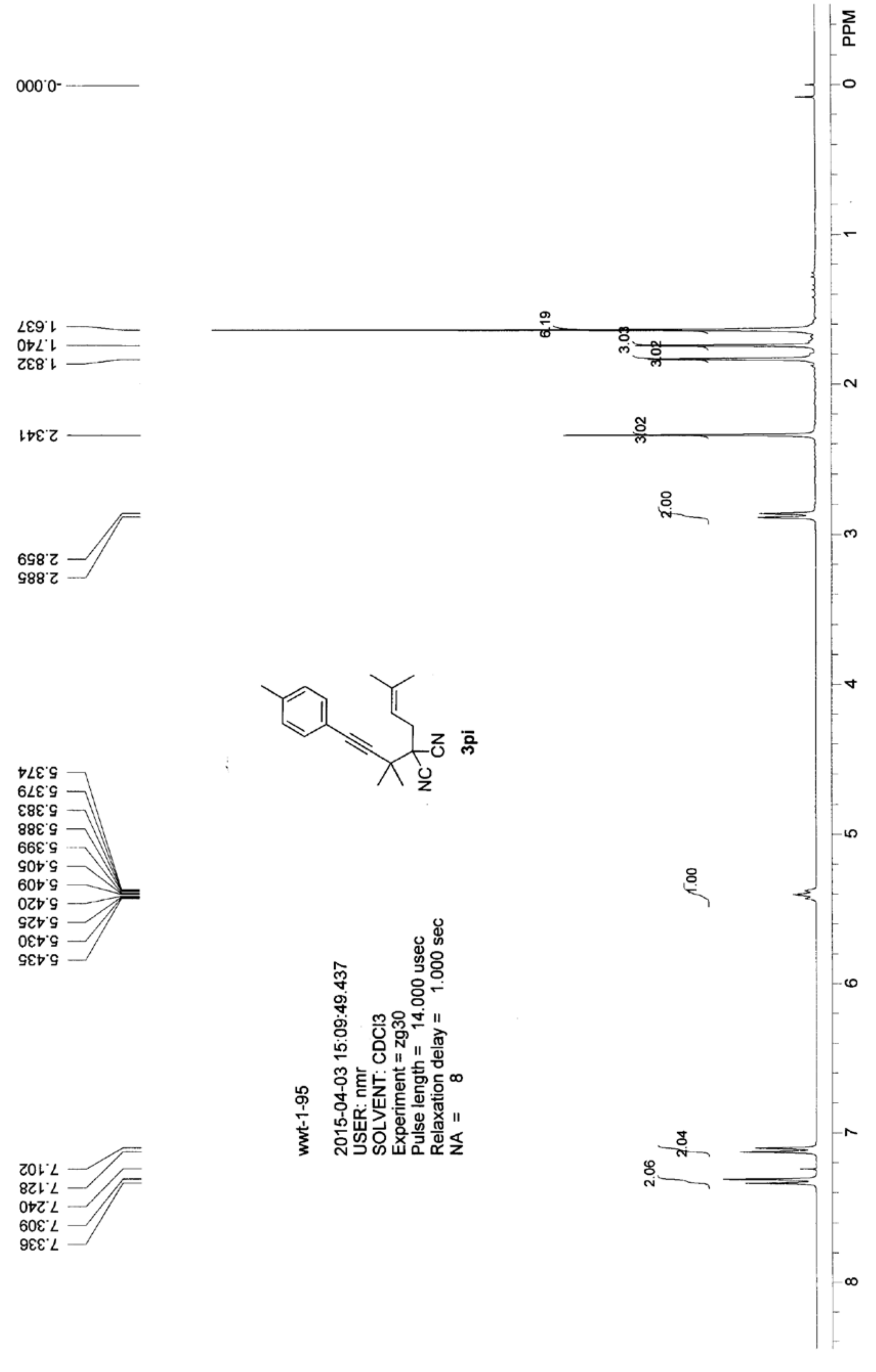

Supplementary Figure 70. **^{1}H NMR (300 MHz, $CDCl_3$) spectrum for 3pi.**

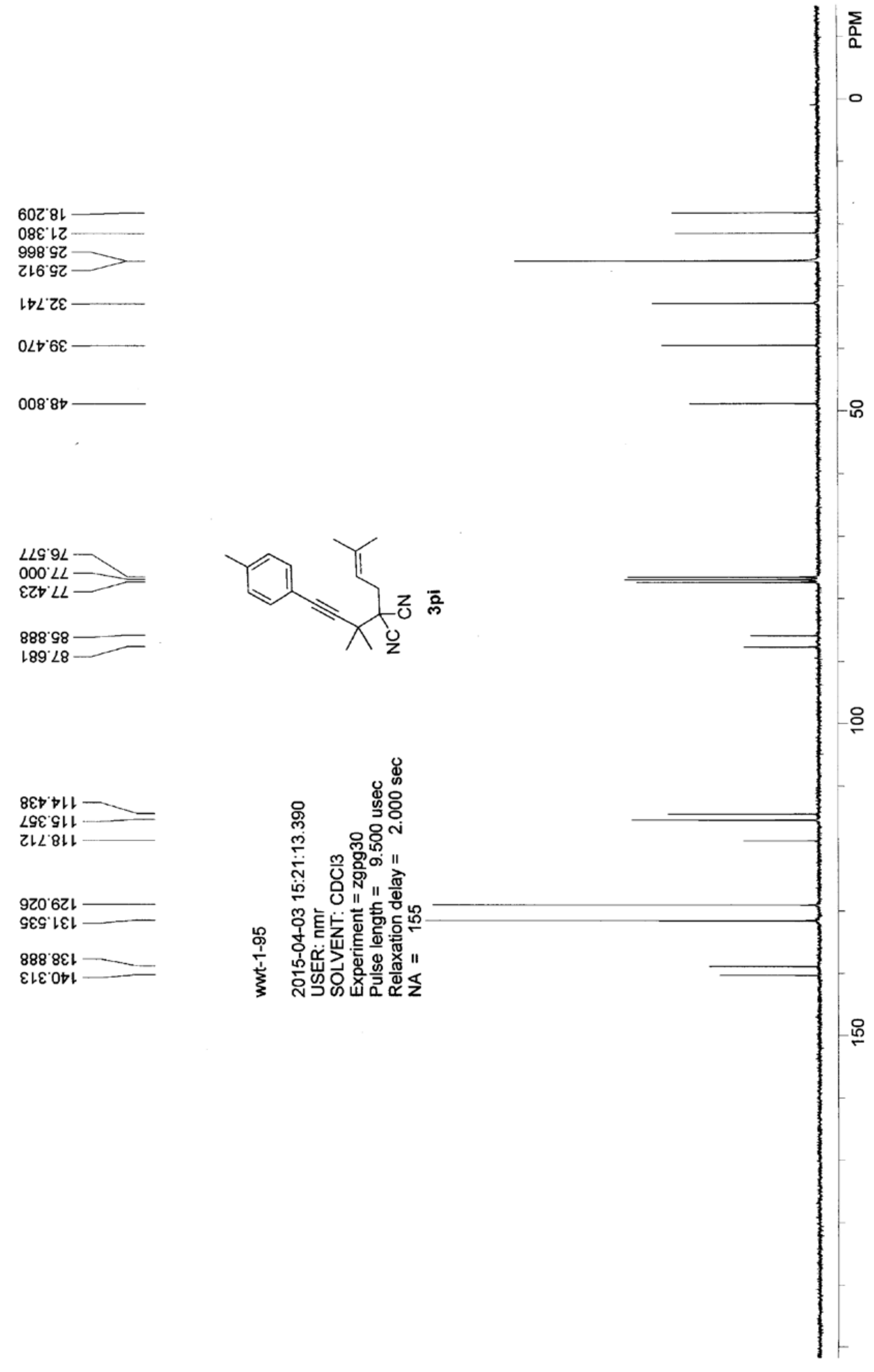

Supplementary Figure 71. **^{13}C NMR (75 MHz, $CDCl_3$) spectrum for 3pi.**

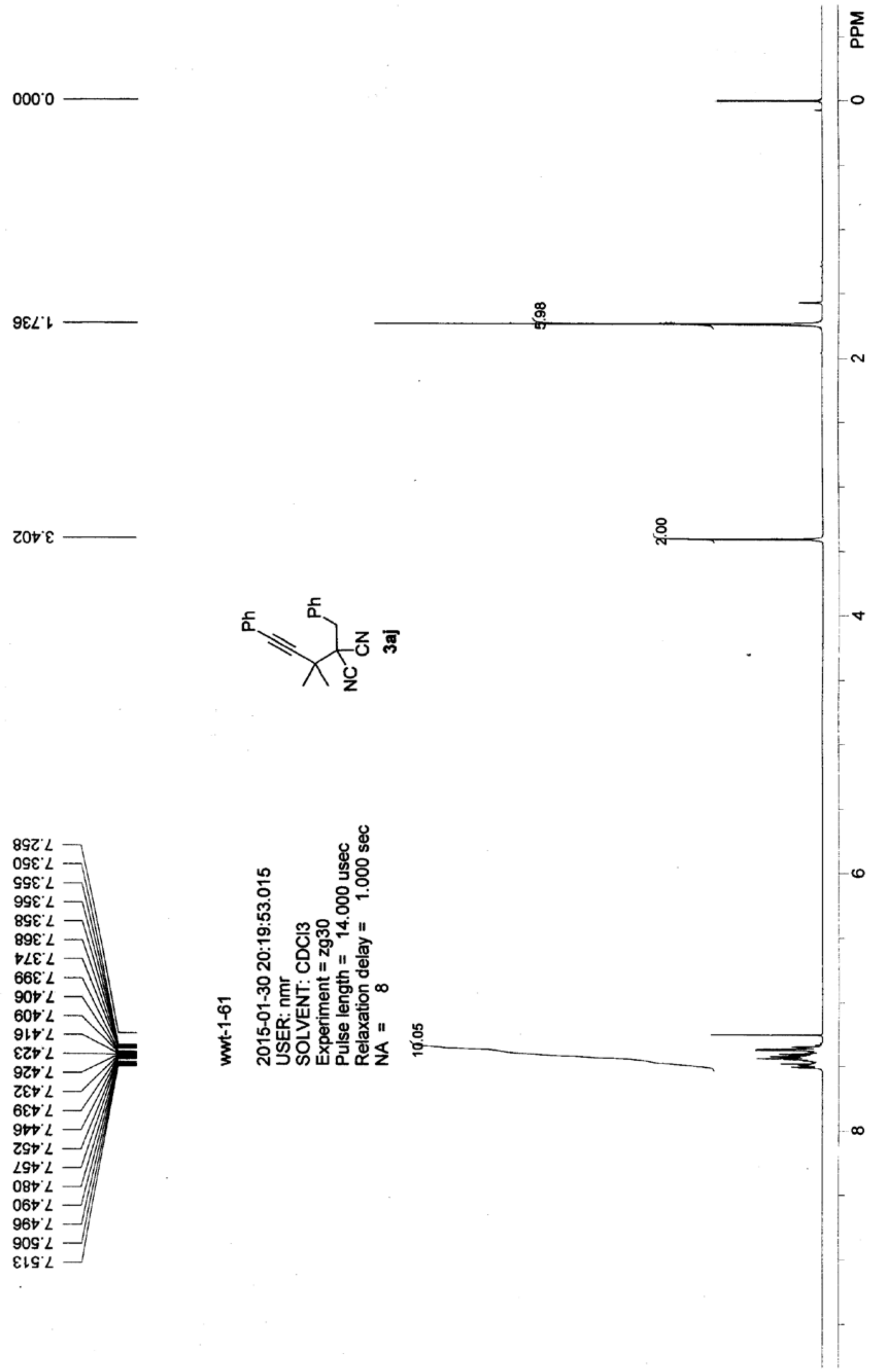

Supplementary Figure 72. ^{1}**H NMR (300 MHz,** $CDCl_3$**) spectrum for 3aj.**

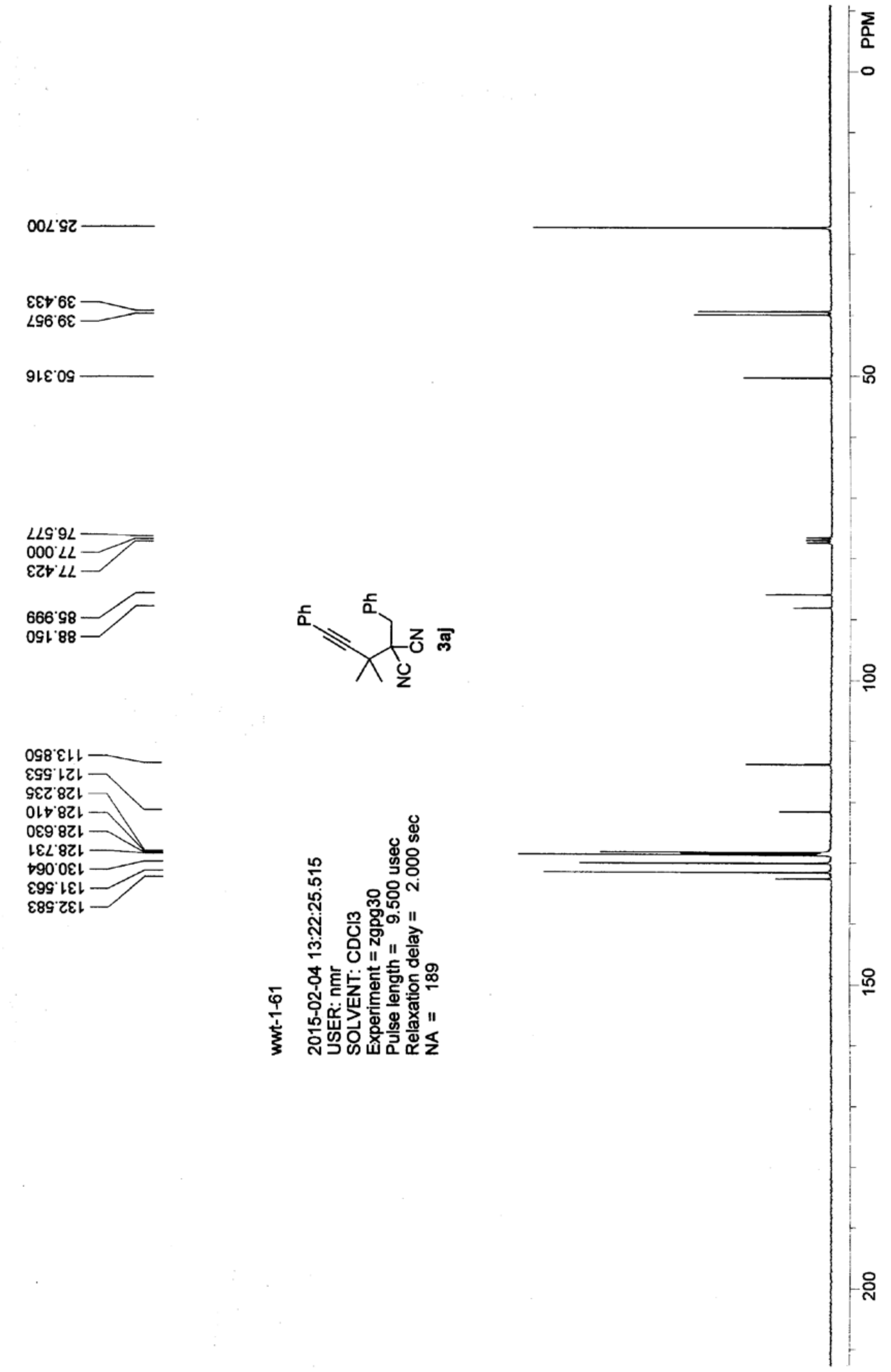

Supplementary Figure 73. ^{13}**C NMR (75 MHz, CDCl**$_3$**) spectrum for 3aj.**

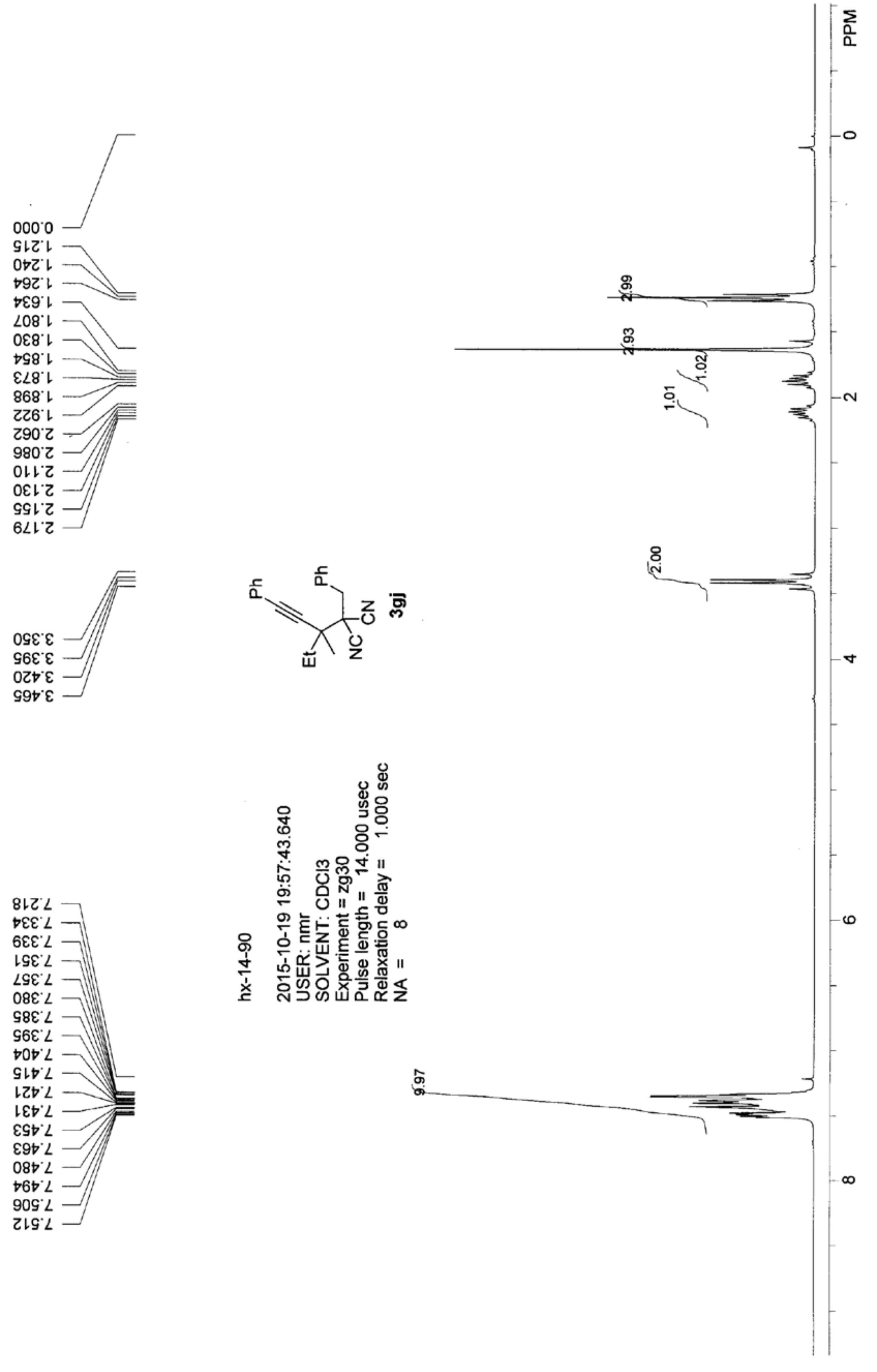

Supplementary Figure 74. ^{1}H NMR (300 MHz, $CDCl_3$) spectrum for 3gj.

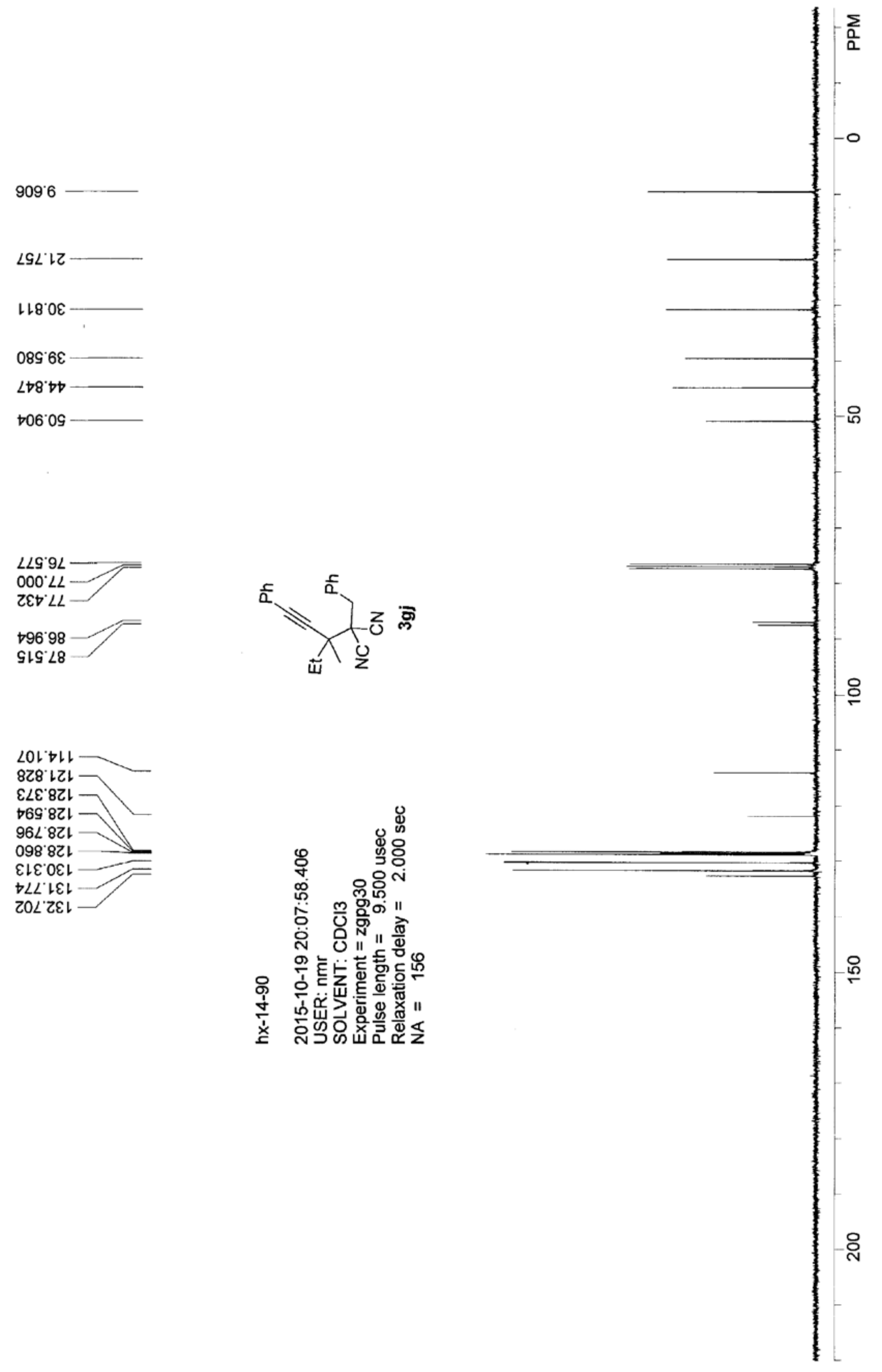

Supplementary Figure 75. ^{13}**C NMR (75 MHz,** $CDCl_3$**) spectrum for 3gj.**

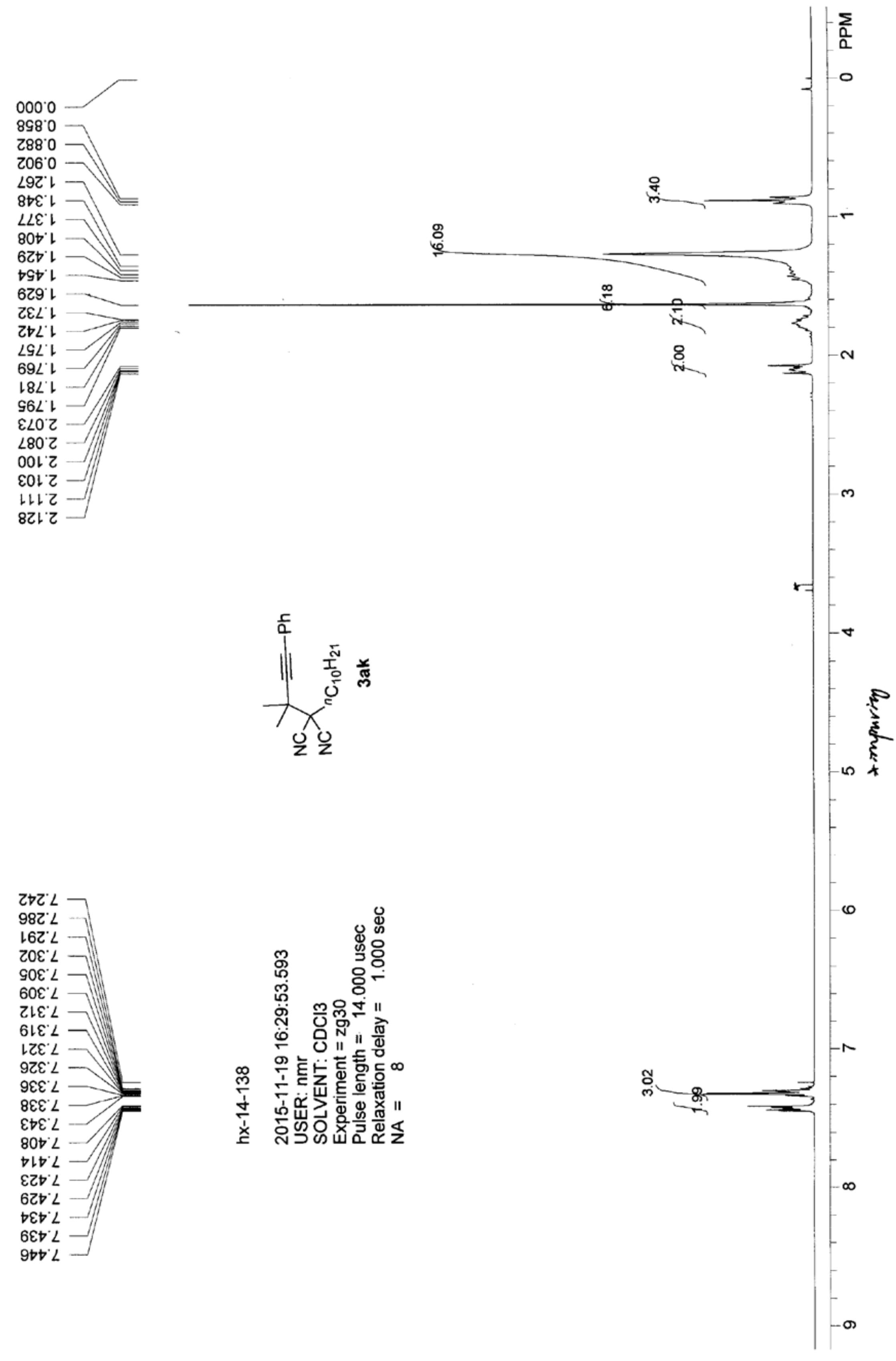

Supplementary Figure 76. **^{1}H NMR (300 MHz, $CDCl_3$) spectrum for 3ak.**

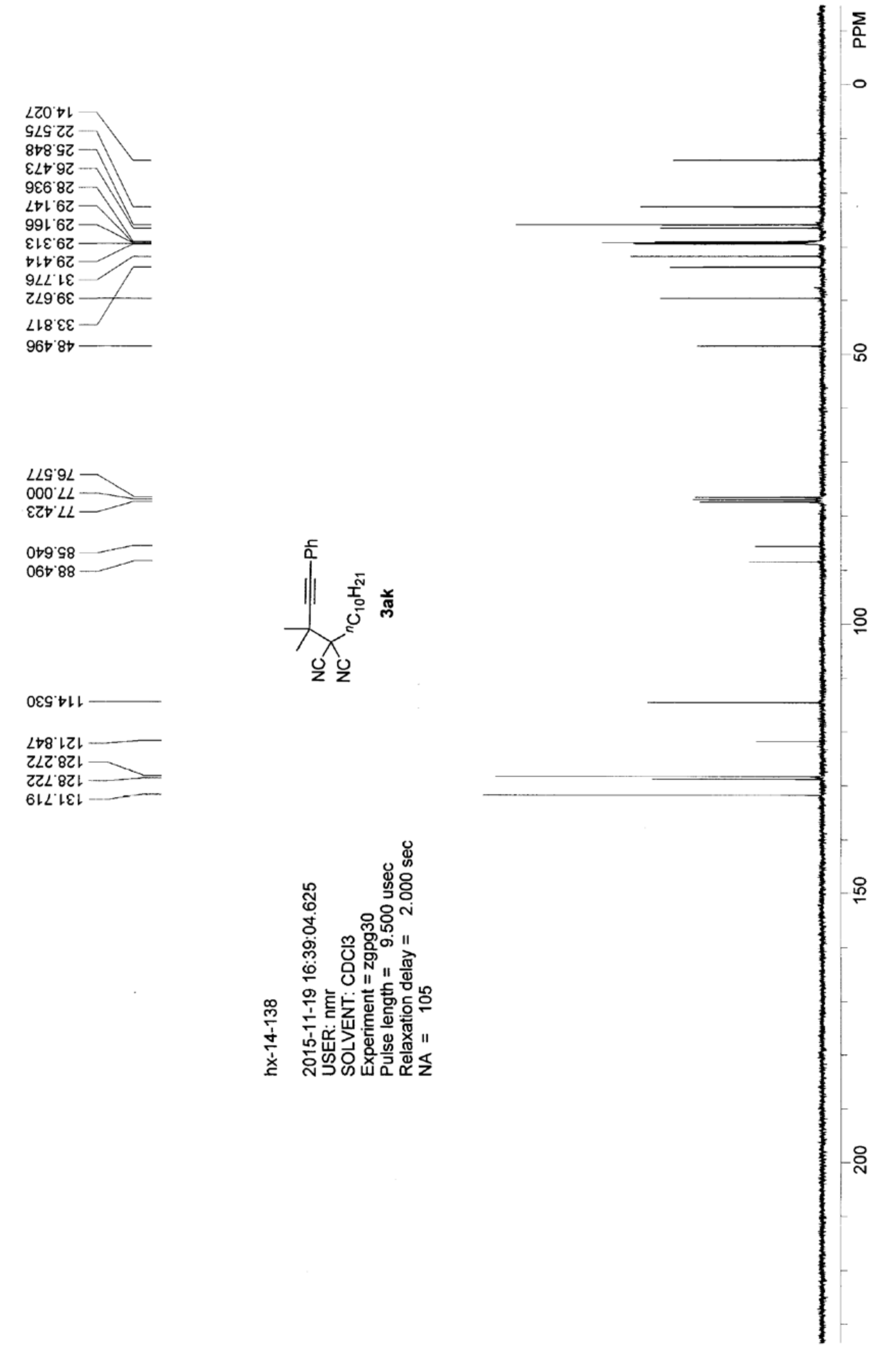

Supplementary Figure 77. ^{13}**C NMR (75 MHz,** $CDCl_3$**) spectrum for 3ak.**

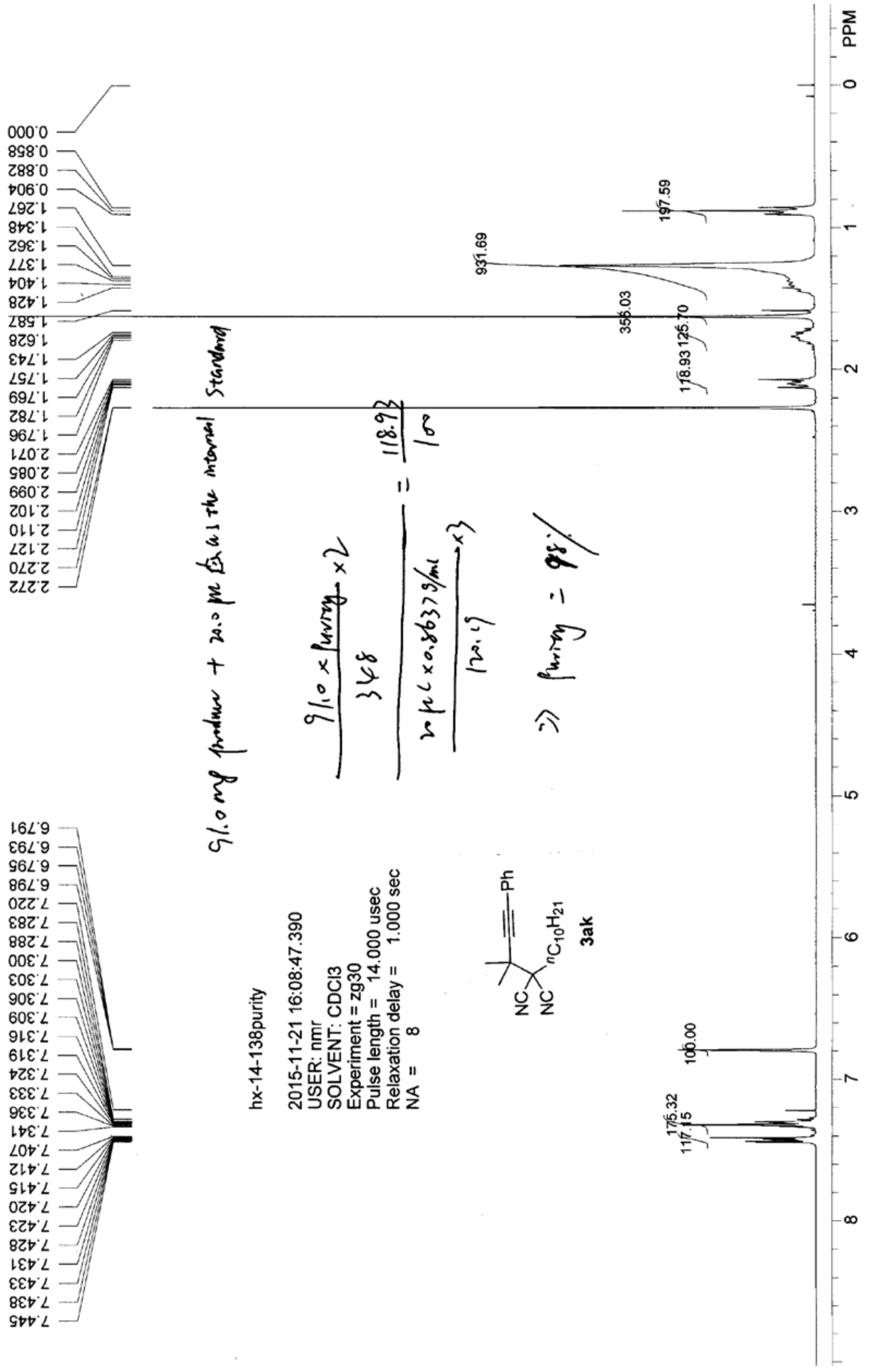

Supplementary Figure 78. **^{1}H NMR (300 MHz, $CDCl_3$) spectrum for the purity of 3ak.**

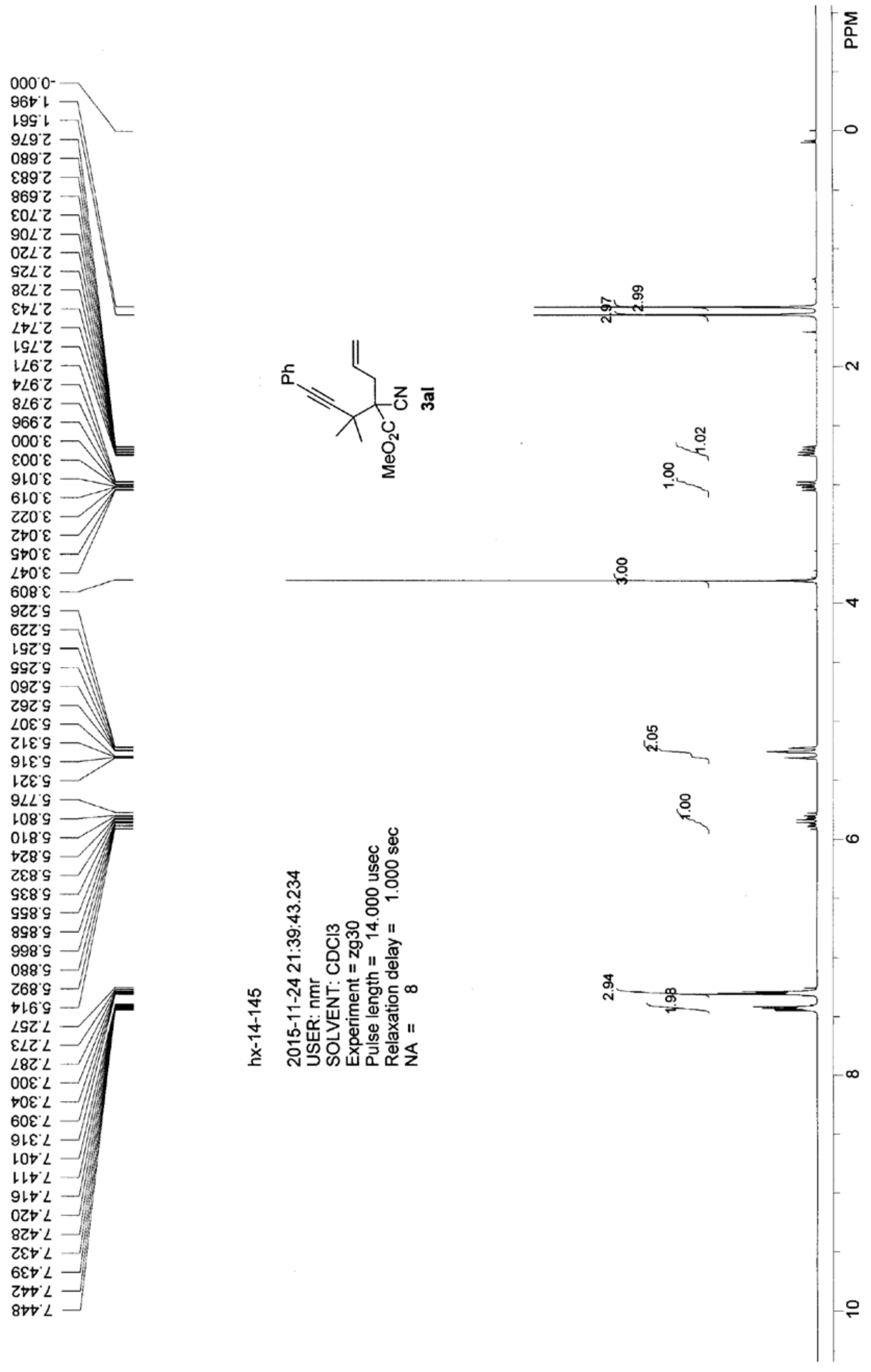

Supplementary Figure 79. ^{1}**H NMR (300 MHz, CDCl**$_3$**) spectrum for 3al.**

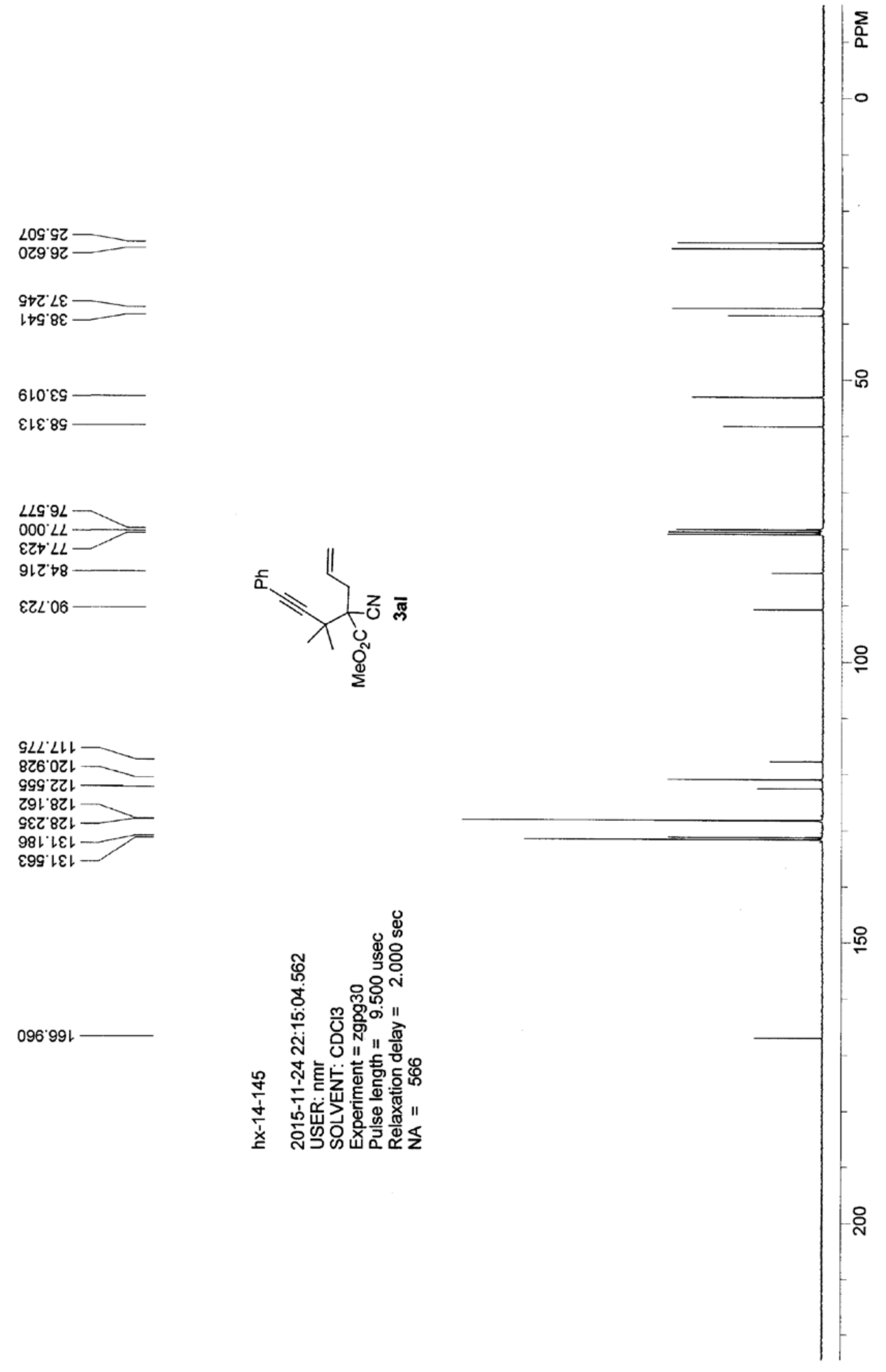

Supplementary Figure 80. **^{13}C NMR (75 MHz, $CDCl_3$) spectrum for 3al.**

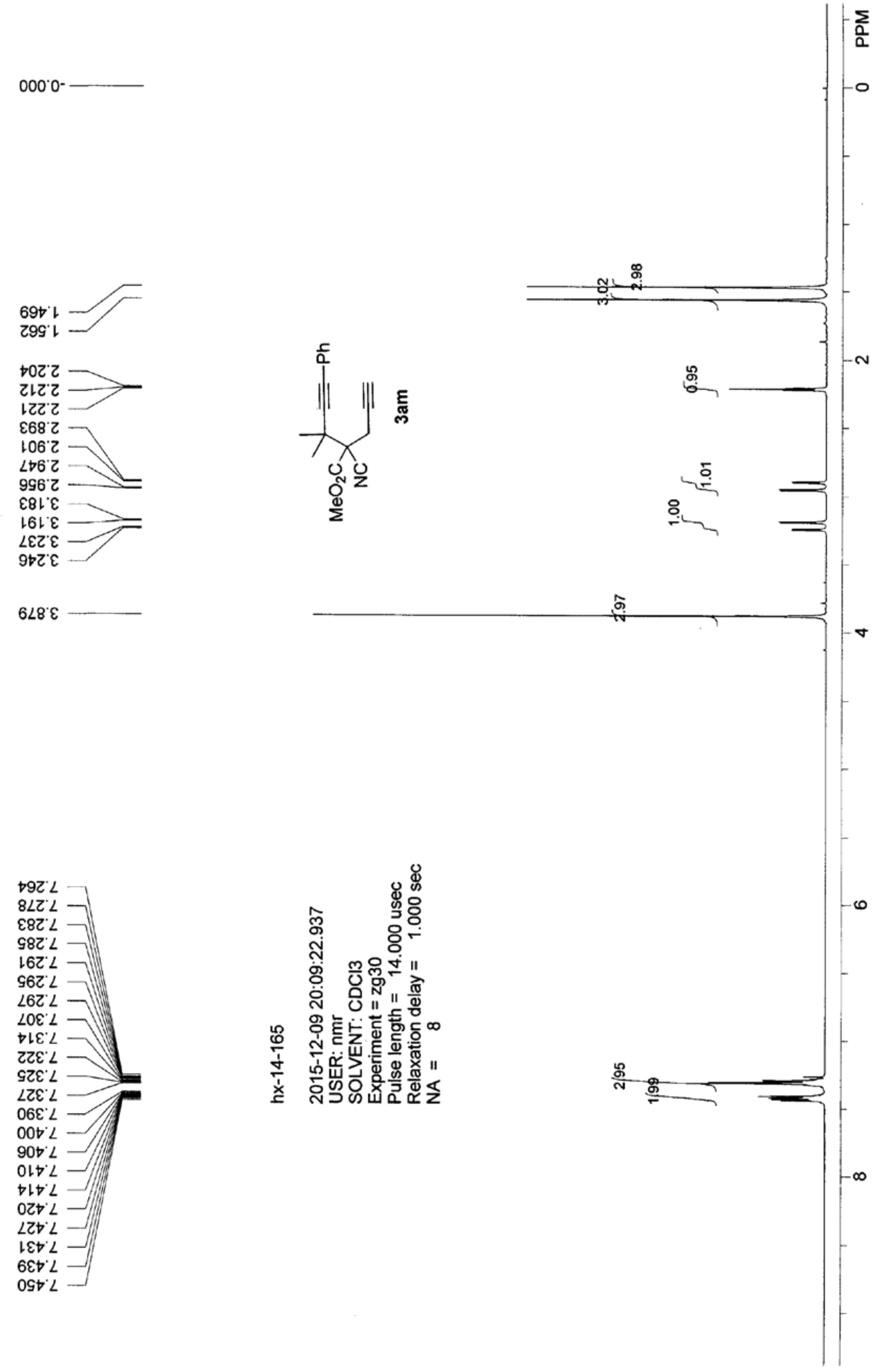

Supplementary Figure 81. ^{1}H NMR (300 MHz, $CDCl_3$) spectrum for 3am.

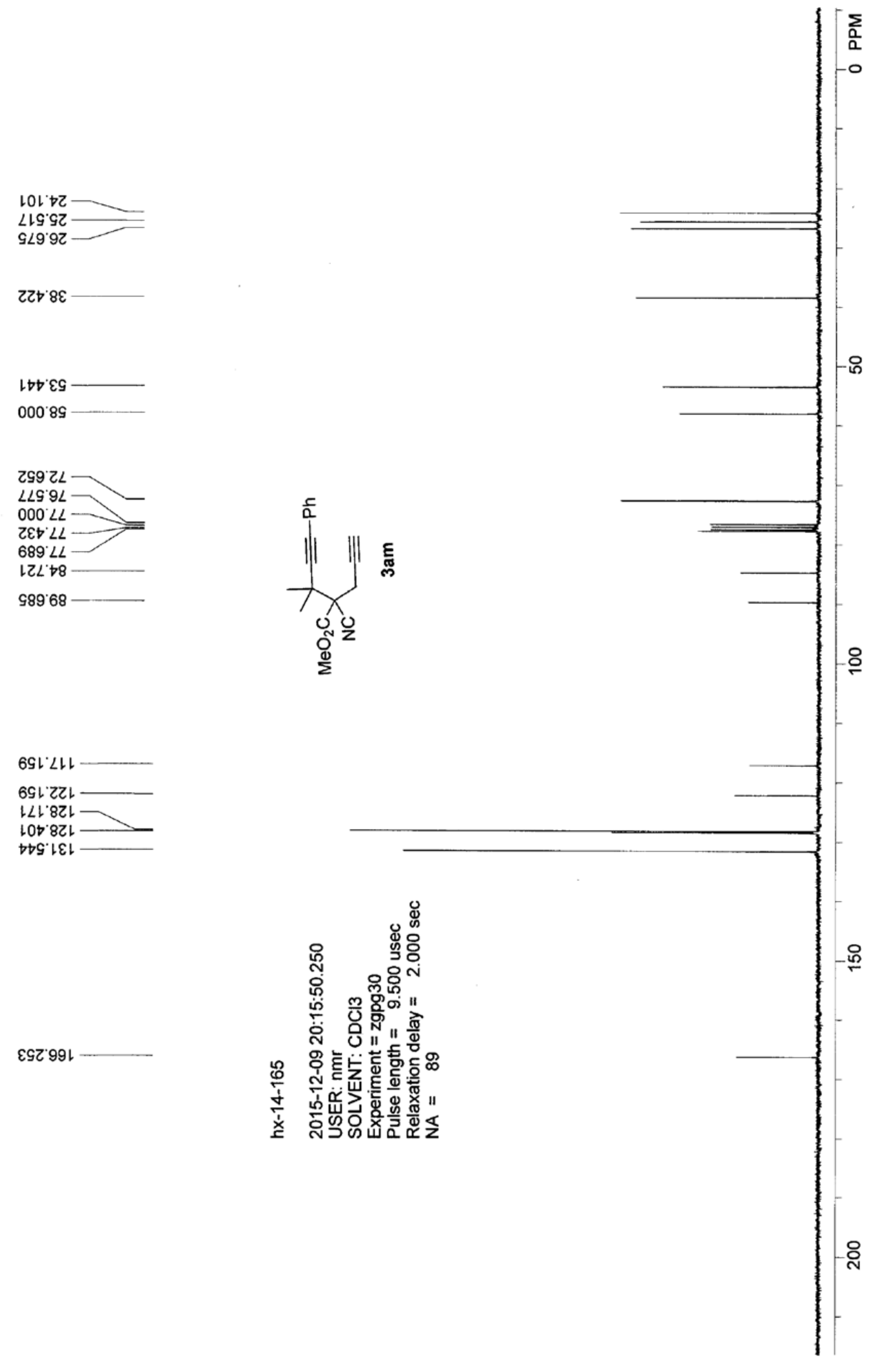

Supplementary Figure 82. **^{13}C NMR (75 MHz, $CDCl_3$) spectrum for 3am.**

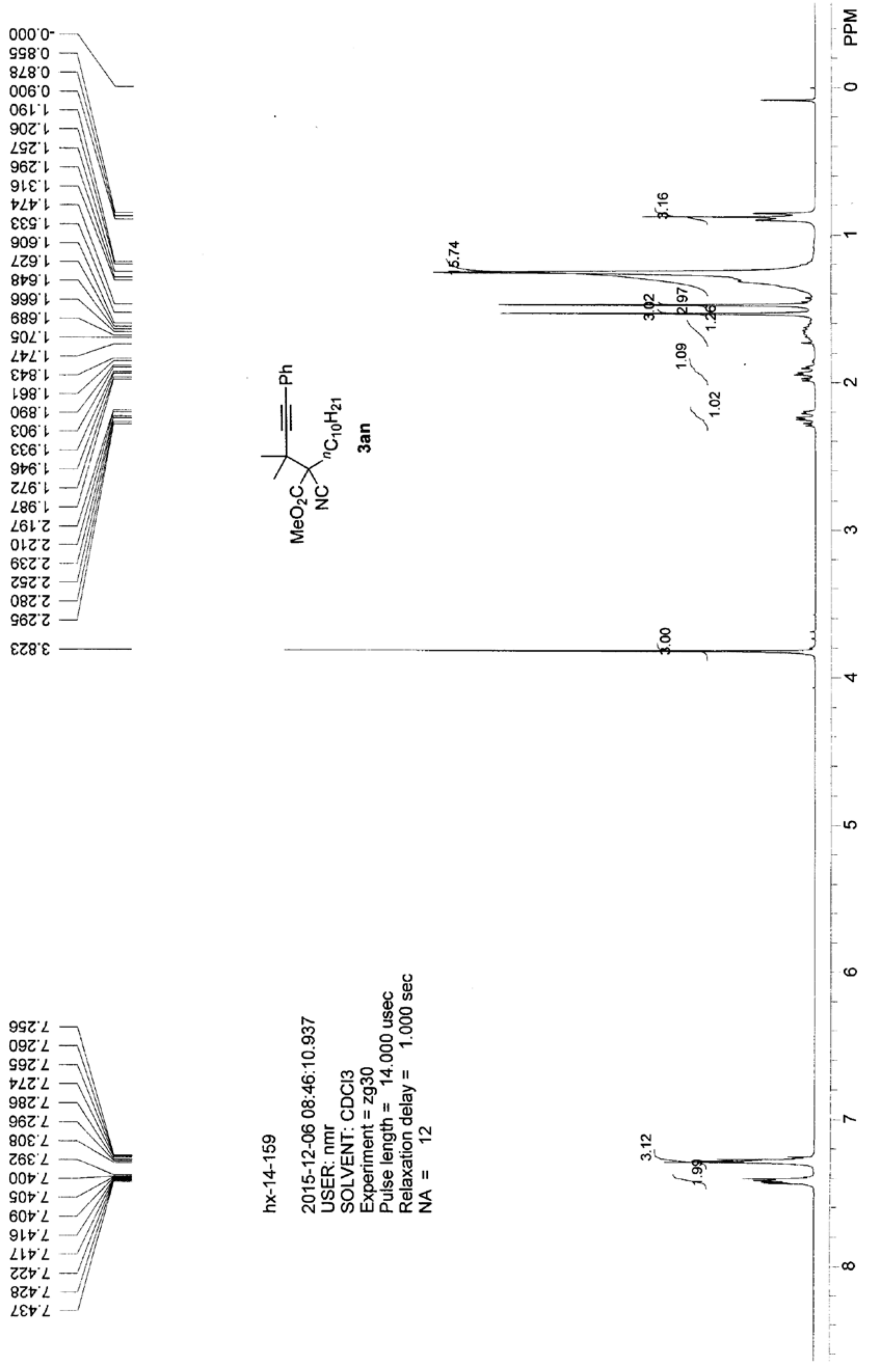

Supplementary Figure 83. **^{1}H NMR (300 MHz, $CDCl_3$) spectrum for 3an.**

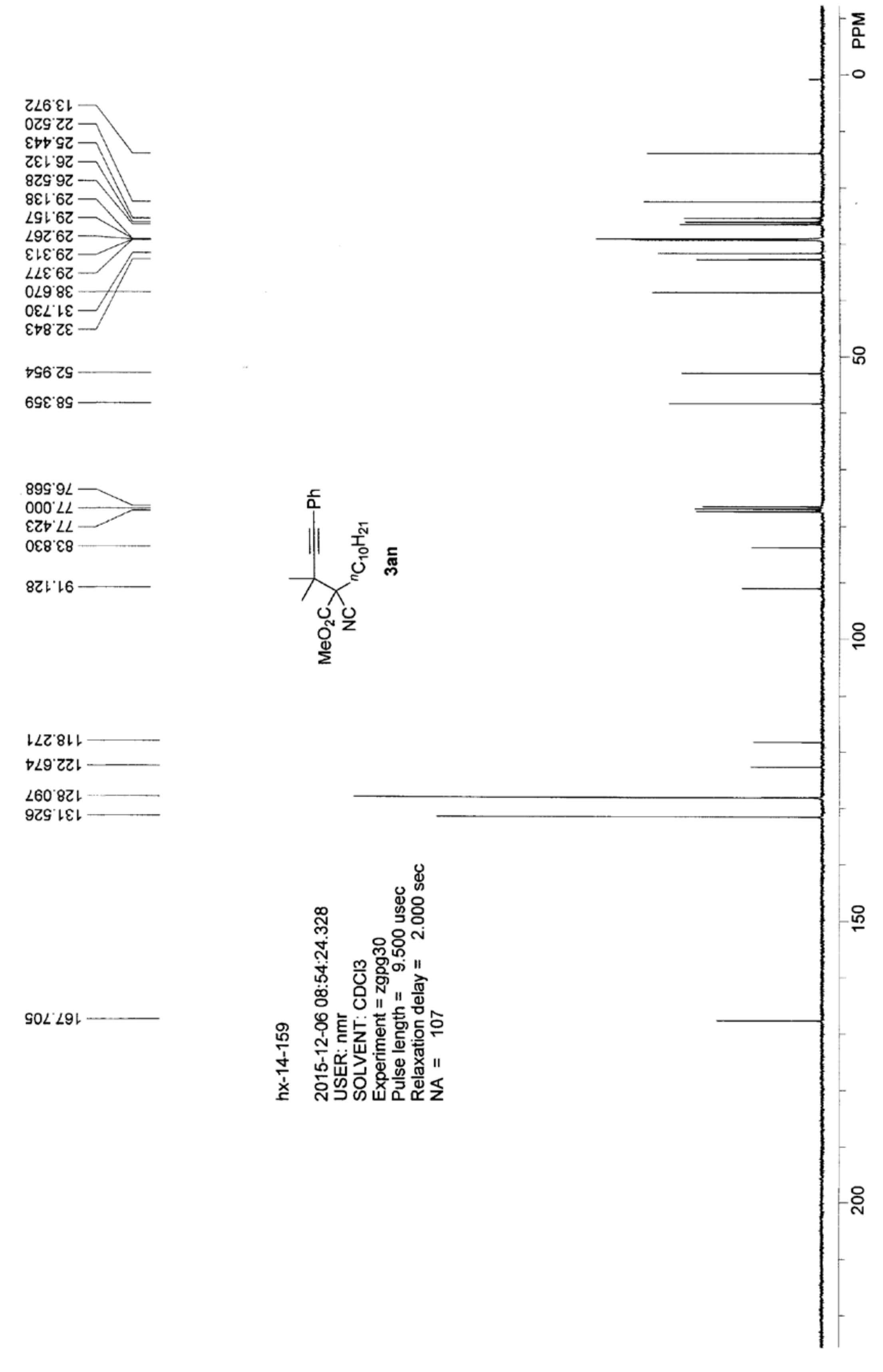

Supplementary Figure 84. ^{13}C NMR (75 MHz, $CDCl_3$) spectrum for 3an.

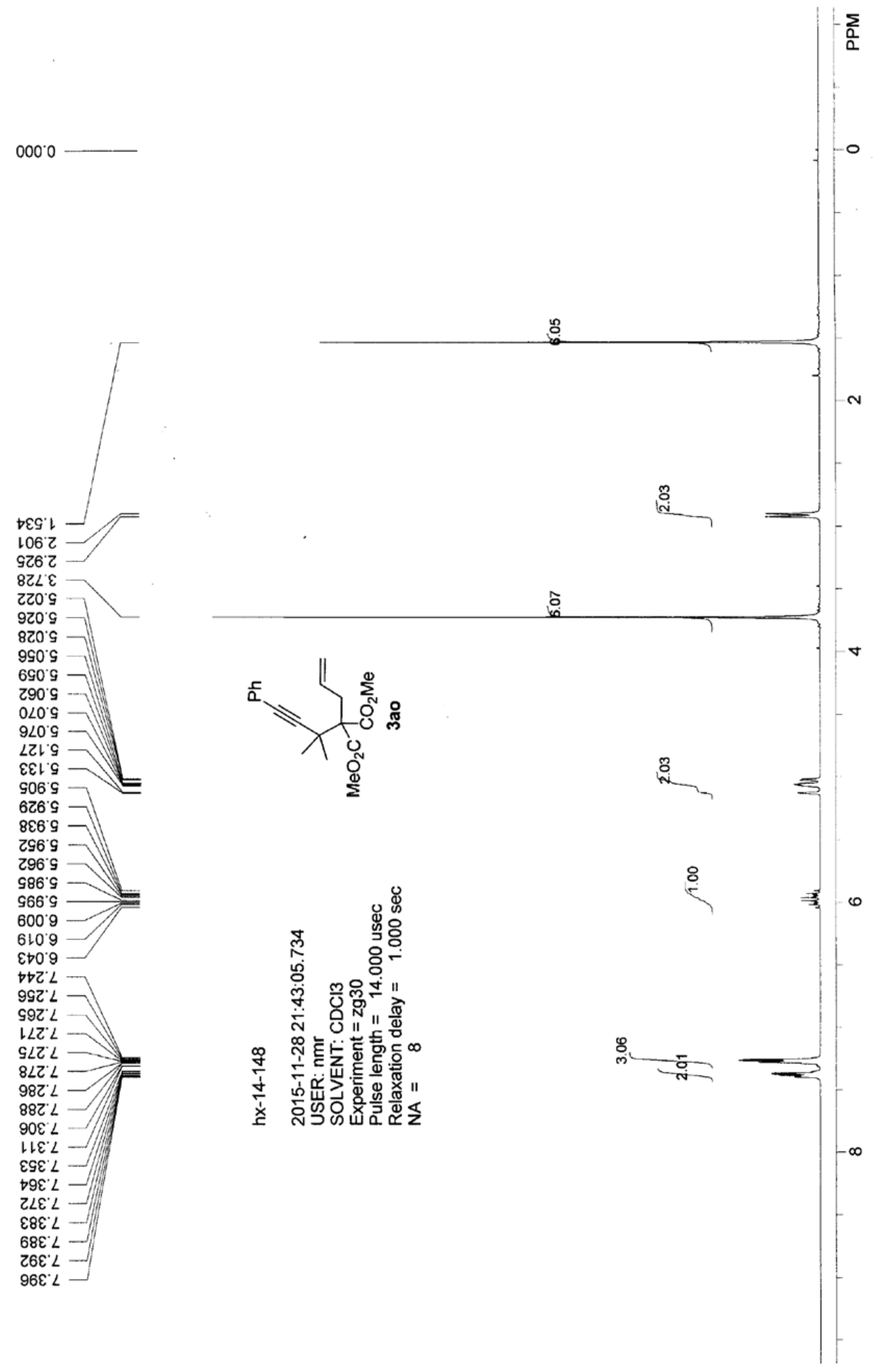

Supplementary Figure 85. ^{1}H NMR (300 MHz, $CDCl_3$) spectrum for 3ao.

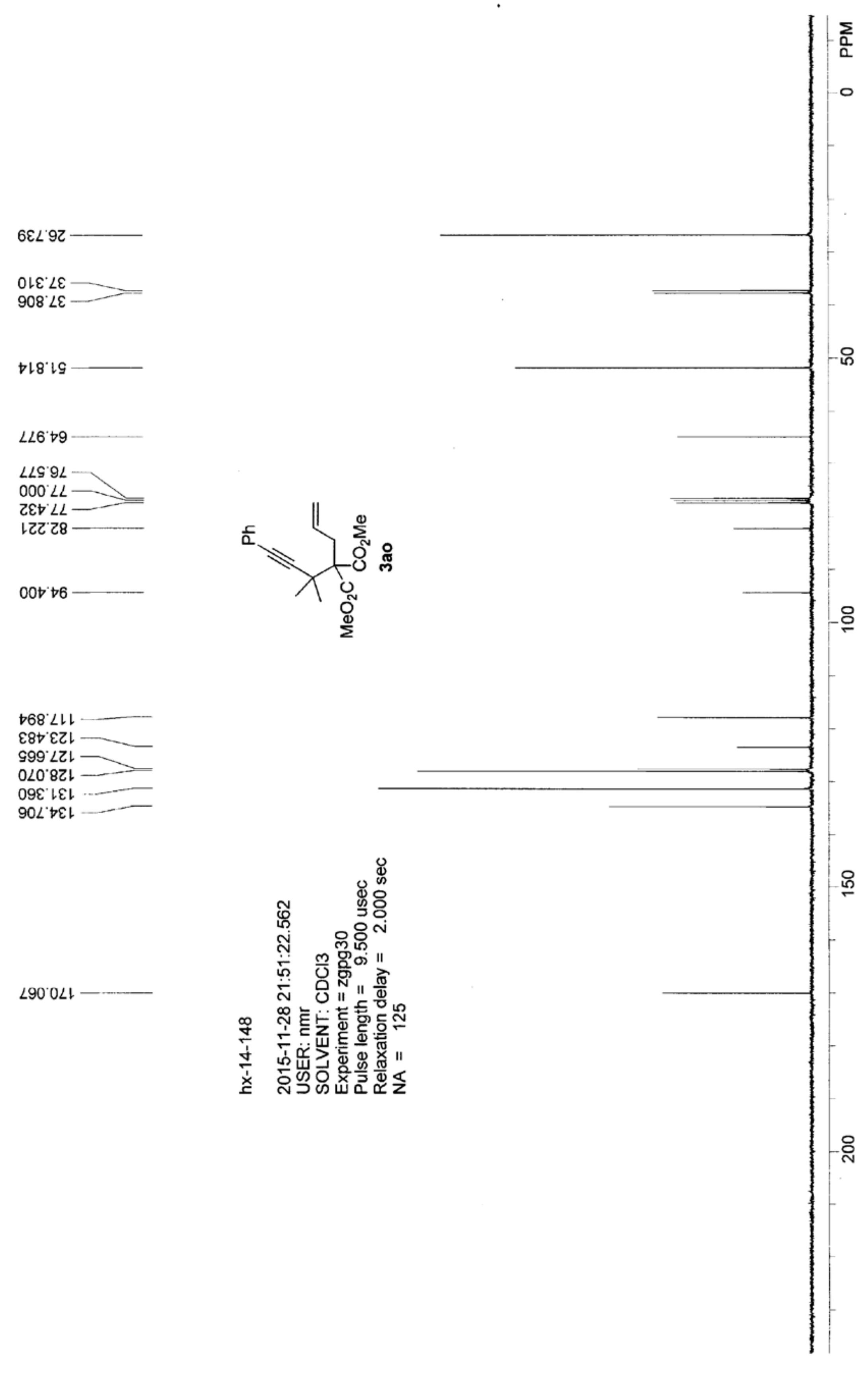

Supplementary Figure 86. **^{13}C NMR (75 MHz, $CDCl_3$) spectrum for 3ao.**

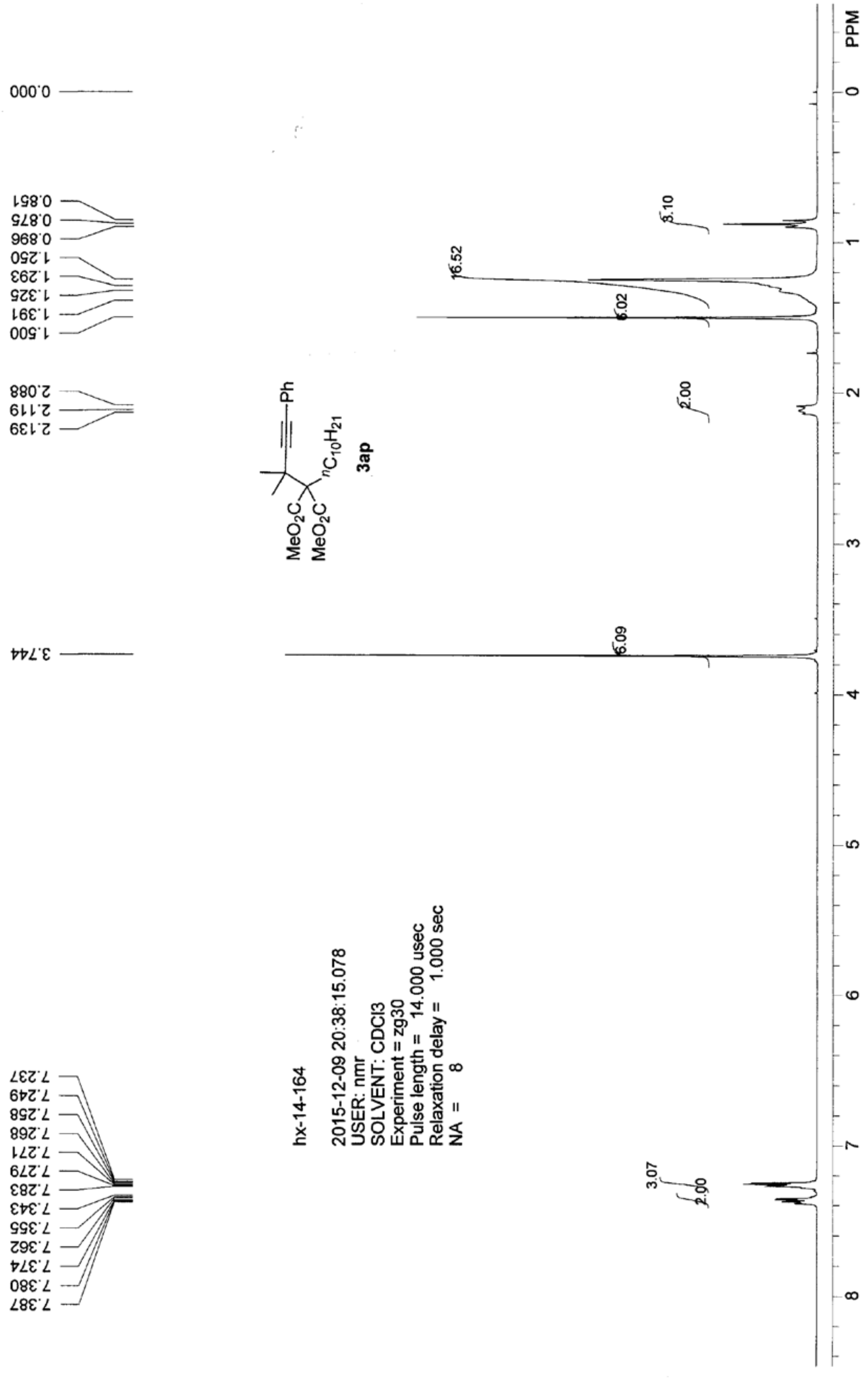

Supplementary Figure 87. ^{1}H NMR (300 MHz, $CDCl_3$) spectrum for 3ap.

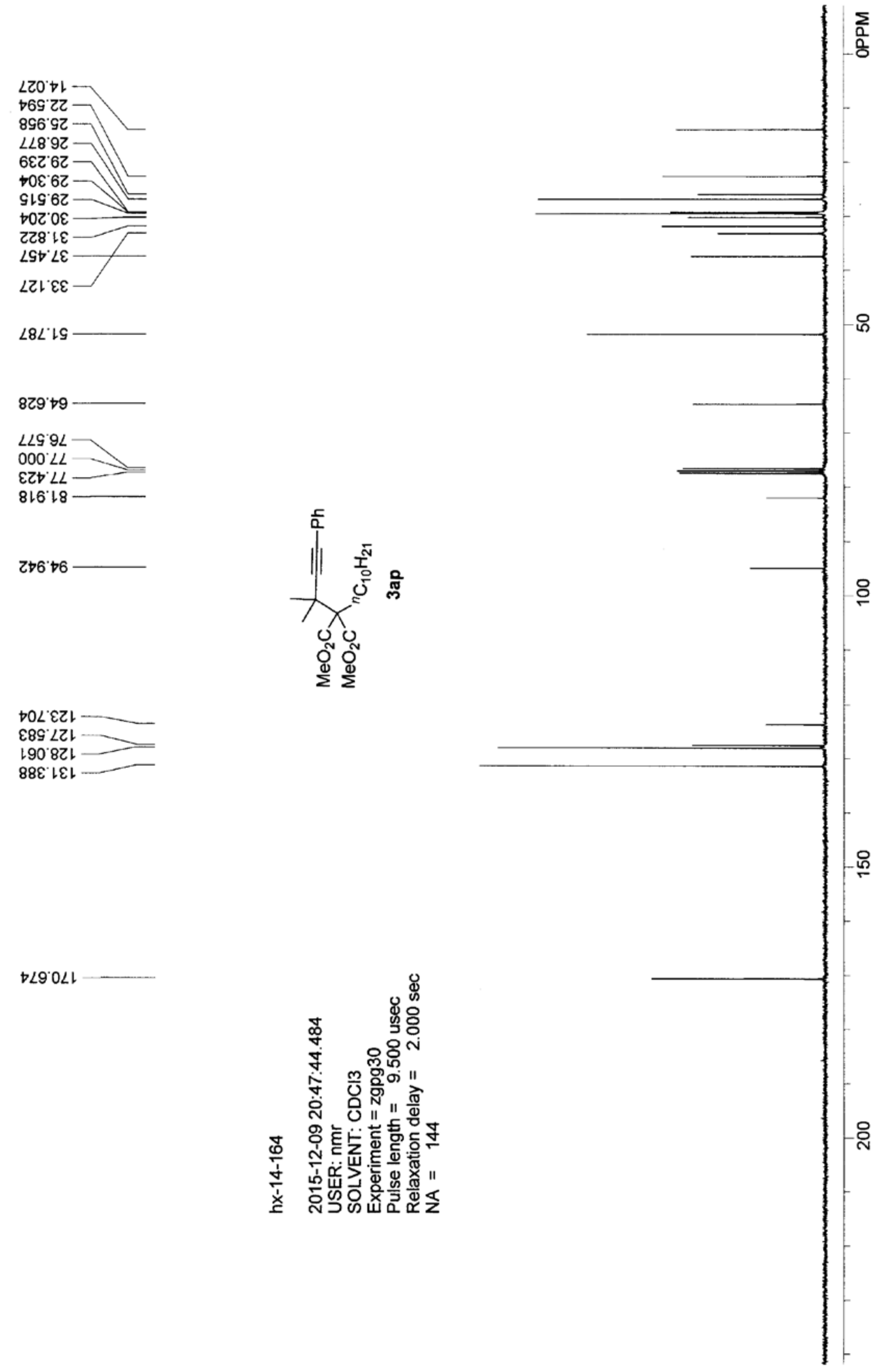

Supplementary Figure 88. ^{13}**C NMR (75 MHz,** $CDCl_3$**) spectrum for 3ap.**

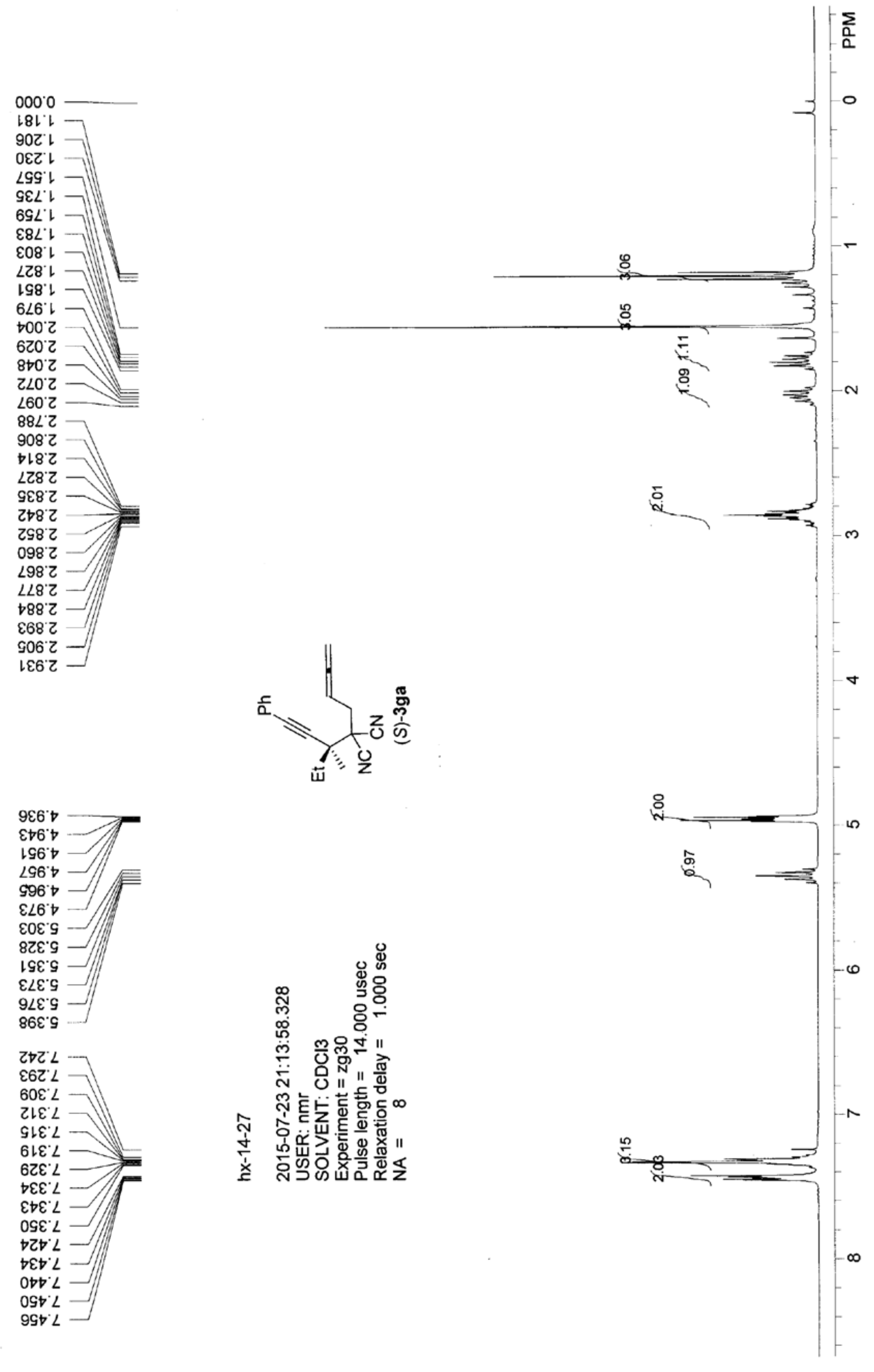

Supplementary Figure 89. ^{1}**H NMR (300 MHz, CDCl$_3$) spectrum for (*S*)-3ga.**

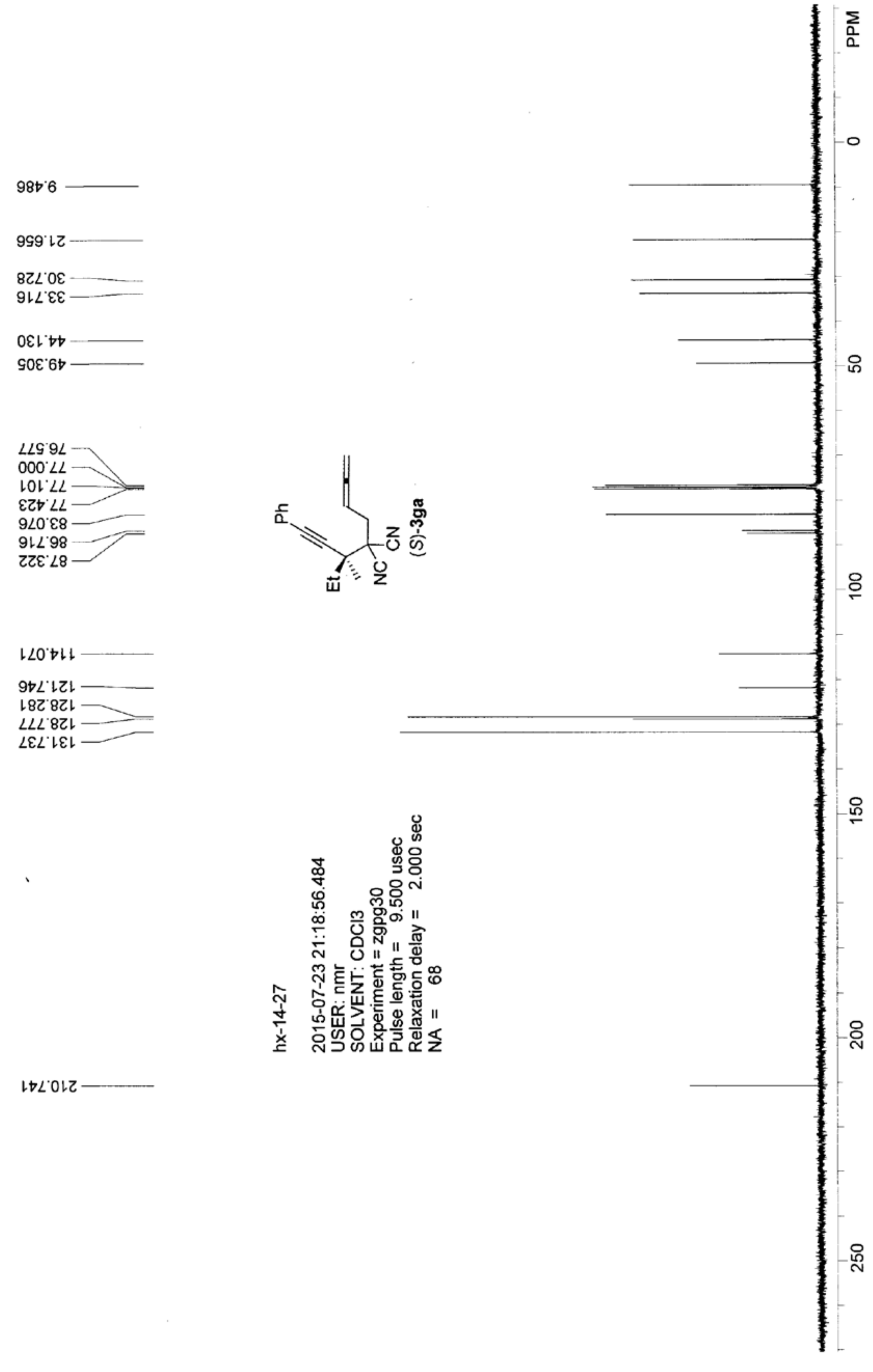

Supplementary Figure 90. ^{13}C **NMR (75 MHz,** $CDCl_3$**) spectrum for (*S*)-3ga.**

hx-14-27

实验单位: zju
实验时间: 2015-07-21, 14:36:13
谱图文件:D:\浙大智达\N2000\样品\B1036.org

实验者: hx
报告时间: 2015-07-21, 15:33:26
积分方法:面积归一法

实验内容简介:
IC, n-hexane/i-PrOH = 200/1, 214 nm, 0.6 ml/min

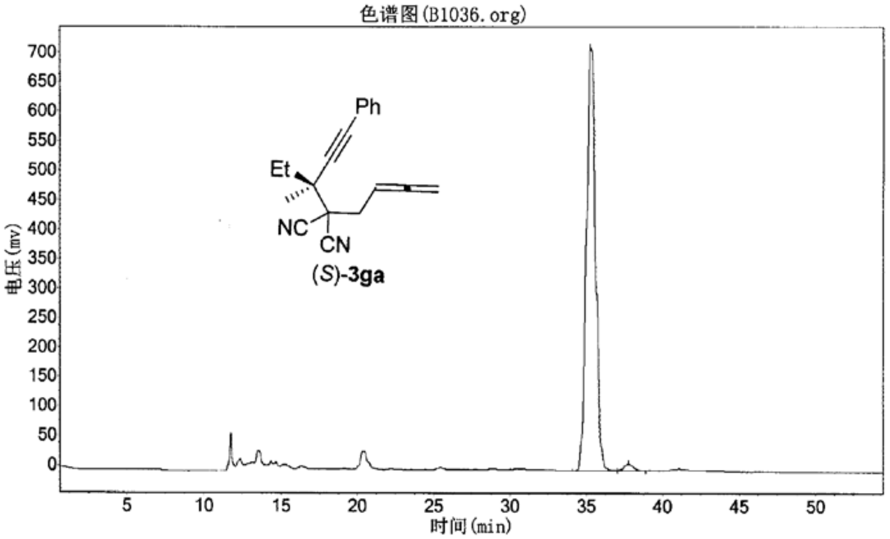

分析结果表

峰号	峰名	保留时间	峰高	峰面积	含量
1		35.277	717043.563	30007784.000	98.3257
2		37.778	11978.059	510987.219	1.6743
总计			729021.621	30518771.219	100.0000

Supplementary Figure 91. HPLC spectrum for (*S*)-3ga.

hx-12-184

实验单位：zju
实验时间：2015-07-21, 15:35:12
谱图文件:D:\浙大智达\N2000\样品\C0000.org

实验者：hx
报告时间：2015-07-21, 16:35:26
积分方法:面积归一法

实验内容简介：
IC, n-hexane/i-PrOH = 200/1, 214 nm, 0.6 ml/min

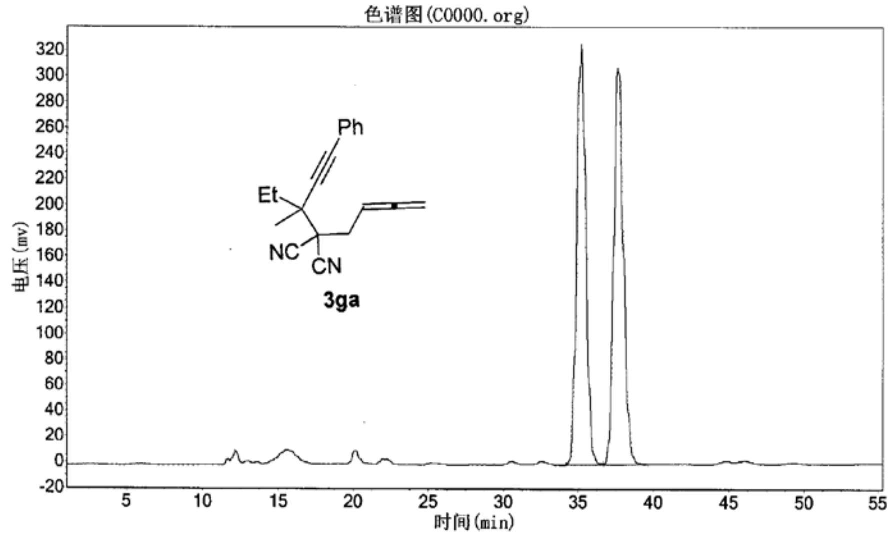

分析结果表

峰号	峰名	保留时间	峰高	峰面积	含量
1		35.205	323444.813	13714475.000	49.9681
2		37.625	304801.219	13731961.000	50.0319
总计			628246.031	27446436.000	100.0000

Supplementary Figure 92. HPLC spectrum for 3ga.

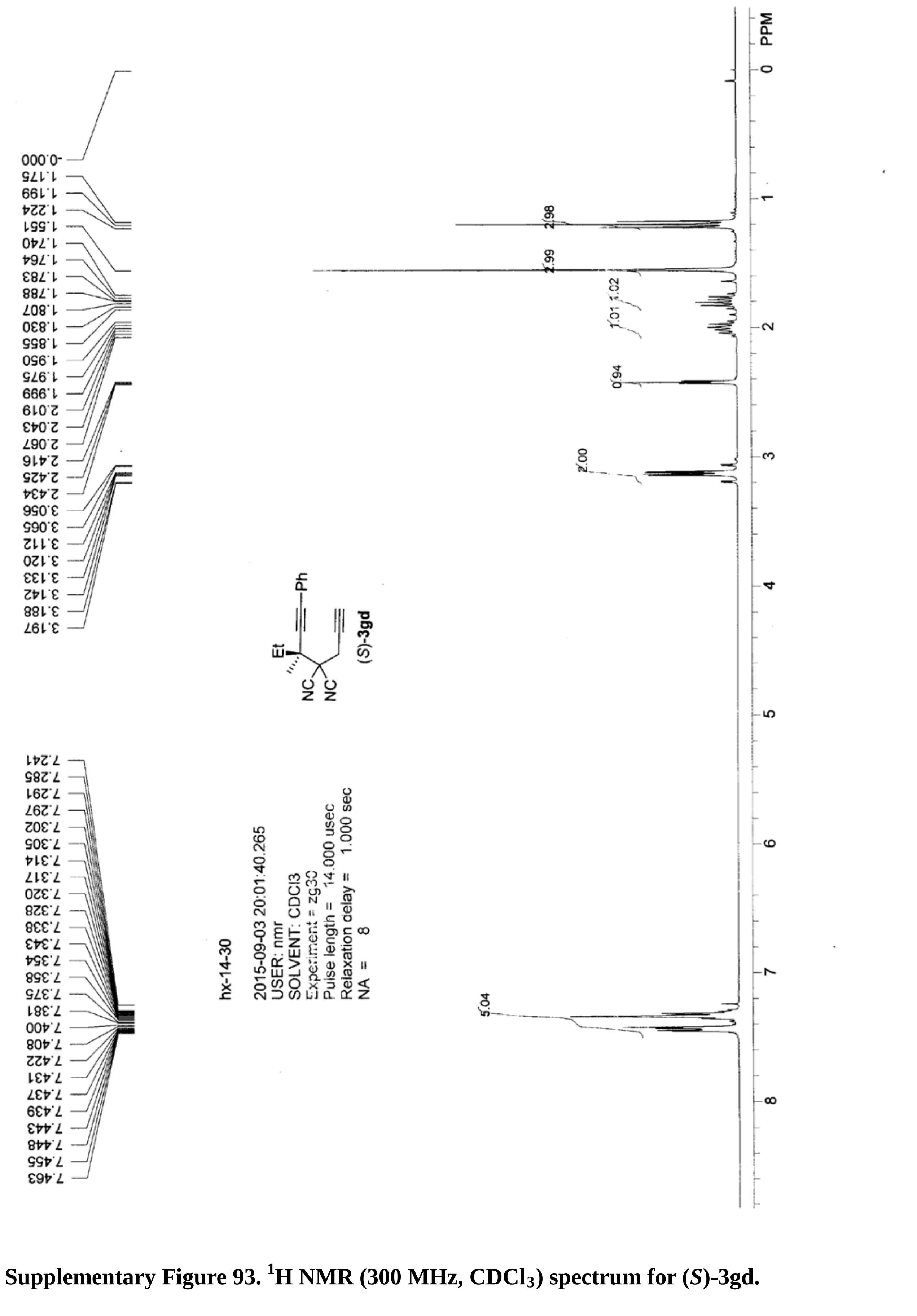

Supplementary Figure 93. ^{1}H NMR (300 MHz, $CDCl_3$) spectrum for (*S*)-3gd.

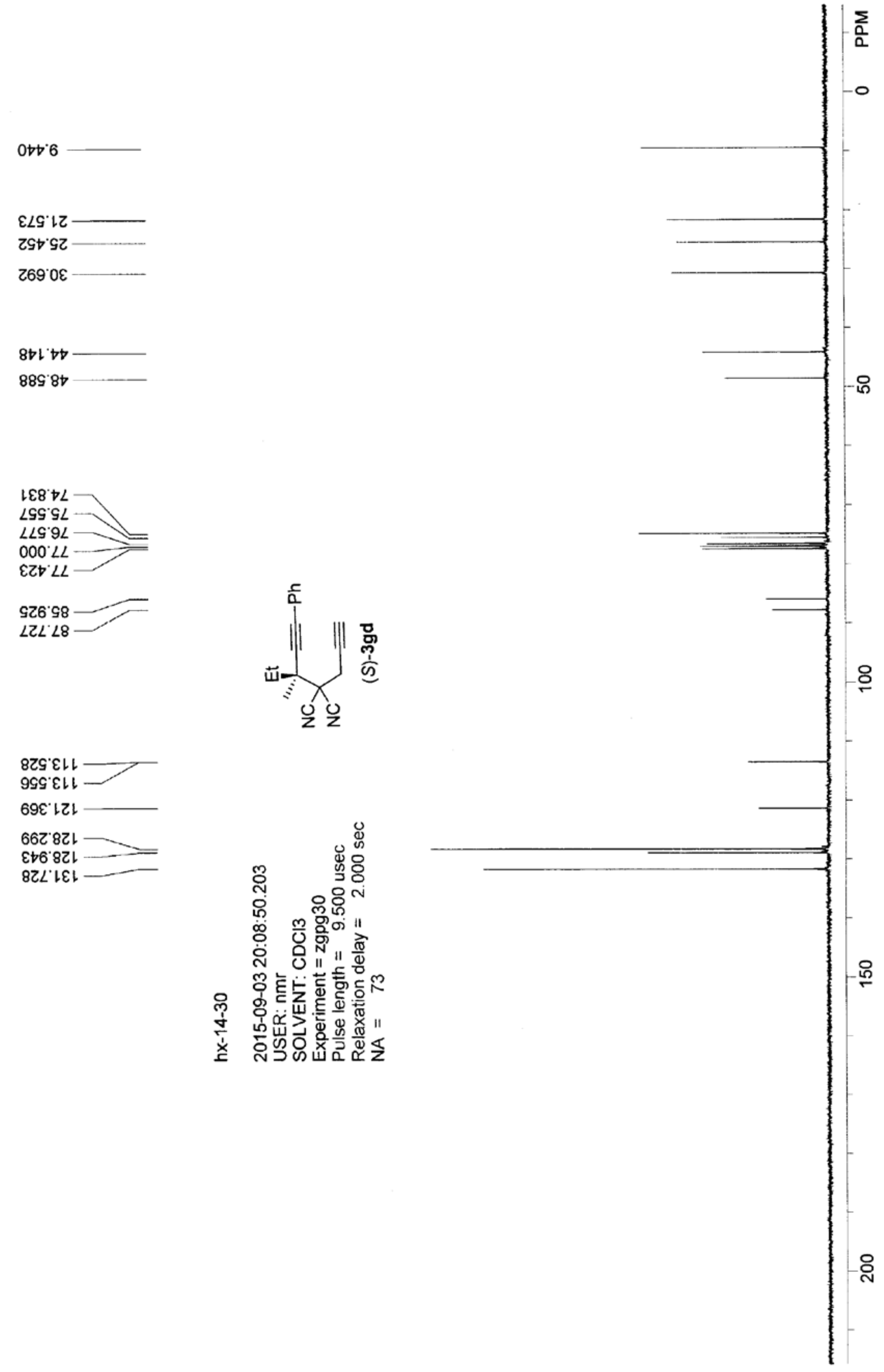

Supplementary Figure 94. ^{13}**C NMR (75 MHz, CDCl$_3$) spectrum for (*S*)-3gd.**

hx-14-30-od-h-200-1-1-214

实验时间：2015/9/18, 14:18:05　　　　　　　　报告时间：2015/9/18, 15:03:25
谱图文件:D:\zhuguangjiong\hx\20150918\hx-14-30-od-200-1-1-214.org

实验内容简介：

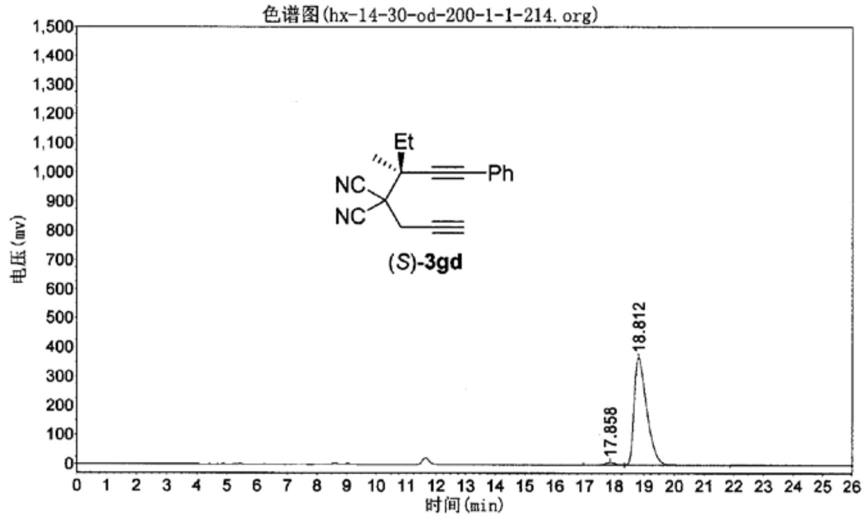

分析结果表

峰号	峰名	保留时间	峰高	峰面积	含量
1		17.858	7989.686	189632.578	1.7465
2		18.812	366917.875	10668008.000	98.2535
总计			374907.561	10857640.578	100.0000

Supplementary Figure 95. HPLC spectrum for (*S*)-3gd.

hx-14-15-od-h-200-1-1-214

实验时间：2015/9/18, 13:48:42　　报告时间：2015/9/18, 15:02:17
谱图文件:D:\zhuguangjiong\hx\20150918\hx-14-15-od-200-1-1-214.org

实验内容简介：

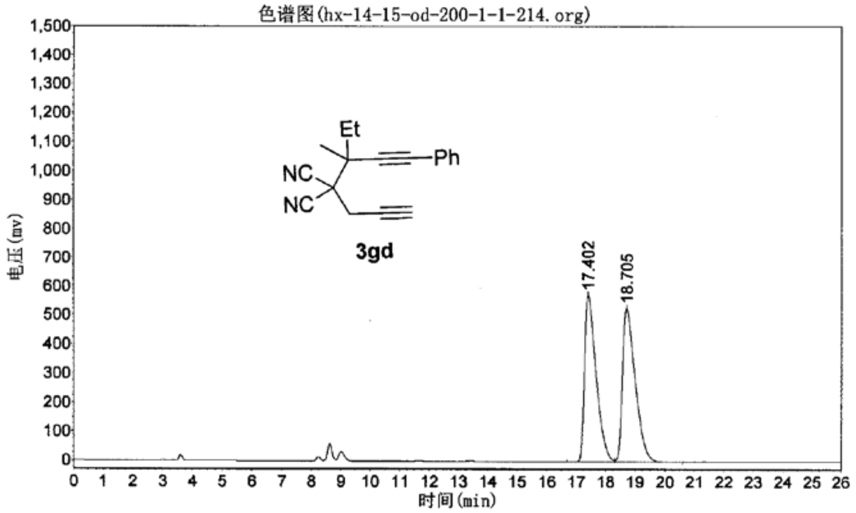

分析结果表

峰号	峰名	保留时间	峰高	峰面积	含量
1		17.402	572824.313	15555898.000	49.6578
2		18.705	526910.063	15770325.000	50.3422
总计			1099734.375	31326223.000	100.0000

Supplementary Figure 96. HPLC spectrum for 3gd.

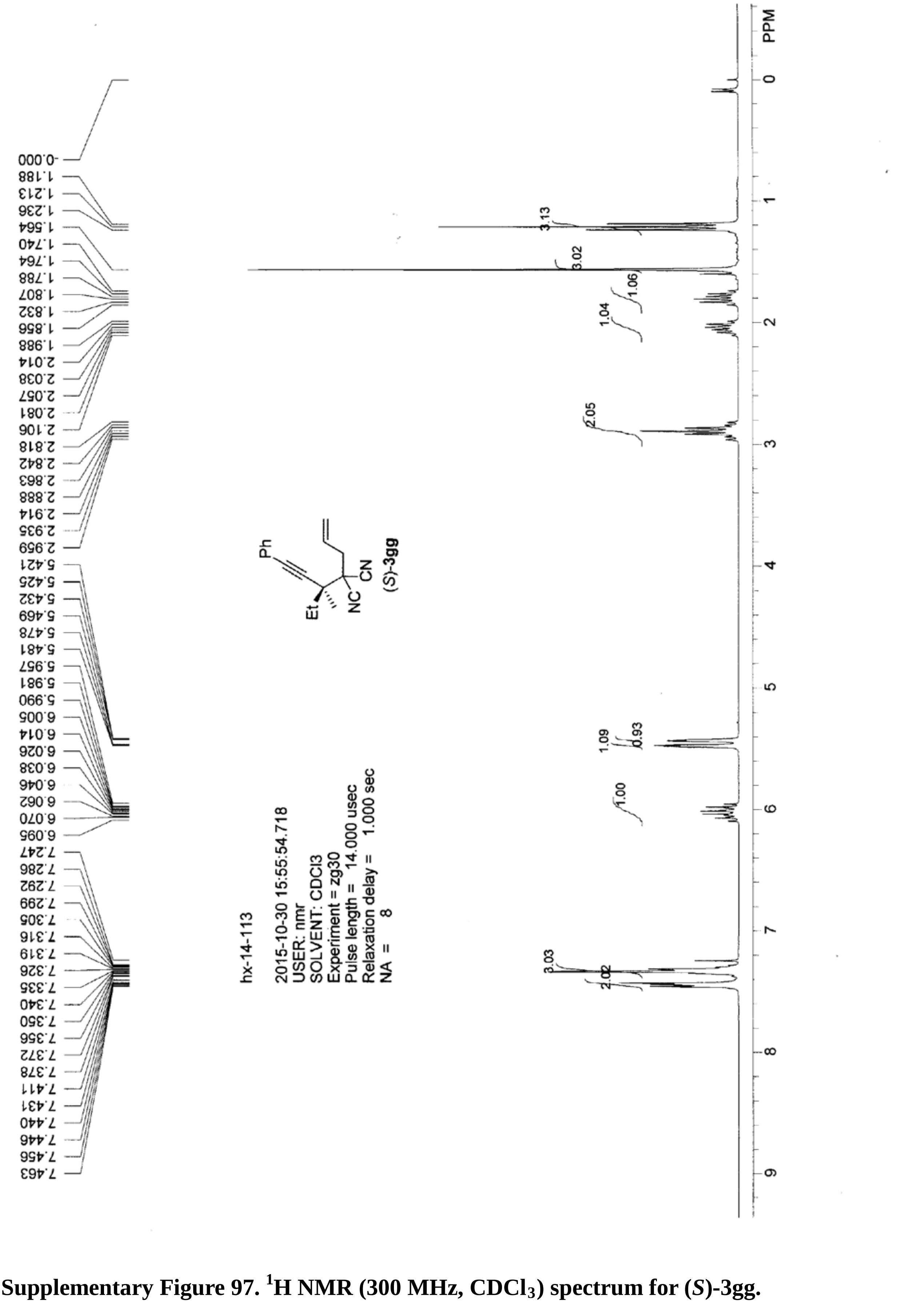

Supplementary Figure 97. ^{1}**H NMR (300 MHz, CDCl$_3$) spectrum for (*S*)-3gg.**

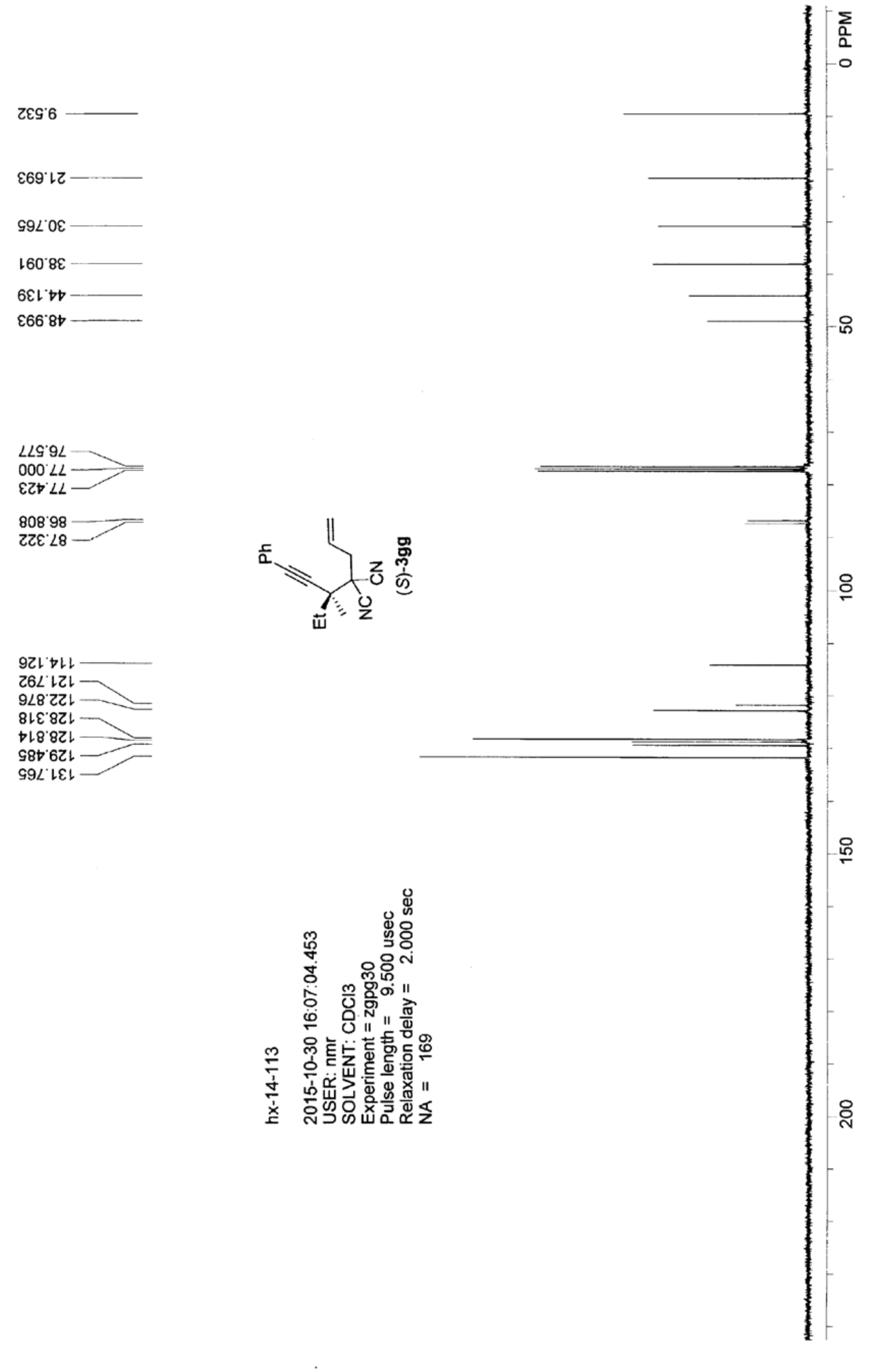

Supplementary Figure 98. **^{13}C NMR (75 MHz, $CDCl_3$) spectrum for (*S*)-3gg.**

hx-14-113

实验单位：zju
实验时间：2015-11-01, 11:32:29
谱图文件:D:\浙大智达\N2000\样品\C0097.org

实验者：hx
报告时间：2015-11-01, 12:58:21
积分方法:面积归一法

实验内容简介：
IC, n-hexane/i-PrOH = 200/1, 214 nm, 0.5 ml/min

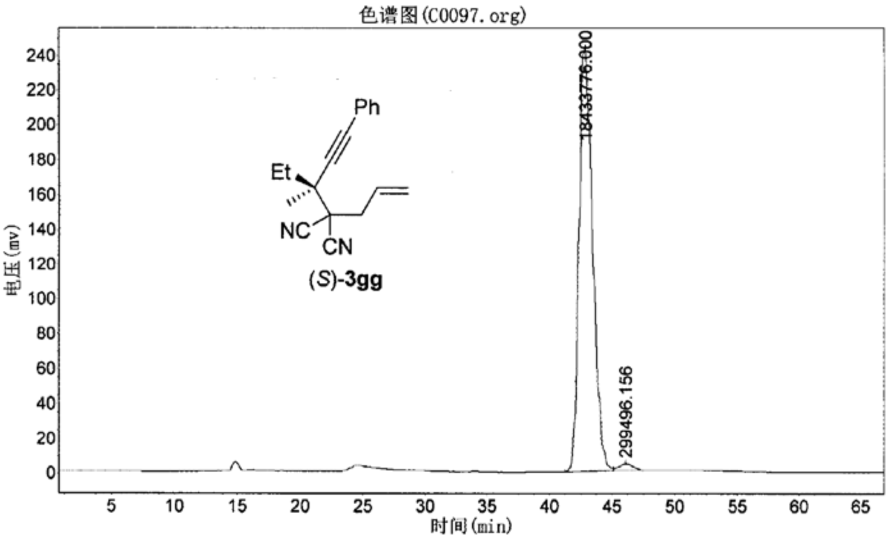

分析结果表

峰号	峰名	保留时间	峰高	峰面积	含量
1		42.890	242371.313	18433776.000	98.4013
2		46.105	3828.926	299496.156	1.5987
总计			246200.239	18733272.156	100.0000

Supplementary Figure 99. HPLC spectrum for (*S*)-3gg.

hx-14-108

实验单位：zju
实验时间：2015-11-01, 9:14:50
谱图文件:D:\浙大智达\N2000\样品\C0095.org

实验者：hx
报告时间：2015-11-01, 10:27:27
积分方法:面积归一法

实验内容简介：
IC, n-hexane/i-PrOH = 200/1, 214 nm, 0.5 ml/min

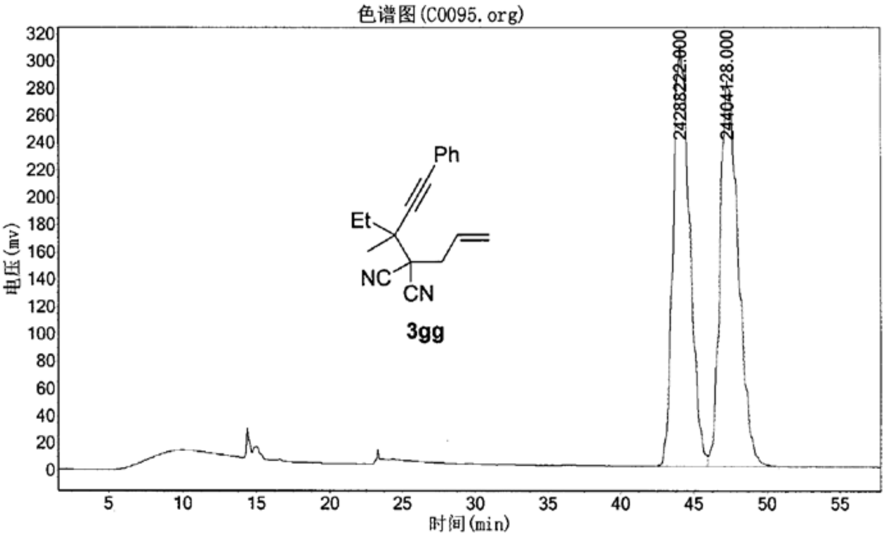

分析结果表

峰号	峰名	保留时间	峰高	峰面积	含量
1		44.052	306119.469	24288222.000	49.8810
2		47.248	278933.188	24404128.000	50.1190
总计			585052.656	48692350.000	100.0000

Supplementary Figure 100. HPLC spectrum for 3gg.

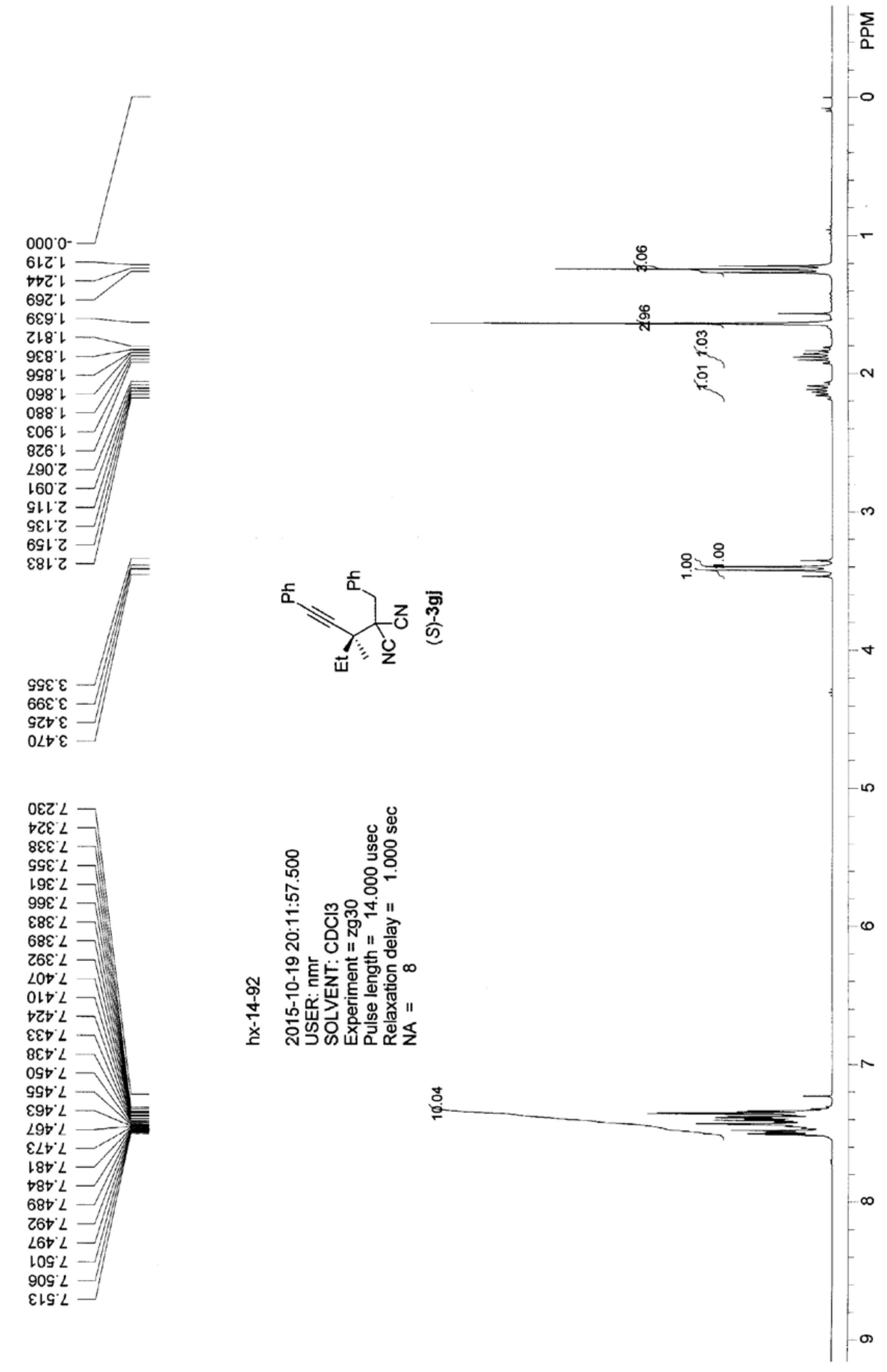

Supplementary Figure 101. ^{1}**H NMR (300 MHz, CDCl$_3$) spectrum for (*S*)-3gj.**

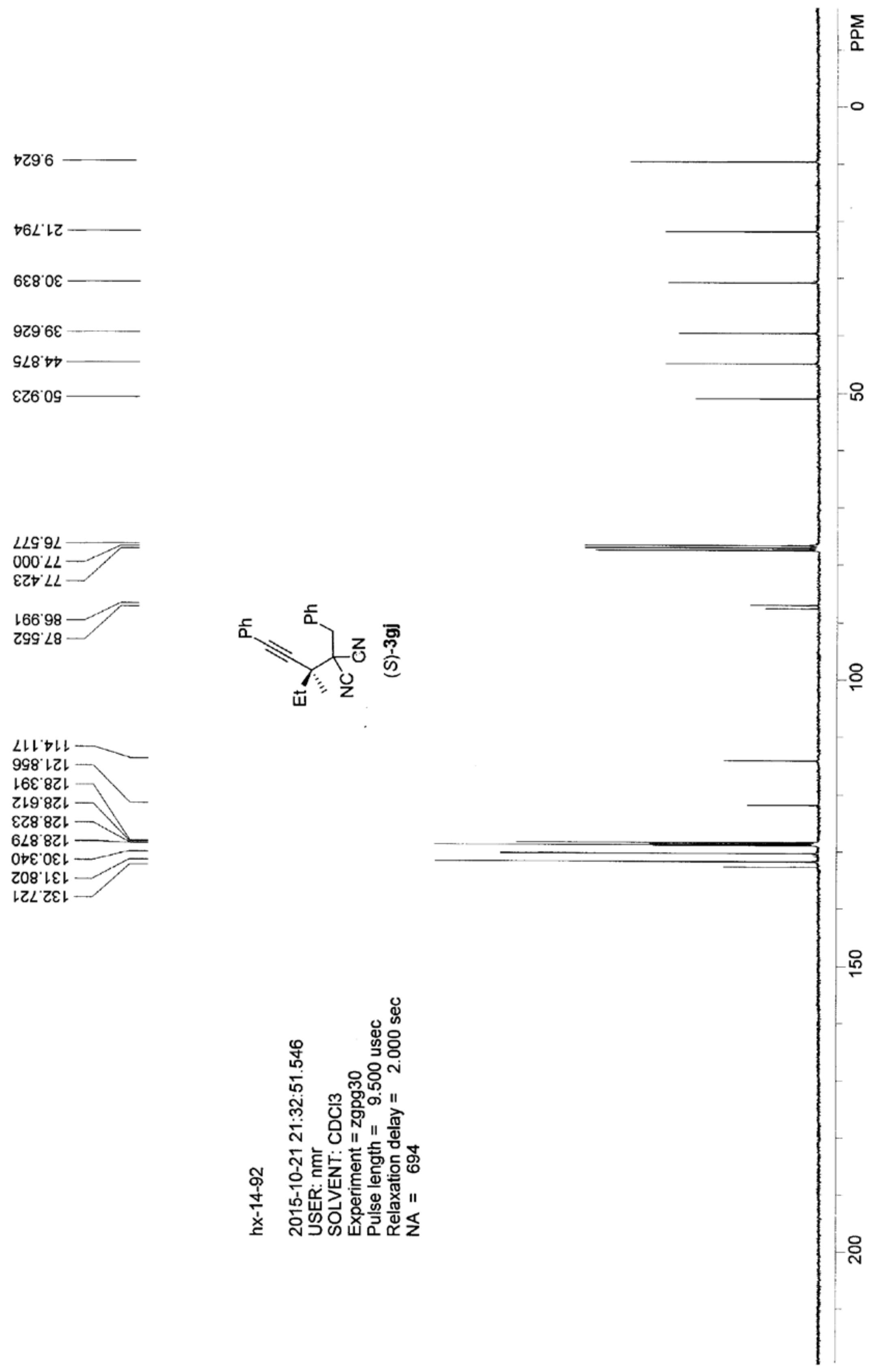

Supplementary Figure 102. **^{13}C NMR (75 MHz, $CDCl_3$) spectrum for (*S*)-3gj.**

hx-14-92

实验单位：zju
实验时间：2015-10-31, 21:04:49
谱图文件:D:\浙大智达\N2000\样品\C0094.org

实验者：hx
报告时间：2015-10-31, 22:00:17
积分方法:面积归一法

实验内容简介：
IC, n-hexane/i-PrOH = 200/1, 214 nm, 1.0 ml/min

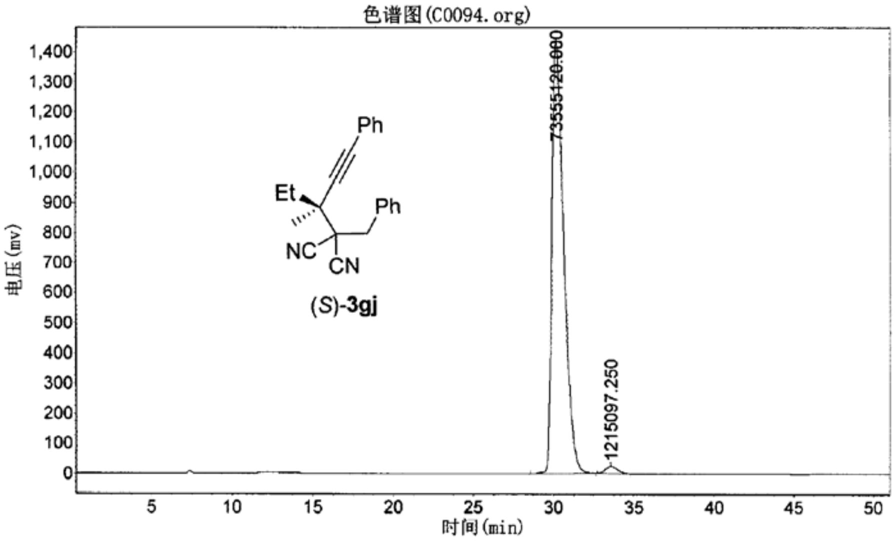

分析结果表

峰号	峰名	保留时间	峰高	峰面积	含量
1		30.167	1431514.375	73555120.000	98.3749
2		33.588	23640.537	1215097.250	1.6251
总计			1455154.912	74770217.250	100.0000

Supplementary Figure 103. HPLC spectrum for (*S*)-3gj.

hx-14-90

实验单位：zju
实验时间：2015-10-31,19:57:53
谱图文件:D:\浙大智达\N2000\样品\C0093.org

实验者：hx
报告时间：2015-10-31,21:03:26
积分方法:面积归一法

实验内容简介：
IC, n-hexane/i-PrOH = 200/1, 214 nm,1.0 ml/min

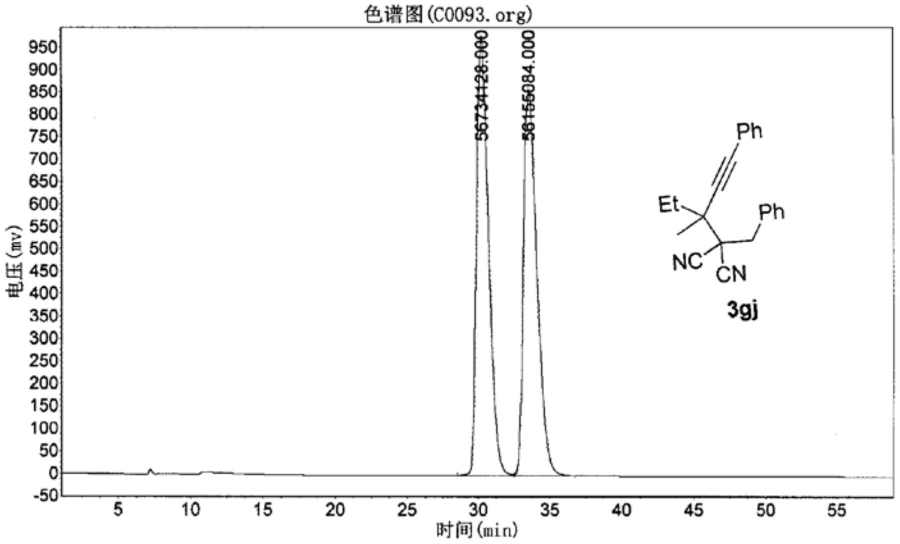

分析结果表

峰号	峰名	保留时间	峰高	峰面积	含量
1		30.193	971600.063	56734128.000	50.2565
2		33.492	860516.563	56155084.000	49.7435
总计			1832116.625	112889212.000	100.0000

Supplementary Figure 104. HPLC spectrum for 3gj.

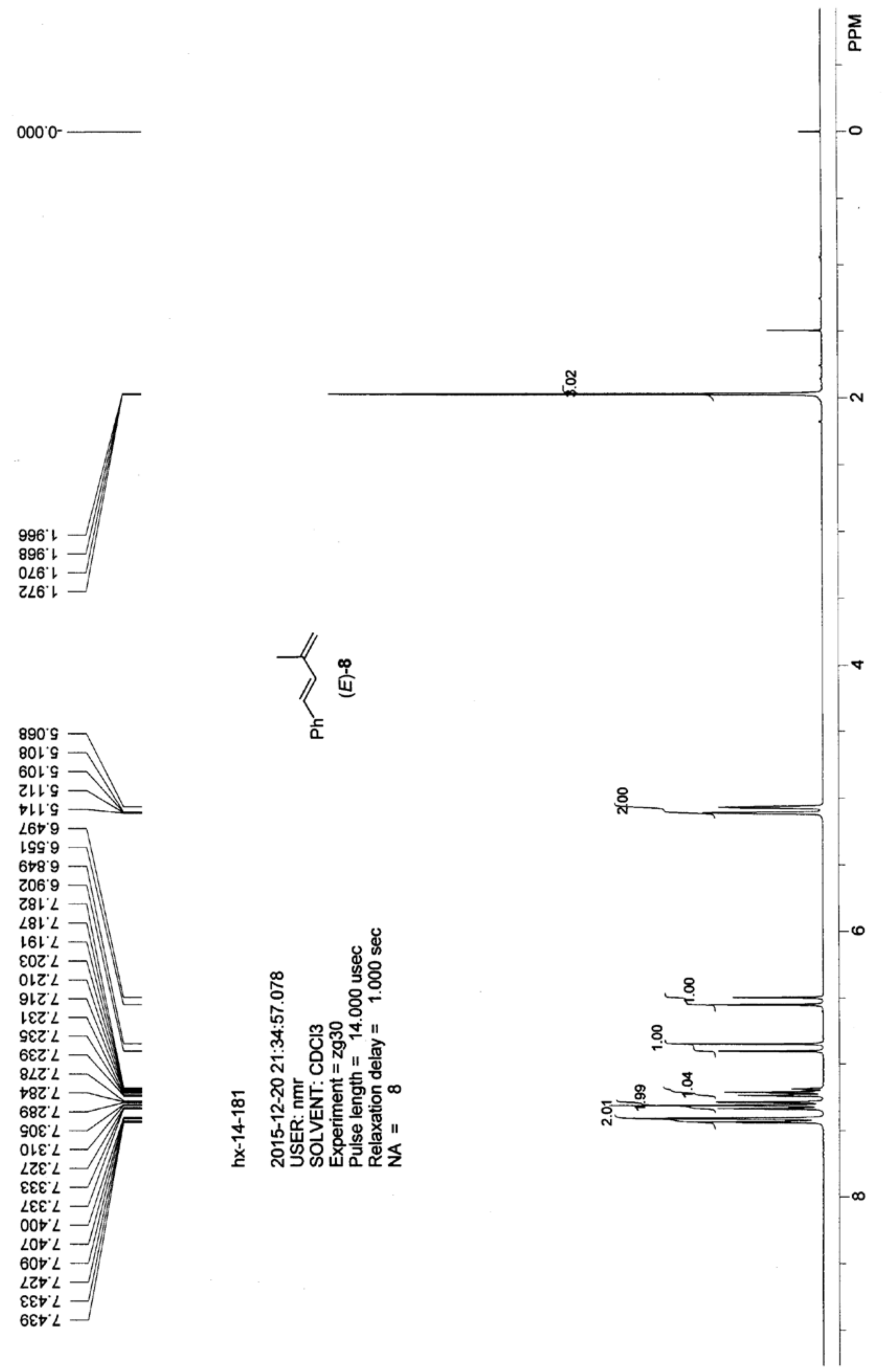

Supplementary Figure 105. ^{1}**H NMR (300 MHz,** $CDCl_3$**) spectrum for (*E*)-8.**

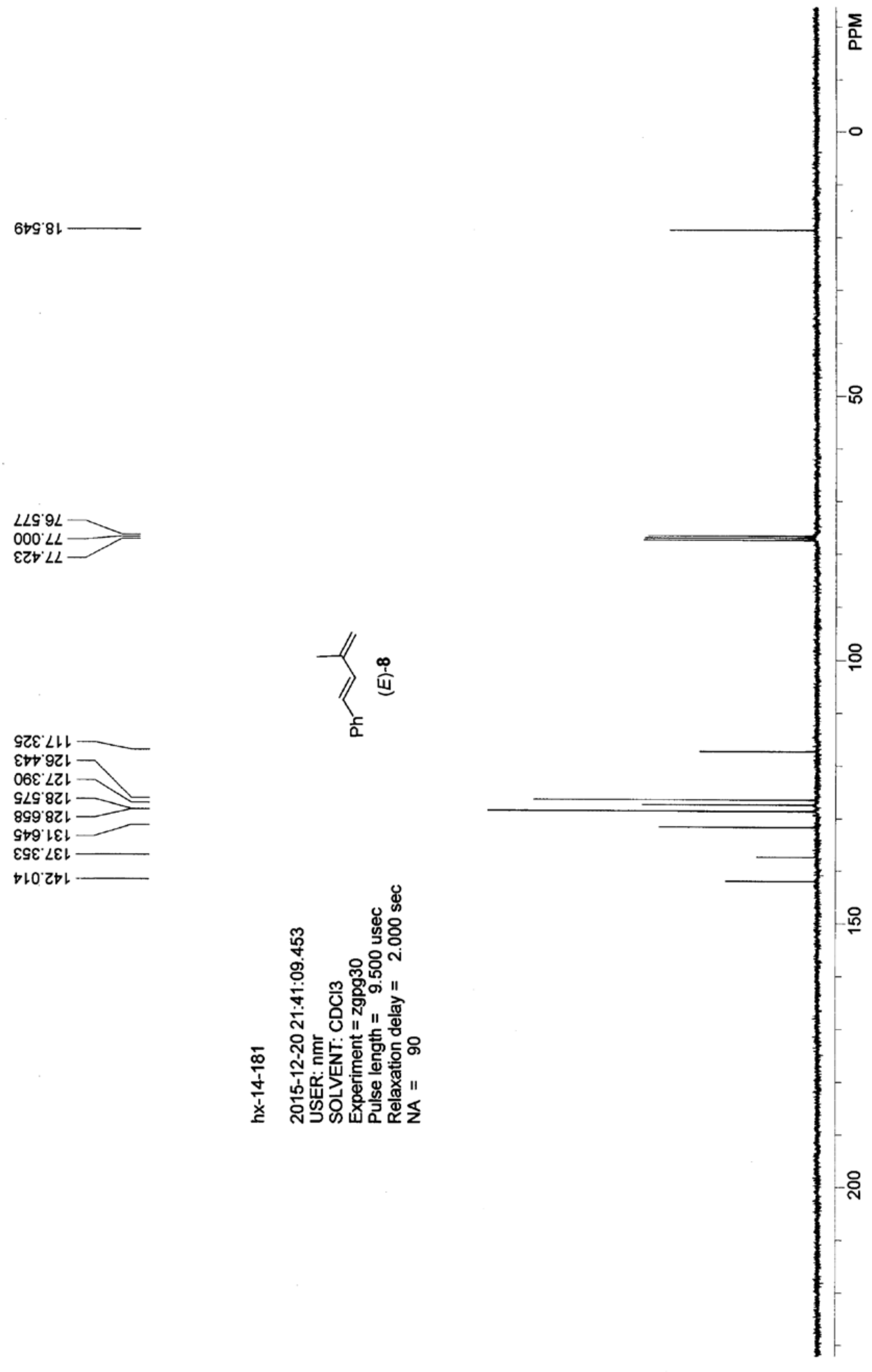

Supplementary Figure 106. ^{13}**C NMR (75 MHz,** $CDCl_3$**) spectrum for (*E*)-8.**

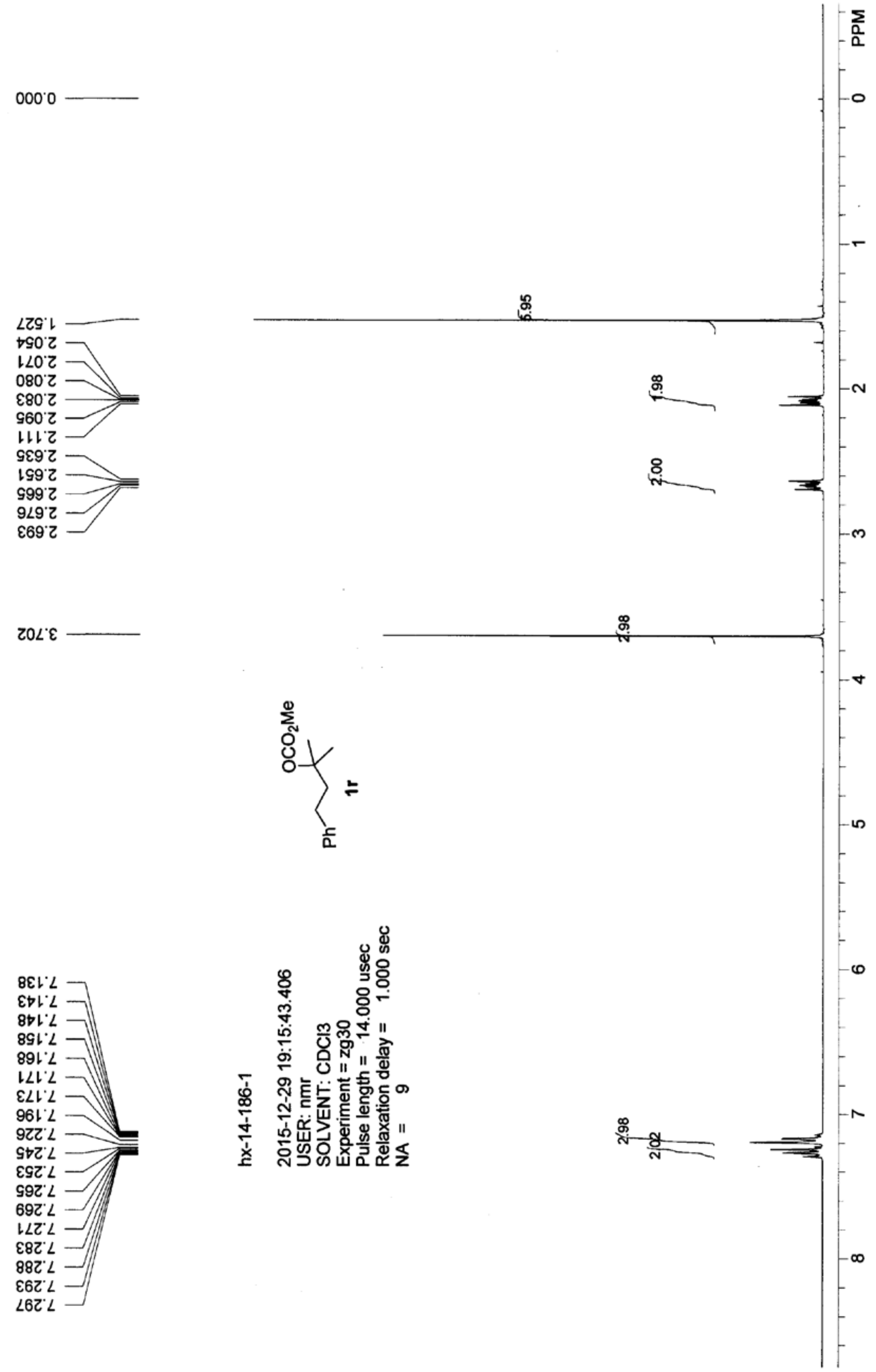

Supplementary Figure 107. ^{1}H **NMR (300 MHz,** $CDCl_3$**) spectrum for 1r.**

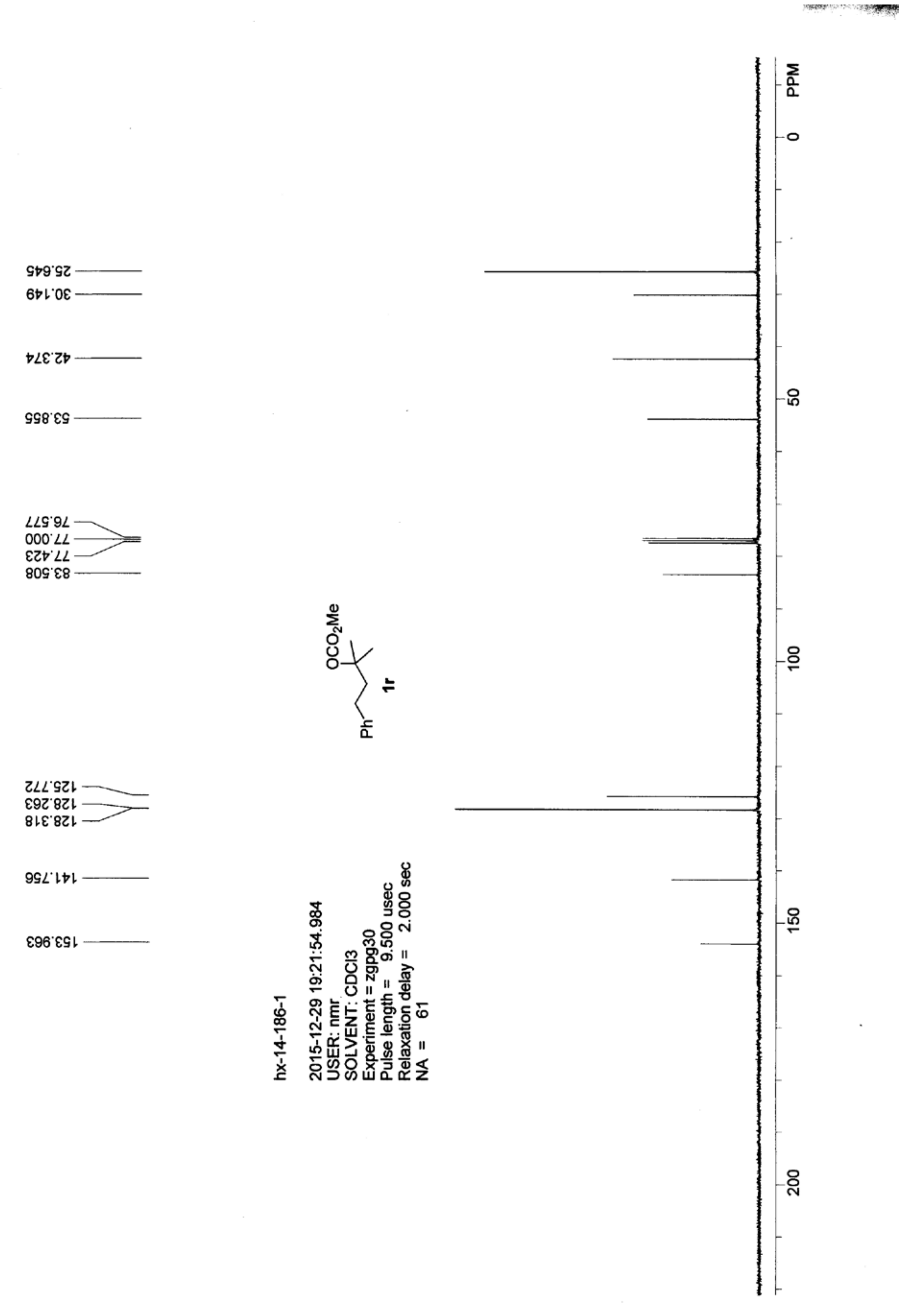

Supplementary Figure 108. **^{13}C NMR (75 MHz, $CDCl_3$) spectrum for 1r.**

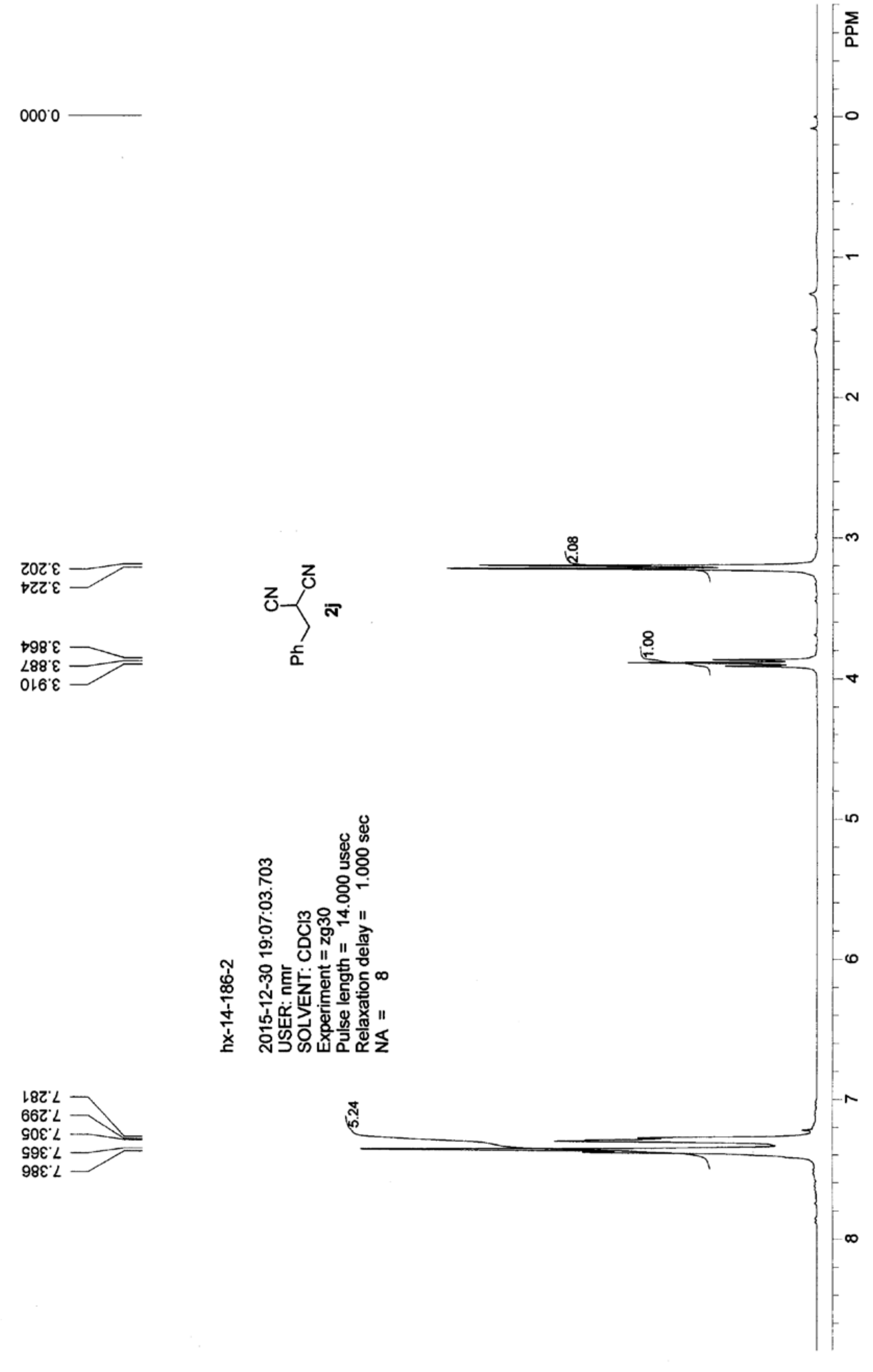

Supplementary Figure 109. ^{1}**H NMR (300 MHz,** $CDCl_3$**) spectrum for 2j.**

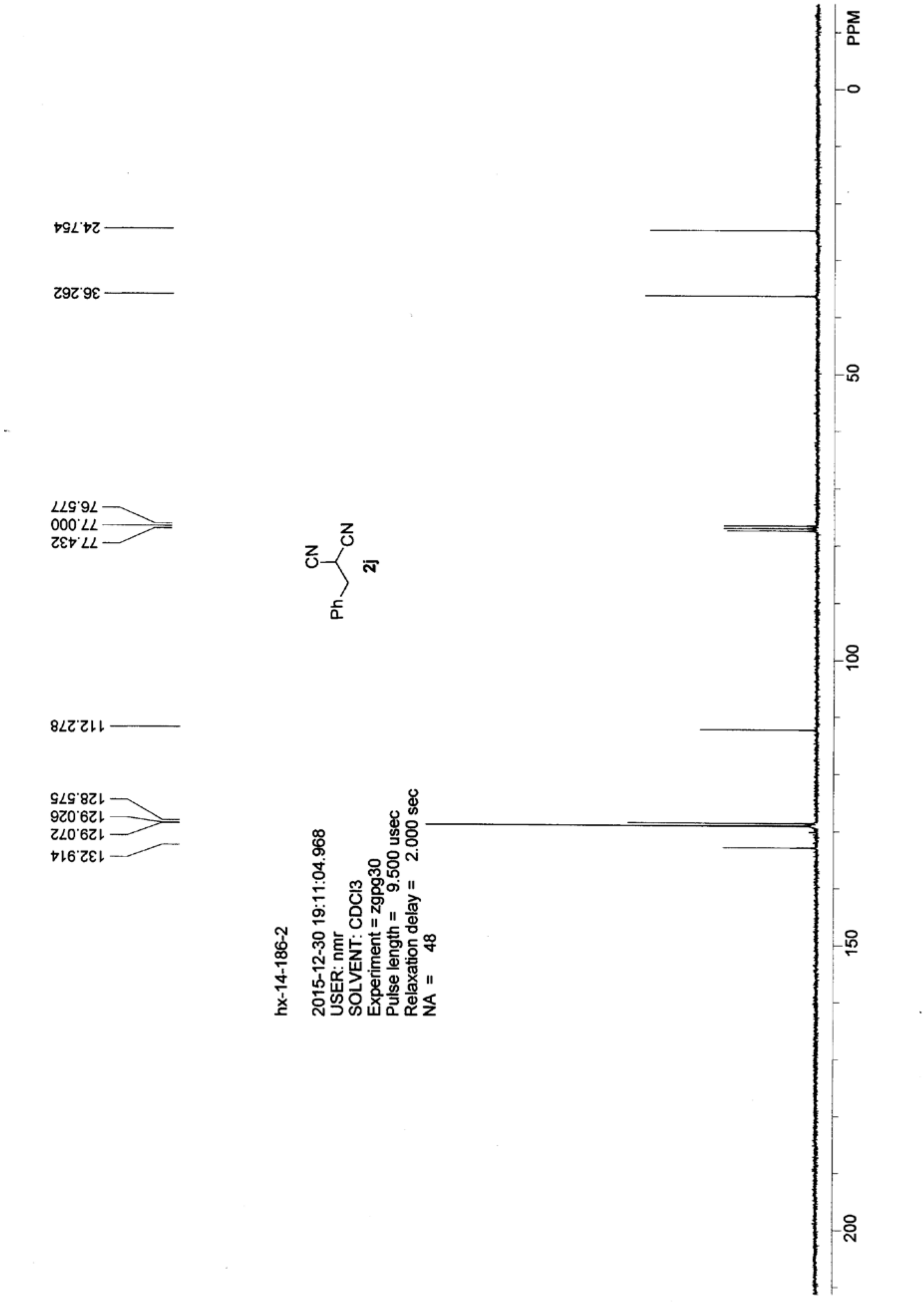

Supplementary Figure 110. ^{13}**C NMR (75 MHz, CDCl$_3$) spectrum for 2j.**

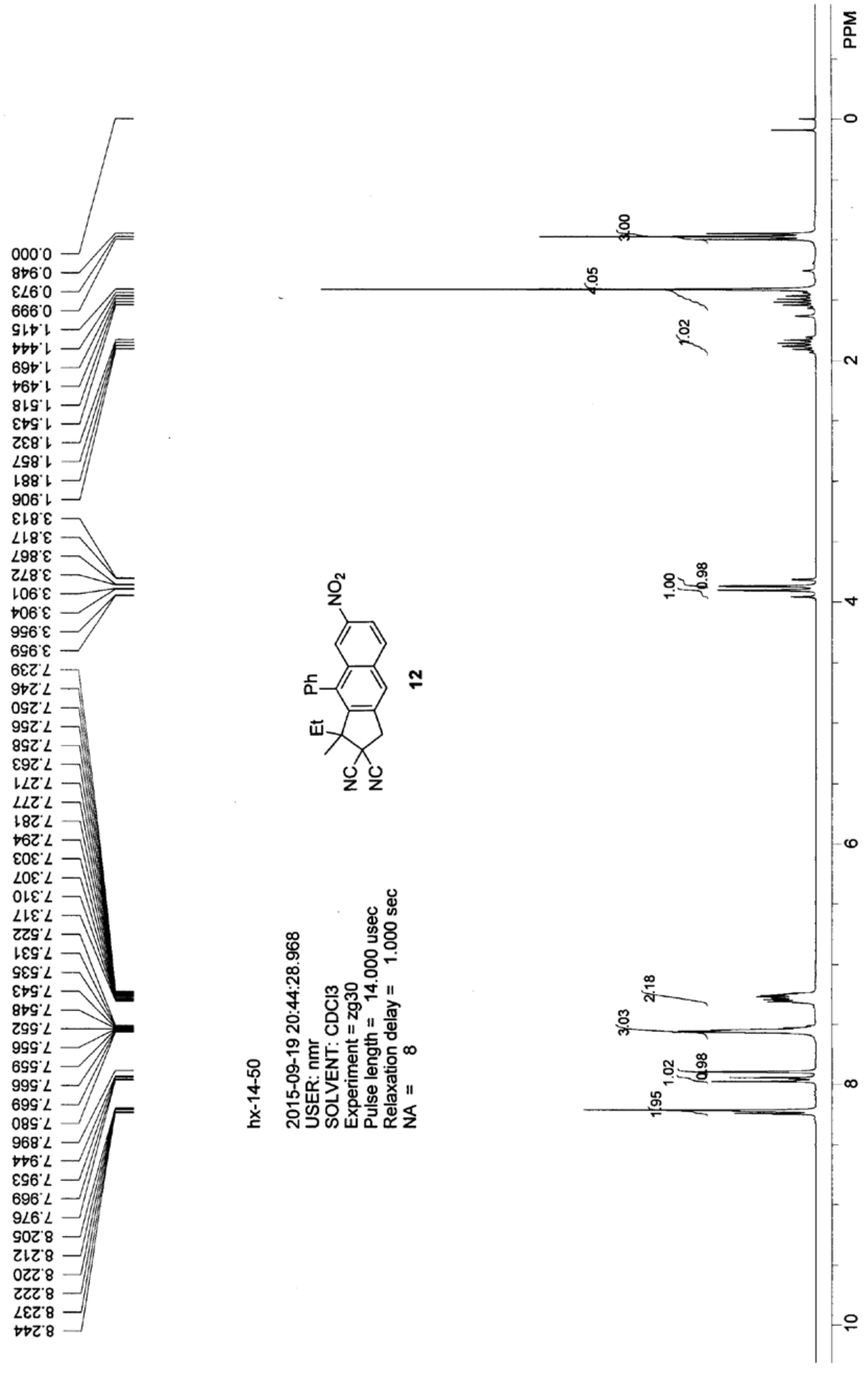

Supplementary Figure 111. **^{1}H NMR (300 MHz, $CDCl_3$) spectrum for 12.**

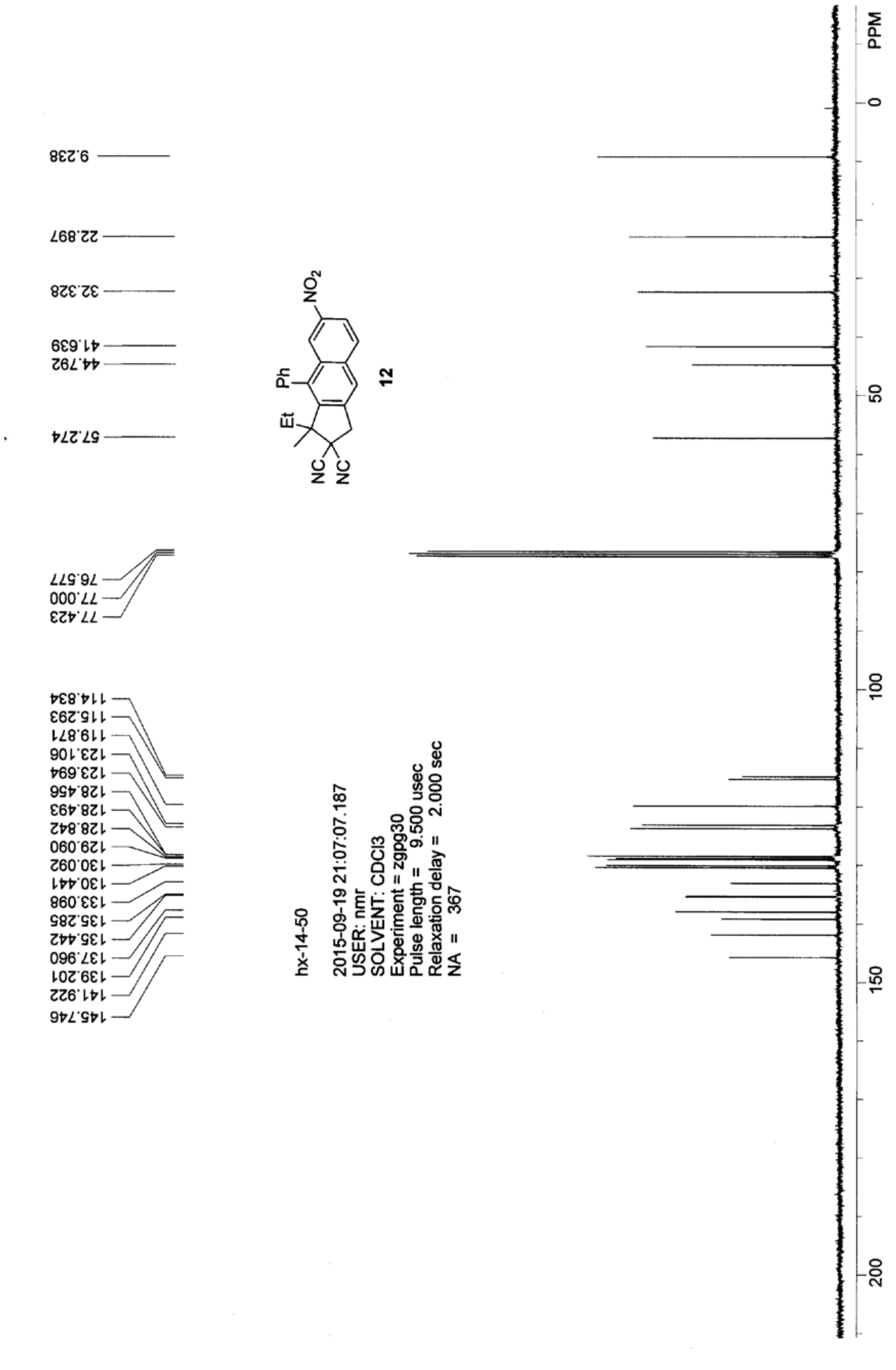

Supplementary Figure 112. ^{13}**C NMR (75 MHz, CDCl$_3$) spectrum for 12.**

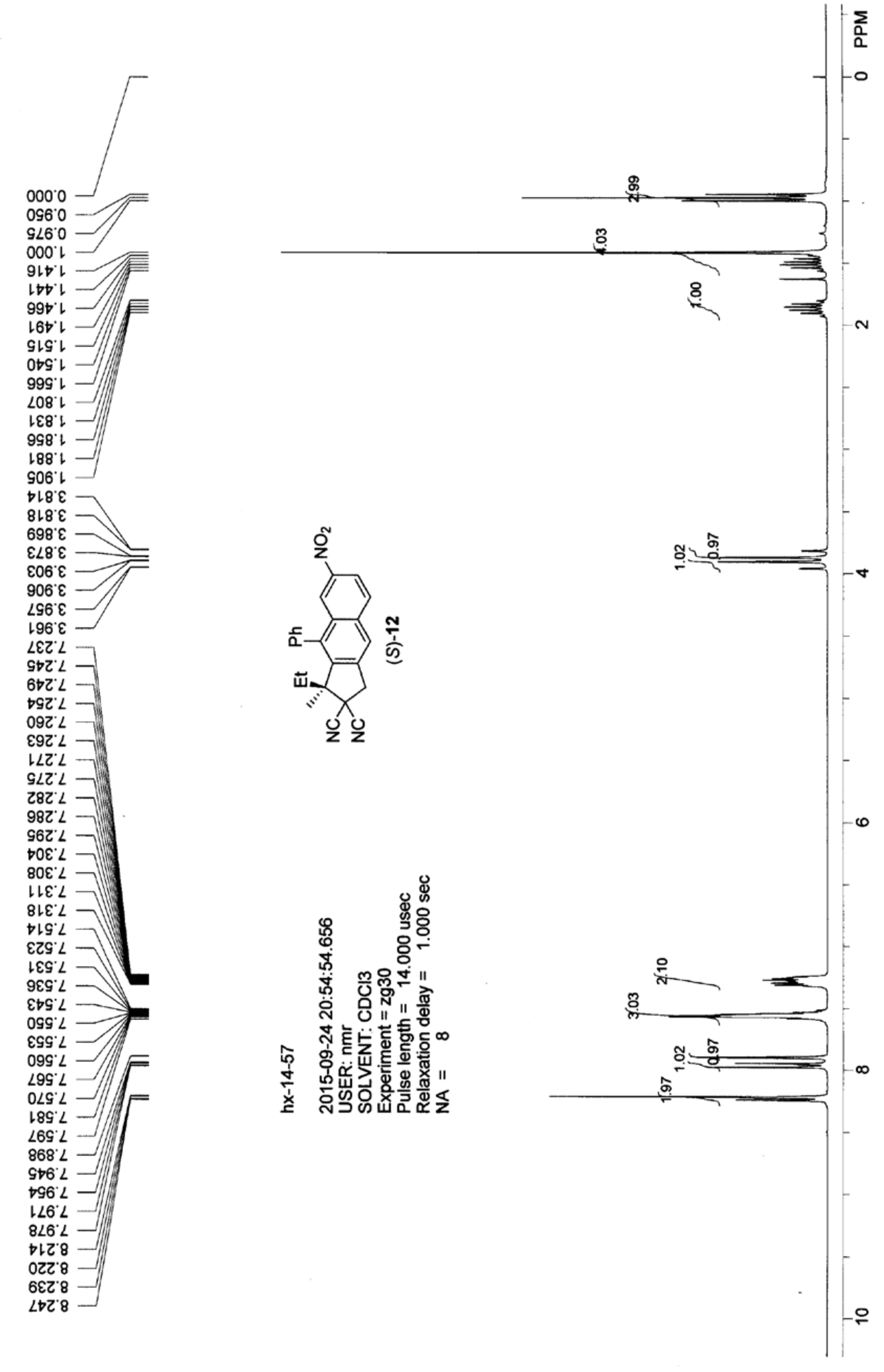

Supplementary Figure 113. ^{1}**H NMR (300 MHz, CDCl$_3$) spectrum for (*S*)-12.**

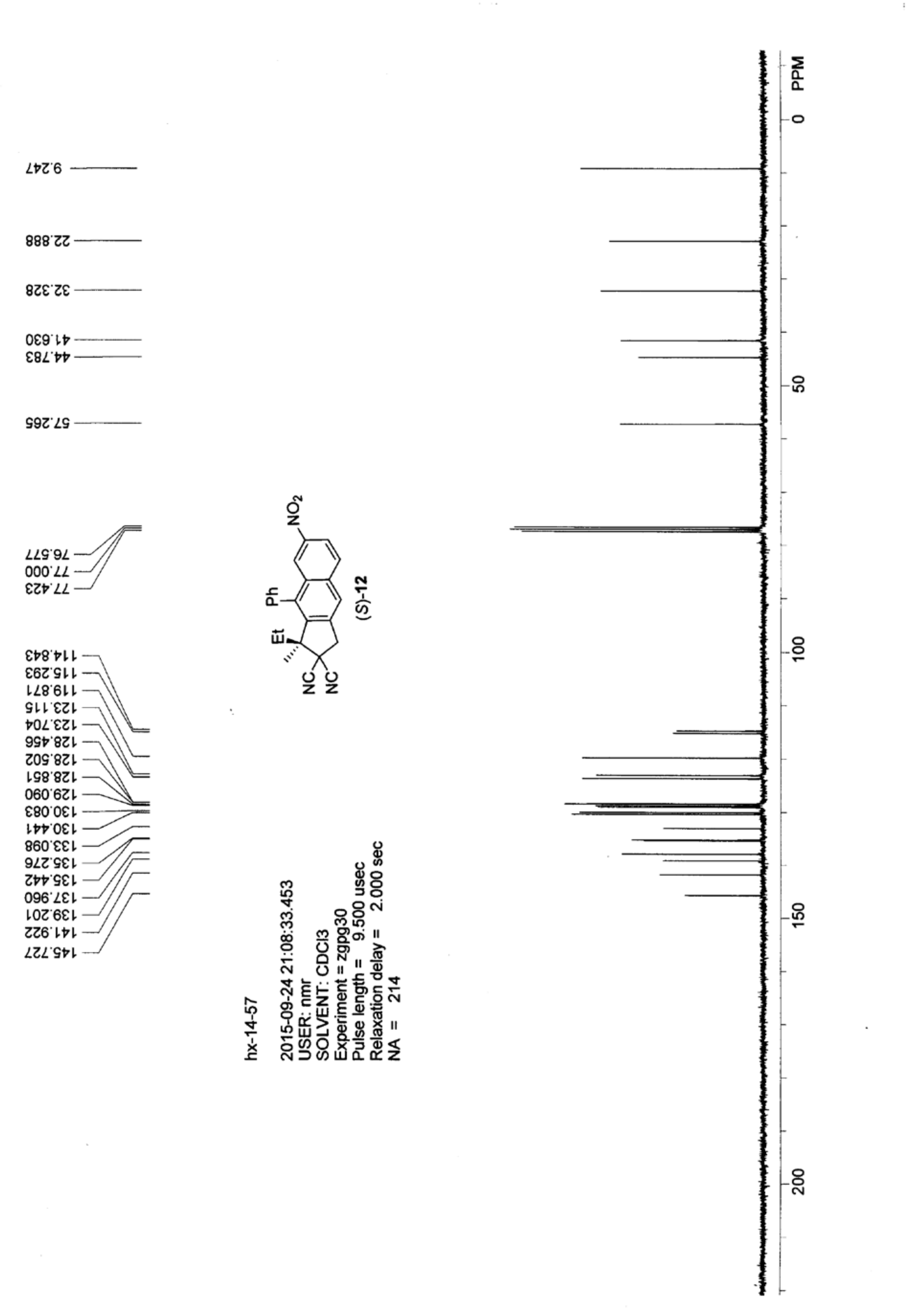

Supplementary Figure 114. ^{13}**C NMR (75 MHz, CDCl**$_3$**) spectrum for (*S*)-12.**

hx-14-57-od-h-80-20-1-214

实验时间：2015-09-30, 16:16:54　　　　报告时间：2015-10-10, 12:19:17
谱图文件:d:\zhuguangjiong\hx\20150930\hx-14-57-od-h-80-20-1-214.org

实验内容简介：

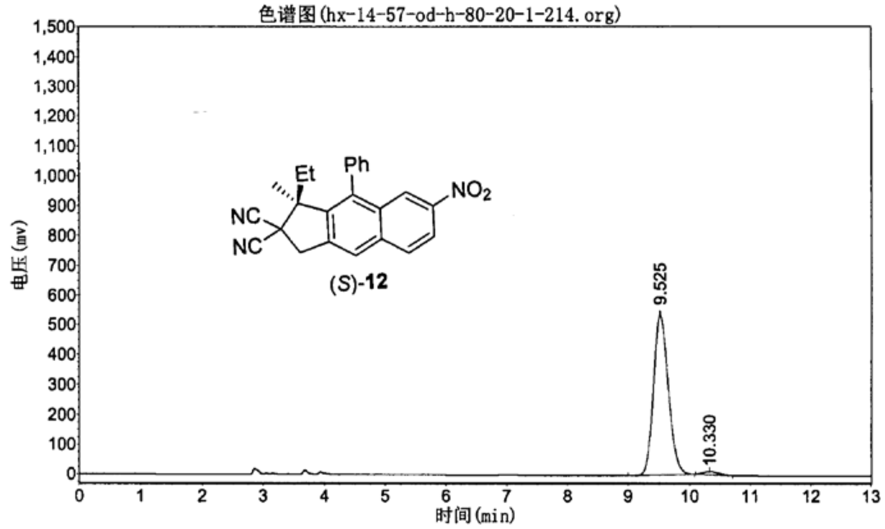

分析结果表

峰号	峰名	保留时间	峰高	峰面积	含量
1		9.525	534236.688	8903052.000	98.0442
2		10.330	10970.186	177601.500	1.9558
总计			545206.873	9080653.500	100.0000

Supplementary Figure 115. HPLC spectrum for (*S*)-12.

hx-14-50-od-h-80-20-1-214

实验时间：2015/9/30, 15:45:55 报告时间：2015/9/30, 16:03:50
谱图文件:D:\zhuguangjiong\hx\20150930\hx-14-50-od-h-80-20-1-214.org

实验内容简介：

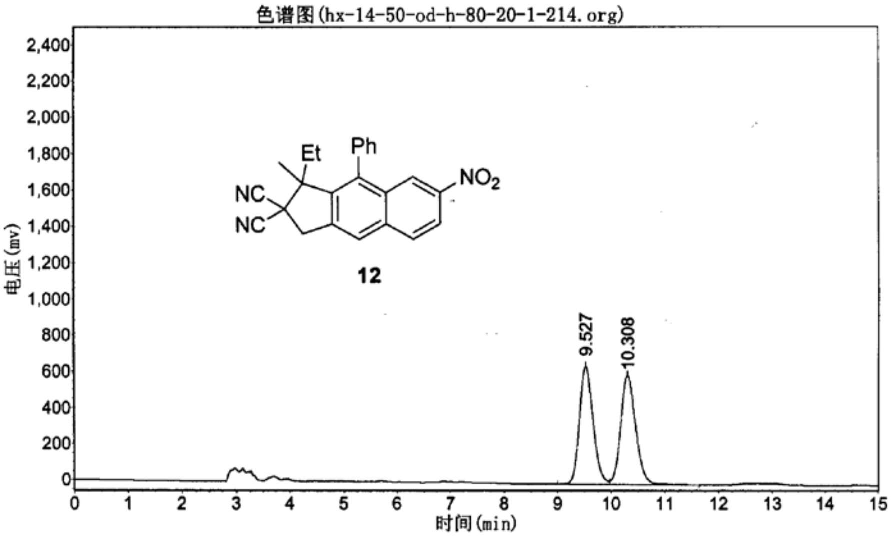

分析结果表

峰号	峰名	保留时间	峰高	峰面积	含量
1		9.527	652430.313	11643319.000	49.9208
2		10.308	602055.688	11680267.000	50.0792
总计			1254486.000	23323586.000	100.0000

Supplementary Figure 116. HPLC spectrum for 12.

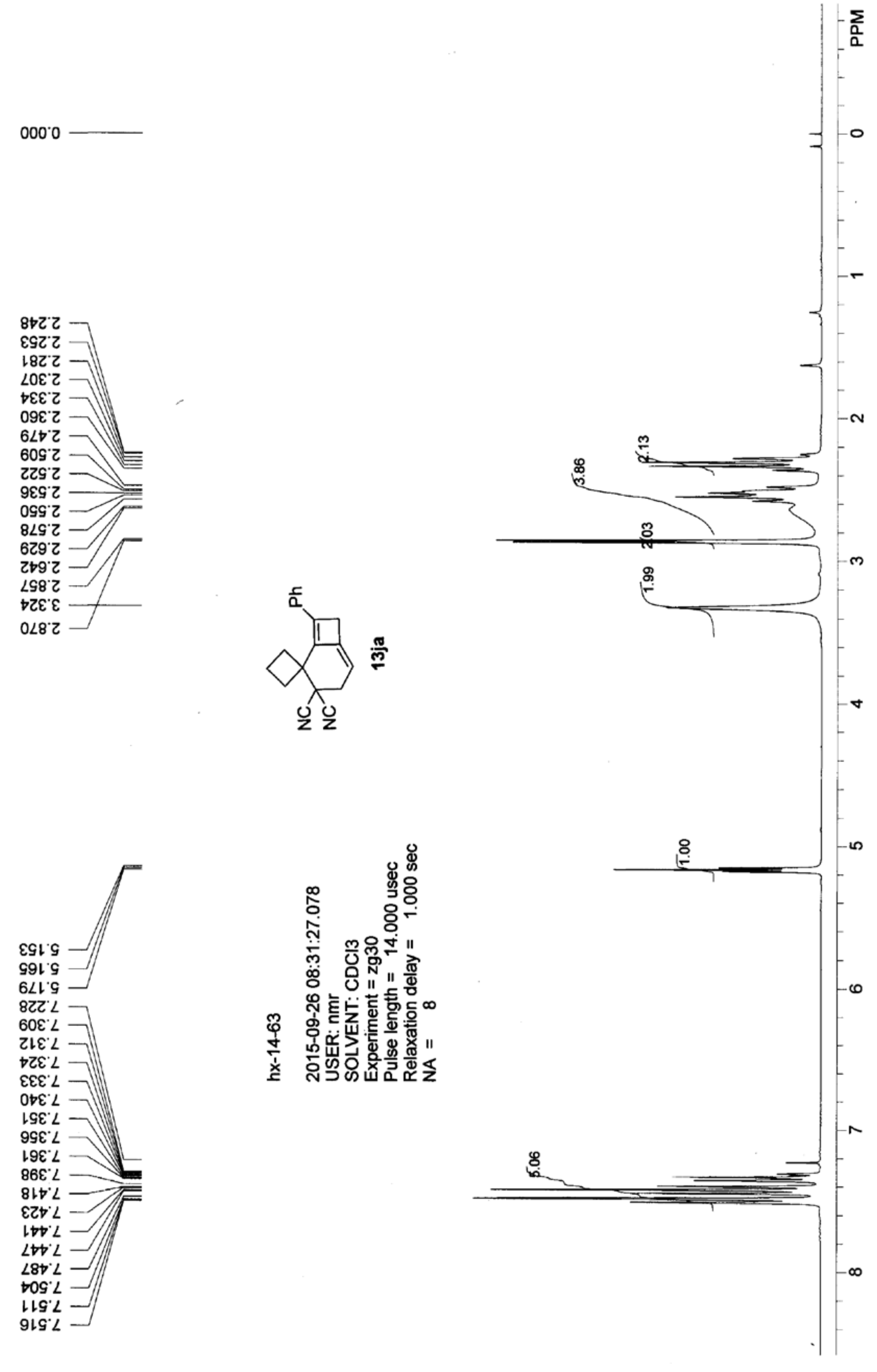

Supplementary Figure 117. ^{1}H NMR (300 MHz, $CDCl_3$) spectrum for 13ja.

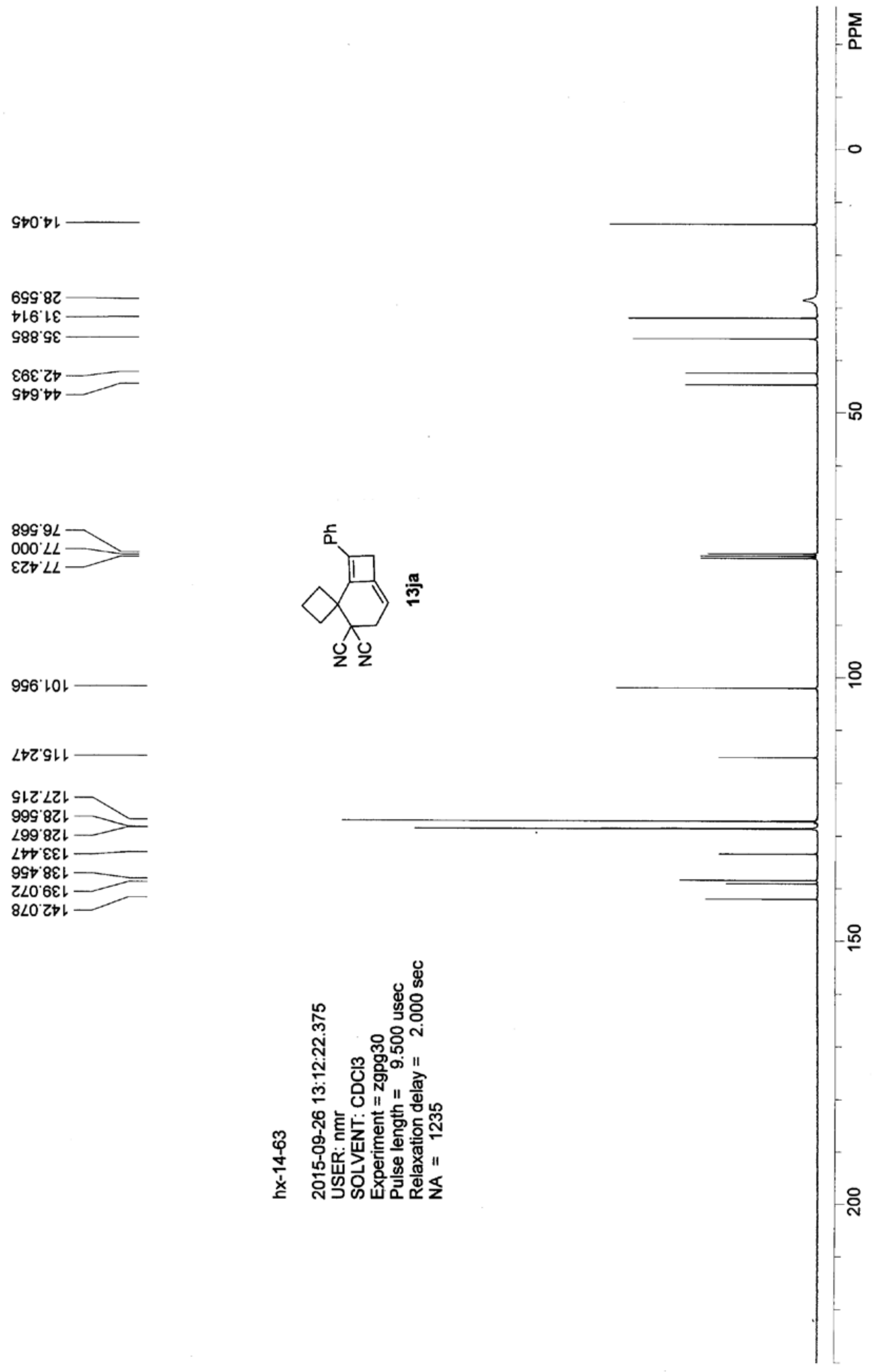

Supplementary Figure 118. ^{13}**C NMR (75 MHz, $CDCl_3$) spectrum for 13ja.**

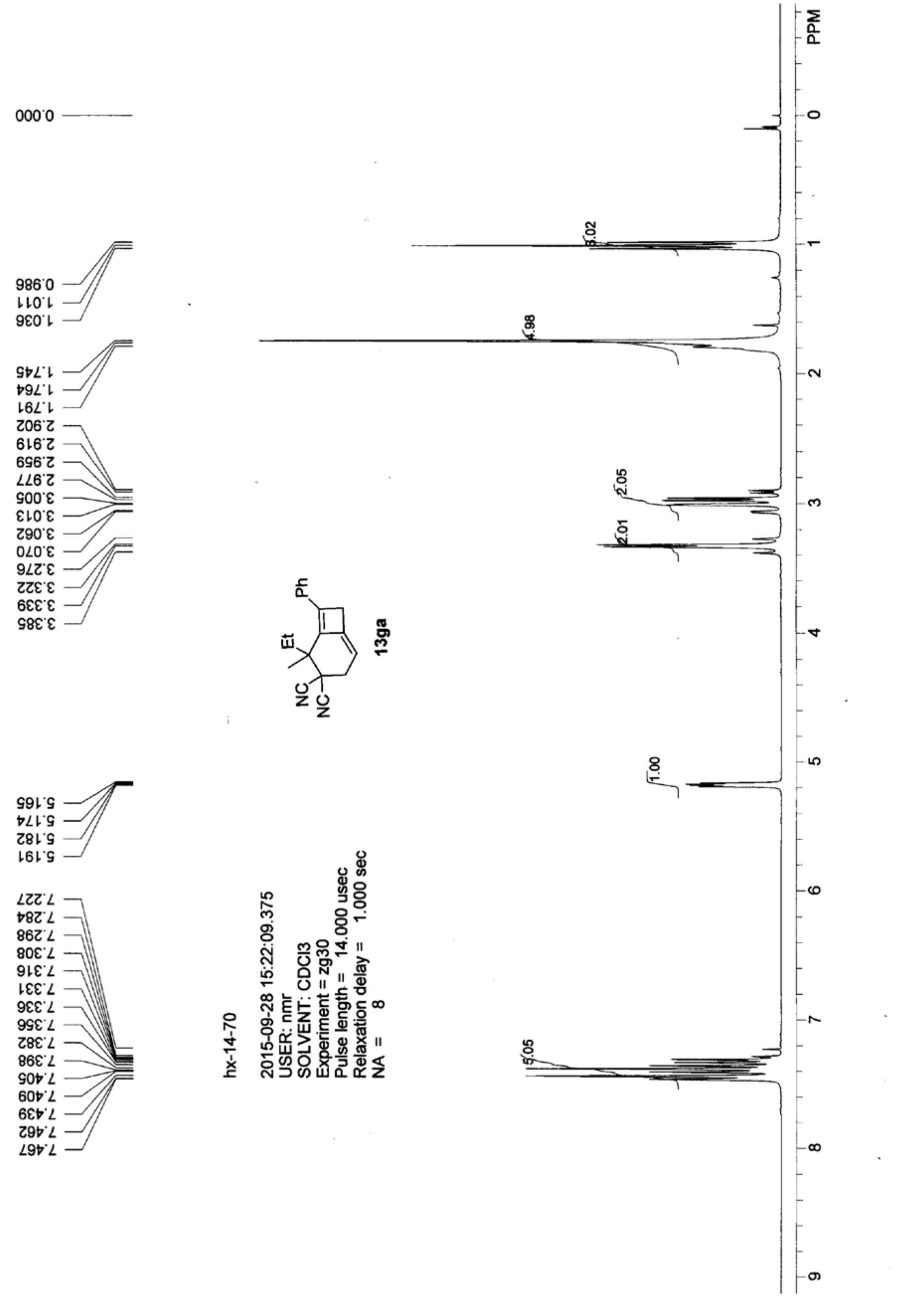

Supplementary Figure 119. ^{1}H NMR (300 MHz, $CDCl_3$) spectrum for 13ga.

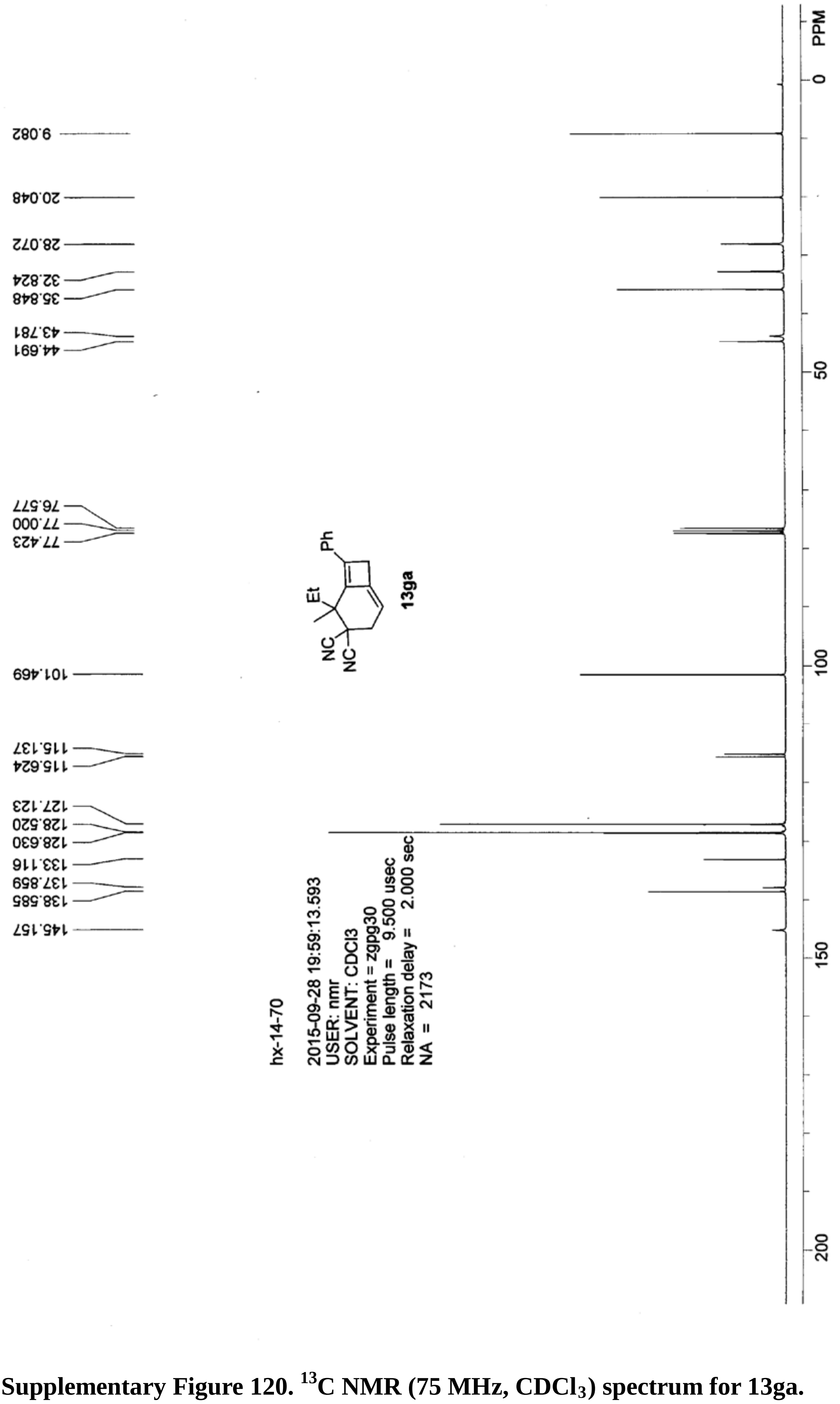

Supplementary Figure 120. ^{13}**C NMR (75 MHz, CDCl$_3$) spectrum for 13ga.**

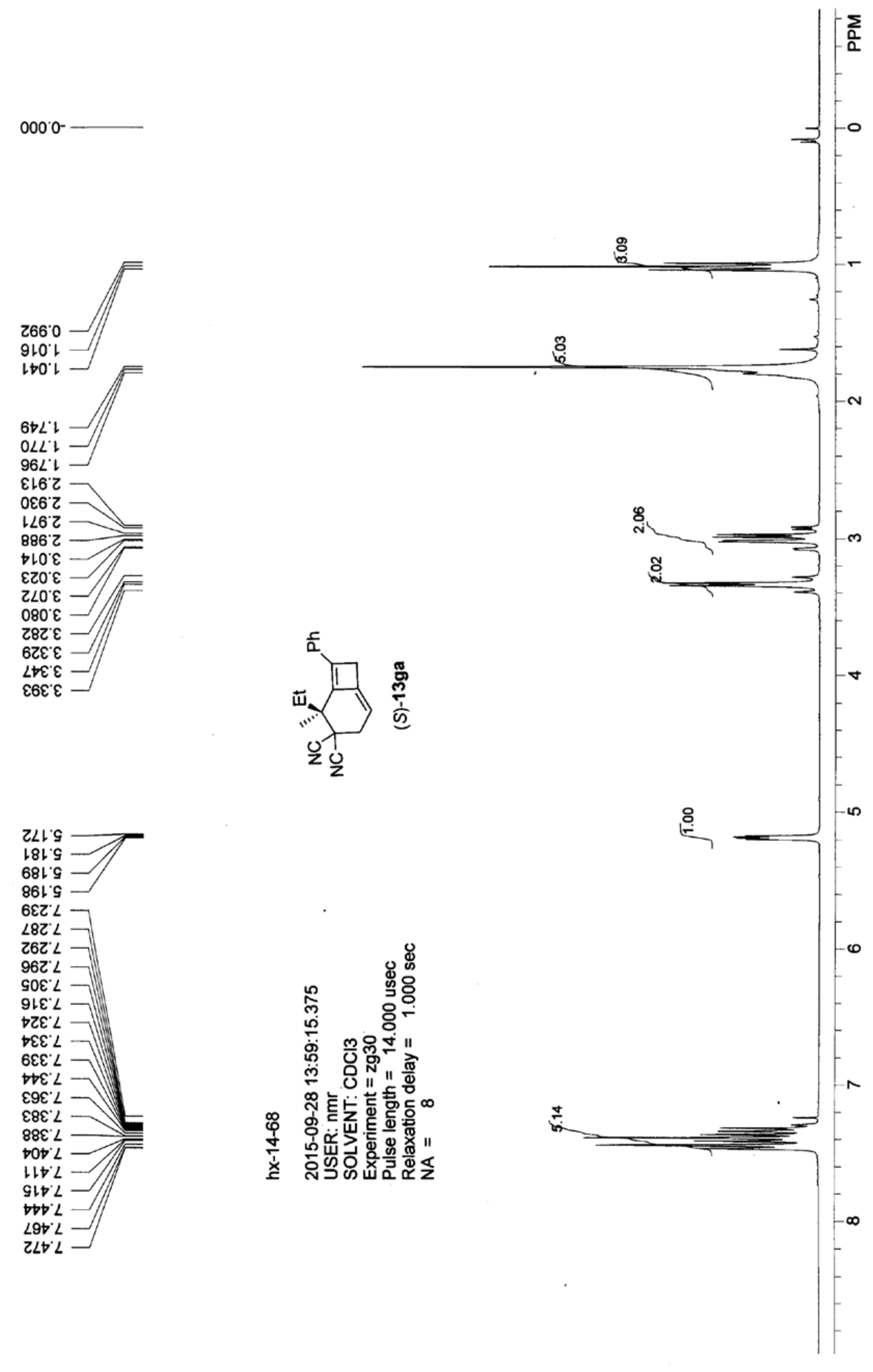

Supplementary Figure 121. **^{1}H NMR (300 MHz, $CDCl_3$) spectrum for (*S*)-13ga.**

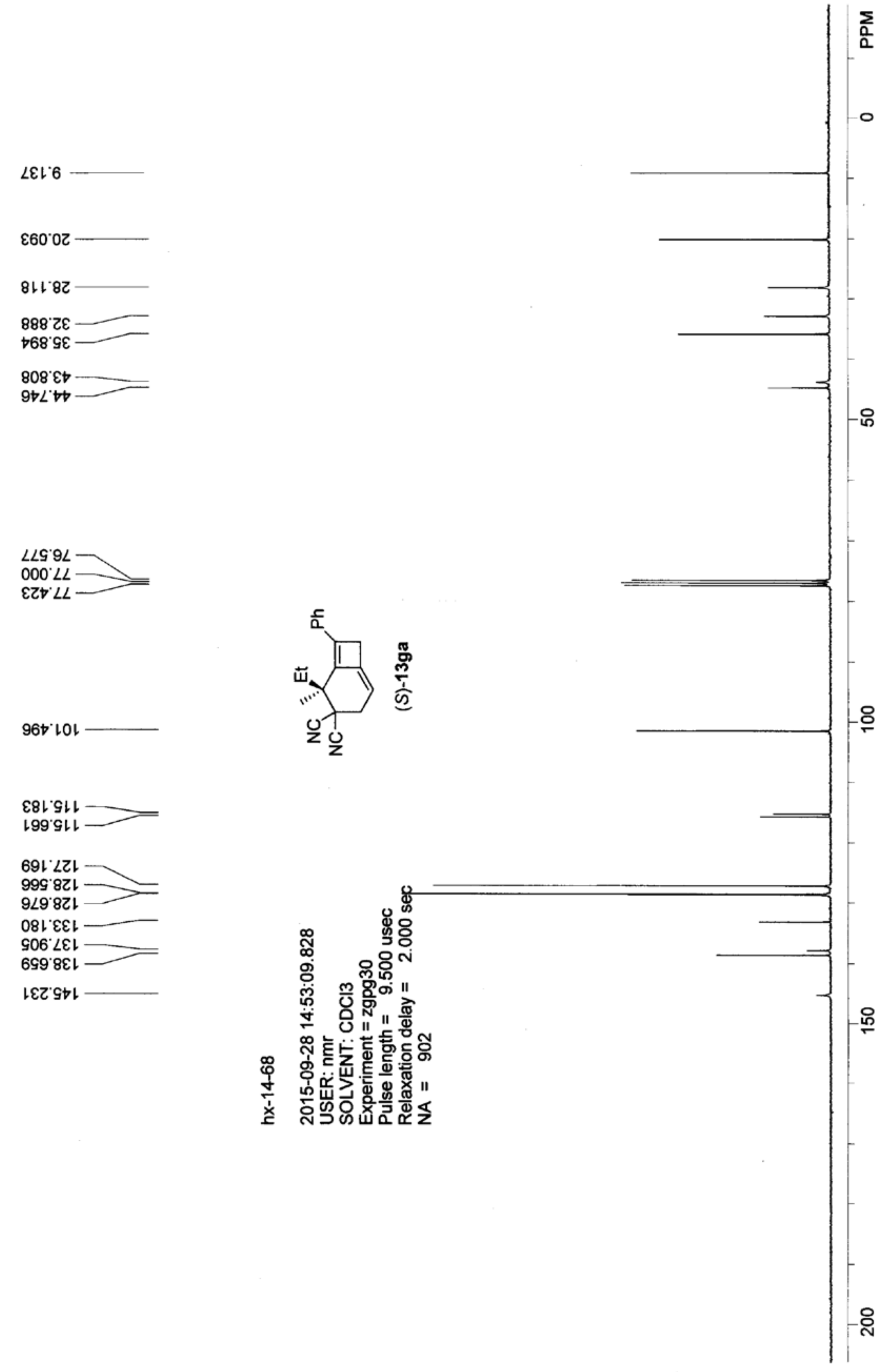

Supplementary Figure 122. **^{13}C NMR (75 MHz, $CDCl_3$) spectrum for (*S*)-13ga.**

hx-14-68

实验单位：zju
实验时间：2015-09-30, 15:07:39
谱图文件:D:\浙大智达\N2000\样品\C0044.org

实验者：hx
报告时间：2015-09-30, 16:46:01
积分方法:面积归一法

实验内容简介：
IC, n-hexane/i-PrOH = 200/1, 214 nm, 1.0 ml/min

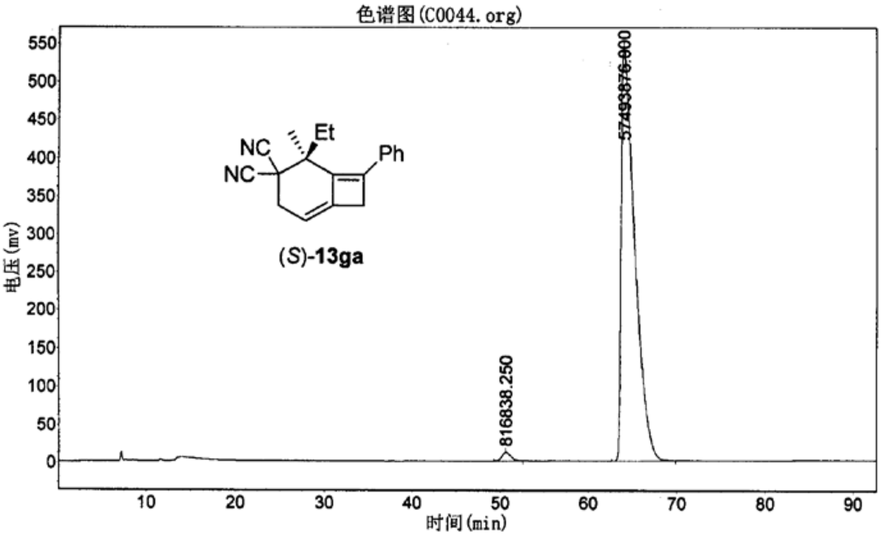

分析结果表

峰号	峰名	保留时间	峰高	峰面积	含量
1		50.637	11453.410	816838.250	1.4008
2		64.027	543515.500	57493876.000	98.5992
总计			554968.910	58310714.250	100.0000

Supplementary Figure 123. HPLC spectrum for (*S*)-13ga.

hx-14-70

实验单位：zju
实验时间：2015-09-30, 13:37:52
谱图文件:D:\浙大智达\N2000\样品\C0043.org

实验者：hx
报告时间：2015-09-30, 15:11:20
积分方法:面积归一法

实验内容简介：
IC, n-hexane/i-PrOH = 200/1, 214 nm, 1.0 ml/min

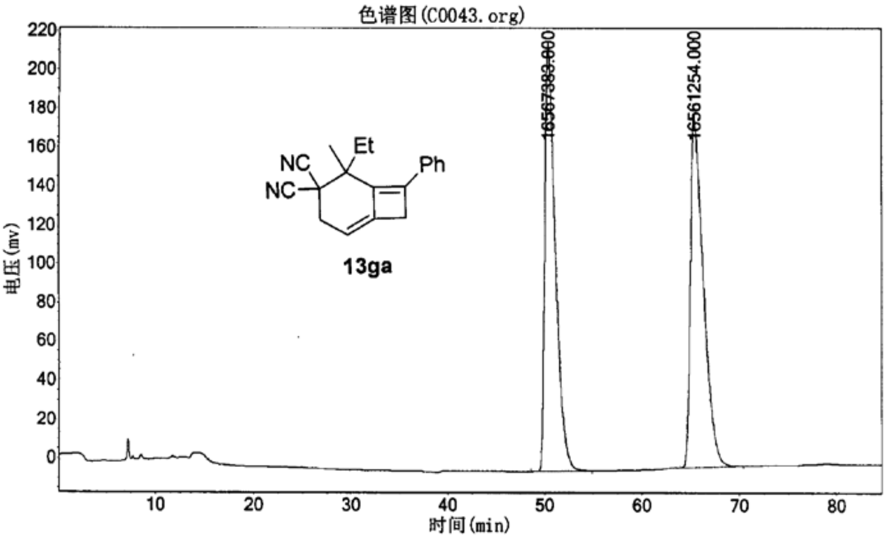

分析结果表

峰号	峰名	保留时间	峰高	峰面积	含量
1		50.315	218581.078	16567383.000	50.0093
2		65.355	179042.266	16561254.000	49.9907
总计			397623.344	33128637.000	100.0000

Supplementary Figure 124. HPLC spectrum for 13ga.

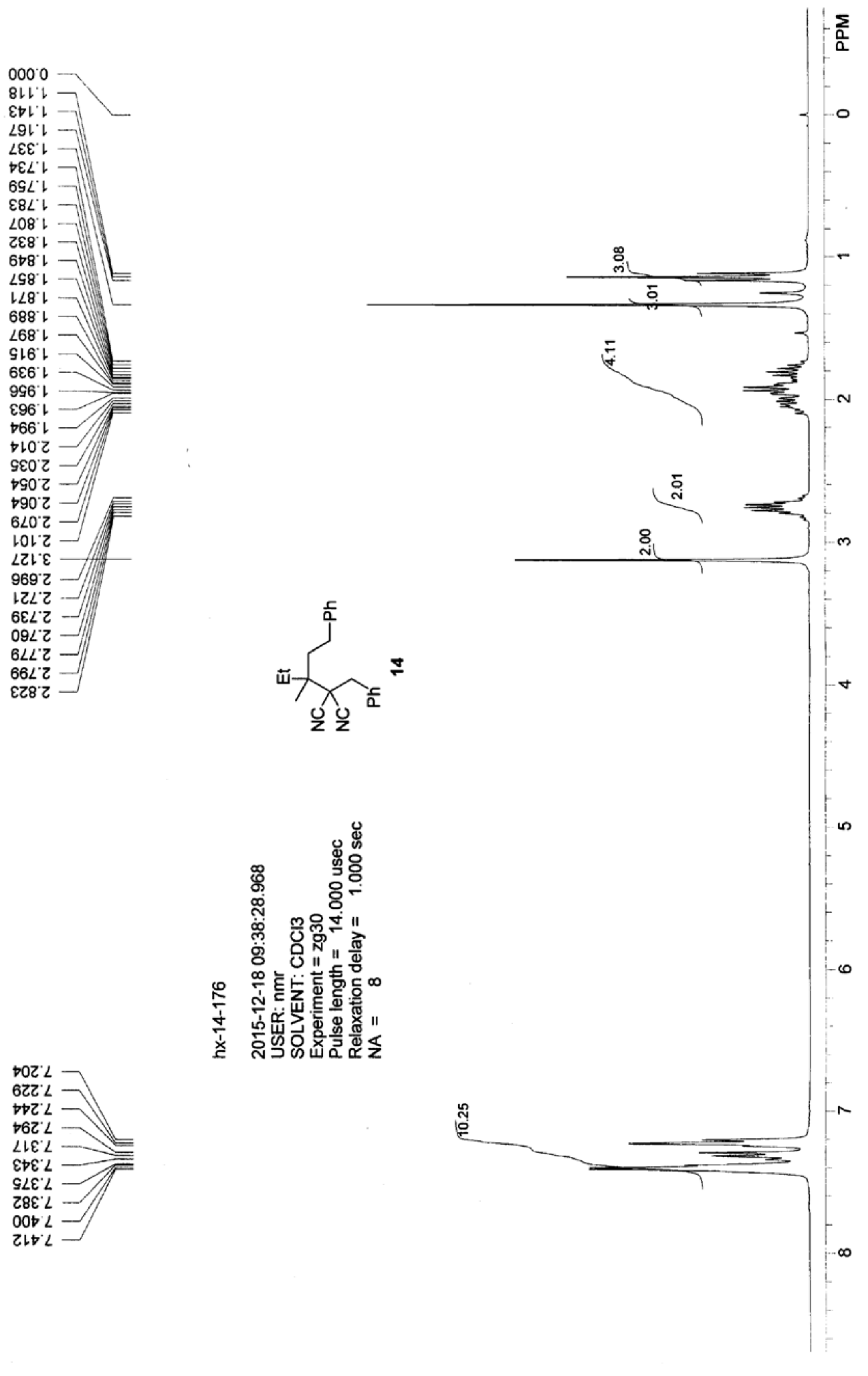

Supplementary Figure 125. ^{1}H NMR (300 MHz, $CDCl_3$) spectrum for 14.

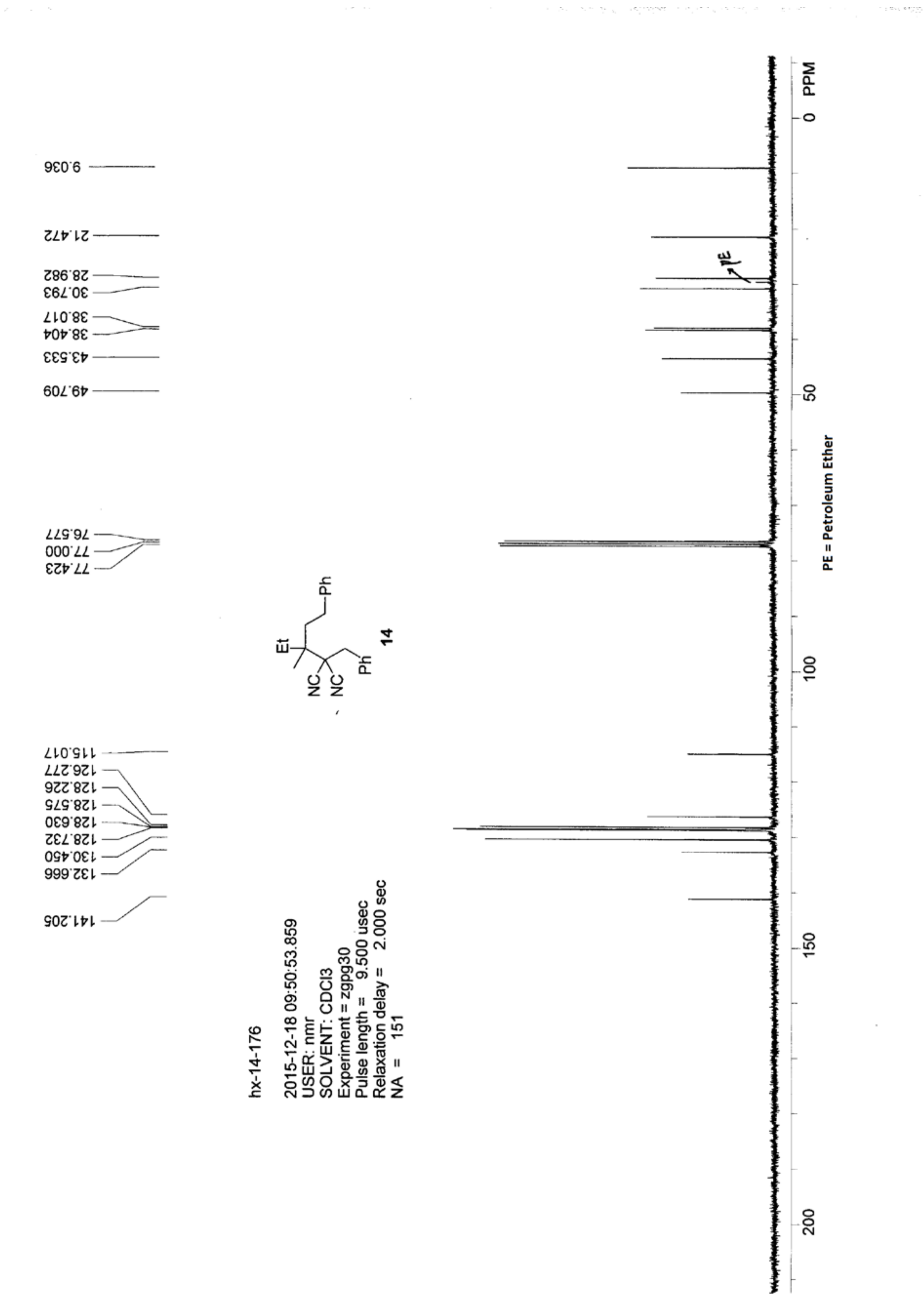

Supplementary Figure 126. ^{13}**C NMR (75 MHz,** $CDCl_3$**) spectrum for 14.**

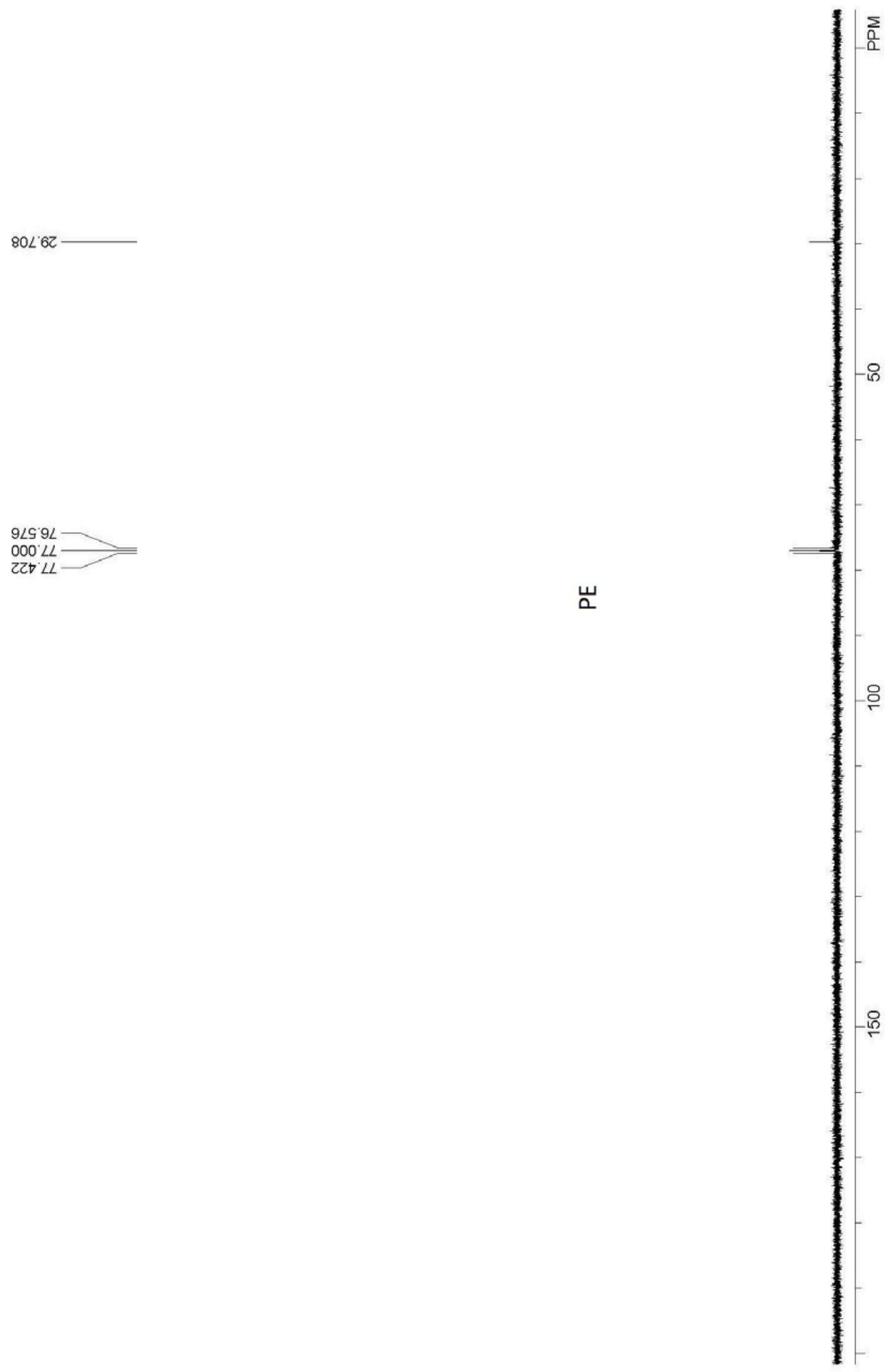

Supplementary Figure 127. ^{13}**C NMR (75 MHz, CDCl$_3$) spectrum for PE (Petroleum Ether).**

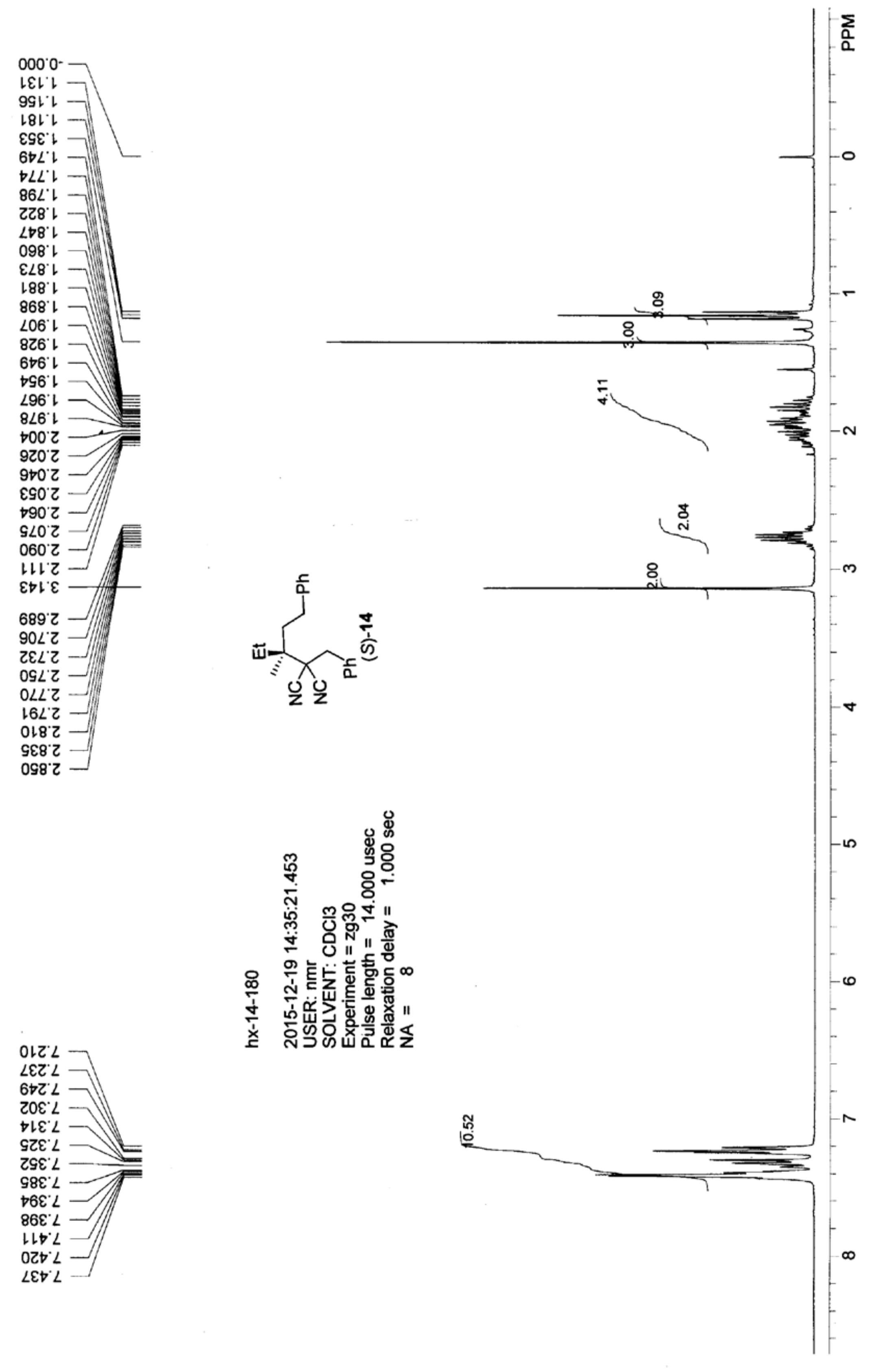

Supplementary Figure 128. ^{1}**H NMR (300 MHz, CDCl$_3$) spectrum for (*S*)-14.**

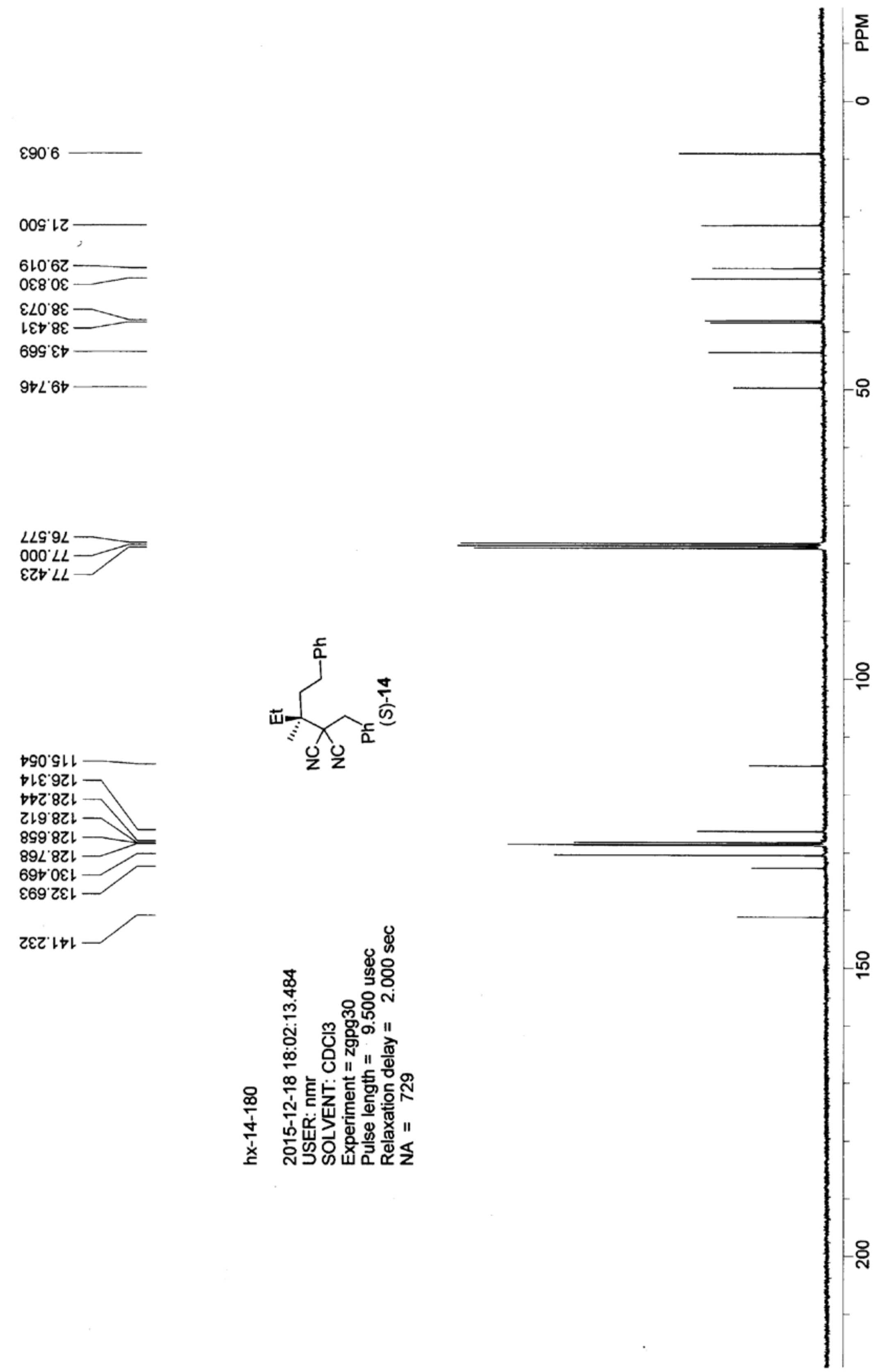

Supplementary Figure 129. ^{13}**C NMR (75 MHz, CDCl**$_3$**) spectrum for (*S*)-14.**

nple Name: hx-14-180

===

Acq. Operator : 系统
Sample Operator : 系统
Acq. Instrument : SFC   Location : Vial 25
Injection Date : 1/13/2016 3:25:38 PM
Inj Volume : 5.000 µl
Acq. Method : C:\CHEM32\1\METHODS\DEF_SFC-XL.M
Last changed : 1/13/2016 2:34:15 PM by 系统
(modified after loading)
Analysis Method : C:\CHEM32\1\METHODS\DEF_LC-TEST-2016.M
Last changed : 1/13/2016 4:09:56 PM by 系统
(modified after loading)
Additional Info : Peak(s) manually integrated

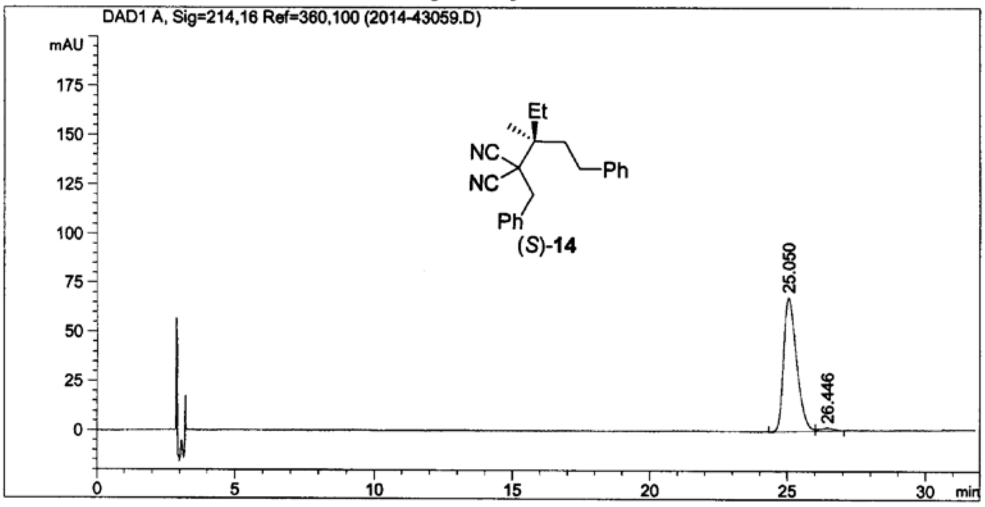

===

Area Percent Report

===

Sorted By : Signal
Multiplier : 1.0000
Dilution : 1.0000
Do not use Multiplier & Dilution Factor with ISTDs

Signal 1: DAD1 A, Sig=214,16 Ref=360,100

Peak #	RetTime [min]	Type	Width [min]	Area [mAU*s]	Height [mAU]	Area %
1	25.050	MF R	0.5457	2228.58032	67.71133	97.8158
2	26.446	FM R	0.5596	49.76266	1.48221	2.1842

Totals : 2278.34299 69.19354

===

*** End of Report ***

Supplementary Figure 130. HPLC spectrum for (*S*)-14.

mple Name: hx-14-176-rac-od-h-95-5-1.3-230

===

Acq. Operator : 系统
Sample Operator : 系统
Acq. Instrument : SFC   Location : Vial 9
Injection Date : 1/13/2016 2:46:21 PM
                                                        Inj Volume : 5.000 µl
Acq. Method : C:\CHEM32\1\METHODS\DEF_SFC-XL.M
Last changed : 1/13/2016 2:34:15 PM by 系统
(modified after loading)
Analysis Method : C:\CHEM32\1\METHODS\DEF_LC-TEST-2016.M
Last changed : 1/13/2016 4:08:17 PM by 系统
(modified after loading)
Additional Info : Peak(s) manually integrated

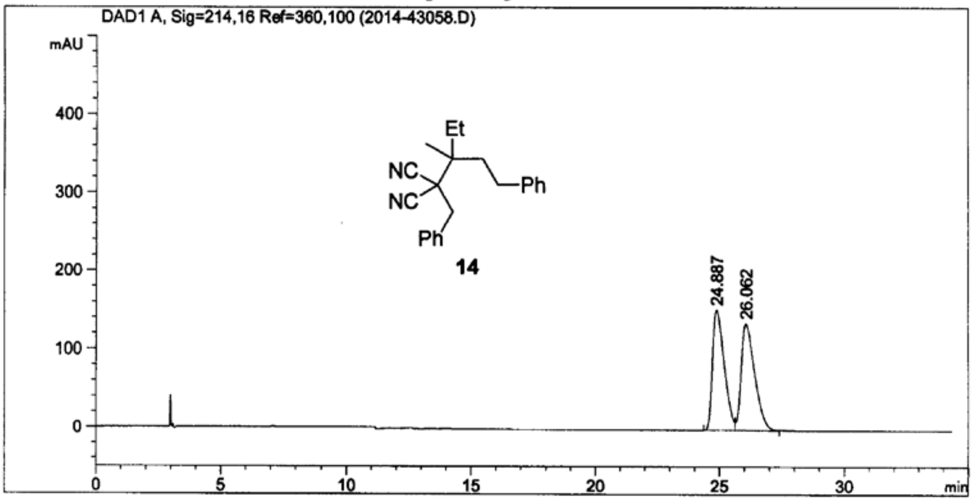

===

Area Percent Report

===

Sorted By : Signal
Multiplier : 1.0000
Dilution : 1.0000
Do not use Multiplier & Dilution Factor with ISTDs

Signal 1: DAD1 A, Sig=214,16 Ref=360,100

Peak #	RetTime [min]	Type	Width [min]	Area [mAU*s]	Height [mAU]	Area %
1	24.887	BV	0.4777	5052.77686	153.62276	49.4922
2	26.062	VV	0.5153	5156.47217	135.69583	50.5078

Totals : 1.02092e4 289.31859

===

*** End of Report ***

Supplementary Figure 131. HPLC spectrum for 14.

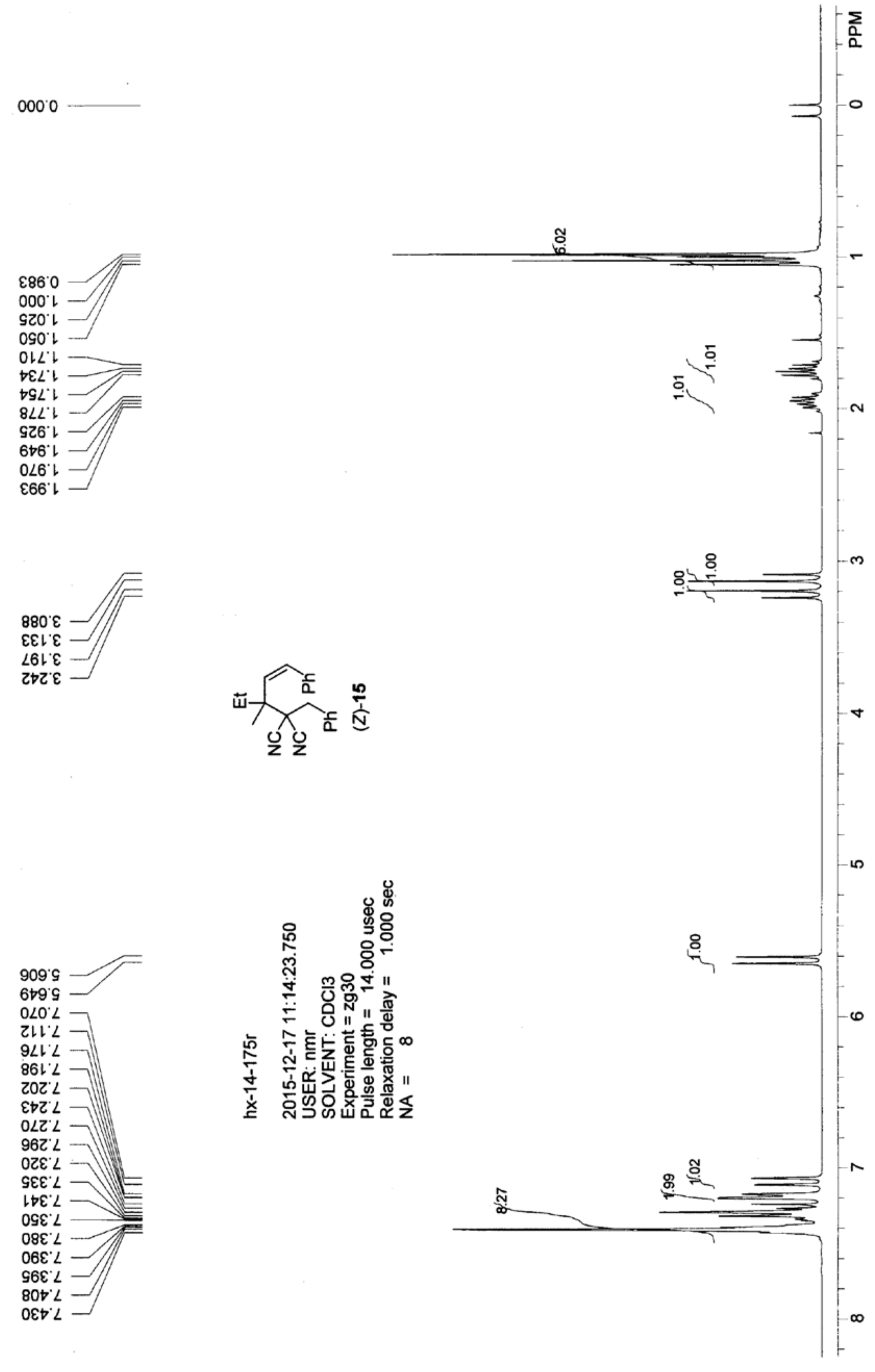

Supplementary Figure 132. ^{1}**H NMR (300 MHz,** $CDCl_3$**) spectrum for (*Z*)-15.**

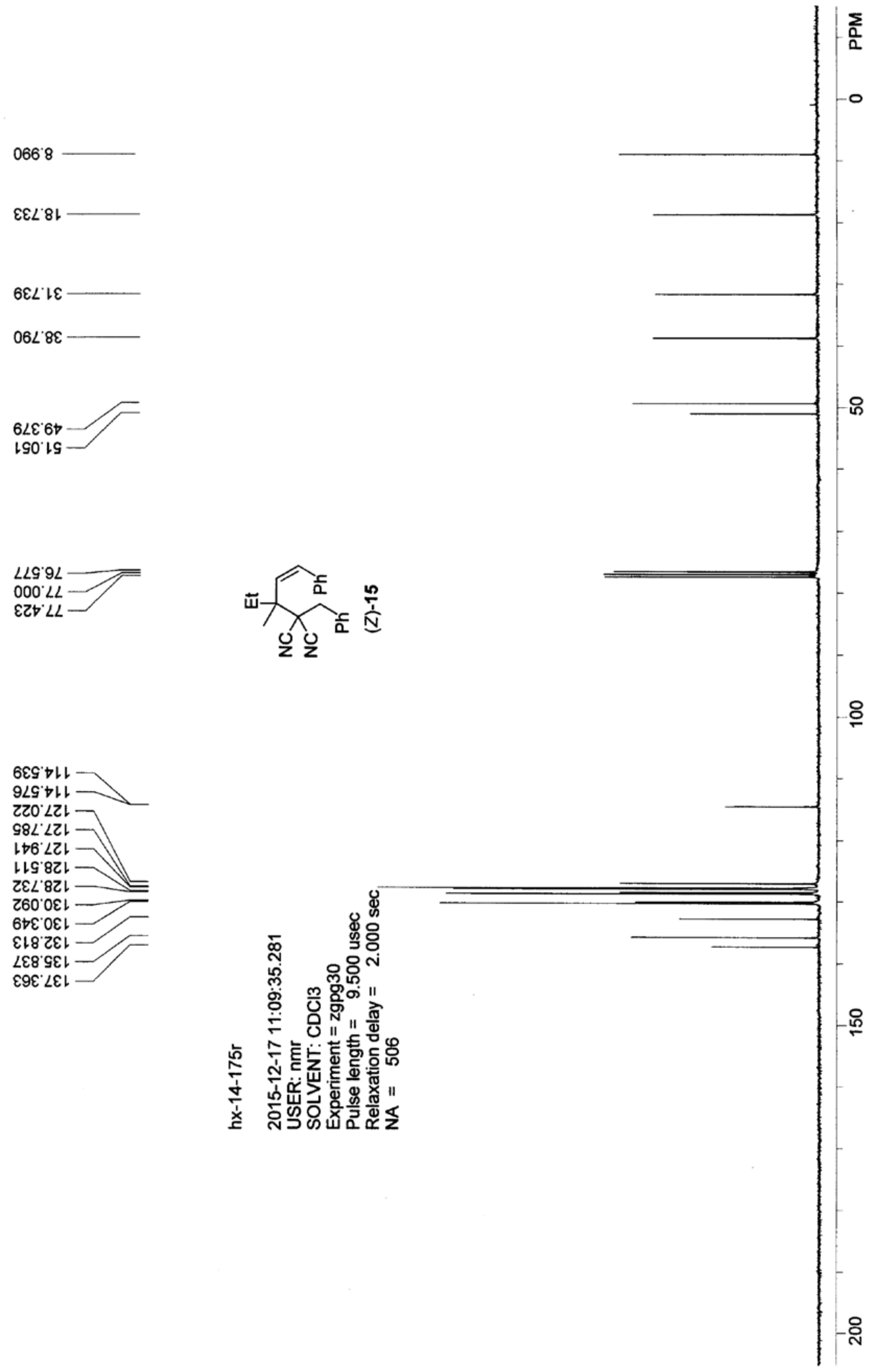

Supplementary Figure 133. ^{13}**C NMR (75 MHz, CDCl$_3$) spectrum for (*Z*)-15.**

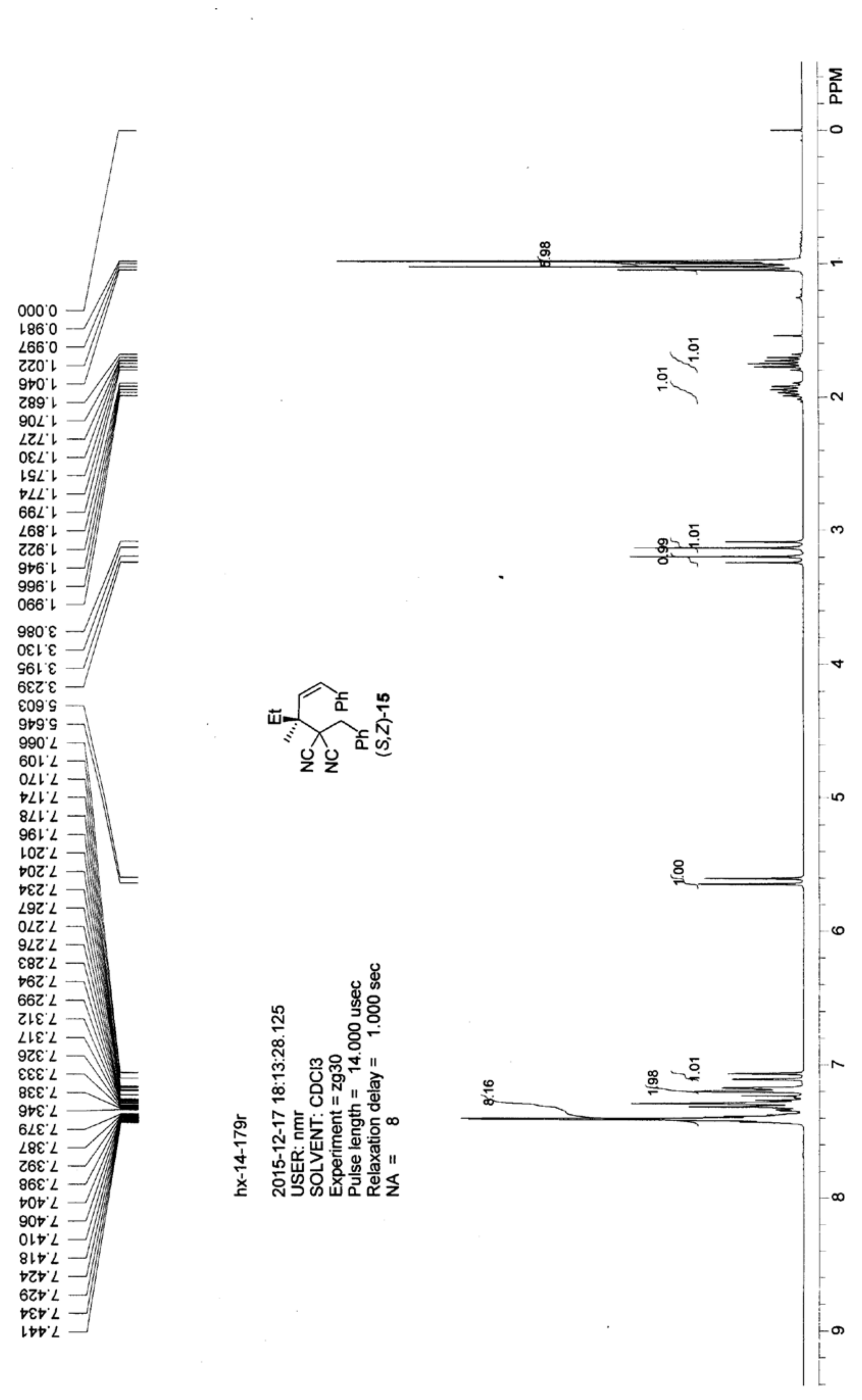

Supplementary Figure 134. ^{1}**H NMR (300 MHz, CDCl$_3$) spectrum for (*S*,*Z*)-15.**

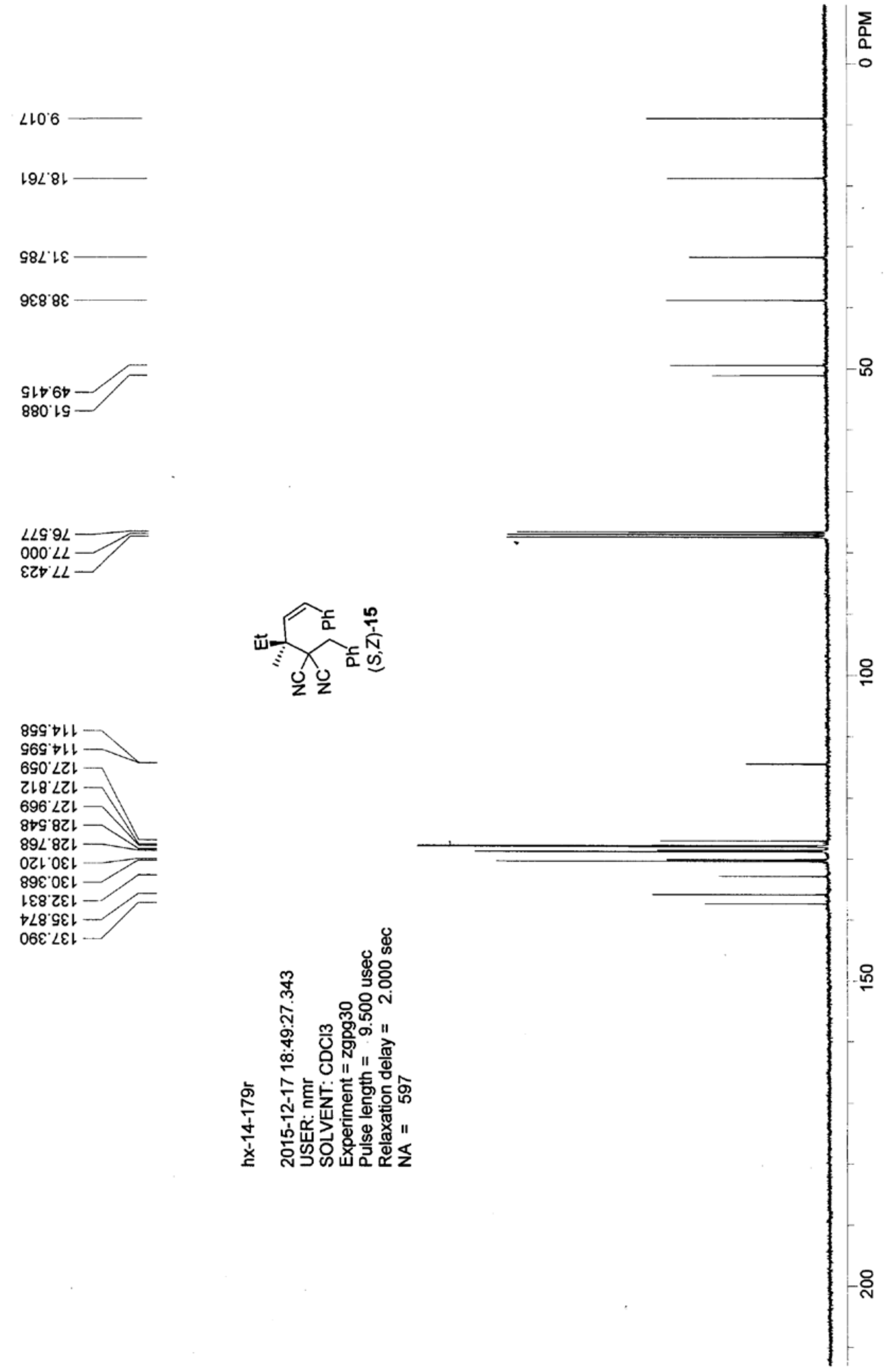

Supplementary Figure 135. **^{13}C NMR (75 MHz, $CDCl_3$) spectrum for (*S*,*Z*)-15.**

hx-14-179

实验单位：zju
实验时间：2015-12-26, 16:06:59
谱图文件:D:\浙大智达\N2000\样品\D0002.org
方法文件:D:\浙大智达\N2000\djx.mtd

实验者：hx
报告时间：2015-12-26, 17:01:34
积分方法:面积归一法

实验内容简介：
AD-H, n-hexane/i-PrOH = 200/1, 214 nm, 0.6 ml/min

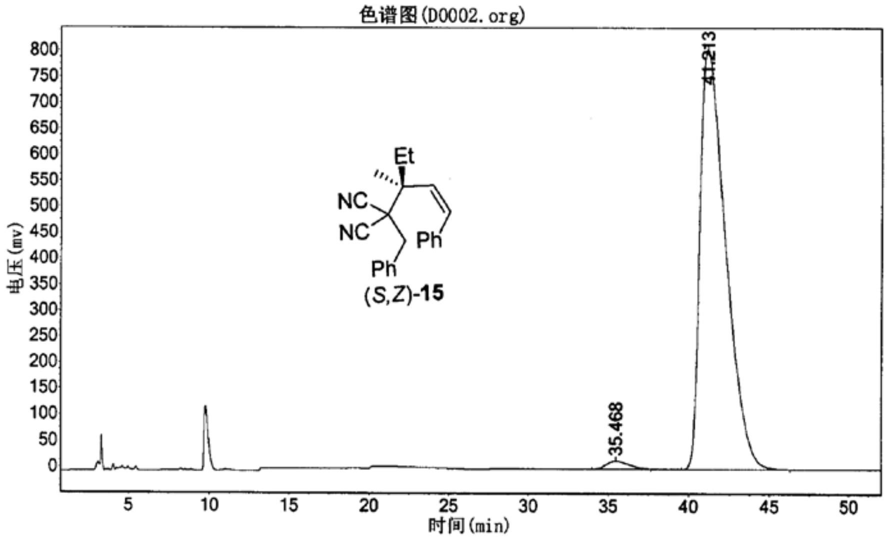

分析结果表

峰号	峰名	保留时间	峰高	峰面积	含量
1		35.468	16106.255	1549522.500	1.7042
2		41.213	805293.500	89373600.000	98.2958
总计			821399.755	90923122.500	100.0000

Supplementary Figure 136. HPLC spectrum for (*S*,*Z*)-15.

hx-14-175

实验单位：zju
实验时间：2015-12-26, 15:13:52
谱图文件:D:\浙大智达\N2000\样品\D0001.org
方法文件:D:\浙大智达\N2000\djx.mtd

实验者：hx
报告时间：2015-12-26, 16:04:26
积分方法:面积归一法

实验内容简介：
AD-H, n-hexane/i-PrOH = 200/1, 214 nm, 0.6 ml/min

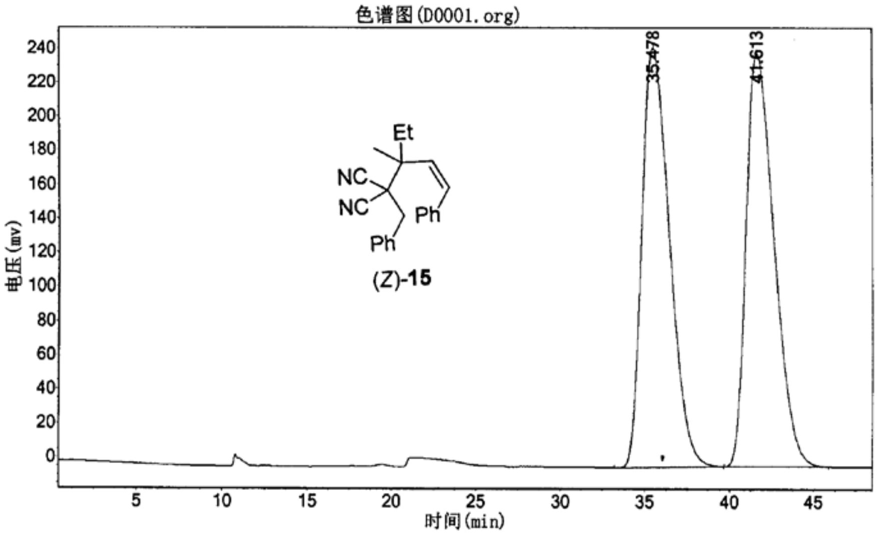

分析结果表

峰号	峰名	保留时间	峰高	峰面积	含量
1		35.478	245625.563	27896166.000	49.9972
2		41.613	241994.531	27899274.000	50.0028
总计			487620.094	55795440.000	100.0000

Supplementary Figure 137. HPLC spectrum for (*Z*)-15.

Supplementary methods

Synthesis of new tertiary propargylic carbonates 1.

1. Preparation of 4-(4-Chlorophenyl)-2-methylbut-3-yn-2-yl methyl carbonate **1b**.[1]

   hx-14-192, lpb-11-78

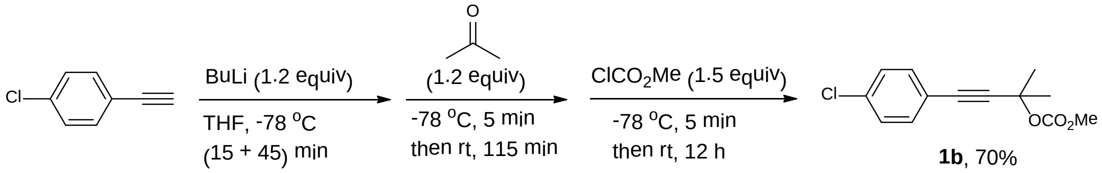

1b, 70%

Typical Procedure I: To a solution of 1-chloro-4-ethynylbenzene (2.7325 g, 20 mmol) in THF (80 mL) was added dropwise *n*-BuLi (2.5 M in hexane, 9.6 mL, 24 mmol) at -78 °C in 15 minutes. After lithiation for 45 min at -78 °C, acetone (1.8 mL, d = 0.80 g/mL, 1.44 g, 24 mmol) was added dropwise at this temperature in 5 minutes. The reaction mixture was warmed up to room temperature and stirred for 115 min. Then methyl chloroformate (2.3 mL, d = 1.22 g/mL, 2.81 g, 30 mmol) was added dropwise at -78 °C in 5 minutes. The mixture was warmed to room temperature, stirred for 12 h as monitored by TLC (eluent: petroleum ether/ethyl acetate = 20/1), and quenched with a saturated aqueous NH_4Cl solution (40 mL). The resulting mixture was extracted with ethyl acetate (50 mL × 2), and the combined organic phase was dried over anhydrous Na_2SO_4. After filtration, evaporation of the solvent and chromatography on silica gel (eluent: petroleum ether/ethyl acetate = 20/1) afforded **1b** (3.5278 g, 70%) as a liquid: ^{1}H NMR (300 MHz, $CDCl_3$) δ 7.40-7.33 (m, 2 H, ArH), 7.30-7.23 (m, 2 H, ArH), 3.78 (s, 3 H, Me), 1.78 (s, 6 H, Me × 2); ^{13}C NMR (75 MHz, $CDCl_3$) δ 153.4, 134.5, 133.0, 128.5, 120.8, 90.3, 83.3, 74.5, 54.3, 28.8; IR

(neat, cm^{-1}) 2990, 2955, 2851, 2227, 1755, 1593, 1489, 1468, 1440, 1398, 1383, 1365, 1266, 1190, 1132, 1090, 1015; MS (EI): *m/z* (%) 254 ($M^+(^{37}Cl)$, 11.45), 252 ($M^+(^{35}Cl)$, 34.61), 141 (100). HRMS Calcd. for $C_{13}H_{13}{}^{35}ClO_3$ ($(M(^{35}Cl)^+)$): 252.0553. Found 252.0551.

2. Preparation of Methyl (2-methyl-4-(*m*-tolyl)but-3-yn-2-yl) carbonate **1c**.[2]

   lpb-11-160

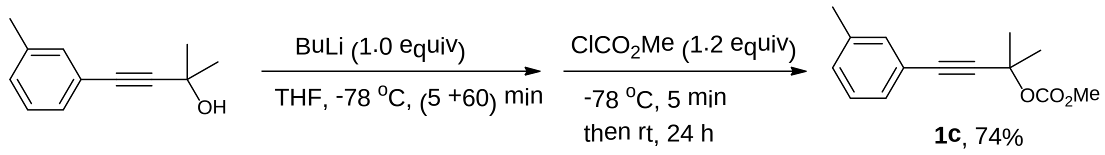

To a solution of 2-methyl-4-(*m*-tolyl)but-3-yn-2-ol (2.5322 g, 14.6 mmol) in THF (40 mL) was added dropwise *n*-BuLi (2.5 M in hexane, 5.6 mL, 14 mmol) at -78 °C within 5 minutes. After lithiation for 1.0 h at -78 °C, $ClCO_2Me$ (1.3 mL, *d* = 1.22 g/mL, 1.59 g, 16.8 mmol) was added dropwise at this temperature within 5 minutes. Then the cooling bath was removed and the reaction mixture was then allowed to warm up to room temperature gradually with stirring for 24 h and quenched with water (20 mL). The mixture was extracted with ethyl ether (20 mL × 2), and the combined organic phase was washed by brine and dried over anhydrous Na_2SO_4. After filtration, evaporation of the solvent and chromatography on silica gel (eluent: petroleum ether/ethyl acetate = 30/1) afforded **1c** (2.5126 g, 74%) as a liquid: ^1H NMR (300 MHz, $CDCl_3$) δ 7.31-7.08 (m, 4 H, ArH), 3.77 (s, 3 H, Me), 2.31 (s, 3 H, Me), 1.79 (s, 6 H, Me × 2); ^{13}C NMR (75 MHz, $CDCl_3$) δ 153.5, 137.8, 132.4, 129.3, 128.9, 128.0, 122.1, 88.9, 84.6, 74.7, 54.3, 28.9, 21.1. IR (neat, cm^{-1}) 2990, 2955, 2851, 2228, 1755, 1601, 1582, 1485, 1440, 1383, 1365, 1267, 1224, 1194, 1159, 1132,

1098; MS (EI): *m/z* (%) 232 (M^+, 39.10), 157 (100). HRMS Calcd. for $C_{14}H_{16}O_3$ (M^+): 232.1099. Found 232.1096.

3. Preparation of methyl (3-methyl-1-phenylhept-1-yn-3-yl) carbonate **1h**. wsz-6-123

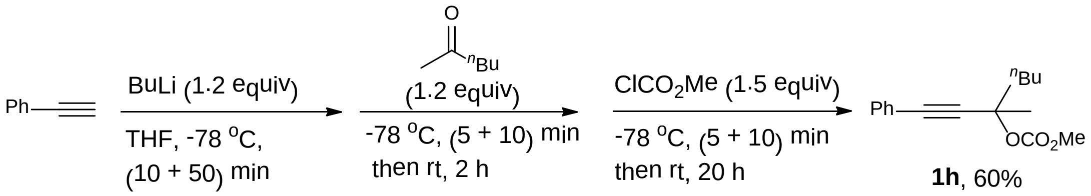

Following **Typical Procedure I**, the reaction of ethynylbenzene (3.3 mL, d = 0.93 g/mL, 3.07 g, 30 mmol), *n*-BuLi (2.5 M in hexane, 14.4 mL, 36.0 mmol), hexan-2-one (4.5 mL, d = 0.81 g/mL, 3.65 g, 36.0 mmol), and $ClCO_2Me$ (3.5 mL, d = 1.22 g/mL, 4.27 g, 45 mmol) in THF (100 mL) afforded **1h** (4.6948 g, 60%) as a liquid (eluent: petroleum ether/ethyl acetate = 100/1 to 70/1): ^1H NMR (300 MHz, $CDCl_3$) δ 7.48-7.40 (m, 2 H, ArH), 7.33-7.24 (m, 3 H, ArH), 3.76 (s, 3 H, Me), 2.12-1.98 (m, 1 H, one proton of CH_2), 1.97-1.84 (m, 1 H, one proton of CH_2), 1.79 (s, 3 H, Me), 1.65-1.45 (m, 2 H, CH_2), 1.45-1.30 (m, 2 H, CH_2), 0.94 (t, *J* = 7.2 Hz, 3 H, Me); ^{13}C NMR (75 MHz, $CDCl_3$) δ 153.4, 131.8, 128.4, 128.1, 122.4, 88.6, 85.4, 77.9, 54.2, 41.3, 26.4, 22.6, 13.9; IR (neat, cm^{-1}) 3051, 2957, 2935, 2872, 2233, 1755, 1602, 1490, 1441, 1375, 1319, 1267, 1185, 1149, 1077, 1040; MS (EI): *m/z* (%) 260 (M^+, 0.07), 184 ($(M-HOCO_2Me)^+$, 20.14), 142 (100); HRMS Calcd. for $C_{16}H_{20}O_3$ (M^+): 260.1412; Found: 260.1415.

4. Preparation of methyl 1-(phenylethynyl)cyclobutyl carbonate **1j**. wwt-3-3

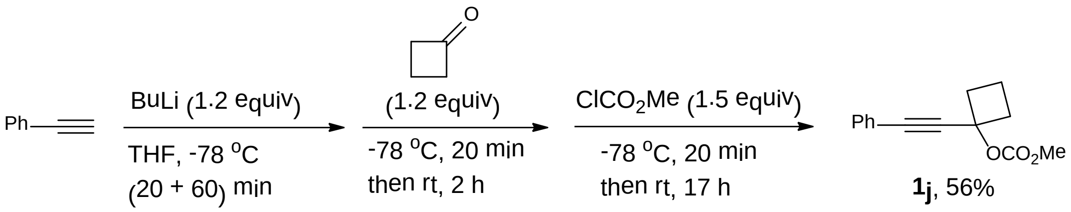

Following **Typical Procedure I**, the reaction of ethynylbenzene (1.1 mL, d = 0.93 g/ mL, 1.02 g, 10 mmol), *n*-BuLi (2.5 M in hexane, 4.8 mL, 12 mmol), cyclobutanone (0.9 mL, d = 0.94 g/mL, 0.85 g, 12 mmol), and $ClCO_2Me$ (1.2 mL, d = 1.22 g/mL, 1.46 g, 15 mmol) in THF (30 mL) afforded **1j** (1.2874 g, 56%) as a liquid (eluent: petroleum ether/ethyl acetate = 30/1): ^{1}H NMR (300 MHz, $CDCl_3$) δ 7.51-7.41 (m, 2 H, ArH), 7.35-7.26 (m, 3 H, ArH), 3.79 (s, 3 H, Me), 2.74-2.46 (m, 4 H, CH_2 × 2), 2.10-1.86 (m, 2 H, CH_2); ^{13}C NMR (75 MHz, $CDCl_3$) δ 153.5, 131.9, 128.4, 128.1, 122.4, 88.5, 84.7, 74.2, 54.5, 36.5, 14.0; IR (neat, cm^{-1}) 3054, 3002, 2955, 2872, 2849, 2236, 1755, 1598, 1492, 1442, 1313, 1275, 1246, 1212, 1152, 1118, 1065, 1027; MS (EI): *m/z* (%) 230 (M$^+$, 4.27), 128 (100); Anal. Calcd. for $C_{14}H_{14}O_3$ (%): C 73.03, H 6.13; Found: C 73.02, H 6.05.

5. Preparation of 4-(4-methoxyphenyl)-2-methylbut-3-yn-2-yl methyl carbonate **1l**. lpb-11-67

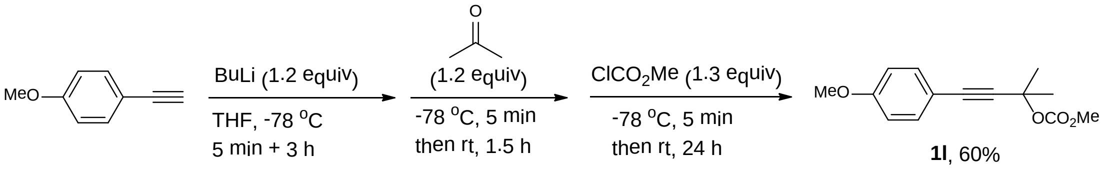

Following **Typical Procedure I**, the reaction of 1-ethynyl-4-methoxybenzene (3.3435 g, 25.3 mmol), *n*-BuLi (2.5 M in hexane, 12 mL, 30 mmol), acetone (2.2 mL, d = 0.79 g/mL, 1.74 g, 30 mmol), $ClCO_2Me$ (2.5 mL, d = 1.22 g/mL, 3.05 g, 32.3 mmol) in THF (100 mL) afforded **1l** (3.7837 g, 60%) as a liquid (eluent: petroleum

ether/ethyl acetate = 100/1~40/1): ^1H NMR (300 MHz, $CDCl_3$) δ 7.41-7.35 (m, 2 H, ArH), 6.85-6.78 (m, 2 H, ArH), 3.80 (s, 3 H, Me), 3.77 (s, 3 H, Me), 1.79 (s, 6 H, Me × 2); ^{13}C NMR (75 MHz, $CDCl_3$) δ 159.7, 153.5, 133.4, 114.4, 113.8, 88.0, 84.4, 74.9, 55.2, 54.3, 29.0; IR (neat, cm^{-1}) 2991, 2956, 2839, 2226, 1754, 1607, 1570, 1510, 1441, 1383, 1365, 1270, 1192, 1171, 1130, 1106, 1031; MS (EI): *m/z* (%) 248 (M^+, 60.38), 172 (100); HRMS Calcd. for $C_{14}H_{16}O_4$ (M^+): 248.1049. Found 248.1049.

6. Preparation of methyl (2-methyl-4-(thiophen-2-yl)but-3-yn-2-yl) carbonate **1n**. wwt-1-51

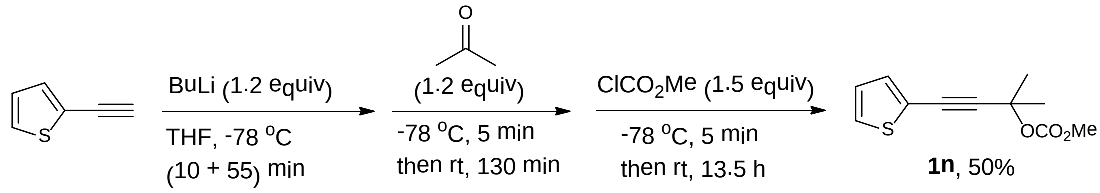

Following **Typical Procedure I**, the reaction of 2-ethynylthiophene (2.1704 g, 20 mmol), *n*-BuLi (2.5 M in hexane, 9.6 mL, 24 mmol), acetone (1.8 mL, d = 0.79 g/mL, 1.42 g, 24 mmol), $ClCO_2Me$ (2.3 mL, d = 1.22 g/mL, 2.81 g, 30 mmol) in THF (80 mL) afforded **1n** (2.2374 g, 50%) as a liquid (eluent: petroleum ether/ethyl acetate = 50/1): ^1H NMR (300 MHz, $CDCl_3$) δ 7.28-7.21 (m, 2 H, ArH), 6.96 (dd, J_1 = 5.1 Hz, J_2 = 3.6 Hz, 1 H, ArH), 3.78 (s, 3 H, Me), 1.79 (s, 6 H, Me × 2); ^{13}C NMR (75 MHz, $CDCl_3$) δ 153.5, 138.5, 131.7, 128.9, 119.3, 88.6, 84.5, 74.8, 54.2, 28.9, 21.4; IR (neat, cm^{-1}) 3108, 2990, 2955, 2849, 2228, 1755, 1440, 1384, 1365, 1279, 1265, 1222, 1192, 1098, 1042; MS (EI): *m/z* (%) 224 (M^+, 9.13), 148 (100); HRMS Calcd. for $C_{11}H_{12}O_3S$ (M^+): 224.0507; Found: 224.0511.

7. Preparation of methyl 3-methyl-1-phenyloct-1-yn-3-yl carbonate **1o**. wwt-3-4

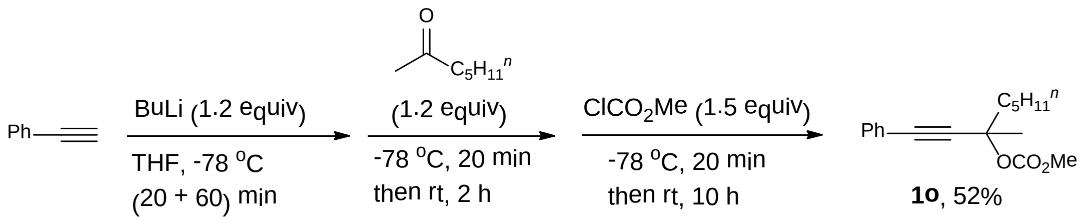

Following **Typical Procedure I**, the reaction of ethynylbenzene (1.1 mL, d = 0.93 g/ mL, 1.02 g, 10 mmol), *n*-BuLi (2.5 M in hexane, 4.8 mL, 12 mmol), heptan-2-one (1.7 mL, d = 0.83 g/mL, 1.41 g, 12 mmol), $ClCO_2Me$ (1.2 mL, d = 1.22 g/mL, 1.46 g, 15 mmol) in THF (30 mL) afforded **1o** (1.3701 g, 52%) as a liquid (eluent: petroleum ether/ethyl acetate = 30/1): ^{1}H NMR (300 MHz, $CDCl_3$) δ 7.47-7.40 (m, 2 H, ArH), 7.34-7.25 (m, 3 H, ArH), 3.76 (s, 3 H, Me), 2.11-1.97 (m, 1 H, one proton of CH_2), 1.96-1.83 (m, 1 H, one proton of CH_2), 1.79 (s, 3 H, Me), 1.68-1.46 (m, 2 H, CH_2), 1.43-1.25 (m, 4 H, CH_2 × 2), 0.91 (t, J = 6.9 Hz, 3 H, Me); ^{13}C NMR (75 MHz, $CDCl_3$) δ 153.5, 131.9, 128.5, 128.2, 122.5, 88.8, 85.5, 78.0, 54.2, 41.5, 31.7, 26.4, 23.9, 22.5, 14.0; IR (neat, cm^{-1}) 2955, 2829, 2870, 2237, 1755, 1599, 1569, 1491, 1441, 1376, 1266, 1212, 1179, 1150, 1081, 1042; MS (EI): *m/z* (%) 274 (M^+, 0.05), 142 (100); Anal. Calcd. for $C_{17}H_{22}O_3$ (%): C 74.42, H 8.08; Found: C 74.32, H 8.16.

Synthesis of carbon nucleophiles 2.

1. Preparation of 2-(2-pentylbuta-2,3-dienyl)malononitrile **2b**.[3] hx-13-97

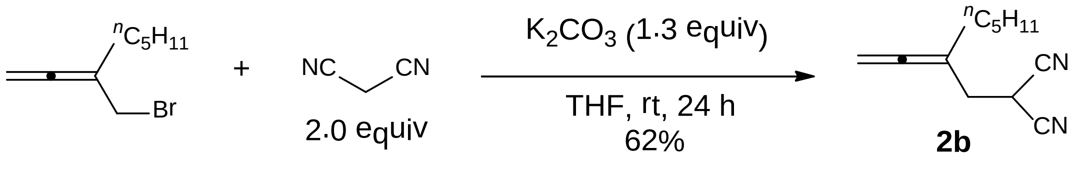

Typical Procedure II: To a three-neck flask were added 3-(bromomethyl)octa-1,2-diene (5.4998 g, purity = 74%, 20 mmol)/THF (80 mL),

malononitrile (2.5 mL, d = 1.05 g/mL, 2.63 g, 40 mmol), and K_2CO_3 (3.5887 g, 26 mmol) sequentially. After being stirred at rt for 24 h, the reaction was complete as monitored by TLC (eluent: petroleum ether/ethyl acetate = 10/1). The resulting mixture was then filtered through a short column packed with quartz sand first (2 cm) and then silica gel (2 cm), and diluted with ethyl acetate (80 mL). After evaporation of the solvent, the residual was purified by chromatography on silica gel (eluent: petroleum ether/ethyl acetate = 20/1) to afford **2b** (2.3480 g, 62%) as a liquid: ^{1}H NMR (300 MHz, $CDCl_3$) δ 5.07-4.97 (m, 2 H, CH_2), 3.86 (t, J = 7.4 Hz, 1 H, CH), 2.66-2.57 (m, 2 H, CH_2), 2.05-1.95 (m, 2 H, CH_2), 1.53-1.23 (m, 6 H, CH_2 × 3), 0.90 (t, J = 6.9 Hz, 3 H, Me); ^{13}C NMR (75 MHz, $CDCl_3$) δ 204.4, 112.6, 98.3, 80.7, 32.3, 31.6, 31.0, 26.7, 22.2, 21.1, 13.8; IR (neat) ν (cm^{-1}) 2957, 2929, 2859, 2257, 1959, 1467, 1434, 1379, 1343, 1310, 1248, 1024; MS (EI): *m/z* (%) 188 (M^+, 0.23), 67 (100); HRMS Calcd. for $C_{12}H_{16}N_2$ (M^+): 188.1313; Found: 188.1314.

2. Preparation of 2-(4-cyclohexylbuta-2,3-dienyl)malononitrile **2c**.[4] hx-13-90, hx-13-91

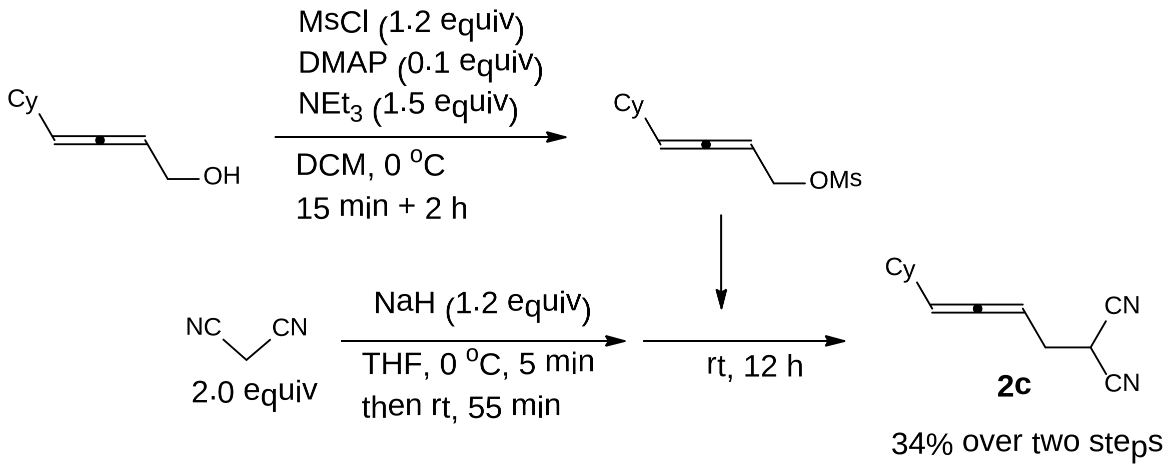

To a flame-dried three-neck flask were added DMAP (0.4281 g, 3.5 mmol), DCM (50 mL), 4-cyclohexylbuta-2,3-dien-1-ol (5.3204 g, 35 mmol)/DCM (40 mL), NEt_3 (7.3 mL, d = 0.73 g/mL, 5.33 g, 52.5 mmol) under nitrogen atmosphere. Methanesulfonyl chloride (3.3 mL, d = 1.48 g/mL, 4.88 g, 42 mmol) was added dropwise within 15 min in an ice-water bath. The reaction mixture was kept stirring at this temperature for 2 h. After the reaction was complete as monitored by TLC (eluent: petroleum ether/ethyl acetate (5/1)), it was quenched with water (50 mL). The organic phase was separated, washed with a saturated $NaHCO_3$ aqueous solution and brine, and dried over anhydrous Na_2SO_4. After filtration, evaporation of the solvent, the residual was submitted to the next step without further purification.

To a flame-dried three-neck flask were added sequentially NaH (1.7695 g, 60% in mineral oil and hydride content ≥ 95%, 42 mmol) and anhydrous THF (80 mL). Malononitrile (4.4 mL, d = 1.05 g/mL, 4.62 g, 70 mmol) was added dropwise within 5 min with an ice-water bath. Then the cooling bath was removed and the reaction mixture was warmed up to room temperature and stirred for 1 h. A solution of the residual from the previous step in anhydrous THF (20 mL) was injected with a syringe. The reaction mixture was stirred for 12 hours at rt as monitored by TLC (eluent: petroleum ether/ethyl acetate = 10/1). After quenching with water (40 mL), the resulting solution was extracted with ethyl acetate (40 mL × 3). The combined organic layer was washed with brine and dried over anhydrous Na_2SO_4. After filtration, evaporation of the solvent, and chromatography on silica gel (eluent: petroleum ether/ethyl acetate (10/1)) afforded **2c** (2.3548 g, 34% over two steps) as a

liquid: ^{1}H NMR (300 MHz, $CDCl_3$) δ 5.44-5.34 (m, 1 H, =CH), 5.23 (ddd, J_1 = 12.6 Hz, J_2 = 6.3 Hz, J_3 = 3.0 Hz, 1 H, =CH), 3.81 (t, J = 6.8 Hz, 1 H, CH), 2.78-2.58 (m, 2 H, CH_2), 2.14-1.98 (m, 1 H, CH), 1.84-1.57 (m, 5 H, five protons of Cy), 1.38-1.02 (m, 5 H, five protons of Cy); ^{13}C NMR (75 MHz, $CDCl_3$) δ 203.6, 112.4, 101.2, 84.9, 36.9, 32.7, 32.6, 30.3, 25.83, 25.76, 25.7, 22.3; IR (neat) ν (cm^{-1}) 2925, 2852, 2257, 1964, 1449, 1303, 1269, 1257, 1031; MS (EI): *m/z* (%) 200 (M^+, 7.48), 67 (100); HRMS Calcd. for $C_{13}H_{16}N_2$ (M^+): 200.1313; Found: 200.1314.

3. Preparation of 2-(undec-2-ynyl)malononitrile **2f**. wwt-1-80

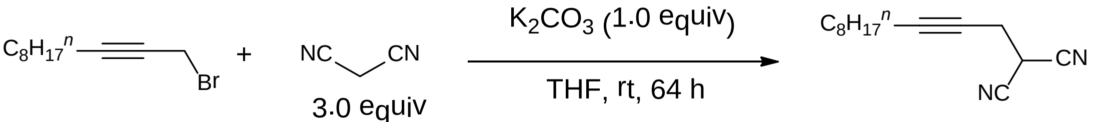

2f, 35%

Following **Typical Procedure II**, the reaction of K_2CO_3 (5.2463 g, 38 mmol), 1-bromoundec-2-yne (8.7594 g, 38 mmol), and malononitrile (7.2 mL, d = 1.05 g/mL, 7.56 g, 114 mmol) in THF (120 mL) at rt for 64 h afforded **2f** (2.8879 g, 35%) after chromatography on silica gel (eluent: petroleum ether/ethyl acetate (10/1) to afford a part of pure **2f** (0.7734 g) and a part of impure **2f**, which was further purified by chromatography on silica gel (eluent: petroleum ether/ethyl acetate (20/1)) to afford another part of pure **2f** (2.1145 g)) as a liquid: ^{1}H NMR (300 MHz, $CDCl_3$) δ 3.89 (t, J = 6.6 Hz, 1 H, CH), 2.90 (dt, J_1 = 6.6 Hz, J_2 = 2.4 Hz, 2 H, CH_2), 2.20 (tt, J_1 = 6.9 Hz, J_2 = 2.3 Hz, 2 H, CH_2), 1.58-1.45 (m, 2 H, CH_2), 1.44-1.18 (m, 10 H, $CH_2 \times 5$), 0.88 (t, J = 6.8 Hz, 3 H, Me); ^{13}C NMR (75 MHz, $CDCl_3$) δ 111.8, 87.5, 71.0, 31.7, 29.1, 29.0, 28.7, 28.3, 23.5, 22.6, 22.3, 18.5, 14.0; IR (neat, cm^{-1}) 2927, 2856, 2303, 2260, 2238, 1466, 1435, 1378, 1338, 1307, 1212, 1033; MS (EI): *m/z* (%) 215 ($(M-1)^+$,

0.56), 55 (100); Anal. Calcd for $C_{14}H_{20}N_2$ (%): C 77.73, H 9.32, N 12.95; Found: C 77.58, H 9.67, N 12.96.

4. Preparation of methyl 2-cyanododecanoate **2n**. hx-14-155

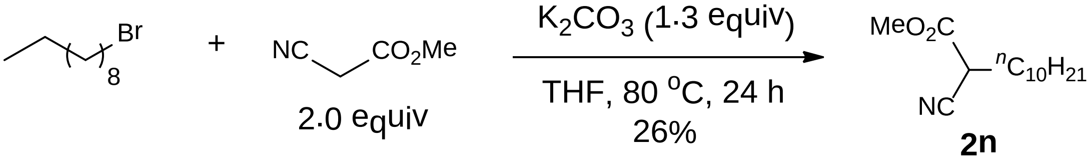

2n

Following **Typical Procedure II**, the reaction of K_2CO_3 (5.3826 g, 39 mmol), 1-bromodecane (6.2 mL, d = 1.07 g/mL, 6.63 g, 30 mmol), and methyl 2-cyanoacetate (5.4 mL, d = 1.11 g/mL, 5.99 g, 60 mmol) in THF (100 mL) at 80 °C for 24 h afforded **2n** (1.8989 g, 26%) after chromatography on silica gel (eluent: petroleum ether to petroleum ether/ethyl acetate (10/1)) as a liquid: ^{1}H NMR (300 MHz, $CDCl_3$) δ 3.82 (s, 3 H, Me), 3.51 (t, J = 7.1 Hz, 1 H, CH), 2.00-1.88 (m, 2 H, CH_2), 1.57-1.41 (m, 2 H, CH_2), 1.40-1.18 (m, 14 H, CH_2 × 7), 0.88 (t, J = 6.6 Hz, 3 H, Me); ^{13}C NMR (75 MHz, $CDCl_3$) δ 166.7, 116.4, 53.3, 37.3, 31.7, 29.7, 29.4, 29.3, 29.15, 29.07, 28.6, 26.7, 22.5, 14.0; IR (neat) ν (cm^{-1}) 2956, 2926, 2855, 2250, 1754, 1466, 1437, 1262, 1209, 1177, 1015; MS (EI): m/z (%) 239 (M^+, 0.33), 238 ($(M-H)^+$, 1.84), 41 (100); HRMS Calcd. for $C_{14}H_{25}NO_2$ (M^+): 239.1885; Found: 239.1889.

Synthesis of product 3.

1. Preparation of 2-(buta-2,3-dienyl)-2-(2-methyl-4-phenylbut-3-yn-2-yl)malono-nitrile **3aa**. hx-12-89, wwt-1-156

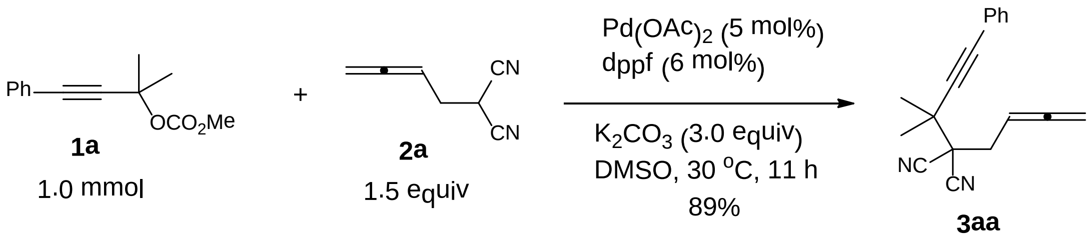

Typical Procedure III: To a flame-dried Schlenk tube containing K_2CO_3 (414.2 mg, 3.0 mmol) were added $Pd(OAc)_2$ (11.4 mg, 0.05 mmol), dppf (33.4 mg, 0.06 mmol), **1a** (217.5 mg, 1.0 mmol)/DMSO (8.0 mL), and **2a** (178.0 mg, 1.5 mmol)/DMSO (2.0 mL) sequentially under nitrogen atmosphere. The reaction was complete after being stirred at 30 °C for 11 h as monitored by TLC (eluent: petroleum ether/ethyl acetate = 20/1). After cooling to room temperature, the resulting mixture was quenched with an aqueous solution of diluted hydrochloric acid ((*v/v* = 10%, prepared via diluting commercially available concentrated hydrochloric acid with water according the volume ratio), 10 mL) slowly and extracted with ethyl ether (30 mL × 3). The combined organic layer was washed with water and brine, and dried over anhydrous Na_2SO_4. After filtration and evaporation of the solvent, chromatography on silica gel (eluent: petroleum ether/ethyl acetate = 20/1) afforded **3aa** (226.4 mg, 89%) as a liquid: ^{1}H NMR (300 MHz, $CDCl_3$) δ 7.46-7.38 (m, 2 H, ArH), 7.36-7.25 (m, 3 H, ArH), 5.40-5.28 (m, 1 H, CH=), 4.95 (dt, J_1 = 6.7 Hz, J_2 = 2.4 Hz, 2 H, $=CH_2$), 2.82 (dt, J_1 = 7.6 Hz, J_2 = 2.3 Hz, 2 H, CH_2), 1.63 (s, 6 H, Me × 2); ^{13}C NMR (75 MHz, $CDCl_3$) δ 210.5, 131.6, 128.7, 128.2, 121.5, 113.9, 87.9, 85.9, 83.0, 77.1, 48.7, 39.4, 33.7, 25.7; IR (neat, cm^{-1}) 3063, 2985, 2942, 2869, 2245, 2221, 1956, 1598, 1491, 1470, 1459, 1443, 1393, 1373, 1291, 1252, 1161, 1091, 1071, 1028; MS (EI): *m/z* (%) 260 (M^+, 100); HRMS calcd. for $C_{18}H_{16}N_2$ (M^+): 260.1313; Found:

260.1310.

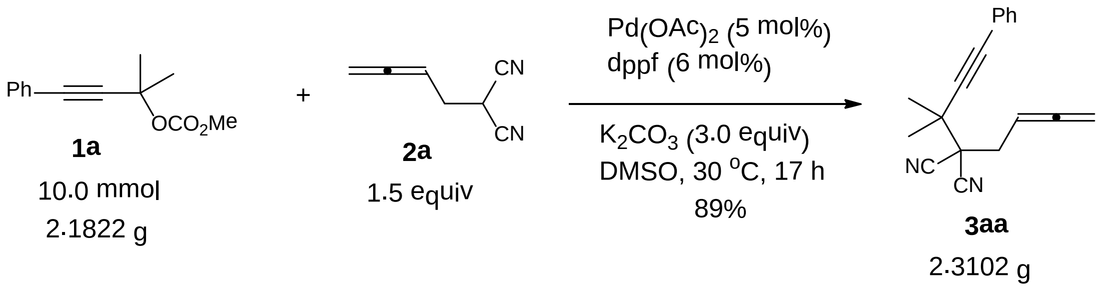

10.0 mmol-scale reaction: To a flame-dried three-neck flask containing K_2CO_3 (4.1445 g, 30.0 mmol) were added $Pd(OAc)_2$ (0.1121 g, 0.5 mmol), dppf (0.3322 g, 0.6 mmol), **1a** (2.1822 g, 10.0 mmol)/DMSO (80.0 mL), and **2a** (1.7696 g, 15.0 mmol)/DMSO (20.0 mL) sequentially under nitrogen atmosphere. The reaction was complete after being stirred at 30 °C for 17 hours as monitored by TLC (developed two times: eluent = petroleum ether/ethyl acetate = 100/1). After cooling to room temperature, the resulting mixture was quenched with an aqueous solution of diluted hydrochloric acid ((*v/v* = 10%), 80 mL) slowly and extracted with ethyl ether (100 mL × 3). The combined organic layer was washed with water followed by brine and dried over anhydrous Na_2SO_4. After filtration and evaporation of the solvent, chromatography on silica gel (eluent: petroleum ether/ethyl acetate = 20/1) afforded **3aa** (2.3102 g, 89%) as a liquid: ^{1}H NMR (300 MHz, $CDCl_3$) δ 7.47-7.38 (m, 2 H, ArH), 7.36-7.27 (m, 3 H, ArH), 5.41-5.28 (m, 1 H, CH=), 4.96 (dt, J_1 = 6.6 Hz, J_2 = 2.4 Hz, 2 H, =CH_2), 2.84 (dt, J_1 = 7.5 Hz, J_2 = 2.4 Hz, 2 H, CH_2), 1.65 (s, 6 H, Me × 2).

2. Preparation of 2-(buta-2,3-dienyl)-2-(4-(4-chlorophenyl)-2-methylbut-3-yn-2-yl)-malononitrile **3ba**. wwt-1-30

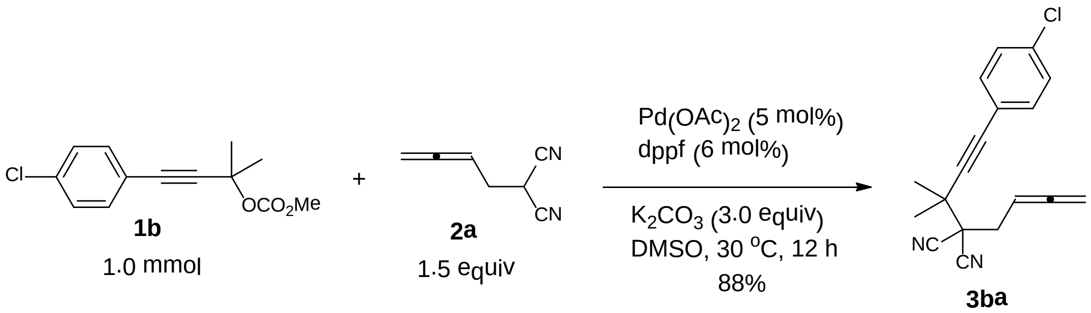

Following **Typical Procedure III**, the reaction of K_2CO_3 (413.8 mg, 3.0 mmol), $Pd(OAc)_2$ (11.3 mg, 0.05 mmol), dppf (33.4 mg, 0.06 mmol), **1b** (252.4 mg, 1.0 mmol)/DMSO (8.0 mL), and **2a** (177.8 mg, 1.5 mmol)/DMSO (2.0 mL) at 30 ^{o}C for 12 h afforded **3ba** (258.9 mg, 88%) as a liquid (eluent: petroleum ether/ethyl acetate = 20/1): ^{1}H NMR (300 MHz, $CDCl_3$) δ 7.41-7.32 (m, 2 H, ArH), 7.31-7.22 (m, 2 H, ArH), 5.41-5.27 (m, 1 H, CH=), 4.96 (dt, J_1 = 6.6 Hz, J_2 = 2.4 Hz, 2 H, =CH_2), 2.81 (dt, J_1 = 7.6 Hz, J_2 = 2.4 Hz, 2 H, CH_2), 1.64 (s, 6 H, Me × 2); ^{13}C NMR (75 MHz, $CDCl_3$) δ 210.5, 134.7, 132.9, 128.5, 120.0, 113.8, 89.0, 84.8, 82.9, 77.2, 48.7, 39.4, 33.6, 25.6; IR (neat, cm^{-1}) 3070, 2986, 2943, 2872, 2247, 2221, 1956, 1593, 1489, 1470, 1439, 1398, 1374, 1291, 1251, 1161, 1092, 1015; MS (EI): *m/z* (%) 296 (M(^{37}Cl)$^{+}$, 31.9), 294 (M(^{35}Cl)$^{+}$, 100); HRMS calcd. for $C_{18}H_{15}N_2Cl$ (M(^{35}Cl)$^{+}$): 294.0924; Found: 294.0923.

3. Preparation of 2-(buta-2,3-dienyl)-2-(2-methyl-4-(*m*-tolyl)but-3-yn-2-yl)malono-nitrile **3ca**. wwt-1-107

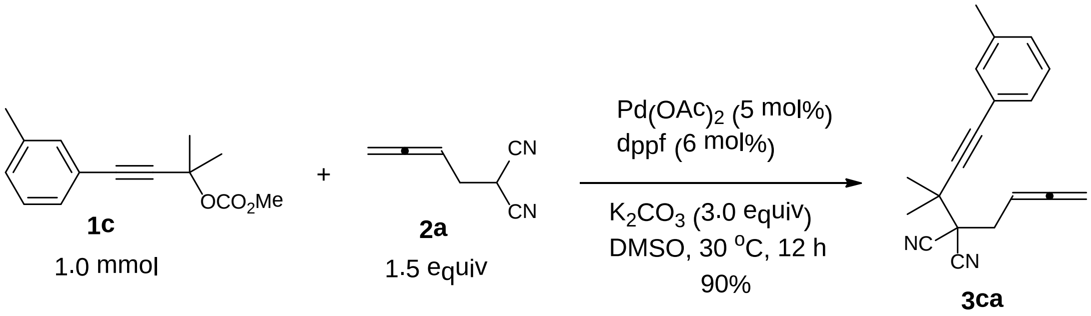

Following **Typical Procedure III**, the reaction of K_2CO_3 (414.0 mg, 3.0 mmol), $Pd(OAc)_2$ (11.5 mg, 0.05 mmol), dppf (33.7 mg, 0.06 mmol), **1c** (232.8 mg, 1.0 mmol)/DMSO (8.0 mL), and **2a** (176.7 mg, 1.5 mmol)/DMSO (2.0 mL) at 30 ^oC for 12 h afforded **3ca** (248.4 mg, 90%) as a liquid (eluent: petroleum ether/ethyl acetate = 35/1): ^1H NMR (300 MHz, $CDCl_3$) δ 7.30-7.09 (m, 4 H, ArH), 5.41-5.28 (m, 1 H, CH=), 4.96 (dt, J_1 = 6.6 Hz, J_2 = 2.4 Hz, 2 H, $=CH_2$), 2.82 (dt, J_1 = 7.7 Hz, J_2 = 2.5 Hz, 2 H, CH_2), 2.31 (s, 3 H, Me), 1.63 (s, 6 H, Me × 2); ^{13}C NMR (75 MHz, $CDCl_3$) δ 210.6, 137.9, 132.2, 129.6, 128.7, 128.1, 121.4, 113.9, 87.6, 86.1, 83.1, 77.1, 48.8, 39.4, 33.7, 25.8, 21.0; IR (neat, cm^{-1}) 2985, 2941, 2864, 2245, 2224, 1956, 1754, 1600, 1581, 1485, 1456, 1439, 1393, 1373, 1298, 1271, 1217, 1156, 1092; MS (EI): *m/z* (%) 274 (M^+, 100); HRMS calcd. for $C_{19}H_{18}N_2$ (M^+): 274.1470; Found: 274.1471.

4. Preparation of 2-(buta-2,3-dienyl)-2-(2-methyl-4-(*o*-tolyl)but-3-yn-2-yl)malono-nitrile **3da**. wwt-1-47

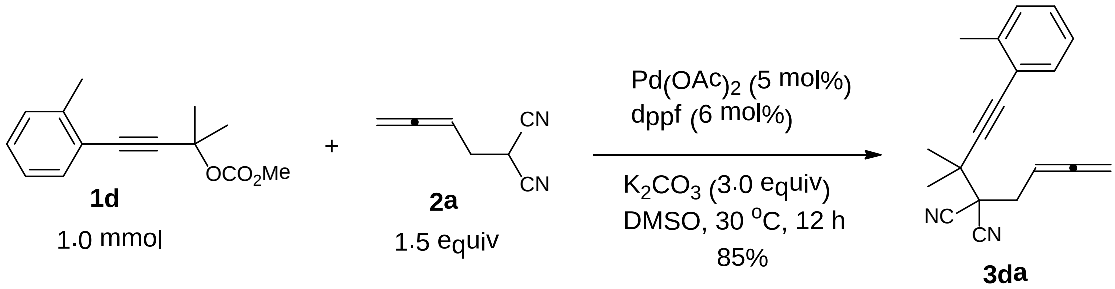

Following **Typical Procedure III**, the reaction of K_2CO_3 (414.0 mg, 3.0 mmol), $Pd(OAc)_2$ (11.4 mg, 0.05 mmol), dppf (33.5 mg, 0.06 mmol), **1d** (232.6 mg, 1.0 mmol)/DMSO (8.0 mL), and **2a** (178.8 mg, 1.5 mmol)/DMSO (2.0 mL) at 30 ^oC for

12 h afforded **3da** (234.5 mg, 85%) as a liquid (eluent: petroleum ether/ethyl acetate = 20/1): ^{1}H NMR (300 MHz, $CDCl_3$) δ 7.39 (d, J = 7.5 Hz, 1 H, ArH), 7.28-7.09 (m, 3 H, ArH), 5.40-5.27 (m, 1 H, CH=), 4.95 (dt, J_1 = 6.6 Hz, J_2 = 2.3 Hz, 2 H, $=CH_2$), 2.85 (dt, J_1 = 7.6 Hz, J_2 = 2.4 Hz, 2 H, CH_2), 2.43 (s, 3 H, Me), 1.67 (s, 6 H, Me × 2); ^{13}C NMR (75 MHz, $CDCl_3$) δ 210.6, 140.3, 131.9, 129.4, 128.8, 125.5, 121.4, 113.9, 91.9, 84.9, 83.0, 77.1, 48.8, 39.7, 33.7, 25.9, 20.6; IR (neat, cm^{-1}) 3066, 3024, 2984, 2944, 2873, 2248, 2221, 1956, 1485, 1457, 1439, 1393, 1373, 1295, 1278, 1161, 1090; MS (EI): m/z (%) 274 (M^+, 19.6), 157 (100); HRMS calcd. for $C_{19}H_{18}N_2$ (M^+): 274.1470; Found: 274.1473.

5. Preparation of 2-(buta-2,3-dienyl)-2-(2-methyl-4-(thien-3-yl)but-3-yn-2-yl)-malononitrile **3ea**. wwt-1-31

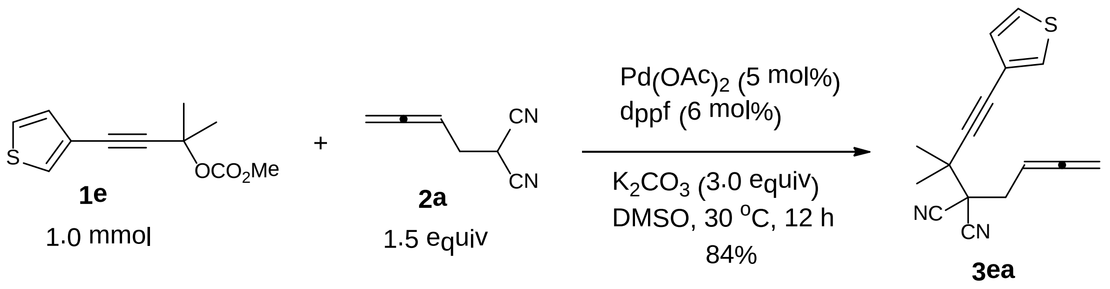

Following **Typical Procedure III**, the reaction of K_2CO_3 (414.0 mg, 3.0 mmol), $Pd(OAc)_2$ (11.2 mg, 0.05 mmol), dppf (33.4 mg, 0.06 mmol), **1e** (224.2 mg, 1.0 mmol)/DMSO (8.0 mL), and **2a** (177.7 mg, 1.5 mmol)/DMSO (2.0 mL) at 30 ^{o}C for 12 h afforded **3ea** (223.3 mg, 84%) as a liquid (eluent: petroleum ether/ethyl acetate (20/1) for the first round to get one part of pure **3ea** and a mixture of **3ea** and impurity, which was further purified by chromatography with petroleum ether/ethyl acetate (25/1) as the eluent): ^{1}H NMR (300 MHz, $CDCl_3$) δ 7.46 (dd, J_1 = 3.0 Hz, J_2 = 1.2

Hz, 1 H, ArH), 7.26 (dd, J_1 = 5.1 Hz, J_2 = 3.0 Hz, 1 H, ArH), 7.09 (dd, J_1 = 5.0 Hz, J_2 = 1.4 Hz, 1 H, ArH), 5.40-5.28 (m, 1 H, CH=), 4.96 (dt, J_1 = 6.6 Hz, J_2 = 2.4 Hz, 2 H, =CH_2), 2.82 (dt, J_1 = 7.7 Hz, J_2 = 2.3 Hz, 2 H, CH_2), 1.63 (s, 6 H, Me × 2); ^{13}C NMR (75 MHz, $CDCl_3$) δ 210.6, 129.7, 129.5, 125.5, 120.6, 113.9, 87.6, 83.0, 81.1, 77.2, 48.7, 39.5, 33.7, 25.8; IR (neat, cm^{-1}) 3111, 2985, 2942, 2872, 2248, 2225, 1956, 1470, 1457, 1436, 1393, 1373, 1359, 1272, 1247, 1210, 1155, 1090; MS (EI): *m/z* (%) 266 (M^+, 100); HRMS calcd. for $C_{16}H_{14}N_2S$ (M^+): 266.0878; Found: 266.0877.

6. Preparation of 2-(buta-2,3-dienyl)-2-(2-methyloct-3-yn-2-yl)malononitrile **3fa**. Hx-12-185, wwt-1-27

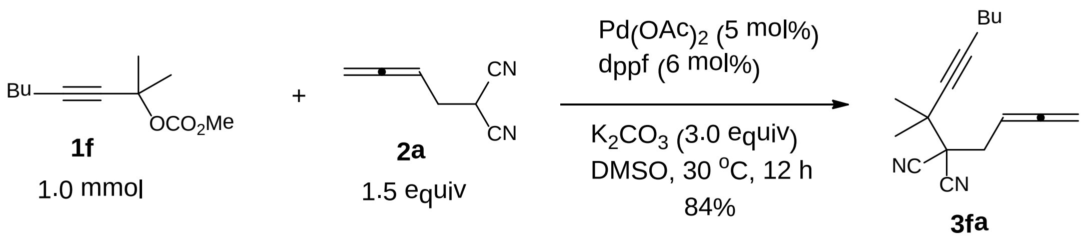

Following **Typical Procedure III**, the reaction of K_2CO_3 (414.7 mg, 3.0 mmol), $Pd(OAc)_2$ (11.2 mg, 0.05 mmol), dppf (33.1 mg, 0.06 mmol), **1f** (198.2 mg, 1.0 mmol)/DMSO (8.0 mL), and **2a** (177.3 mg, 1.5 mmol)/DMSO (2.0 mL) at 30 °C for 12 h afforded **3fa** (209.1 mg, purity = 96%, 84%) as a liquid (eluent: petroleum ether/ethyl acetate = 25/1): ^{1}H NMR (300 MHz, $CDCl_3$) δ 5.38-5.25 (m, 1 H, =CH), 4.95 (dt, J_1 = 6.6 Hz, J_2 = 2.4 Hz, 2 H, =CH_2), 2.76 (dt, J_1 = 7.7 Hz, J_2 = 2.3 Hz, 2 H, CH_2), 2.20 (t, J = 6.9 Hz, 2 H, CH_2), 1.56-1.33 (m, 10 H, Me × 2 and CH_2 × 2), 0.91 (t, J = 7.4 Hz, 3 H, Me); ^{13}C NMR (75 MHz, $CDCl_3$) δ 210.6, 114.0, 86.7, 83.1, 79.2, 76.9, 48.9, 38.9, 33.6, 30.3, 26.0, 21.7, 18.0, 13.4; IR (neat, cm^{-1}) 2983, 2959, 2936, 2874, 2242, 1956, 1755, 1457, 1435, 1393, 1373, 1328, 1266, 1242, 1164, 1093; MS

(EI): *m/z* (%) 240 (M^+, 0.78), 81 (100); HRMS calcd. for $C_{16}H_{20}N_2$ (M^+): 240.1626; Found: 240.1626.

7. Preparation of 2-(buta-2,3-dienyl)-2-(3-methyl-1-phenylpent-1-yn-3-yl)malono-nitrile **3ga**. Hx-12-184, wwt-1-28

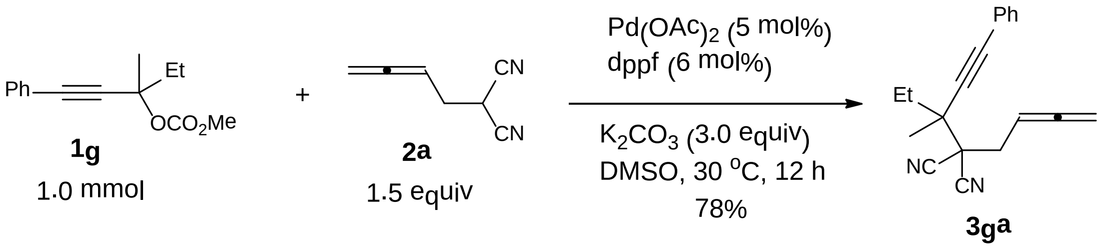

Following **Typical Procedure III**, the reaction of K_2CO_3 (414.9 mg, 3.0 mmol), $Pd(OAc)_2$ (11.3 mg, 0.05 mmol), dppf (33.3 mg, 0.06 mmol), **1g** (230.7 mg, 1.0 mmol)/DMSO (8.0 mL), and **2a** (176.9 mg, 1.5 mmol)/DMSO (2.0 mL) at 30 ^oC for 12 h afforded **3ga** (224.6 mg, purity = 95%, 78%) as a liquid (eluent: petroleum ether/ethyl acetate = 20/1): ^1H NMR (300 MHz, $CDCl_3$) δ 7.50-7.24 (m, 5 H, ArH), 5.41-5.28 (m, 1 H, =CH), 4.95 (dt, J_1 = 6.6 Hz, J_2 = 2.3 Hz, 2 H, $=CH_2$), 2.94-2.65 (m, 2 H, CH_2), 2.10-1.95 (m, 1 H, one proton of CH_2), 1.85-1.70 (m, 1 H, one proton of CH_2), 1.55 (s, 3 H, Me), 1.20 (t, J = 7.4 Hz, 3 H, Me); ^{13}C NMR (75 MHz, $CDCl_3$) δ 210.7, 131.7, 128.7, 128.2, 121.7, 114.02, 114.00, 87.2, 86.7, 83.0, 77.05, 49.2, 44.1, 33.6, 30.6, 21.6, 9.4; IR (neat, cm^{-1}) 3060, 2979, 2941, 2883, 2248, 2228, 1956, 1598, 1490, 1461, 1443, 1386, 1321, 1250, 1131, 1091, 1070; MS (EI): *m/z* (%) 274 (M^+, 100); HRMS calcd. for $C_{19}H_{18}N_2$ (M^+): 274.1470; Found: 274.1472.

8. Preparation of 2-(buta-2,3-dienyl)-2-(3-methyl-1-phenylhept-1-yn-3-yl)malono-nitrile **3ha**. wwt-1-100

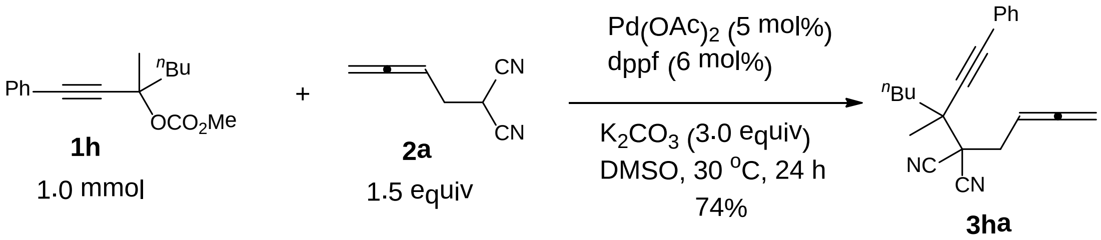

Following **Typical Procedure III**, the reaction of K_2CO_3 (413.9 mg, 3.0 mmol), $Pd(OAc)_2$ (11.6 mg, 0.05 mmol), dppf (33.6 mg, 0.06 mmol), **1h** (258.5 mg, 1.0 mmol)/DMSO (8.0 mL), and **2a** (177.3 mg, 1.5 mmol)/DMSO (2.0 mL) at 30 ^{o}C for 24 h afforded **3ha** (232.5 mg, purity = 95%, 74%) as a liquid (eluent: petroleum ether/ethyl acetate = 25/1): ^{1}H NMR (300 MHz, $CDCl_3$) δ 7.48-7.38 (m, 2 H, ArH), 7.38-7.24 (m, 3 H, ArH), 5.41-5.27 (m, 1 H, =CH), 4.95 (dt, J_1 = 6.6 Hz, J_2 = 2.3 Hz, 2 H, $=CH_2$), 2.95-2.76 (m, 2 H, CH_2), 2.00-1.88 (m, 1 H, one proton of CH_2), 1.80-1.31 (m, 8 H, one proton of CH_2 and CH_2 × 2 and Me), 0.97 (t, J = 7.2 Hz, 3 H, Me); ^{13}C NMR (75 MHz, $CDCl_3$) δ 210.7, 131.7, 128.7, 128.2, 121.7, 114.04, 114.03, 87.1, 87.0, 83.0, 77.04, 49.4, 43.5, 37.4, 33.6, 27.2, 22.7, 22.3, 13.9; IR (neat, cm^{-1}) 2958, 2865, 2245, 2221, 1956, 1748, 1596, 1491, 1467, 1443, 1384, 1325, 1272, 1248, 1132; MS (EI): *m/z* (%) 302 (M$^+$, 100); HRMS calcd. for $C_{21}H_{22}N_2$ (M$^+$): 302.1783; Found: 302.1787.

9. Preparation of 2-(buta-2,3-dienyl)-2-(3-methyl-1-(4-methoxycarbonyl)phenyl-5-phenylpent-1-yn-3-yl)malononitrile **3ia**. Hx-13-31

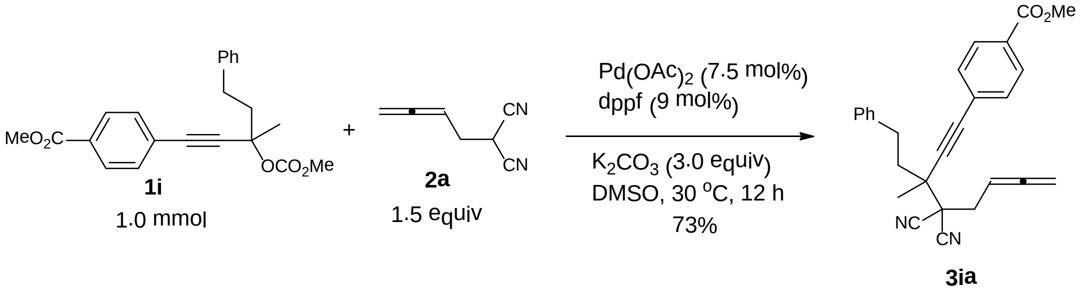

Following **Typical Procedure III**, the reaction of K_2CO_3 (414.5 mg, 3.0 mmol), $Pd(OAc)_2$ (16.8 mg, 0.075 mmol), dppf (50.0 mg, 0.09 mmol), **1i** (367.4 mg, 1.0 mmol)/DMSO (8.0 mL), and **2a** (178.1 mg, 1.5 mmol)/DMSO (2.0 mL) at 30 ^{o}C for 12 h afforded **3ia** (298.1 mg, 73%) as a solid (eluent: petroleum ether/ethyl acetate = 10/1): m.p. 88-89 ^{o}C (*n*-hexane/DCM); ^{1}H NMR (300 MHz, $CDCl_3$) δ 8.05-7.97 (m, 2 H, ArH), 7.58-7.49 (m, 2 H, ArH), 7.37-7.29 (m, 2 H, ArH), 7.29-7.20 (m, 3 H, ArH), 5.41-5.28 (m, 1 H, =CH), 4.96 (dt, J_1 = 6.6 Hz, J_2 = 2.3 Hz, 2 H, $=CH_2$), 3.93 (s, 3 H, Me), 3.07-2.78 (m, 4 H, $CH_2 \times 2$), 2.32-2.18 (m, 1 H, one proton of CH_2), 2.14-2.01 (m, 1 H, one proton of CH_2), 1.71 (s, 3 H, Me); ^{13}C NMR (75 MHz, $CDCl_3$) δ 210.8, 166.2, 140.3, 131.8, 130.2, 129.5, 128.6, 128.3, 126.4, 126.1, 113.8, 89.6, 87.0, 82.9, 77.3, 52.2, 49.3, 43.7, 39.6, 33.7, 31.6, 22.4; IR (KBr) ν (cm^{-1}) 3063, 3027, 2993, 2952, 2863, 2248, 1956, 1724, 1605, 1497, 1455, 1435, 1405, 1308, 1277, 1192, 1176, 1108, 1018; MS (EI): *m/z* (%) 408 (M^+, 4.3), 91 (100); Anal. Calcd. for $C_{27}H_{24}N_2O_2$(%): C 79.39, H 5.92, N 6.86; Found: C 78.97, H 5.97, N 6.67.

10. Preparation of 2-(buta-2,3-dienyl)-2-(1-(phenylethynyl)cyclobutyl)malononitrile **3ja**. Wwt-1-148

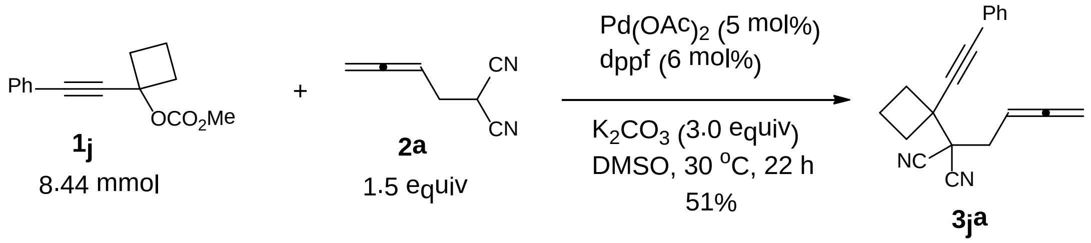

To a flame-dried three-neck flask equipped containing K_2CO_3 (3.4939 g, 25.32 mmol) were added $Pd(OAc)_2$ (0.0943 g, 0.422 mmol), dppf (0.2809 g, 0.506 mmol), **1j** (1.9421 g, 8.44 mmol)/DMSO (64.4 mL), and **2a** (1.4935 g, 12.66 mmol)/DMSO

(20.0 mL) sequentially under nitrogen atmosphere. The reaction was complete after being stirred at 30 °C for 22 h as monitored by TLC (eluent: petroleum ether/ethyl acetate = 20/1). The resulting mixture was quenched with an aqueous solution of diluted hydrochloric acid (*v/v* = 10%, 80 mL) slowly and extracted with ethyl ether (100 mL × 3). The combined organic layer was washed with water followed by brine and dried over anhydrous Na_2SO_4. After filtration and evaporation of the solvent, chromatography on silica gel (eluent: petroleum ether/ethyl acetate = 100/1) afforded **3ja** (1.1654 g, 51%) as a liquid: ^{1}H NMR (300 MHz, $CDCl_3$) δ 7.52-7.41 (m, 2 H, ArH), 7.40-7.27 (m, 3 H, ArH), 5.34-5.20 (m, 1 H, =CH), 4.96 (dt, J_1 = 6.6 Hz, J_2 = 2.4 Hz, 2 H, $=CH_2$), 2.75 (dt, J_1 = 7.7 Hz, J_2 = 2.5 Hz, 2 H, CH_2), 2.70-2.57 (m, 2 H, CH_2), 2.54-2.27 (m, 3 H, CH_2 and one proton of CH_2), 2.15-2.00 (m, 1 H, one proton of CH_2); ^{13}C NMR (75 MHz, $CDCl_3$) δ 210.6, 131.7, 128.8, 128.3, 121.8, 113.8, 87.6, 87.1, 82.6, 77.2, 47.0, 43.1, 33.7, 32.2, 15.7; IR (neat, cm^{-1}) 3059, 2997, 2948, 2855, 2248, 2218, 1956, 1598, 1491, 1443, 1311, 1256, 1158, 1110, 1070, 1027; MS (EI): *m/z* (%) 272 (M^+, 44.8), 244 (100); HRMS calcd. for $C_{19}H_{16}N_2$ (M^+): 272.1313; Found: 272.1314.

11. Preparation of 2-(buta-2,3-dienyl)-2-(1-(phenylethynyl)cyclopentyl)malononitrile **3ka**. Wwt-1-41

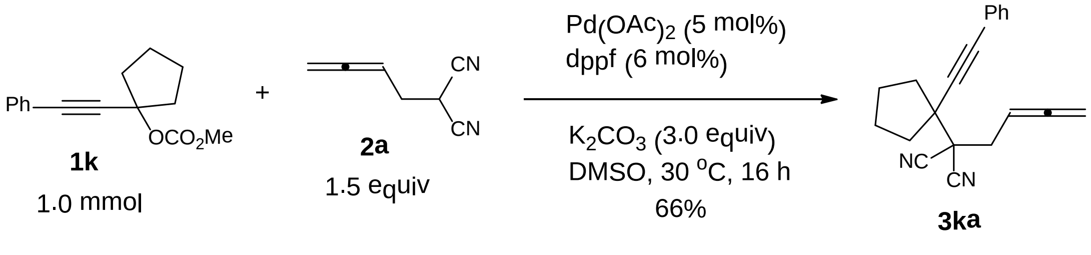

Following **Typical Procedure III**, the reaction of K_2CO_3 (414.2 mg, 3.0 mmol),

$Pd(OAc)_2$ (11.0 mg, 0.05 mmol), dppf (33.0 mg, 0.06 mmol), **1k** (243.6 mg, 1.0 mmol)/DMSO (8.0 mL), and **2a** (176.1 mg, 1.5 mmol)/DMSO (2.0 mL) at 30 °C for 16 h afforded **3ka** (187.2 mg, 66%) as a liquid (eluent: petroleum ether/ethyl acetate (30/1) afforded a part of pure **3ka** and a mixture of **3ka** and impurity, which was further purified via chromatography (eluent: petroleum ether/ethyl acetate = 50/1)): ^{1}H NMR (300 MHz, $CDCl_3$) δ 7.47-7.27 (m, 5 H, ArH), 5.40-5.28 (m, 1 H, =CH), 4.96 (dt, J_1 = 6.6 Hz, J_2 = 2.4 Hz, 2 H, =CH_2), 2.89 (dt, J_1 = 7.6 Hz, J_2 = 2.6 Hz, 2 H, CH_2), 2.30-2.17 (m, 2 H, CH_2), 2.14-1.84 (m, 6 H, CH_2 × 3); ^{13}C NMR (75 MHz, $CDCl_3$) δ 210.7, 131.7, 128.8, 128.3, 121.9, 114.3, 88.3, 86.6, 83.1, 77.3, 50.3, 47.4, 37.7, 35.2, 24.3; IR (neat, cm^{-1}) 3057, 2970, 2875, 2245, 2224, 1956, 1596, 1491, 1443, 1328, 1248, 1197, 1069, 1025; MS (EI): *m/z* (%) 286 (M^+, 52.8), 221 (100); HRMS calcd. for $C_{20}H_{18}N_2$ (M^+): 286.1470; Found: 286.1467.

12. Preparation of 2-(4-(4-methoxyphenyl)-2-methylbut-3-yn-2-yl)-2-(2-pentyl- -2,3-butadienyl)malononitrile **3lb**. hx-13-139

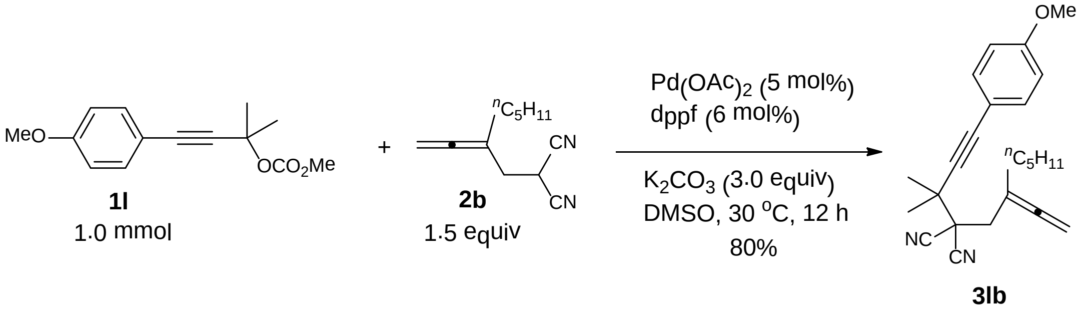

Following **Typical Procedure III**, the reaction of K_2CO_3 (414.5 mg, 3.0 mmol), $Pd(OAc)_2$ (11.3 mg, 0.05 mmol), dppf (33.1 mg, 0.06 mmol), **1l** (248.9 mg, 1.0 mmol)/DMSO (8.0 mL), and **2b** (282.7 mg, 1.5 mmol)/DMSO (2.0 mL) at 30 °C for 12 h afforded **3lb** (289.5 mg, 80%) as a liquid (eluent: petroleum ether/ethyl acetate

(100/1 to 30/1)): ^{1}H NMR (300 MHz, $CDCl_3$) δ 7.40-7.32 (m, 2 H, ArH), 6.87-6.79 (m, 2 H, ArH), 5.09-5.00 (m, 2 H, $=CH_2$), 3.79 (s, 3 H, Me), 2.77 (t, J = 3.0 Hz, 2 H, CH_2), 2.16-2.04 (m, 2 H, CH_2), 1.64 (s, 6 H, Me × 2), 1.54-1.40 (m, 2 H, CH_2), 1.39-1.24 (m, 4 H, CH_2 × 2), 0.95-0.82 (m, 3 H, Me); ^{13}C NMR (75 MHz, $CDCl_3$) δ 206.1, 159.9, 133.1, 114.4, 113.83, 113.76, 97.5, 86.9, 85.9, 80.4, 55.2, 47.1, 40.3, 35.9, 32.1, 31.2, 26.9, 25.8, 22.3, 13.9; IR (neat, cm^{-1}) 2985, 2959, 2931, 2858, 2245, 2221, 1958, 1607, 1570, 1511, 1467, 1442, 1393, 1373, 1285, 1250, 1170, 1107, 1032; MS (EI): *m/z* (%) 360 (M^+, 41.5), 303 (100); HRMS calcd. for $C_{24}H_{28}N_2O$ (M^+): 360.2202; Found: 360.2206.

13. Preparation of 2-(4-cyclohexylbuta-2,3-dienyl)-2-(2-methyl-4-(4-methoxycarbonyl)phenyl-3-butyn-2-yl)malononitrile **3mc**. hx-13-122

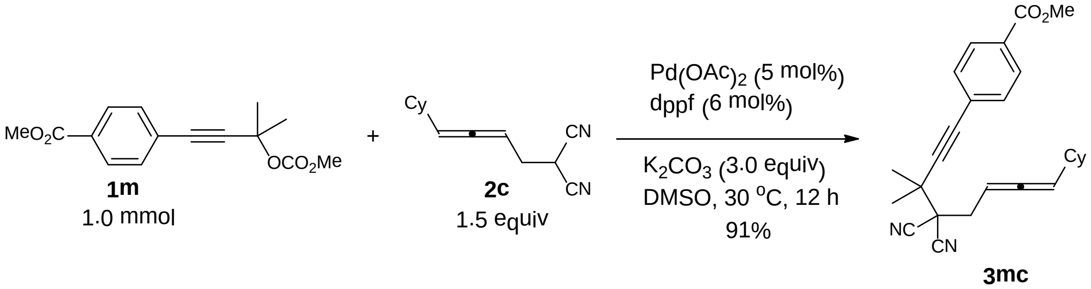

Following **Typical Procedure III**, the reaction of K_2CO_3 (414.2 mg, 3.0 mmol), $Pd(OAc)_2$ (11.3 mg, 0.05 mmol), dppf (33.3 mg, 0.06 mmol), **1m** (276.3 mg, 1.0 mmol)/DMSO (8.0 mL), and **2c** (300.2 mg, 1.5 mmol)/DMSO (2.0 mL) at 30 °C for 12 h afforded **3mc** (365.8 mg, 91%) as a liquid (eluent: petroleum ether/ethyl acetate (20/1)): ^{1}H NMR (300 MHz, $CDCl_3$) δ 7.98 (d, J = 8.4 Hz, 2 H, ArH), 7.50 (d, J = 8.4 Hz, 2 H, ArH), 5.40-5.25 (m, 2 H, CH=C=CH), 3.90 (s, 3 H, Me), 2.86-2.77 (m, 2 H, CH_2), 2.15-1.98 (m, 1 H, CH), 1.86-1.56 (m, 11 H, one proton of CH_2 and CH_2 × 2

and Me × 2), 1.37-1.05 (m, 5 H, one proton of CH_2 and CH_2 × 2); ^{13}C NMR (75 MHz, $CDCl_3$) δ 205.2, 165.9, 131.5, 129.8, 129.2, 126.1, 113.8, 113.7, 99.3, 91.0, 84.9, 84.1, 51.9, 48.5, 39.5, 36.7, 34.5, 32.6, 32.5, 25.7, 25.62, 25.59, 25.5, 25.4; IR (neat, cm^{-1}) 2985, 2927, 2852, 2248, 1963, 1727, 1606, 1436, 1405, 1373, 1307, 1276, 1189, 1175, 1107, 1019; MS (EI): *m/z* (%) 400 (M^+, 100); HRMS calcd. for $C_{26}H_{28}N_2O_2$ (M^+): 400.2151; Found: 400.2158.

14. Preparation of 2-(2-methyl-4-phenylbut-3-yn-2-yl)-2-(prop-2-ynyl)malononitrile **3ad**. hx-12-90

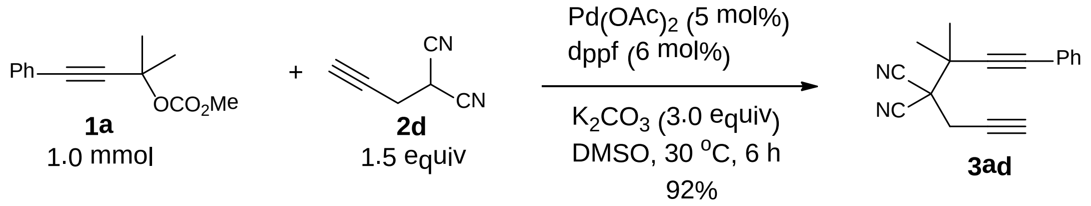

Following **Typical Procedure III**, the reaction of K_2CO_3 (414.3 mg, 3.0 mmol), $Pd(OAc)_2$ (11.3 mg, 0.05 mmol), dppf (33.4 mg, 0.06 mmol), **1a** (217.7 mg, 1.0 mmol)/DMSO (8.0 mL), and **2d** (157.7 mg, 1.5 mmol)/DMSO (2.0 mL) at 30 ^oC for 6 h afforded **3ad** (226.7 mg, 92%) as a liquid (eluent: petroleum ether/ethyl acetate (15/1)): ^1H NMR (300 MHz, $CDCl_3$) δ 7.46-7.38 (m, 2 H, ArH), 7.37-7.26 (m, 3 H, ArH), 3.09 (d, J = 2.4 Hz, 2 H, CH_2), 2.43 (t, J = 2.7 Hz, 1 H, CH), 1.63 (s, 6 H, Me × 2); ^{13}C NMR (75 MHz, $CDCl_3$) δ 131.6, 128.9, 128.2, 121.2, 113.4, 87.2, 86.3, 75.5, 74.8, 48.1, 39.5, 25.7, 25.4; IR (neat, cm^{-1}) 3296, 2986, 2941, 2251, 2230, 2129, 1593, 1491, 1471, 1458, 1443, 1429, 1395, 1375, 1292, 1260, 1188, 1162, 1083, 1071; MS (EI): *m/z* (%) 246 (M^+, 1.4), 143 (100); HRMS calcd. for $C_{17}H_{14}N_2$ (M^+): 246.1157; Found: 246.1154.

15. Preparation of 2-(3-methyl-1-phenylpent-1-yn-3-yl)-2-(prop-2-yn-1-yl)malono-nitrile **3gd**. Hx-14-15

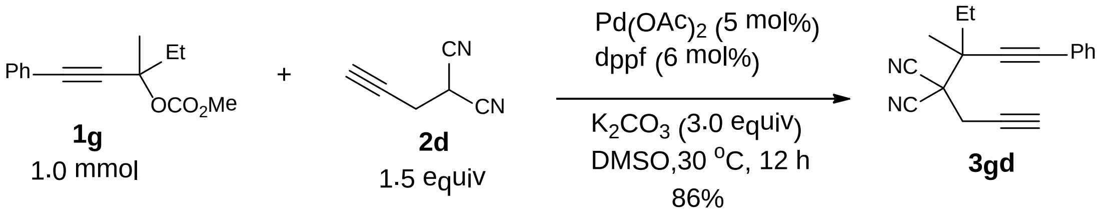

Following **Typical Procedure III**, the reaction of K_2CO_3 (414.7 mg, 3.0 mmol), $Pd(OAc)_2$ (11.4 mg, 0.05 mmol), dppf (33.2 mg, 0.06 mmol), **1g** (232.0 mg, 1.0 mmol)/DMSO (8.0 mL), and **2d** (156.4 mg, 1.5 mmol)/DMSO (2.0 mL) at 30 °C for 12 h afforded **3gd** (224.3 mg, 86%) as a liquid (eluent: petroleum ether/ethyl acetate (20/1)): ^{1}H NMR (300 MHz, $CDCl_3$) δ 7.48-7.39 (m, 2 H, ArH), 7.38-7.26 (m, 3 H, ArH), 3.16 (dd, J_1 = 16.8 Hz, J_2 = 2.7 Hz, 1 H, one proton of CH_2), 3.08 (dd, J_1 = 16.7 Hz, J_2 = 2.6 Hz, 1 H, one proton of CH_2), 2.42 (t, J = 2.6 Hz, 1 H, CH≡), 2.08-1.93 (m, 1 H, one proton of CH_2), 1.86-1.70 (m, 1 H, one proton of CH_2), 1.55 (s, 3 H, Me), 1.20 (t, J = 7.4 Hz, 3 H, Me); ^{13}C NMR (75 MHz, $CDCl_3$) δ 131.7, 128.9, 128.3, 121.4, 113.54, 113.52, 87.7, 85.9, 75.6, 74.8, 48.6, 44.1, 30.7, 25.4, 21.6, 9.4; IR (neat, cm^{-1}) 3296, 3057, 2979, 2941, 2884, 2248, 2224, 2132, 1598, 1575, 1491, 1462, 1444, 1387, 1322, 1259, 1183, 1131, 1084, 1071; MS (EI): *m/z* (%) 260 (M^+, 0.51), 157 (100); Anal. Calcd. for $C_{18}H_{16}N_2$ (%): C 83.04, H 6.19, N 10.76; Found: C 83.05, H 6.18, N 10.67.

16. Preparation of 2-(4-(4-methoxyphenyl)-2-methylbut-3-yn-2-yl)-2-(prop-2-ynyl)-malononitrile **3ld**. wwt-1-35

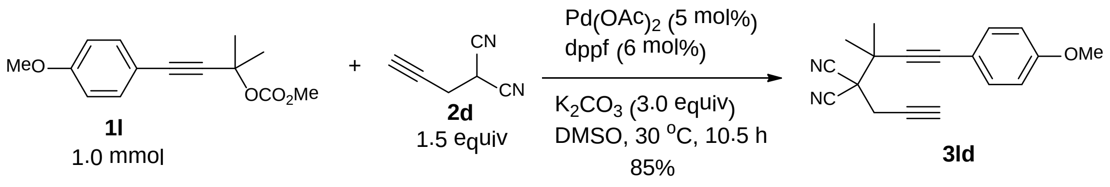

Typical Procedure IV: To a flame-dried Schlenk tube containing K_2CO_3 (414.7 mg, 3.0 mmol) were added $Pd(OAc)_2$ (11.4 mg, 0.05 mmol), dppf (33.3 mg, 0.06 mmol), **2d** (156.9 mg, 1.5 mmol), and **1l** (248.2 mg, 1.0 mmol)/DMSO (10.0 mL) sequentially under nitrogen atmosphere. The reaction was complete after being stirred at 30 ^{o}C for 10.5 h as monitored by TLC (eluent: petroleum ether/ethyl acetate = 10/1). After cooling to room temperature, the resulting mixture was quenched with an aqueous solution of diluted hydrochloric acid (*v/v* = 10%, 15 mL) slowly and extracted with ethyl ether (30 mL × 3). The combined organic layer was washed with water followed by brine and dried over anhydrous Na_2SO_4. After filtration and evaporation of the solvent, chromatography on silica gel (eluent: petroleum ether/ethyl acetate = 10/1) afforded **3ld** (234.1 mg, 85%) as a solid: m.p. 66-67 ^{o}C (*n*-hexane/DCM); ^{1}H NMR (300 MHz, $CDCl_3$) δ 7.40-7.33 (m, 2 H, ArH), 6.88-6.80 (m, 2 H, ArH), 3.80 (s, 3 H, OMe), 3.11 (d, *J* = 2.7 Hz, 2 H, CH_2), 2.43 (t, *J* = 2.6 Hz, 1 H, CH≡), 1.64 (s, 6 H, Me × 2); ^{13}C NMR (75 MHz, $CDCl_3$) δ 160.0, 133.3, 113.9, 113.5, 113.3, 86.4, 85.8, 75.6, 74.8, 55.2, 48.3, 39.6, 25.9, 25.6; IR (KBr) ν (cm^{-1}) 3293, 2985, 2939, 2840, 2229, 1607, 1571, 1511, 1468, 1442, 1395, 1374, 1286, 1251, 1171, 1108, 1081, 1030; MS (EI): *m/z* (%) 276 (M^+, 4.7), 173 (100); Anal. Calcd. for $C_{18}H_{16}N_2O$ (%): C 78.24, H 5.84, N 10.14; Found: C 78.18, H 5.90, N 10.12.

17. Preparation of 2-(2-methyl-4-(thien-2-yl)but-3-yn-2-yl)-2-(3-phenylprop-2-ynyl)-malononitrile **3ne**. wwt-1-56

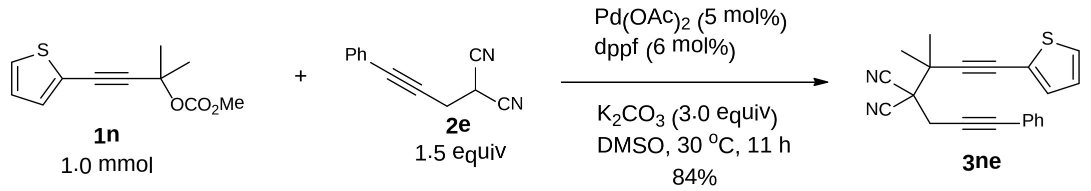

Following **Typical Procedure IV**, the reaction of K_2CO_3 (414.8 mg, 3.0 mmol), $Pd(OAc)_2$ (11.4 mg, 0.05 mmol), dppf (33.7 mg, 0.06 mmol), **2e** (270.2 mg, 1.5 mmol), and **1n** (222.6 mg, 1.0 mmol)/DMSO (10.0 mL) at 30 ^{o}C for 12 h afforded **3ne** (274.3 mg, 84%) as a solid (eluent: petroleum ether/ethyl acetate (20/1)): m.p. 94-95 ^{o}C (*n*-hexane/DCM); ^{1}H NMR (300 MHz, $CDCl_3$) δ 7.54-7.45 (m, 2 H, ArH), 7.38-7.23 (m, 5 H, ArH), 6.98 (dd, J_1 = 5.1 Hz, J_2 = 3.9 Hz, 1 H, ArH), 3.32 (s, 2 H, CH_2), 1.69 (s, 6 H, Me × 2); ^{13}C NMR (75 MHz, $CDCl_3$) δ 133.0, 131.7, 128.7, 128.2, 127.9, 127.0, 121.7, 121.0, 113.5, 91.0, 86.4, 80.7, 79.7, 48.4, 39.8, 26.4, 25.7; IR (KBr) ν (cm^{-1}) 3108, 3078, 3051, 2985, 2940, 2875, 2225, 1596, 1491, 1470, 1453, 1443, 1426, 1394, 1374, 1313, 1273, 1251, 1220, 1166, 1153, 1082, 1070, 1042; MS (EI): *m/z* (%) 328 (M^+, 82.7), 313 (100); Anal. Calcd. for $C_{21}H_{16}N_2S$ (%): C 76.80, H 4.91, N 8.53; Found: C 76.75, H 4.86, N 8.52.

18. Preparation of 2-(3-methyl-1-phenyloct-1-yn-3-yl)-2-(undec-2-yn-1-yl)malono-nitrile **3of**. wwt-1-103

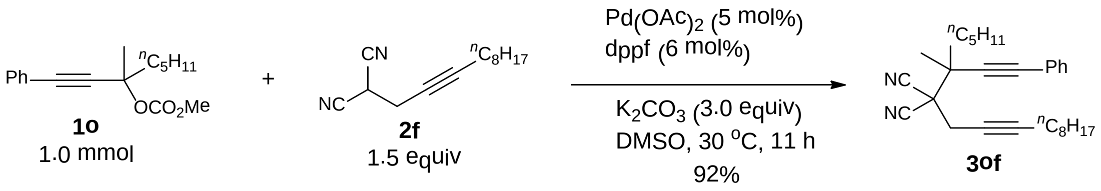

Following **Typical Procedure III**, the reaction of K_2CO_3 (414.4 mg, 3.0 mmol), $Pd(OAc)_2$ (11.5 mg, 0.05 mmol), dppf (33.4 mg, 0.06 mmol), **1o** (273.3 mg, 1.0 mmol)/DMSO (8.0 mL), and **2f** (324.4 mg, 1.5 mmol)/DMSO (2.0 mL) at 30 ^{o}C for

11 h afforded **3of** (380.5 mg, 92%) as a liquid (eluent: petroleum ether/ethyl acetate (40/1)): ^{1}H NMR (300 MHz, $CDCl_3$) δ 7.50-7.24 (m, 5 H, ArH), 3.12 (dt, J_1 = 16.2 Hz, J_2 = 2.2 Hz, 1 H, one proton of CH_2), 3.04 (dt, J_1 = 16.5 Hz, J_2 = 2.1 Hz, 1 H, one proton of CH_2), 2.30-2.16 (m, 2 H, CH_2), 1.97-1.82 (m, 1 H, one proton of CH_2), 1.80-1.18 (m, 22 H, one proton of CH_2 and CH_2 × 9 and Me), 1.00-0.78 (m, 6 H, Me × 2); ^{13}C NMR (75 MHz, $CDCl_3$) δ 131.7, 128.8, 128.2, 121.6, 114.0, 113.9, 87.2, 86.6, 71.5, 49.5, 43.4, 37.6, 31.7, 31.6, 29.0, 28.9, 28.6, 28.3, 25.8, 24.7, 22.5, 22.32, 22.25, 18.5, 14.0, 13.8; IR (neat, cm^{-1}) 2953, 2929, 2857, 2239, 1598, 1491, 1466, 1444, 1383, 1329, 1259, 1133, 1078, 1071; MS (EI): *m/z* (%) 414 (M$^+$, 33.8), 41 (100); HRMS calcd. for $C_{29}H_{38}N_2$ (M$^+$): 414.3035; Found: 414.3032.

19. Preparation of 2-allyl-2-(2-methyl-4-phenylbut-3-yn-2-yl)malononitrile **3ag**.

hx-12-188

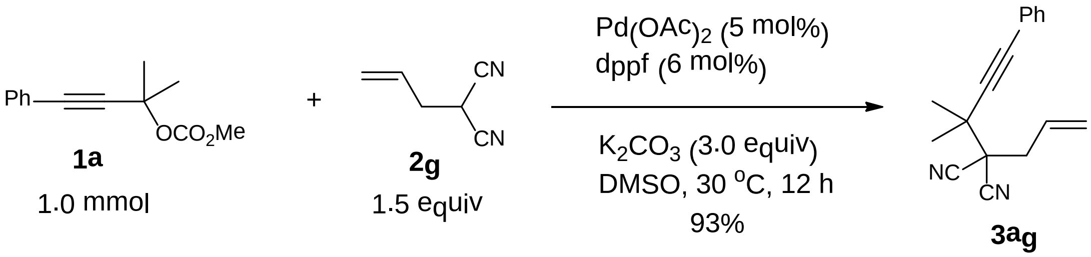

Following **Typical Procedure III**, the reaction of K_2CO_3 (414.5 mg, 3.0 mmol), $Pd(OAc)_2$ (11.2 mg, 0.05 mmol), dppf (33.2 mg, 0.06 mmol), **1a** (219.4 mg, 1.0 mmol)/DMSO (8.0 mL), and **2g** (158.9 mg, 1.5 mmol)/DMSO (2.0 mL) at 30 ^{o}C for 12 h afforded **3ag** (231.9 mg, 93%) as a liquid (eluent: petroleum ether/ethyl acetate (20/1)): ^{1}H NMR (300 MHz, $CDCl_3$) δ 7.47-7.41 (m, 2 H, ArH), 7.37-7.27 (m, 3 H, ArH), 6.10-5.94 (m, 1 H, =CH), 5.51-5.40 (m, 2 H, =CH_2), 2.87 (d, J = 7.5 Hz, 2 H, CH_2), 1.66 (s, 6 H, Me × 2); ^{13}C NMR (75 MHz, $CDCl_3$) δ 131.7, 129.4, 128.8, 128.3,

122.8, 121.6, 113.9, 88.0, 85.9, 48.5, 39.4, 38.1, 25.8; IR (neat, cm^{-1}) 3084, 3057, 2986, 2941, 2869, 2247, 2224, 1644, 1598, 1491, 1470, 1456, 1443, 1418, 1393, 1373, 1292, 1265, 1164, 1070, 1029; MS (EI): *m/z* (%) 248 (M^+, 0.78), 143 (100); Anal. Calcd. for $C_{17}H_{16}N_2$ (%): C 82.22, H 6.49, N 11.28; Found: C 82.12, H 6.39, N 11.10.

20. Preparation of 2-allyl-2-(2-methyl-4-phenylbut-3-yn-2-yl)malononitrile **3gg**.

hx-14-108

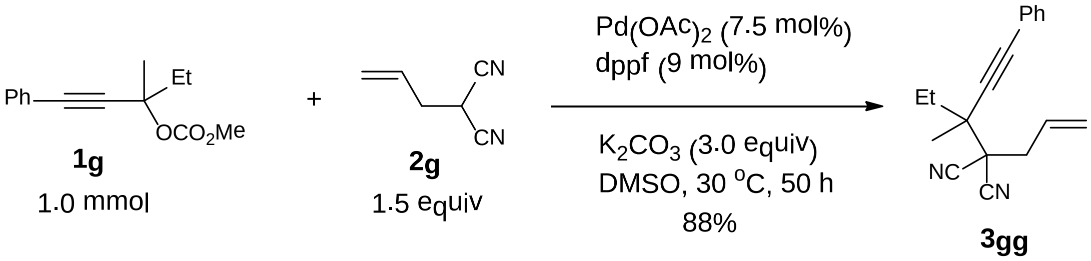

Following **Typical Procedure III**, the reaction of K_2CO_3 (415.1 mg, 3.0 mmol), $Pd(OAc)_2$ (16.7 mg, 0.075 mmol), dppf (50.0 mg, 0.09 mmol), **1g** (232.4 mg, 1.0 mmol)/DMSO (8.0 mL), and **2g** (160.3 mg, 1.5 mmol)/DMSO (2.0 mL) at 30 ^{o}C for 50 h afforded **3gg** (231.5 mg, 88%) as a liquid (eluent: petroleum ether/ethyl acetate (40/1)): ^{1}H NMR (300 MHz, $CDCl_3$) δ 7.50-7.39 (m, 2 H, ArH), 7.38-7.26 (m, 3 H, ArH), 6.11-5.93 (m, 1 H, =CH), 5.50-5.45 (m, 1 H, one proton of $=CH_2$), 5.45-5.40 (m, 1 H, one proton of $=CH_2$), 2.98-2.80 (m, 2 H, CH_2), 2.12-1.97 (m, 1 H, one proton of CH_2), 1.87-1.72 (m, 1 H, one proton of CH_2), 1.56 (s, 3 H, Me), 1.21 (t, J = 7.4 Hz, 3 H, Me); ^{13}C NMR (75 MHz, $CDCl_3$) δ 131.8, 129.5, 128.8, 128.3, 122.9, 121.8, 114.1, 87.3, 86.8, 49.0, 44.1, 38.1, 30.8, 21.7, 9.5; IR (neat, cm^{-1}) 3084, 3057, 2980, 2941, 2883, 2248, 2227, 1641, 1596, 1491, 1461, 1443, 1386, 1319, 1260, 1129, 1070; MS (EI): *m/z* (%) 262 (M^+, 1.52), 142 (100); HRMS calcd. for $C_{18}H_{18}N_2$ (M^+):

262.1470; Found: 262.1471.

21. Preparation of 2-(3-methyl-1-phenylpent-1-yn-3-yl)-2-(2-methylallyl)malono-nitrile **3gh**. wwt-1-84

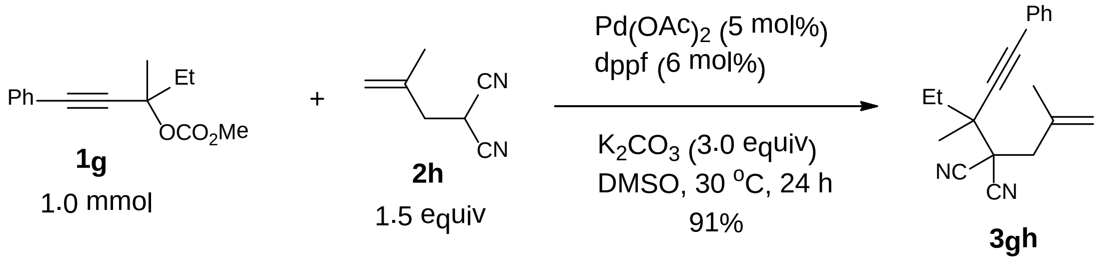

Following **Typical Procedure III**, the reaction of K_2CO_3 (414.2 mg, 3.0 mmol), $Pd(OAc)_2$ (11.5 mg, 0.05 mmol), dppf (33.5 mg, 0.06 mmol), **1g** (232.7 mg, 1.0 mmol)/DMSO (8.0 mL), and **2h** (179.9 mg, 1.5 mmol)/DMSO (2.0 mL) at 30 ^{o}C for 24 h afforded **3gh** (251.1 mg, 91%) as a liquid (eluent: petroleum ether/ethyl acetate (20/1)): ^{1}H NMR (300 MHz, $CDCl_3$) δ 7.50-7.41 (m, 2 H, ArH), 7.40-7.28 (m, 3 H, ArH), 5.19 (s, 1 H, one proton of $=CH_2$), 5.15 (s, 1 H, one proton of $=CH_2$), 2.90 (d, J = 13.8 Hz, 1 H, one proton of CH_2), 2.84 (d, J = 13.8 Hz, 1 H, one proton of CH_2), 2.13-1.97 (m, 4 H, one proton of CH_2 and Me), 1.90-1.73 (m, 1 H, one proton of CH_2), 1.57 (s, 3 H, Me), 1.22 (t, J = 7.4 Hz, 3 H, Me); ^{13}C NMR (75 MHz, $CDCl_3$) δ 137.9, 131.8, 128.8, 128.4, 121.9, 118.5, 114.60, 114.57, 87.3, 86.9, 47.9, 45.1, 41.5, 30.5, 23.1, 21.6, 9.6; IR (neat, cm^{-1}) 3082, 2977, 2941, 2882, 2245, 2224, 1652, 1598, 1491, 1443, 1383, 1321, 1262, 1244, 1128, 1070, 1029; MS (EI): m/z (%) 276 (M^+, 1.16), 142 (100); HRMS calcd. for $C_{19}H_{20}N_2$ (M^+): 276.1626; Found: 276.1627.

22. Preparation of 2-(2-methyl-4-(*p*-tolyl)but-3-yn-2-yl)-2-(3-methylbut-2-en-1-yl)-malononitrile **3pi**. wwt-1-95

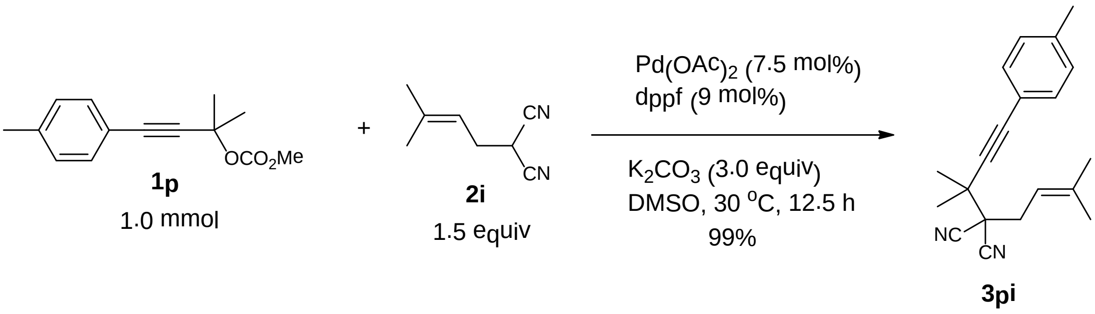

Following **Typical Procedure III**, the reaction of K_2CO_3 (413.4 mg, 3.0 mmol), $Pd(OAc)_2$ (17.0 mg, 0.075 mmol), dppf (50.3 mg, 0.09 mmol), **1p** (233.0 mg, 1.0 mmol)/DMSO (8.0 mL), and **2i** (201.0 mg, 1.5 mmol)/DMSO (2.0 mL) at 30 °C for 12.5 h afforded **3pi** (288.0 mg, 99%) as a liquid (eluent: petroleum ether/ethyl acetate (20/1)): ^{1}H NMR (300 MHz, $CDCl_3$) δ 7.32 (d, J = 8.1 Hz, 2 H, ArH), 7.12 (d, J = 7.8 Hz, 2 H, ArH), 5.41 (tm, J = 7.7 Hz, 1 H, =CH), 2.87 (d, J = 7.8 Hz, 2 H, CH_2), 2.34 (s, 3 H, Me), 1.83 (s, 3 H, Me), 1.74 (s, 3 H, Me), 1.64 (s, 6 H, Me × 2); ^{13}C NMR (75 MHz, $CDCl_3$) δ 140.3, 138.9, 131.5, 129.0, 118.7, 115.4, 114.4, 87.7, 85.9, 48.8, 39.5, 32.7, 25.91, 25.87, 21.4, 18.2; IR (neat, cm^{-1}) 3030, 2983, 2938, 2917, 2866, 2245, 2218, 1510, 1453, 1374, 1290, 1160, 1072; MS (EI): m/z (%) 290 (M$^+$, 8.19), 157 (100); HRMS calcd. for $C_{20}H_{22}N_2$ (M$^+$): 290.1783; Found: 290.1784.

23. Preparation of 2-benzyl-2-(2-methyl-4-phenylbut-3-yn-2-yl)malononitrile **3aj**.

wwt-1-61

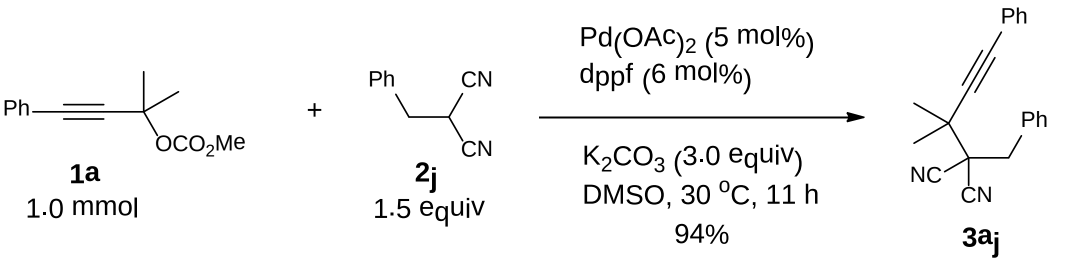

Following **Typical Procedure IV**, the reaction of K_2CO_3 (414.1 mg, 3.0 mmol), $Pd(OAc)_2$ (11.3 mg, 0.05 mmol), dppf (33.4 mg, 0.06 mmol), **2j** (233.8 mg, 1.5

mmol)/DMSO (2.0 mL), and **1a** (218.3 mg, 1.0 mmol)/DMSO (8.0 mL) at 30 °C for 11 h afforded **3aj** (279.9 mg, 94%) as a solid (eluent: petroleum ether/ethyl acetate (20/1)): m.p. 116-117 °C (*n*-hexane/DCM); ^{1}H NMR (300 MHz, $CDCl_3$) δ 7.52-7.31 (m, 10 H, ArH), 3.40 (s, 2 H, CH_2), 1.74 (s, 6 H, Me × 2); ^{13}C NMR (75 MHz, $CDCl_3$) δ 132.6, 131.6, 130.1, 128.7, 128.6, 128.4, 128.2, 121.6, 113.9, 88.2, 86.0, 50.3, 40.0, 39.4, 25.7; IR (KBr) ν (cm^{-1}) 3063, 3034, 2985, 2942, 2872, 2245, 2218, 1598, 1491, 1456, 1443, 1393, 1373, 1291, 1242, 1159, 1090; MS (EI): *m/z* (%) 298 (M^+, 1.80), 143 (100); Anal. Calcd. for $C_{21}H_{18}N_2$ (%): C 84.53, H 6.08, N 9.39; Found: C 83.73, H 6.06, N 9.21.

24. Preparation of 2-benzyl-2-(3-methyl-1-phenylpent-1-yn-3-yl)malononitrile **3gj**.

Hx-14-90

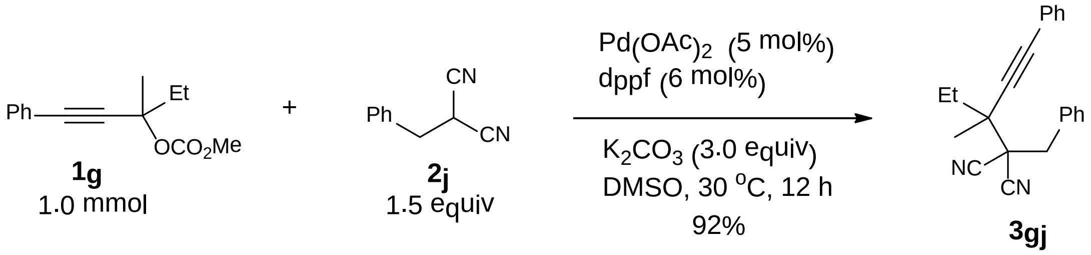

Following **Typical Procedure IV**, the reaction of K_2CO_3 (415.1 mg, 3.0 mmol), $Pd(OAc)_2$ (11.1 mg, 0.05 mmol), dppf (32.9 mg, 0.06 mmol), **2j** (233.5 mg, 1.5 mmol)/DMSO (2.0 mL), and **1g** (234.9 mg, 1.0 mmol)/DMSO (8.0 mL) at 30 °C for 12 h afforded **3gj** (291.1 mg, 92%) as a solid (eluent: petroleum ether/ethyl acetate (20/1)): m.p. 98-100 °C (*n*-hexane/DCM); ^{1}H NMR (300 MHz, $CDCl_3$) δ 7.55-7.28 (m, 10 H, ArH), 3.44 (d, J = 13.5 Hz, 1 H, one proton of CH_2), 3.37 (d, J = 13.5 Hz, 1 H, one proton of CH_2), 2.20-2.04 (m, 1 H, one proton of CH_2), 1.93-1.79 (m, 1 H, one proton of CH_2), 1.63 (s, 3 H, Me), 1.24 (t, J = 7.4 Hz, 3 H, Me); ^{13}C NMR (75 MHz,

$CDCl_3$) δ 132.7, 131.8, 130.3, 128.9, 128.8, 128.6, 128.4, 121.8, 114.1, 87.5, 87.0, 50.9, 44.8, 39.6, 30.8, 21.8, 9.6; IR (KBr) ν (cm^{-1}) 3064, 3034, 2979, 2941, 2882, 2245, 2224, 1598, 1491, 1456, 1443, 1386, 1321, 1263, 1242, 1129, 1090, 1070, 1029; MS (EI): *m/z* (%) 312 (M^+, 47.26), 91 (100); Anal. Calcd. for $C_{22}H_{20}N_2$ (%): C 84.58, H 6.45, N 8.97; Found: C 84.51, H 6.56, N 8.89.

25. Preparation of 2-decyl-2-(2-methyl-4-phenylbut-3-yn-2-yl)malononitrile **3ak**.

Hx-14-138

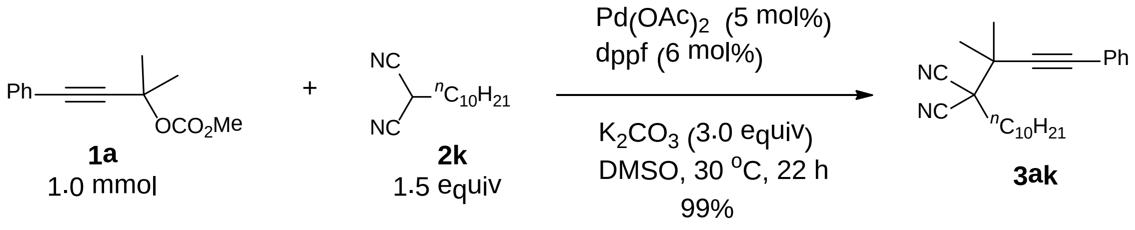

Following **Typical Procedure III**, the reaction of K_2CO_3 (413.7 mg, 3.0 mmol), $Pd(OAc)_2$ (11.4 mg, 0.05 mmol), dppf (33.4 mg, 0.06 mmol), **1a** (219.3 mg, 1.0 mmol)/DMSO (8.0 mL), and **2k** (308.4 mg, 1.5 mmol)/DMSO (2.0 mL) at 30 °C for 22 h afforded **3ak** (355.0 mg, purity = 98%, 99%) as a liquid (eluent: petroleum ether/ethyl acetate (30/1)): ^{1}H NMR (300 MHz, $CDCl_3$) δ 7.47-7.38 (m, 2 H, ArH), 7.37-7.26 (m, 3 H, ArH), 2.15-2.05 (m, 2 H, CH_2), 1.88-1.69 (m, 2 H, CH_2), 1.63 (s, 6 H, Me × 2), 1.50-1.18 (m, 14 H, CH_2 × 7), 0.88 (t, J = 6.6 Hz, 3 H, Me); ^{13}C NMR (75 MHz, $CDCl_3$) δ 131.7, 128.7, 128.3, 121.8, 114.5, 88.5, 85.6, 48.5, 39.7, 33.8, 31.8, 29.4, 29.3, 29.2, 29.1, 28.9, 26.5, 25.8, 22.6, 14.0; IR (neat) ν (cm^{-1}) 2928, 2855, 2245, 2215, 1599, 1491, 1467, 1443, 1393, 1373, 1275, 1261, 1161, 1070, 1025; MS (EI): *m/z* (%) 348 (M^+, 0.20), 143 (100); HRMS calcd. for $C_{24}H_{32}N_2$ (M^+): 348.2565;

Found: 348.2563.

26. Preparation of methyl 2-cyano-2-(2-methyl-4-phenylbut-3-yn-2-yl)pent-4-enoate **3al**. Hx-14-145

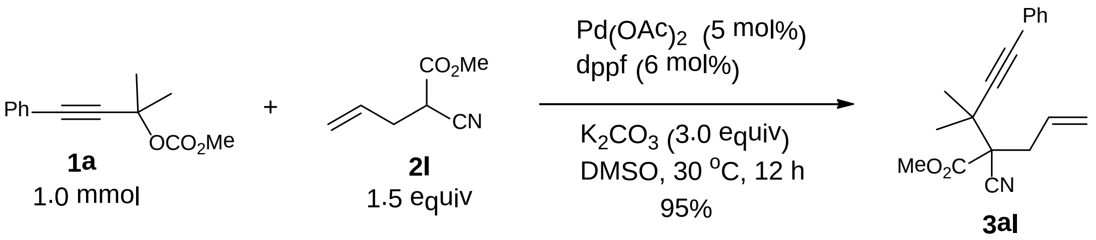

Following **Typical Procedure III**, the reaction of K_2CO_3 (414.7 mg, 3.0 mmol), $Pd(OAc)_2$ (11.3 mg, 0.05 mmol), dppf (33.3 mg, 0.06 mmol), **1a** (218.9 mg, 1.0 mmol)/DMSO (8.0 mL), and **2l** (208.5 mg, 1.5 mmol)/DMSO (2.0 mL) at 30 ^{o}C for 12 h afforded **3al** (266.8 mg, 95%) as a liquid (eluent: petroleum ether/ethyl acetate (20/1)): ^{1}H NMR (300 MHz, $CDCl_3$) δ 7.48-7.38 (m, 2 H, ArH), 7.34-7.26 (m, 3 H, ArH), 5.93-5.75 (m, 1 H, CH=), 5.33-5.20 (m, 2 H, =CH_2), 3.81 (s, 3 H, Me), 3.01 (ddt, J_1 = 13.2 Hz, J_2 = 7.7 Hz, J_3 = 0.9 Hz, 1 H, one proton of CH_2), 2.71 (ddt, J_1 = 13.5 Hz, J_2 = 6.9 Hz, J_3 = 1.2 Hz, 1 H, one proton of CH_2), 1.56 (s, 3 H, Me), 1.50 (s, 3 H, Me); ^{13}C NMR (75 MHz, $CDCl_3$) δ 167.0, 131.6, 131.2, 128.24, 128.16, 122.6, 120.9, 117.8, 90.7, 84.2, 58.3, 53.0, 38.5, 37.2 26.6, 25.5; IR (neat) ν (cm^{-1}) 3082, 3057, 2984, 2953, 2245, 1746, 1643, 1598, 1490, 1442, 1392, 1372, 1275, 1232, 1159, 1071, 1042; MS (EI): *m/z* (%) 281 (M^+, 0.52), 143 (100); Anal. Calcd. for $C_{18}H_{19}NO_2$ (%): C 76.84, H 6.81, N 4.98; Found: C 76.78, H 6.80, N 4.89.

27. Preparation of methyl 2-cyano-3,3-dimethyl-5-phenyl-2-(prop-2-ynyl)pent-4-ynoate **3am**. Hx-14-165

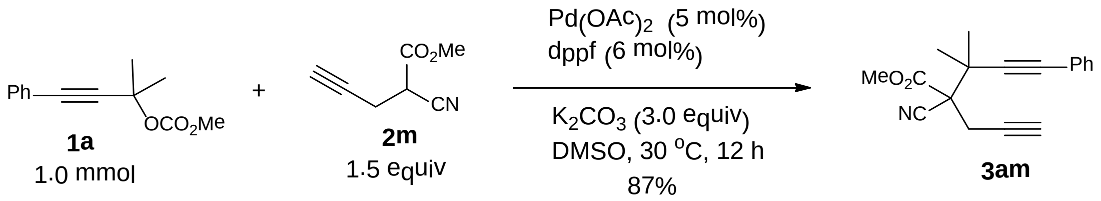

Following **Typical Procedure III**, the reaction of K_2CO_3 (415.3 mg, 3.0 mmol), $Pd(OAc)_2$ (11.4 mg, 0.05 mmol), dppf (33.5 mg, 0.06 mmol), **1a** (218.4 mg, 1.0 mmol)/DMSO (8.0 mL), and **2m** (206.2 mg, 1.5 mmol)/DMSO (2.0 mL) at 30 ^{o}C for 12 h afforded **3am** (242.2 mg, 87%) as a liquid (eluent: petroleum ether/ethyl acetate (10/1)): ^{1}H NMR (300 MHz, $CDCl_3$) δ 7.46-7.37 (m, 2 H, ArH), 7.36-7.27 (m, 3 H, ArH), 3.88 (s, 3 H, Me), 3.21 (dd, J_1 = 16.4 Hz, J_2 = 2.6 Hz, 1 H, one proton of CH_2), 2.92 (dd, J_1 = 16.4 Hz, J_2 = 2.6 Hz, 1 H, one proton of CH_2), 2.21 (t, J = 2.6 Hz, 1 H, CH), 1.56 (s, 3 H, Me), 1.47 (s, 3 H, Me); ^{13}C NMR (75 MHz, $CDCl_3$) δ 166.3, 131.5, 128.4, 128.2, 122.2, 117.2 89.7, 84.7, 77.7, 72.7, 58.0, 53.4, 38.4, 26.7, 25.5, 24.1; IR (neat) ν (cm^{-1}) 3292, 2985, 2954, 2248, 2126, 1749, 1598, 1490, 1443, 1393, 1373, 1311, 1273, 1236, 1189, 1159, 1097, 1067; MS (EI): *m/z* (%) 279 (M^+, 1.12), 143 (100); HRMS calcd. for $C_{18}H_{17}NO_2$ (M^+): 279.1259; Found: 279.1258.

28. Preparation of methyl 2-cyano-2-(2-methyl-4-phenylbut-3-yn-2-yl)dodecanoate **3an**. Hx-14-159

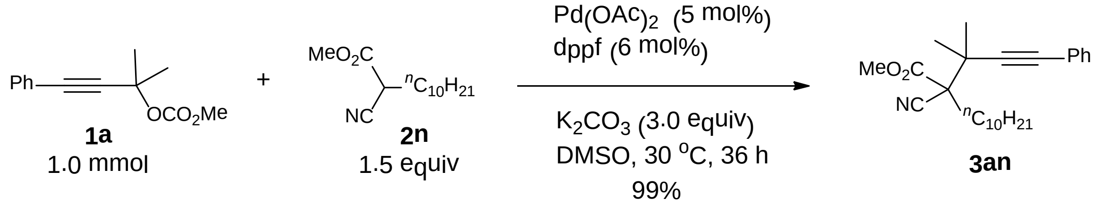

Following **Typical Procedure III**, the reaction of K_2CO_3 (414.6 mg, 3.0 mmol), $Pd(OAc)_2$ (11.4 mg, 0.05 mmol), dppf (33.2 mg, 0.06 mmol), **1a** (218.1 mg, 1.0 mmol)/DMSO (8.0 mL), and **2n** (359.1 mg, 1.5 mmol)/DMSO (2.0 mL) at 30 ^{o}C for

36 h afforded **3an** (377.3 mg, 99%) as a liquid (eluent: petroleum ether/ethyl acetate (30/1)): ^{1}H NMR (300 MHz, $CDCl_3$) δ 7.46-7.37 (m, 2 H, ArH), 7.33-7.23 (m, 3 H, ArH), 3.82 (s, 3 H, Me), 2.32-2.18 (m, 1 H, one proton of CH_2), 2.01-1.84 (m, 1 H, one proton of CH_2), 1.76-1.56 (m, 1 H, one proton of CH_2), 1.53 (s, 3 H, Me), 1.47 (s, 3 H, Me), 1.41-1.15 (m, 15 H, one proton of CH_2 and CH_2 × 7), 0.88 (t, J = 6.8 Hz, 3 H, Me); ^{13}C NMR (75 MHz, $CDCl_3$) δ 167.7, 131.5, 128.1, 122.7, 118.3, 91.1, 83.8, 58.4, 53.0, 38.7, 32.8, 31.7, 29.4, 29.31, 29.27, 29.2, 29.1, 26.5, 26.1, 25.4, 22.5, 14.0; IR (neat) ν (cm^{-1}) 2926, 2855, 2942, 1744, 1598, 1490, 1466, 1443, 1391, 1372, 1290, 1244, 1157, 1092, 1070, 1027; MS (EI): *m/z* (%) 381 (M$^+$, 0.23), 143 (100); HRMS calcd. for $C_{25}H_{35}NO_2$ (M$^+$): 381.2668; Found: 381.2672.

29. Preparation of dimethyl 2-allyl-2-(2-methyl-4-phenylbut-3-yn-2-yl)malonate **3ao**.

Hx-14-148

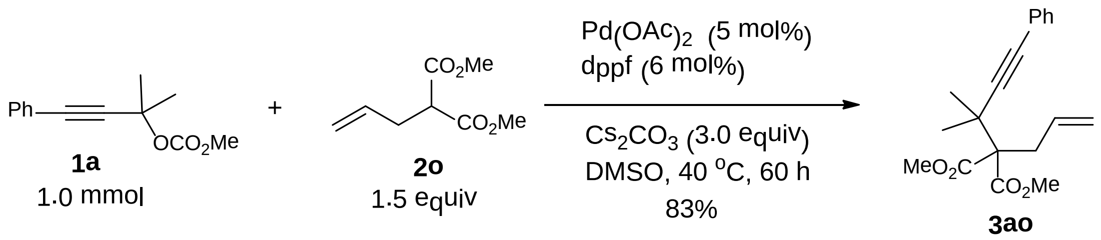

Following **Typical Procedure III**, the reaction of Cs_2CO_3 (978.2 mg, 3.0 mmol), $Pd(OAc)_2$ (11.4 mg, 0.05 mmol), dppf (33.0 mg, 0.06 mmol), **1a** (217.2 mg, 1.0 mmol)/DMSO (8.0 mL), and **2o** (257.0 mg, 1.5 mmol)/DMSO (2.0 mL) at 40 °C for 60 h afforded **3ao** (258.9 mg, 83%) as a liquid (eluent: petroleum ether/ethyl acetate (30/1)): ^{1}H NMR (300 MHz, $CDCl_3$) δ 7.43-7.34 (m, 2 H, ArH), 7.32-7.23 (m, 3 H, ArH), 6.05-5.88 (m, 1 H, CH=), 5.15-5.00 (m, 2 H, =CH_2), 3.73 (s, 6 H, Me × 2), 2.91 (d, J = 7.2 Hz, 2 H, CH_2), 1.53 (s, 6 H, Me × 2); ^{13}C NMR (75 MHz, $CDCl_3$) δ

170.1, 134.7 131.4, 128.1, 127.7, 123.5, 117.9, 94.4, 82.2, 65.0, 51.8, 37.8, 37.3, 26.7; IR (neat) ν (cm^{-1}) 3079, 2983, 2951, 2837, 2239, 1732, 1637, 1598, 1490, 1434, 1386, 1365, 1304, 1291, 1243, 1212, 1152, 1128, 1075, 1047; MS (EI): *m/z* (%) 314 (M^+, 0.10), 282 [$(M-MeOH)^+$, 7.10], 143 (100); Anal. Calcd. for $C_{19}H_{22}O_4$ (%): C 72.59, H 7.05; Found: C 72.60, H 6.99.

30. Preparation of dimethyl 2-allyl-2-(2-methyl-4-phenylbut-3-yn-2-yl)malonate **3ap**.

Hx-14-164

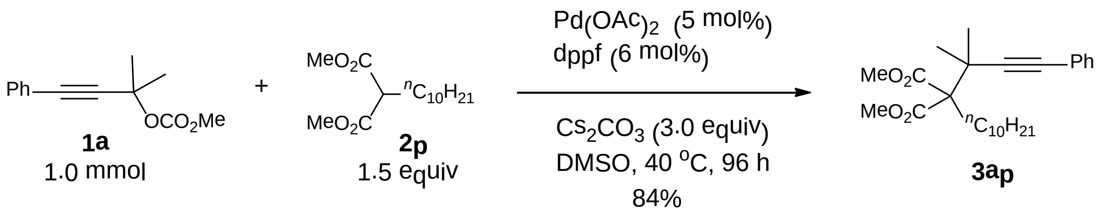

Following **Typical Procedure III**, the reaction of Cs_2CO_3 (978.2 mg, 3.0 mmol), $Pd(OAc)_2$ (11.3 mg, 0.05 mmol), dppf (33.4 mg, 0.06 mmol), **1a** (218.4 mg, 1.0 mmol)/DMSO (8.0 mL), and **2p** (408.6 mg, 1.5 mmol)/DMSO (2.0 mL) at 40 °C for 96 h afforded **3ap** (348.7 mg, 84%) as a liquid (eluent: petroleum ether/ethyl acetate (30/1)): ^{1}H NMR (300 MHz, $CDCl_3$) δ 7.42-7.33 (m, 2 H, ArH), 7.32-7.23 (m, 3 H, ArH), 3.74 (s, 6 H, Me × 2), 2.17-2.05 (m, 2 H, CH_2), 1.50 (s, 6 H, Me × 2), 1.44-1.18 (m, 16 H, CH_2 × 8), 0.88 (t, J = 6.8 Hz, 3 H, Me); ^{13}C NMR (75 MHz, $CDCl_3$) δ 170.7, 131.4, 128.1, 127.6, 123.7, 94.9, 81.9, 64.6, 51.8, 37.5, 33.1, 31.8, 30.2, 29.5, 29.3, 29.2, 26.9, 26.0, 22.6, 14.0; IR (neat) ν (cm^{-1}) 2950, 2925, 2854, 2242, 1732, 1598, 1490, 1463, 1434, 1386, 1366, 1288, 1243, 1157, 1127, 1086, 1029; MS (EI): *m/z* (%) 414 (M^+, 1.20), 143 (100); HRMS calcd. for $C_{26}H_{38}O_4$ (M^+): 414.2770; Found: 414.2768.

Mechanistic studies

1. Preparation of (*S*)-2-(buta-2,3-dien-1-yl)-2-(3-methyl-1-phenylpent-1-yn-3-yl)-malononitrile (*S*)-**3ga**. Hx-14-27

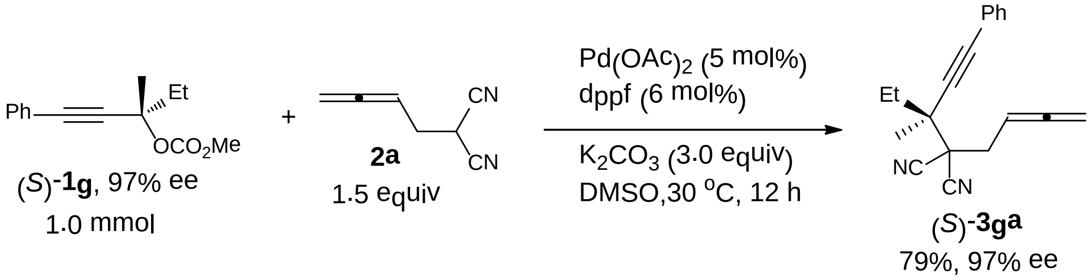

Following **Typical Procedure III**, the reaction of K_2CO_3 (413.7 mg, 3.0 mmol), $Pd(OAc)_2$ (11.4 mg, 0.05 mmol), dppf (33.3 mg, 0.06 mmol), (*S*)-**1g** (97% ee, 233.2 mg, 1.0 mmol)/DMSO (8.0 mL), and **2a** (177.5 mg, 1.5 mmol)/DMSO (2.0 mL) at 30 ^oC for 12 h afforded (*S*)-**3ga** (217.5 mg, 79%) as a liquid (eluent: petroleum ether/ethyl acetate = 20/1): 97% ee (HPLC conditions: Chiralcel IC column, hexane/*i*-PrOH = 200/1, 0.6 mL/min, λ = 214 nm, t_R(major) = 35.3 min, t_R(minor) = 37.8 min); $[\alpha]_D^{20}$ = +32.2 (*c* = 1.055, $CHCl_3$); ^1H NMR (300 MHz, $CDCl_3$) δ 7.49-7.39 (m, 2 H, ArH), 7.38-7.26 (m, 3 H, ArH), 5.41-5.28 (m, 1 H, =CH), 4.95 (dt, J_1 = 6.5 Hz, J_2 = 2.3 Hz, 2 H, $=CH_2$), 2.94-2.75 (m, 2 H, CH_2), 2.11-1.95 (m, 1 H, one proton of CH_2), 1.86-1.71 (m, 1 H, one proton of CH_2), 1.56 (s, 3 H, Me), 1.21 (t, *J* = 7.4 Hz, 3 H, Me); ^{13}C NMR (75 MHz, $CDCl_3$) δ 210.7, 131.7, 128.8, 128.3, 121.7, 114.1, 87.3, 86.7, 83.1, 77.1, 49.3, 44.1, 33.7, 30.7, 21.7, 9.5; IR (neat, cm^{-1}) 3062, 2978, 2941, 2883, 2247, 2228, 1956, 1598, 1491, 1461, 1443, 1386, 1321, 1250, 1131, 1091, 1070; MS (EI): *m/z* (%) 274 (M^+, 100); HRMS calcd. for $C_{19}H_{18}N_2$ (M^+): 274.1470; Found: 274.1469.

2. Preparation of (*S*)-2-(3-methyl-1-phenylpent-1-yn-3-yl)-2-(prop-2-yn-1-yl)-

malononitrile (*S*)-**3gd**. Hx-14-30

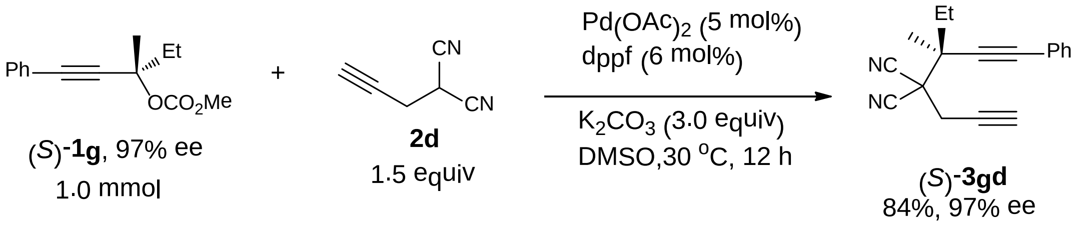

Following **Typical Procedure III**, the reaction of K_2CO_3 (414.6 mg, 3.0 mmol), $Pd(OAc)_2$ (11.2 mg, 0.05 mmol), dppf (33.3 mg, 0.06 mmol), (*S*)-**1g** (97% ee, 232.2 mg, 1.0 mmol)/DMSO (8.0 mL), and **2d** (156.2 mg, 1.5 mmol)/DMSO (2.0 mL) at 30 ^{o}C for 12 h afforded (*S*)-**3gd** (218.6 mg, 84%) as a liquid (eluent: petroleum ether/ethyl acetate (20/1 to 15/1)): 97% ee (HPLC conditions: Chiralcel OD-H column, hexane/*i*-PrOH = 200/1, 1.0 mL/min, λ = 214 nm, t_R(minor) = 17.9 min, t_R(major) = 18.8 min); $[\alpha]_D^{20}$ = +41.4 (*c* = 1.22, $CHCl_3$); ^{1}H NMR (300 MHz, $CDCl_3$) δ 7.50-7.26 (m, 5 H, ArH), 3.17 (dd, J_1 = 16.5 Hz, J_2 = 2.7 Hz, 1 H, one proton of CH_2), 3.09 (dd, J_1 = 16.7 Hz, J_2 = 2.6 Hz, 1 H, one proton of CH_2), 2.43 (t, *J* = 2.7 Hz, 1 H, CH), 2.08-1.92 (m, 1 H, one proton of CH_2), 1.87-1.71 (m, 1 H, one proton of CH_2), 1.55 (s, 3 H, Me), 1.20 (t, *J* = 7.4 Hz, 3 H, Me); ^{13}C NMR (75 MHz, $CDCl_3$) δ 131.7, 128.9, 128.3, 121.4, 113.6, 113.5, 87.7, 85.9, 75.6, 74.8, 48.6, 44.1, 30.7, 25.5, 21.6, 9.4; IR (neat, cm^{-1}) 3296, 3057, 2979, 2942, 2883, 2248, 2228, 2129, 1598, 1491, 1460, 1444, 1387, 1322, 1258, 1131, 1071; MS (EI): *m/z* (%) 260 (M^+, 0.44), 142 (100); HRMS calcd. for $C_{18}H_{16}N_2$ (M^+): 260.1313; Found: 260.1316.

3. Preparation of (*S*)-2-allyl-2-(2-methyl-4-phenylbut-3-yn-2-yl)malononitrile (*S*)-**3gg**. hx-14-113

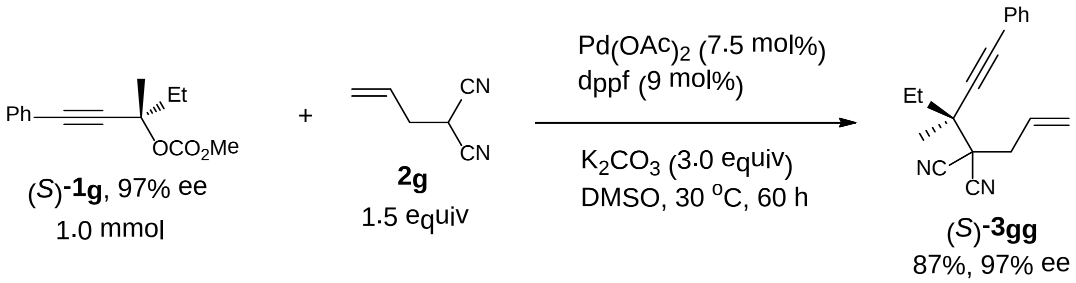

Following **Typical Procedure III**, the reaction of K_2CO_3 (413.7 mg, 3.0 mmol), $Pd(OAc)_2$ (16.7 mg, 0.075 mmol), dppf (50.1 mg, 0.09 mmol), (*S*)-**1g** (231.4 mg, 1.0 mmol)/DMSO (8.0 mL), and **2g** (159.4 mg, 1.5 mmol)/DMSO (2.0 mL) at 30 ^{o}C for 60 h afforded (*S*)-**3gg** (228.1 mg, 87%) as a liquid (eluent: petroleum ether/ethyl acetate (40/1)): 97% ee (HPLC conditions: IC column, hexane/*i*-PrOH = 200/1, 0.5 mL/min, λ = 214 nm, t_R(major) = 42.9 min, t_R(minor) = 46.1 min); $[\alpha]_D^{20}$ = +32.9 (*c* = 1.065, $CHCl_3$); ^{1}H NMR (300 MHz, $CDCl_3$) δ 7.50-7.39 (m, 2 H, ArH), 7.38-7.26 (m, 3 H, ArH), 6.11-5.93 (m, 1 H, =CH), 5.51-5.45 (m, 1 H, one proton of $=CH_2$), 5.45-5.40 (m, 1 H, one proton of $=CH_2$), 2.98-2.80 (m, 2 H, CH_2), 2.13-1.96 (m, 1 H, one proton of CH_2), 1.87-1.72 (m, 1 H, one proton of CH_2), 1.56 (s, 3 H, Me), 1.21 (t, *J* = 7.2 Hz, 3 H, Me); ^{13}C NMR (75 MHz, $CDCl_3$) δ 131.8, 129.5, 128.8, 128.3, 122.9, 121.8, 114.1, 87.3, 86.8, 49.0, 44.1, 38.1, 30.8, 21.7, 9.5; IR (neat, cm^{-1}) 3084, 3051, 2980, 2941, 2883, 2247, 2224, 1643, 1598, 1491, 1461, 1443, 1419, 1386, 1321, 1262, 1129, 1070, 1029; MS (EI): *m/z* (%) 262 (M^+, 2.09), 142 (100); HRMS calcd. for $C_{18}H_{18}N_2$ (M^+): 262.1470; Found: 262.1474.

4. Preparation of (*S*)-2-benzyl-2-(3-methyl-1-phenylpent-1-yn-3-yl)malononitrile (*S*)-**3gj**. Hx-14-92

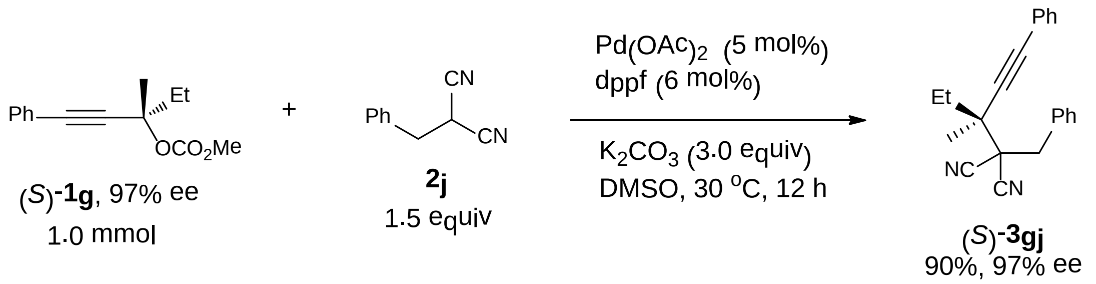

Following **Typical Procedure IV**, the reaction of K_2CO_3 (416.0 mg, 3.0 mmol), $Pd(OAc)_2$ (11.2 mg, 0.05 mmol), dppf (33.4 mg, 0.06 mmol), **2j** (234.5 mg, 1.5 mmol)/DMSO (2.0 mL), and (*S*)-**1g** (233.7 mg, 1.0 mmol)/DMSO (8.0 mL) at 30 ^{o}C for 12 h afforded (*S*)-**3gj** (282.8 mg, 90%) as a solid (eluent: petroleum ether/ethyl acetate (20/1)): 97% ee (HPLC conditions: Chiralcel IC column, hexane/*i*-PrOH = 200/1, 1.0 mL/min, λ = 214 nm, t_R(major) = 30.2 min, t_R(minor) = 33.6 min); $[\alpha]_D^{20}$ = +44.3 (*c* = 1.105, $CHCl_3$); m.p. 98-100 ^{o}C (*n*-hexane/DCM); ^{1}H NMR (300 MHz, $CDCl_3$) δ 7.55-7.30 (m, 10 H, ArH), 3.45 (d, *J* = 13.5 Hz, 1 H, one proton of CH_2), 3.38 (d, *J* = 13.2 Hz, 1 H, one proton of CH_2), 2.20-2.05 (m, 1 H, one proton of CH_2), 1.94-1.80 (m, 1 H, one proton of CH_2), 1.64 (s, 3 H, Me), 1.24 (t, *J* = 7.5 Hz, 3 H, Me); ^{13}C NMR (75 MHz, $CDCl_3$) δ 132.7, 131.8, 130.3, 128.9, 128.8, 128.6, 128.4, 121.9, 114.1, 87.6, 87.0, 50.9, 44.9, 39.6, 30.8, 21.8, 9.6; IR (KBr) ν (cm^{-1}) 3064, 3034, 2979, 2941, 2882, 2245, 2227, 1598, 1491, 1456, 1443, 1386, 1321, 1260, 1242, 1129, 1090, 1070, 1029; MS (EI): *m/z* (%) 312 (M^+, 7.71), 91 (100); Anal. Calcd. for $C_{22}H_{20}N_2$ (%): C 84.58, H 6.45, N 8.97; Found: C 84.45, H 6.49, N 8.90.

The reaction of tertiary allyl carbonate (*E*)-1q with 2-benzylmalononitrile 2j under the standard conditions. Hx-14-181

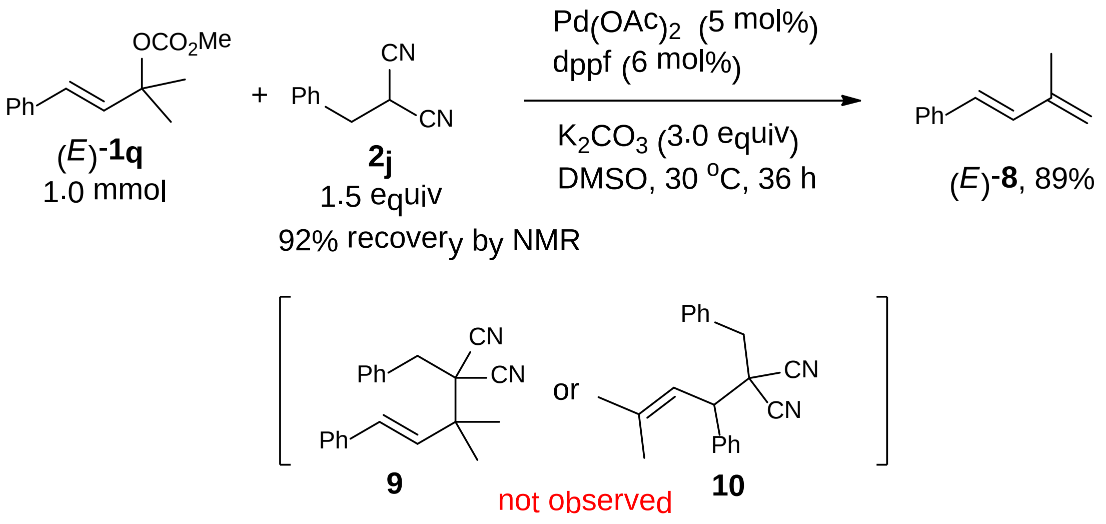

Following **Typical Procedure IV**, the reaction of K_2CO_3 (415.1 mg, 3.0 mmol), $Pd(OAc)_2$ (11.4 mg, 0.05 mmol), dppf (33.1 mg, 0.06 mmol), **2j** (234.7 mg, 1.5 mmol)/DMSO (2.0 mL), and (*E*)-**1q** (220.1 mg, 1.0 mmol)/DMSO (8.0 mL) at 30 ^{o}C for 36 h did not afford the expected nucleophilic allylation product **9** or **10**. Instead, β-elimination product, conjugated diene (*E*)-**8**[5] (128.6 mg, 89%), was afforded (eluent: petroleum ether/ethyl acetate (100/1)): ^{1}H NMR (300 MHz, $CDCl_3$) δ 7.46-7.38 (m, 2 H, ArH), 7.35-7.26 (m, 2 H, ArH), 7.25-7.17 (m, 1 H, ArH), 6.88 (d, *J* = 15.9 Hz, 1 H, =CH), 6.52 (d, *J* = 16.2 Hz, 1 H, =CH), 5.11 (s, 1 H, one proton of $=CH_2$), 5.07 (s, 1 H, one proton of $=CH_2$), 1.97 (s, 3 H, Me); ^{13}C NMR (75 MHz, $CDCl_3$) δ 142.0, 137.4, 131.6, 128.7, 128.6, 127.4, 126.4, 117.3, 18.5; IR (neat) ν (cm^{-1}) 3080, 3059, 3025, 2971, 2946, 2914, 1605, 1493, 1446, 1377, 1316, 1073; MS (EI): *m/z* (%) 144 (M^+, 29.21), 129 (100).

The reaction of tertiary alkyl carbonate 1r with 2-benzylmalononitrile 2j under the standard conditions. Hx-14-186

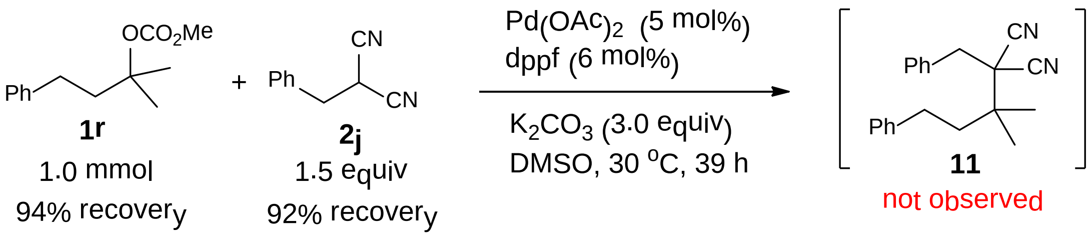

Following **Typical Procedure IV**, the reaction of K_2CO_3 (416.1 mg, 3.0 mmol), $Pd(OAc)_2$ (11.3 mg, 0.05 mmol), dppf (33.3 mg, 0.06 mmol), **2j** (234.4 mg, 1.5 mmol)/DMSO (2.0 mL), and **1r** (223.1 mg, 1.0 mmol)/DMSO (8.0 mL) at 30 ^{o}C for 39 h did not afford the expected nucleophilic substitution product **11**. The starting materials **1r** (209.6 mg, 94%) and **2j** (216.0 mg, 92%) were recovered after usual workup and chromatography on silica gel (eluent: petroleum ether/ethyl acetate (10/1 to 5/1)).

1r:[6] liquid; ^{1}H NMR (300 MHz, $CDCl_3$) δ 7.32-7.22 (m, 2 H, ArH), 7.21-7.13 (m, 3 H, ArH), 3.70 (s, 3 H, Me), 2.71-2.62 (m, 2 H, CH_2), 2.13-2.04 (m, 2 H, CH_2), 1.53 (s, 6 H, Me × 2); ^{13}C NMR (75 MHz, $CDCl_3$) δ 154.0, 141.8, 128.32, 128.26, 125.8, 83.5, 53.9, 42.4, 30.1, 25.6; IR (neat) ν (cm^{-1}) 3084, 3063, 3027, 2980, 2954, 2867, 1744, 1604, 1497, 1441, 1387, 1370, 1284, 1203, 1171, 1126, 1100, 1073; MS (EI): *m/z* (%) 222 (M^{+}, 0.01), 146 [(M-HOCO$_2$Me)$^{+}$, 99.94], 91(100).

2j:[7] solid; m.p. 89-90 ^{o}C (hexane/DCM); (Lit.[8] m.p. 90-91 ^{o}C); ^{1}H NMR (300 MHz, $CDCl_3$) δ 7.52-7.15 (m, 5 H, ArH), 3.89 (t, J = 6.9 Hz, 1 H, CH), 3.21 (d, J = 6.6 Hz, 2 H, CH_2); ^{13}C NMR (75 MHz, $CDCl_3$) δ 132.9, 129.1, 129.0, 128.6, 112.3, 36.3, 24.8; IR (KBr) ν (cm^{-1}) 3084, 3067, 3030, 2986, 2956, 2914, 2262, 1496, 1454, 1325, 1281, 1251, 1206, 1159, 1075, 1031, 1010; MS (EI): *m/z* (%) 156 (M^{+}, 10.54), 91(100).

Synthetic application of the products

1. Preparation of 1-ethyl-1-methyl-2,2-dicyano-7-nitro-9-phenyl-2,3-dihydro-1*H*-cyclopenta[*b*]naphthalene **12**.[9] hx-14-50

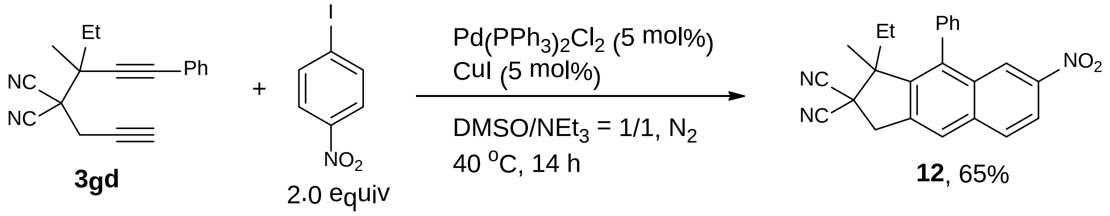

Typical Procedure V: To a flame-dried Schlenk tube were added $Pd(PPh_3)_2Cl_2$ (17.6 mg, 0.025 mmol), CuI (4.9 mg, 0.025 mmol), 4-nitrophenyl iodide (248.6 mg, 1.0 mmol), **3gd** (129.3 mg, 0.5 mmol)/DMSO (2.0 mL), and NEt_3 (2.0 mL) sequentially under nitrogen atmosphere. The reaction was complete after being stirred at 40 °C for 14 h as monitored by TLC (eluent: petroleum ether/ethyl acetate = 10/1). After cooling to room temperature, the resulting mixture was diluted with ethyl acetate (30 mL) and washed with water (20 mL). The organic layer was separated and the aqueous layer was extracted with ethyl acetate (20 mL). The combined organic layer was washed with brine and dried over anhydrous Na_2SO_4. After filtration and evaporation, the residue was purified by chromatography (eluent: petroleum ether/ethyl acetate/dichloromethane = 10/1/1) on silica gel to afford **12** (123.8 mg, 65%) as a solid: m.p. 205-206 °C (*n*-hexane/DCM); ^1H NMR (300 MHz, $CDCl_3$) δ 8.26-8.18 (m, 2 H, ArH), 8.00-7.92 (m, 1 H, ArH), 7.90 (s, 1 H, ArH), 7.62-7.49 (m, 3 H, ArH), 7.34-7.21 (m, 2 H, ArH), 3.93 (dd, J_1 = 16.5 Hz, J_2 = 0.9 Hz, 1 H, one proton of CH_2), 3.84 (dd, J_1 = 16.4 Hz, J_2 = 1.4 Hz, 1 H, one proton of CH_2),

1.95-1.78 (m, 1 H, one proton of CH_2), 1.58-1.43 (m, 1 H, one proton of CH_2), 1.42 (s, 3 H, Me), 0.97 (t, J = 7.7 Hz, 3 H, Me); ^{13}C NMR (75 MHz, $CDCl_3$) δ 145.7, 141.9, 139.2, 138.0, 135.4, 135.3, 133.1, 130.4, 130.1, 129.1, 128.8, 128.49, 128.46, 123.7, 123.1, 119.9, 115.3, 114.8, 57.3, 44.8, 41.6, 32.3, 22.9, 9.2; IR (KBr, cm^{-1}) 3081, 3058, 2979, 2940, 2883, 2248, 1628, 1610, 1584, 1531, 1490, 1461, 1442, 1391, 1378, 1339, 1287, 1266, 1218, 1090, 1054, 1028; MS (EI): *m/z* (%) 381 (M^+, 50.47), 352 (100); Anal. Calcd. for $C_{24}H_{19}N_3O_2$ (%): C 75.57, H 5.02, N 11.02; Found: C 75.58, H 5.11, N 11.17.

2. Preparation of (*S*)-1-ethyl-1-methyl-2,2-dicyano-7-nitro-9-phenyl-2,3-dihydro-1*H*-cyclopenta[*b*]naphthalene (*S*)-**12**.[9] hx-14-57

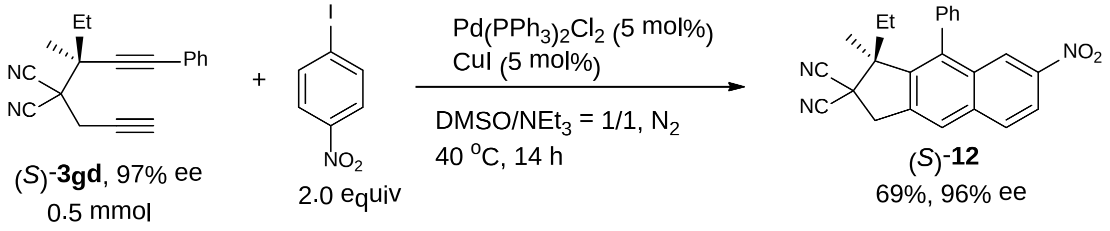

Following **Typical Procedure V**, the reaction of $Pd(PPh_3)_2Cl_2$ (17.6 mg, 0.025 mmol), CuI (4.9 mg, 0.025 mmol), 4-nitrophenyl iodide (249.3 mg, 1.0 mmol), (*S*)-**3gd** (97% ee, 130.2 mg, 0.5 mmol)/DMSO (2.0 mL), and NEt_3 (2.0 mL) at 40 °C for 14 h afforded (*S*)-**12** (129.5 mg, 69%) as a solid (eluent: petroleum ether/ethyl acetate/dichloromethane = 10/1/1): 96% ee (HPLC conditions: Chiralcel OD-H column, hexane/*i*-PrOH = 80/20, 1.0 mL/min, λ = 214 nm, t_R(major) = 9.5 min, t_R(minor) = 10.3 min); $[\alpha]_D^{20}$ = -33.9 (*c* = 0.975, $CHCl_3$); m.p. 205-206 °C (*n*-hexane/DCM); ^{1}H NMR (300 MHz, $CDCl_3$) δ 8.26-8.18 (m, 2 H, ArH), 8.00-7.93 (m, 1 H, ArH), 7.90 (s, 1 H, ArH), 7.62-7.49 (m, 3 H, ArH), 7.34-7.22 (m, 2 H, ArH),

3.93 (dd, J_1 = 16.4 Hz, J_2 = 1.1 Hz, 1 H, one proton of CH_2), 3.84 (dd, J_1 = 16.5 Hz, J_2 = 1.2 Hz, 1 H, one proton of CH_2), 1.95-1.78 (m, 1 H, one proton of CH_2), 1.58-1.39 (m, 1 H, one proton of CH_2), 1.42 (s, 3 H, Me), 0.98 (t, J = 7.5 Hz, 3 H, Me); ^{13}C NMR (75 MHz, $CDCl_3$) δ 145.7, 141.9, 139.2, 138.0, 135.4, 135.3, 133.1, 130.4, 130.1, 129.1, 128.9, 128.50, 128.46, 123.7, 123.1, 119.9, 115.3, 114.8, 57.3, 44.8, 41.6, 32.3, 22.9, 9.2; IR (KBr, cm^{-1}) 3078, 3057, 2978, 2931, 2883, 2249, 1628, 1610, 1584, 1531, 1490, 1460, 1442, 1391, 1377, 1339, 1287, 1266, 1218, 1090, 1054, 1028; MS (EI): *m/z* (%) 381 (M^+, 51.55), 352 (100); Anal. Calcd. for $C_{24}H_{19}N_3O_2$ (%): C 75.57, H 5.02, N 11.02; Found: C 75.60, H 5.05, N 11.19.

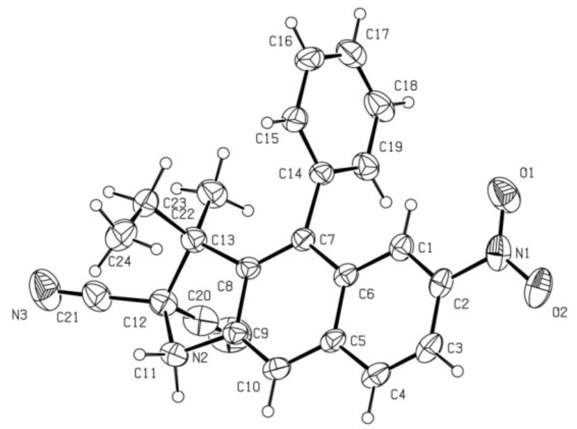

Crystal data for compound (*S*)-**12**: $C_{24}H_{19}N_3O_2$, MW = 381.42, monoclinic, space group *P 1 21 1*, final *R* indices [I > 2σ(I)], *R1* = 0.0407, *wR2* = 0.1061, *R* indices (all data) *R1* = 0.0460, *wR2* = 0.1126, a = 8.6612(2) Å, b = 16.2229(3) Å, c = 14.9775(4) Å, α = 90.00°, β = 111.523(2)°, γ = 90.00°, V = 1957.74(8) $Å^3$, T = 293(2) K, Z = 4, reflections collected/unique 13617 / 6849 (Rint = 0.0257), number of observations [> 2σ(I)] 6184, parameters: 527. CCDC 1444661 contains the supplementary

crystallographic data for this paper. These data can be obtained free of charge from The Cambridge Crystallographic Data Centre via www.ccdc.cam.ac.uk/data_request/cif.

3. Preparation of 3,3-dicyano-8-phenylspiro[bicyclo[4.2.0]octa[1(8),5]diene-2,1'-cyclobutane] **13ja**.[10,11] Hx-14-63

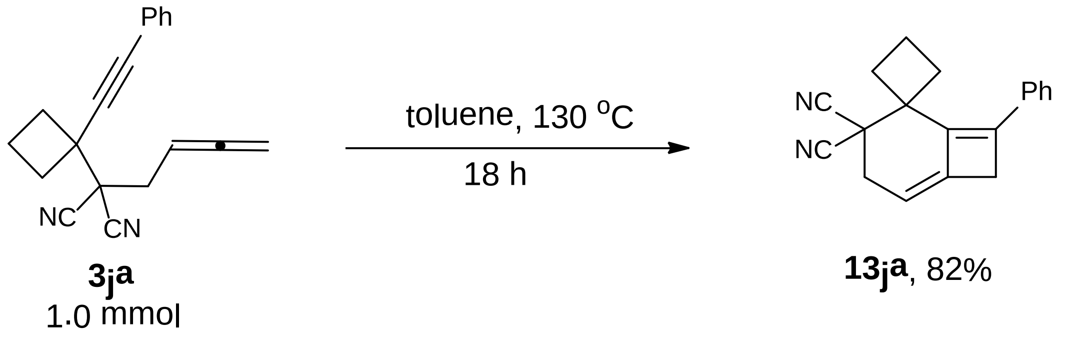

Typical Procedure VI: To a flame-dried Schlenk tube were added **3ja** (273.2 mg, 1.0 mmol)/toluene (10 mL) under nitrogen atmosphere. The reaction was complete after being stirred at 130 °C for 18 hours as monitored by TLC (eluent: petroleum ether/ethyl acetate = 20/1). After cooling to room temperature and evaporation of the solvent, the residue was purified by chromatography (eluent: petroleum ether/ethyl acetate/dichloromethane = 20/1/1) on silica gel to afford **13ja** (223.1 mg, 82%) as a solid: m.p. 133-135 °C (*n*-hexane/DCM); ^{1}H NMR (300 MHz, $CDCl_3$) δ 7.54-7.29 (m, 5 H, ArH), 5.17 (t, *J* = 3.9 Hz, 1 H, =CH), 3.32 (s, 2 H, CH_2), 2.86 (d, *J* = 3.9 Hz, 2 H, CH_2), 2.80-2.43 (m, 4 H, CH_2 × 2), 2.40-2.21 (m, 2 H, CH_2); ^{13}C NMR (75 MHz, $CDCl_3$) δ 142.1, 139.1, 138.5, 133.4, 128.7, 128.6, 127.2, 115.2, 102.0, 44.6, 42.4, 35.9, 31.9, 28.6, 14.0; IR (KBr, cm^{-1}) 3056, 2991, 2941, 2914, 2247, 1754, 1595, 1490, 1445, 1435, 1353, 1321, 1246, 1180, 1157, 1097, 1033; MS (EI): *m/z* (%) 272 (M^+, 39.83), 244 (100); Anal. Calcd. for $C_{19}H_{16}N_2$ (%): C 83.79, H 5.92, N 10.29;

Found: C 83.58, H 5.85, N 10.25.

4. Preparation of 2-ethyl-2-methyl-3,3-dicyano-8-phenylbicyclo[4.2.0]octa-1(8),5-diene **13ga**. Hx-14-70

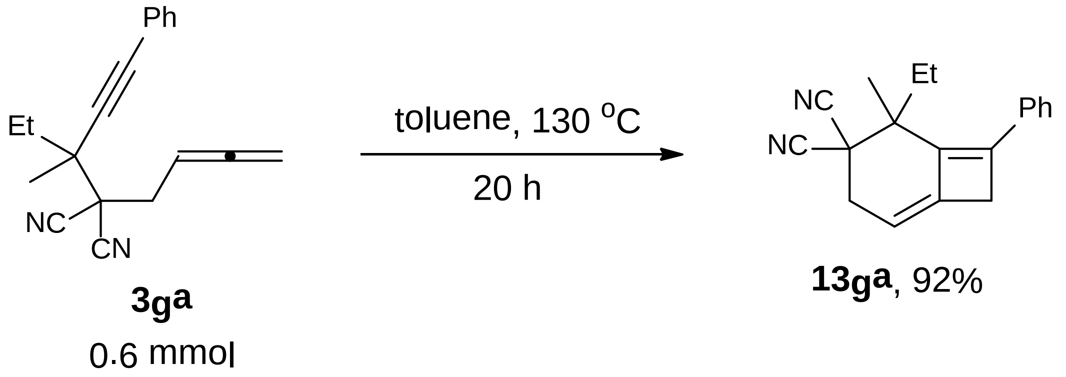

Following **Typical Procedure VI**, the reaction of **3ga** (165.1 mg. 0.6 mmol) in toluene (6.0 mL) at 130 °C for 20 h afforded **13ga** (151.2 mg, 92%) as a liquid (eluent: petroleum ether/ethyl acetate = 20/1): ^{1}H NMR (300 MHz, $CDCl_3$) δ 7.50-7.27 (m, 5 H, ArH), 5.18 (dd, J_1 = 5.1 Hz, J_2 = 2.7 Hz, 1 H, =CH), 3.36 (d, J = 13.8 Hz, 1 H, one proton of CH_2), 3.30 (d, J = 13.8 Hz, 1 H, one proton of CH_2), 3.04 (dd, J_1 = 17.1 Hz, J_2 = 2.4 Hz, 1 H, one proton of CH_2), 2.94 (dd, J_1 = 17.3 Hz, J_2 = 5.3 Hz, 1 H, one proton of CH_2), 1.88-1.65 (m, 5 H, CH_2 + Me), 1.01 (t, J = 7.5 Hz, 3 H); ^{13}C NMR (75 MHz, $CDCl_3$) δ 145.2, 138.6, 137.9, 133.1, 128.6, 128.5, 127.1, 115.6, 115.1, 101.5, 44.7, 43.8, 35.8, 32.8, 28.1, 20.0, 9.1; IR (neat, cm^{-1}) 3055, 2974, 2938, 2920, 2881, 2858, 2830, 2246, 1697, 1594, 1490, 1462, 1445, 1388, 1356, 1320, 1242, 1154, 1121, 1089; MS (EI): *m/z* (%) 274 (M^+, 99.65), 209 (100); HRMS calcd. for $C_{19}H_{18}N_2$ (M^+): 274.1470; Found: 274.1472.

5. Preparation of (S)-2-ethyl-2-methyl-3,3-dicyano-8-phenylbicyclo[4.2.0]octa-1(8),5-diene (*S*)-**13ga**.[10,11] Hx-14-68

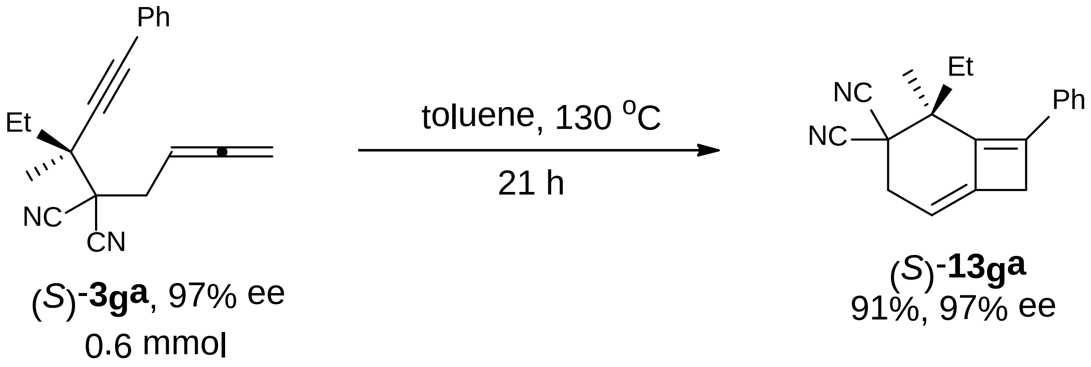

Following **Typical Procedure VI**, the reaction of (*S*)-**3ga** (97% ee, 164.9 mg. 0.6 mmol) in toluene (6.0 mL) at 130 °C for 20 h afforded (*S*)-**13ga** (149.6 mg, 91%) as a liquid (eluent: petroleum ether/ethyl acetate = 20/1): 97% ee (HPLC conditions: Chiralcel IC column, hexane/*i*-PrOH = 200/1, 1.0 mL/min, λ = 214 nm, t_R(minor) = 50.6 min, t_R(major) = 64 min); $[\alpha]_D^{20}$ = -43.5 (*c* = 0.93, $CHCl_3$); ^1H NMR (300 MHz, $CDCl_3$) δ 7.51-7.27 (m, 5 H, ArH), 5.19 (dd, J_1 = 5.1 Hz, J_2 = 2.7 Hz, 1 H, =CH), 3.37 (d, *J* = 13.8 Hz, 1 H, one proton of CH_2), 3.31 (d, *J* = 14.1 Hz, 1 H, one proton of CH_2), 3.05 (dd, J_1 = 17.3 Hz, J_2 = 2.6 Hz, 1 H, one proton of CH_2), 2.95 (dd, J_1 = 17.4 Hz, J_2 = 5.1 Hz, 1 H, one proton of CH_2), 1.90-1.64 (m, 5 H, CH_2 + Me), 1.02 (t, *J* = 7.4 Hz, 3 H); ^{13}C NMR (75 MHz, $CDCl_3$) δ 145.2, 138.7, 137.9, 133.2, 128.7, 128.6, 127.2, 115.7, 115.2, 101.5, 44.7, 43.8, 35.9, 32.9, 28.1, 20.1, 9.1; IR (neat, cm^{-1}) 3055, 2974, 2938, 2920, 2881, 2858, 2825, 2242, 1697, 1593, 1490, 1462, 1445, 1387, 1320, 1154, 1089; MS (EI): *m/z* (%) 274 (M^+, 99.98), 209 (100); HRMS calcd. for $C_{19}H_{18}N_2$ (M^+): 274.1470; Found: 274.1467.

6. Preparation of 2-benzyl-2-(3-methyl-1-phenylpent-3-yl)malononitrile **14**.[12]

Hx-14-176

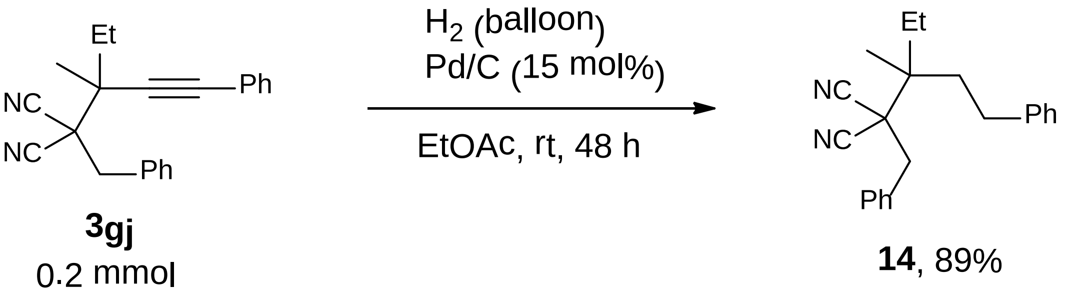

3gj
0.2 mmol

14, 89%

Typical Procedure VII: To a dry Schlenk tube were added Pd/C (dry, *w/w* (Pd) = 10%, 32.2 mg, 0.03 mmol) and **3gj** (63.0 mg, 0.2 mmol)/EtOAc (4 mL) sequentially. The resulting mixture was frozen with a liquid nitrogen bath, degassed and refilled with H_2 for three times. Then the reaction was allowed to stir at rt with a H_2 balloon. After 48 h, the reaction was complete as monitored by TLC (petroleum ether / ethyl acetate = 20/1). The mixture was filtrated through a short column of silica gel with EtOAc (20 mL × 3). After evaporation, the residue was purified by chromatography (eluent: petroleum ether/ethyl acetate = 15/1) on silica gel to afford **14** (57.1 mg, 89%) as a solid: m.p. 112-113 ^{o}C (hexane/DCM); ^{1}H NMR (300 MHz, $CDCl_3$): δ 7.50-7.15 (m, 10 H, ArH), 3.13 (s, 2 H, CH_2), 2.86-2.64 (m, 2 H, CH_2), 2.12-1.70 (m, 4 H, CH_2 × 2), 1.34 (s, 3 H, Me), 1.14 (t, J = 7.4 Hz, 3 H, Me); ^{13}C NMR (75 MHz, $CDCl_3$) δ 141.2, 132.7, 130.5, 128.7, 128.63, 128.58, 128.2, 126.3, 115.0, 49.7, 43.5, 38.4, 38.0, 30.8, 29.0, 21.5, 9.0; IR (KBr, cm^{-1}) 3087, 3066, 3024, 2975, 2949, 2881, 2239, 1599, 1498, 1472, 1455, 1435, 1386, 1239, 1090, 1031, 1010; MS (EI): *m/z* (%) 316 (M^+, 2.17), 91 (100); Anal. Calcd. for $C_{22}H_{24}N_2$ (%): C 83.50, H 7.64, N 8.85; Found: C 83.41, H 7.72, N 8.75.

7. Preparation of (*S*)-2-benzyl-2-(3-methyl-1-phenylpent-3-yl)malononitrile (*S*)-**14**.[12]

Hx-14-180

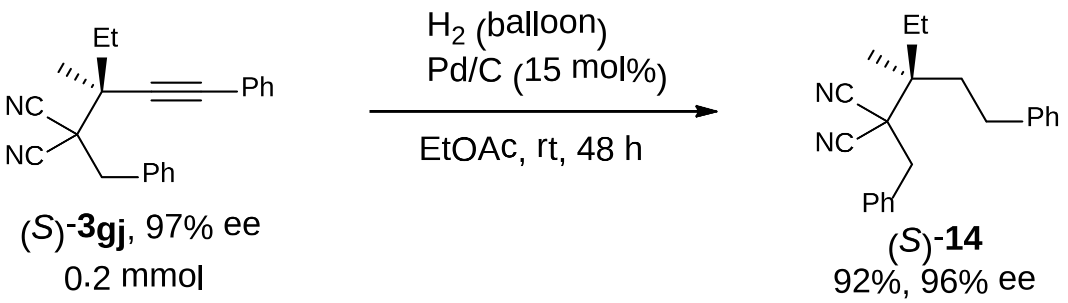

Following **Typical Procedure VII**, the reaction of Pd/C (dry, *w/w* (Pd) = 10%, 32.0 mg, 0.03 mmol), (*S*)-**3gj** (97% ee, 62.7 mg. 0.2 mmol), and H_2 in EtOAc (4.0 mL) at rt for 48 h afforded (*S*)-**14** (58.7 mg, 92%) as a solid (eluent: petroleum ether/ethyl acetate = 15/1): 96% ee (HPLC conditions: Chiralcel OD-H column, CO_2/*i*-PrOH = 95/5, 1.3 mL/min, λ = 230 nm, t_R(major) = 25.1 min, t_R(minor) = 26.5 min); $[\alpha]_D^{20}$ = +9.7 (*c* = 1.09, $CHCl_3$); m.p. 110-111 °C (hexane/DCM); ^{1}H NMR (300 MHz, $CDCl_3$): δ 7.48-7.18 (m, 10 H, ArH), 3.14 (s, 2 H, CH_2), 2.86-2.66 (m, 2 H, CH_2), 2.14-1.70 (m, 4 H, CH_2 × 2), 1.35 (s, 3 H, Me), 1.16 (t, *J* = 7.5 Hz, 3 H, Me); ^{13}C NMR (75 MHz, $CDCl_3$) δ 141.2, 132.7, 130.5, 128.8, 128.7, 128.6, 128.2, 126.3, 115.1, 49.7, 43.6, 38.4, 38.1, 30.8, 29.0, 21.5, 9.1; IR (KBr, cm^{-1}) 3087, 3066, 3024, 2975, 2949, 2885, 2866, 2242, 1599, 1498, 1472, 1455, 1432, 1386, 1242, 1090, 1028, 1010; MS (EI): *m/z* (%) 316 (M^+, 1.93), 91 (100); Anal. Calcd. for $C_{22}H_{24}N_2$ (%): C 83.50, H 7.64, N 8.85; Found: C 83.51, H 7.60, N 8.86.

8. Preparation of 2-benzyl-2-(3-methyl-1-phenylpent-1(*Z*)-en-3-yl)malononitrile (*Z*)-**15**.[13] Hx-14-175

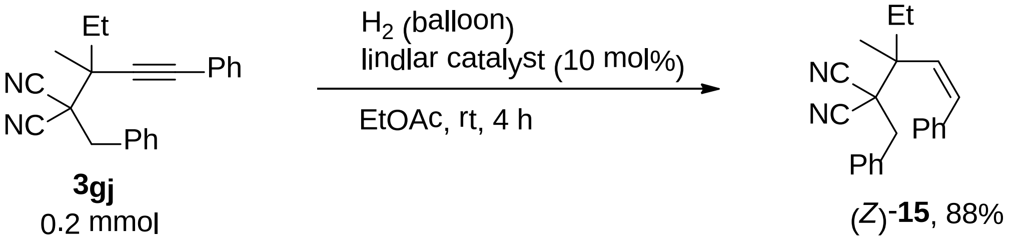

Following **Typical Procedure VII**, the reaction of Lindlar catalyst (palladium on calcium carbonate, *w/w* (Pd) = 5%, poisoned with lead acetate, 42.8 mg, 0.02 mmol), **3gj** (62.4 mg. 0.2 mmol), and H_2 in EtOAc (4.0 mL) at rt for 4 h afforded (*Z*)-**15** (55.3 mg, 88%) as a solid (eluent: petroleum ether/ethyl acetate = 30/1): m.p. 115-117 ^{o}C (hexane/DCM); ^{1}H NMR (300 MHz, $CDCl_3$): δ 7.48-7.23 (m, 8 H, ArH), 7.22-7.13 (m, 2 H, ArH), 7.09 (d, *J* = 12.6 Hz, 1 H, CH=), 5.63 (d, *J* = 12.9 Hz, 1 H, CH=), 3.22 (d, *J* = 13.5 Hz, 1 H, one proton of CH_2), 3.11 (d, *J* = 13.5 Hz, 1 H, one proton of CH_2), 2.03-1.88 (m, 1 H, one proton of CH_2), 1.82-1.66 (m, 1 H, one proton of CH_2), 1.03 (t, *J* = 7.5 Hz, 3 H, Me), 0.98 (s, 3 H, Me); ^{13}C NMR (75 MHz, $CDCl_3$) δ 137.4, 135.8, 132.8, 130.3, 130.1, 128.7, 128.5, 127.9, 127.8, 127.0, 114.6, 114.5, 51.1, 49.4, 38.8, 31.7, 18.7, 9.0; IR (KBr, cm^{-1}) 3062, 3033, 2974, 2941, 2882, 2244, 1599, 1492, 1456, 1442, 1388, 1340, 1242, 1124, 1091, 1071, 1029, 1005; MS (EI): *m/z* (%) 314 (M^+, 0.11), 159 (100); Anal. Calcd. for $C_{22}H_{22}N_2$ (%): C 84.04, H 7.05, N 8.91; Found: C 83.94, H 7.10, N 8.80.

9. Preparation of (*S*)-2-benzyl-2-(3-methyl-1-phenylpent-1(*Z*)-en-3-yl)malononitrile (*S,Z*)-**15**.[13] Hx-14-179

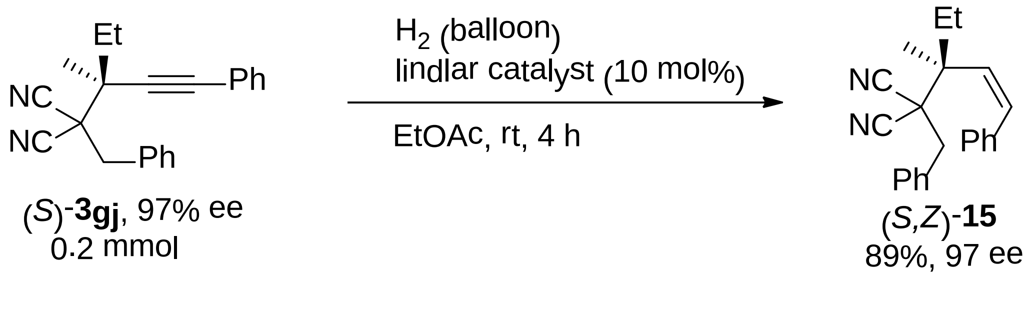

Following **Typical Procedure VII**, the reaction of Lindlar catalyst (palladium on calcium carbonate, *w/w* (Pd) = 5%, poisoned with lead acetate, 42.5 mg, 0.02 mmol), (*S*)-**3gj** (62.0 mg. 0.2 mmol) and H_2 in EtOAc (4.0 mL) at rt for 4 h afforded (*S,Z*)-**15**

(55.7 mg, 89%) as a solid (eluent: petroleum ether/ethyl acetate = 30/1): 97% ee (HPLC conditions: Chiralcel AD-H column, hexane/*i*-PrOH = 200/1, 0.6 mL/min, λ = 214 nm, t_R(minor) = 35.5 min, t_R(major) = 41.2 min); $[\alpha]_D^{20}$ = +142.7 (*c* = 0.96, $CHCl_3$); m.p. 112-113 °C (hexane/DCM); ^{1}H NMR (300 MHz, $CDCl_3$): δ 7.47-7.23 (m, 8 H, ArH), 7.22-7.14 (m, 2 H, ArH), 7.09 (d, *J* = 12.9 Hz, 1 H, CH=), 5.62 (d, *J* = 12.9 Hz, 1 H, CH=), 3.22 (d, *J* = 13.2 Hz, 1 H, one proton of CH_2), 3.11 (d, *J* = 13.2 Hz, 1 H, one proton of CH_2), 2.04-1.88 (m, 1 H, one proton of CH_2), 1.81-1.67 (m, 1 H, one proton of CH_2), 1.02 (t, *J* = 7.4 Hz, 3 H, Me), 0.98 (s, 3 H, Me); ^{13}C NMR (75 MHz, $CDCl_3$) δ 137.4, 135.9, 132.8, 130.4, 130.1, 128.8, 128.5, 128.0, 127.8, 127.1, 114.60, 114.56, 51.1, 49.4, 38.8, 31.8, 18.8, 9.0; IR (KBr, cm^{-1}) 3062, 3033, 2974, 2941, 2881, 2242, 1599, 1492, 1456, 1442, 1388, 1124, 1090, 1071, 1025; MS (EI): *m/z* (%) 314 (M^+, 0.13), 159 (100); Anal. Calcd. for $C_{22}H_{22}N_2$ (%): C 84.04, H 7.05, N 8.91; Found: C 83.95, H 7.11, N 8.78.

Supplementary References:

1. Li, Y., Zou, H., Gong, J., Xiang, J., Luo, T., Quan, J., Wang, G. & Yang, Z. Efficient synthesis of maleimides and carbazoles via $Zn(OTf)_2$-catalyzed tandem annulations of isonitriles and allenic esters. *Org. Lett.* **9**, 4057-4060 (2007).
2. Ito, H., Sasaki, Y. & Sawamura, M. Copper(I)-catalyzed substitution of propargylic carbonates with diboron: selective synthesis of multisubstituted allenylboronates. *J. Am. Chem. Soc.* **130**, 15774-15775 (2008).
3. Fernάndez-Mateos, A., Teijón, P. H., Burón, L. M., Clemente, R. R. & Gonzάlez, R. R. On the mechanism and kinetics of radical reactions of epoxyketones and epoxynitriles induced by titanocene chloride. *J. Org. Chem.* **72**, 9973-9982 (2007).
4. Ye, J., Fan, W. & Ma, S. *tert*-Butyldimethylsilyl-directed highly enantioselective approach to axially chiral α-Allenols. *Chem. Eur. J.* **19**, 716-720 (2013).
5. Ting, C., Hsu, Y. & Liu, R. Gold-catalyzed isomerization of unactivated allenes into 1,3-dienes under ambient conditions. *Chem. Commun.* **48**, 6577-6579 (2012).
6. Hatano, M., Kamiya, S. & Ishihara, K. In situ generated ‘‘lanthanum(III) nitrate alkoxide’’ as a highly active and nearly neutral transesterification catalyst. *Chem. Commun.* **48**, 9465-9467 (2012).
7. Wang, J., Song, G., Peng, Y. & Zhu, Y. 3-Butyl-1-methylimidazolinium borohydride ([bmim][BH_4])-a novel reducing agent for the selective reduction of carbon–carbon double bonds in activated conjugated alkenes. *Tetrahedron Lett.* **49**, 6518-6520 (2008).

8. Zhang, Z., Gao, J., Xia, J. & Wang, G. Solvent-free mechanochemical and one-pot reductive benzylizations of malononitrile and 4-methylaniline using Hantzsch 1,4-dihydropyridine as the reductant. *Org. Biomol. Chem.* **3**, 1617-1619 (2005).
9. Zhu, S., Cao, J., Wu, L. & Huang, X. Synthesis of polycyclic psoindoline derivatives via tandem Pd-catalyzed coupling, propargyl–allenyl isomerization, [4 + 2] cycloaddition and aromatization reaction. *J. Org. Chem.* **77**, 10409-10415 (2012).
10. Ohno, H., Mizutani, T., Kadoh, Y., Miyamura, K. & Tanaka T. Thermal intramolecular [2+2] cycloaddition of allenenes and allenynes: diastereoselective access to bicyclic nitrogen heterocycles. *Angew. Chem. Int. Ed.* **44**, 5113-5115 (2005).
11. Mailyan, A. K., Krylov, I. M., Bruneau, C., Dixneuf, P. H. & Osipov, S. N. Thermal [2+2] cycloaddition of CF_3-substituted allenynes: access to novel cyclobutene-containing α-amino acids. *Synlett* **16**, 2321-2324 (2011).
12. Huang, X., Jiang, X., Fu, C. & Ma, S. Palladium(0)-catalyzed regioselective synthesis of macrocyclesfrom allenes with a nucleophilic functionality and organic iodides. *Adv. Synth. Catal.* **355**, 3295-3303 (2013).
13. Demel, P., Keller, M. & Breit, B. *o*-DPPB-directed copper-mediated and -catalyzed allylic substitution with Grignard reagents. *Chem. Eur. J.* **12**, 6669-6683 (2006).
